# Nonsystematic Reporting Biases of the SARS-CoV-2 Variant Mu Could Impact Our Understanding of the Epidemiological Dynamics of Emerging Variants

**DOI:** 10.1093/gbe/evad052

**Published:** 2023-03-28

**Authors:** Mary E Petrone, Carolina Lucas, Bridget Menasche, Mallery I Breban, Inci Yildirim, Melissa Campbell, Saad B Omer, Edward C Holmes, Albert I Ko, Nathan D Grubaugh, Akiko Iwasaki, Craig B Wilen, Chantal B F Vogels, Joseph R Fauver

**Affiliations:** Department of Epidemiology of Microbial Diseases, Yale School of Public Health; Sydney Institute for Infectious Diseases, School of Medical Sciences, University of Sydney, NSW, Australia; Department of Immunobiology, Yale University School of Medicine; Department of Laboratory Medicine, Yale University School of Medicine; Department of Epidemiology of Microbial Diseases, Yale School of Public Health; Department of Epidemiology of Microbial Diseases, Yale School of Public Health; Department of Pediatric, Section of Infectious Diseases and Global Health, Yale University School of Medicine; Yale Institute for Global Health, Yale University; Department of Medicine, Section of Infectious Diseases, Yale University School of Medicine; Department of Epidemiology of Microbial Diseases, Yale School of Public Health; Yale Institute for Global Health, Yale University; Department of Medicine, Section of Infectious Diseases, Yale University School of Medicine; Sydney Institute for Infectious Diseases, School of Medical Sciences, University of Sydney, NSW, Australia; Department of Epidemiology of Microbial Diseases, Yale School of Public Health; Department of Medicine, Section of Infectious Diseases, Yale University School of Medicine; Department of Epidemiology of Microbial Diseases, Yale School of Public Health; Department of Ecology and Evolutionary Biology, Yale University; Department of Immunobiology, Yale University School of Medicine; Howard Hughes Medical Institute; Department of Immunobiology, Yale University School of Medicine; Department of Laboratory Medicine, Yale University School of Medicine; Department of Epidemiology of Microbial Diseases, Yale School of Public Health; Department of Epidemiology of Microbial Diseases, Yale School of Public Health; College of Public Health, University of Nebraska Medical Center

**Keywords:** SARS-CoV-2, phylogenetics, Mu, variant of concern, COVID-19

## Abstract

Developing a timely and effective response to emerging SARS-CoV-2 variants of concern (VOCs) is of paramount public health importance. Global health surveillance does not rely on genomic data alone to identify concerning variants when they emerge. Instead, methods that utilize genomic data to estimate the epidemiological dynamics of emerging lineages have the potential to serve as an early warning system. However, these methods assume that genomic data are uniformly reported across circulating lineages. In this study, we analyze differences in reporting delays among SARS-CoV-2 VOCs as a plausible explanation for the timing of the global response to the former VOC Mu. Mu likely emerged in South America in mid-2020, where its circulation was largely confined. In this study, we demonstrate that Mu was designated as a VOC ∼1 year after it emerged and find that the reporting of genomic data for Mu differed significantly than that of other VOCs within countries, states, and individual laboratories. Our findings suggest that nonsystematic biases in the reporting of genomic data may have delayed the global response to Mu. Until they are resolved, the surveillance gaps that affected the global response to Mu could impede the rapid and accurate assessment of future emerging variants.

**Significance**

The trajectory of the COVID-19 pandemic has been defined by the emergence of novel viral variants, and timely reporting of novel viral sequences is crucial for a timely response. In this study, we identified nonsystematic biases in the reporting of genomic data for the Mu variant that likely delayed its designation as a variant of concern. Identifying gaps in surveillance infrastructure will improve our collective ability to swiftly identify emerging SARS-CoV-2 variants.

## Introduction

Evaluating the clinical and epidemiological characteristics of novel SARS-CoV-2 variants soon after they emerge is of paramount global health importance. By the end of 2021, 13 variants of public health significance had been identified by the World Health Organization (WHO) (WHO 2021). Of these, the highly transmissible Alpha and Delta variants sequentially became dominant in many parts of the world (Davies et al. 2021; Lopez Bernal et al. 2021; Lucas et al. 2021; Mlcochova et al. 2021; Planas et al. 2021; Supasa et al. 2021; Wang et al. 2021; Allen et al. 2022), whereas variants characterized by immune evasive mutations resulting from amino acid substitutions in the spike protein gene were arguably less impactful (Bushman et al. 2021). The emergence and persistence of Omicron, which is both highly transmissible and capable of evading vaccine-induced immunity (Ferguson et al. 2021; Karim and Karim 2021; Collie et al. 2022; Garcia-Beltran et al. 2022), and the exponential rise in Omicron infections that ensued suggested that the combination of these factors conferred a higher level of viral fitness than was previously observed in the COVID-19 pandemic. However, definitive molecular correlates of variant fitness have yet to be identified, in part because the epidemiological context in which a variant emerges (e.g., population density and immunization status) also affects the growth rate of the variant.

Such a complex of factors means that other metrics are required to understand the potential impact of novel variants when they emerge. Soon after it was detected in late 2020, the transmissibility of Alpha (pango lineage B.1.1.7) relative to cocirculating lineages was estimated by comparing the growth rates of the number of sequences collected per week (Davies et al. 2021). In a similar fashion, Annavajhala et al. (2021) applied a multinomial regression model to sampling dates of publicly available genomic data on GISAID (gisaid.org) to measure the transmissibility of Iota (pango lineage B.1.526). More recently, methods have been developed to estimate the effective reproduction number (*R*_t_) for individual variants. Petrone et al. (2021) and Earnest et al. (2021) used reported case data and variant frequencies to infer the number of cases or infections that could be attributed to individual variants, while Figgins and Bedford (2022) incorporated both count and frequency data to assess the growth advantage of previous and current variants of concern (VOCs). In each instance, it was necessarily assumed that data for cocirculating variants in a given location were uniformly reported and that data reported prospectively captured *bona fide* trends in variant dynamics. As a consequence, the accurate assessment of the epidemiological dynamics of variants during the early stages of emergence requires uniform and timely reporting of genomic data for all circulating SARS-CoV-2 lineages.

In this study, we use the variant Mu (pango lineage B.1.621) to test the robustness of these assumptions. The lineage B.1.621 was first detected in Colombia in late 2020, and its emergence coincided with a rise in reported COVID-19 cases (Laiton-Donato et al. 2021). B.1.621 was briefly designated as a VOC (Mu) by the WHO in 2021 (WHO 2021). Three sublineages of B.1.621 emerged to comprise the Mu clade: B.1.621.1, B.1.621.2, and BB.2 (cov-lineages.org). None of the sublineages were designated as VOCs by the WHO. As of January 1, 2022, Mu clade genome sequences had been identified through sequencing in 52 countries (gisaid.org). Mu initially elicited concern due to a number of mutations associated with immune evasion. Specifically, substitutions (E484K, N501Y, and R346K) in the receptor-binding domain (RBD) of the spike gene are found in other VOCs including Beta, Gamma, and Omicron that have been shown to be less sensitive to neutralization by vaccine-induced antibodies (Uriu et al. 2021). Despite these characteristics and its association with rising cases in Colombia, Mu did not become dominant in countries outside of South and Central America, and minimal local transmission has been reported in other regions (Messali et al. 2021; Mio et al. 2021). Importantly, however, its designation as a VOC occurred 3 months after that of Delta, which had already begun to rise to dominance globally (McCrone et al. 2022). Herein, we investigate the reasons for the ostensible delay in the global response to Mu and evaluate the consistency of reporting patterns of variants within and between geographic locations.

## Results

### Delayed Global Response to the SARS-CoV-2 Variant Mu

**Fig. 1.**
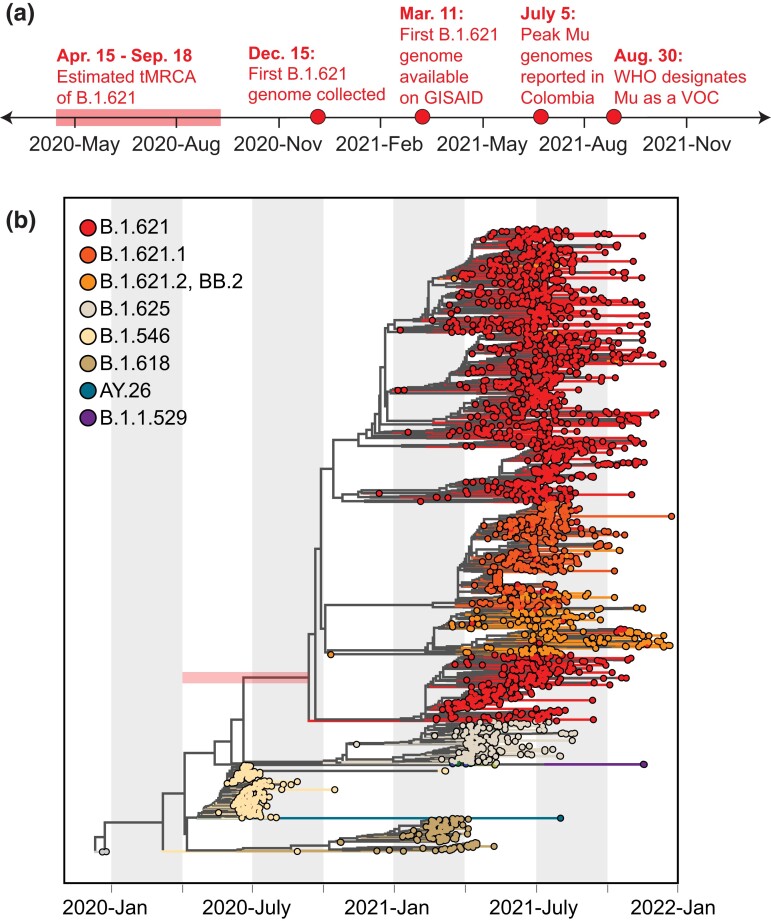
—Delayed global response to Mu. (*a*) Timeline of events for the emergence and response to Mu. (*b*) Time-resolved phylogenetic tree inferred using TreeTimev1.6.3 (Sagulenko et al. 2018). The estimated tMRCA range for Mu is indicated by the horizontal bar.

The designation of Mu as a VOC in August 2021 occurred ∼1 year after its associated lineage—B.1.621—likely emerged (fig. 1*a*). The clinical sample containing the first sequenced B.1.621 genome was collected on December 15, 2020, in Bogota, Colombia (table 1). However, the detection of the sublineage BB.2 predates that of B.1.621 by 2 months (October 2020, in Antioquia, Colombia), indicating that B.1.621 was circulating and evolving before December 15. In further support of this hypothesis, international spread of B.1.621 and sublineages was documented in December 2020 and January 2021. A sample containing B.1.621.1 was sequenced in December in Florida, USA, and B.1.621 was detected in Venezuela on January 21, 2021.

**Table 1:** Mu and B.1.621 Sublineages Likely Emerged in the Americas in mid-2020

Lineage	Collection Date	Submission Date	Location Collected
B.1.621	Dec. 15, 2020	Dec. 18, 2021	Bogota, Colombia
B.1.621.1	Dec. 2020^a^	July 19, 2021	Florida, USA
B.1.621.2	May 2, 2021	Aug. 13, 2021	Cesar, Colombia
BB.2	Oct. 14, 2020	Nov. 19, 2021	Antioquia, Colombia

Note.—“Submission date” indicates the date on which the genome became publicly available on GISAID.

a Only month and year were specified on GISAID.

To quantify the delay between emergence and VOC designation, we used phylogenetic analysis to infer that B.1.621 likely emerged by September 2020, but as early as April of the same year (i.e., the 95% credible intervals on the time to the most recent common ancestor [tMRCA] range from April 15, 2020, to September 18, 2020) (fig. 1*b*). The sublineages B.1.621.1, B.1.621.2, and BB.2 emerged shortly thereafter in 2020. Thus, B.1.621 was designated as a VOC between 11 and 16 months after it emerged, by which time three sublineages had evolved and collectively spread to at least three countries. In contrast, Delta and Omicron were designated as VOCs 7 (McCrone et al. 2022) and 2 (Viana et al. 2022) months after their estimated emergence, respectively.

### Limited Global Spread of Mu and Sublineages

**Fig. 2.**
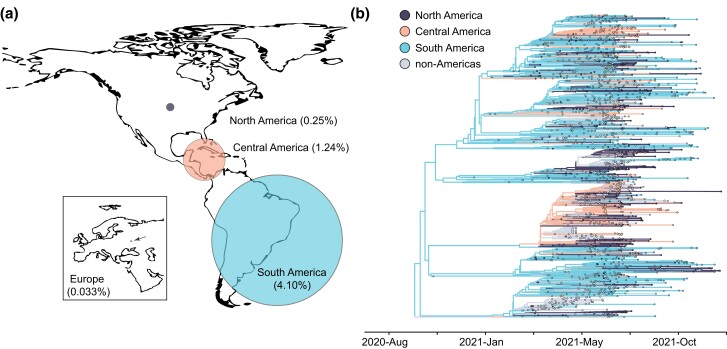
—Mu transmission and evolution primarily occurred in Central and South America. (*a*) Percentage of Mu genomes submitted to GISAID as of December 31, 2021. (*b*) Geographically-resolved Mu clade (shown in fig. 1*b*). The geographic location for internal nodes was inferred using the Markov chain Monte Carlo method in BEAST v.1.10.5. The majority of genome sequences designated as “non-Americas” were collected in Europe.

This delay in detection was particularly concerning because Mu is characterized by mutations associated with immune evasion (supplementary fig. S1, Supplementary Material online) (Lucas et al. 2021; Uriu et al. 2021). We hypothesized that the delay in detection combined with its immune-evasive properties would have enabled the Mu clade to establish an intercontinental circulation pattern. However, a preliminary assessment of the global distribution of sequenced Mu genomes suggested that, despite its low neutralization titer, Mu did not spread substantially beyond the Americas, comprising <1% sequenced genomes in all but Central and South America (fig. 2*a*). Only ten countries reported at least 100 Mu genomes between October 2020 and January 2022 (supplementary table S1, Supplementary Material online). In all cases, Mu frequencies peaked between June and August 2021 (supplementary fig. S2, Supplementary Material online), but only in two (Colombia and Dominican Republic) did Mu comprise more than half of sequenced genomes in a given week (supplementary fig. S2*a*, Supplementary Material online).

We next extracted the Mu clade from our time-resolved phylogenetic tree shown in figure 1 and inferred geographic transitions using a Bayesian discrete phylogeographic approach implemented in BEAST (Suchard et al. 2018; Dellicour et al. 2021). For simplicity, we assigned genome sequences to one of four categories: North America (*n* = 376), Central America (*n* = 342), South America (*n* = 698), and non-Americas (*n* = 538) (fig. 2*b*). The majority of the genome sequences classified as “non-Americas” were collected in Europe, and the remaining 31 were collected in Asia.

Consistent with the global distribution of this variant, the evolution and spread of the Mu clade were largely confined to South and Central America (fig. 2*b*), with South America as the likely location of emergence. Few instances of reintroductions from North America or Europe to South America were observed, indicating that Mu exhibited a unidirectional pattern of spread, radiating out from South America. Differences between the composition of global and local SARS-CoV-2 populations could explain this pattern. Unlike countries in North America and Europe, Colombia did not experience a large Alpha wave, which may have enabled the establishment of the Mu clade through founder effect. Where a perceptible rise in Mu genome frequencies could be observed in North America, the concurrent emergence of Delta likely curtailed further spread of Mu.

### Nonsystematic Reporting Patterns Affected Genomic Data Availability for Mu

Fortunately, the delayed response to Mu did not result in widespread and undetected circulation of an immune-evasive variant. However, this may not be the case for future variants. Given the importance of rapidly responding to the emergence of novel SARS-CoV-2 variants, we investigated whether the availability of genomic data at the time Mu emerged could have influenced decisions pertaining to its designation.

**Fig. 3.**
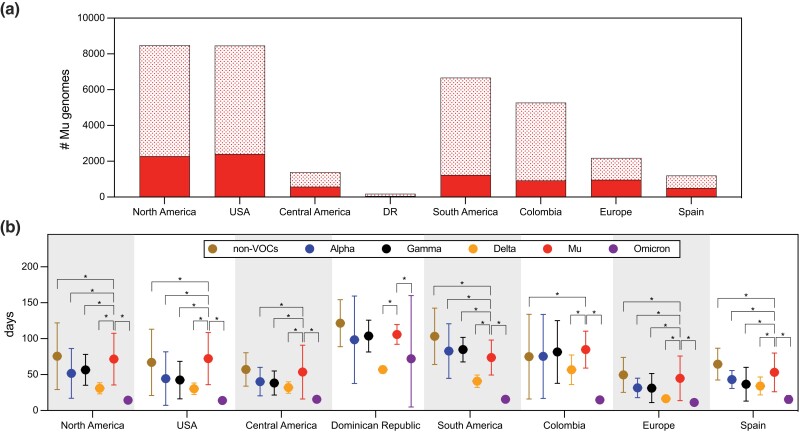
—Heterogeneity in reporting practices among VOCs. (*a*) Number of Mu genomes reported by continent and country as of August 31, 2021 (solid) and by the end of 2021 (solid and checked). In the Dominican Republic (DR), 52 of 126 genomes were publicly available on August 31, 2021. (*b*) Mean, upper limit, and lower limits for submission delays for individual variants. For non-VOCs, the mean time to submission was calculated using genomes sequenced in 2021. A Welch two-sample *t*-test with Bonferroni adjustment was used to compare means. * indicates adjusted *P* < 0.05. Exact *P* values available in supplementary material S1, Supplementary Material online.

The analyses in figures 1 and 2 were performed retrospectively using genomic data that were publicly available through January 2022, 4 months after Mu peaked globally (supplementary fig. S2, Supplementary Material online). We therefore assessed trends in data availability in Colombia, which reported the earliest rise in the frequency of Mu and the largest number of Mu genomes per capita (102.4 genomes per 1 million population; supplementary table S1, Supplementary Material online). When the WHO made the VOC designation in August 2021, only ∼21% of Mu genomes collected in Colombia through August 31 were publicly available (fig. 3*a*). This number was within the range of other countries in South America reporting Mu genomes (9.5% [Peru] to 47.7% [Ecuador]; supplementary table S2, Supplementary Material online). Indeed, the sharp increase in the number of sequenced Mu genomes that can now be observed in Colombia in July was not apparent until those data became available by the end of the year (supplementary fig. S3*a*, Supplementary Material online). A steady increase in the frequency of Mu genomes was observable in June concurrent with rising incident cases, but these frequencies were calculated from a small number of reported Mu genomes per week (mean = 41, range = 1–120) (supplementary fig. S3*b*, Supplementary Material online), rendering interpretation difficult. To assess whether trends in available count or frequency data at the start of the Mu wave were predictive of the trends that emerged once all data were available, we performed Granger causality tests. Count data available in June were significant in Granger causality count data for that time period that became available by the end of the year (*P* < 0.05; supplementary table S3, Supplementary Material online). No significant relationships were observed between frequency time series.

To evaluate whether the delay in data availability for Mu genomes in Colombia was consistent with that of other VOCs in other countries, we measured the time to submission for four current or previously designated VOCs (Alpha, Gamma, Delta, and Omicron) and non-VOCs/variants of interest (VOIs). We defined “time to submission” as the time elapsed between sample collection and the date on which the data became publicly available on GISAID (gisaid.org). We excluded continents that reported fewer than 100 Mu genomes (Africa, Asia, and Oceania) and selected the country that reported the highest number of Mu genomes on each continent. Data reporting for Mu genomes occurred at a slower rate than other VOCs. Time to submission for VOCs generally decreased over time within each region, with earlier variants such as Alpha and Gamma exhibiting longer intervals and the most recent variant Omicron exhibiting the shortest interval across all locations except the Dominican Republic (fig. 3*b*). In every location, however, the median time to submission for Mu genomes was significantly longer than that of Delta and Omicron, the VOCs that preceded and followed it (fig. 3*b*, supplementary material S1, Supplementary Material online).

We tested whether these differences could be attributed to changing genomic surveillance infrastructures as the pandemic progressed. As a case study, we compared reporting practices in the state of Connecticut, USA, where Mu and Delta cocirculated in June and July 2021 (supplementary fig. S4, Supplementary Material online). We hypothesized that reporting should be uniform across variants during this period, but we instead found that the mean time to submission of Mu genomes was more than twice that of Delta genomes across the state (61.8 days vs. 19.9 days; supplementary fig. S5, Supplementary Material online). This pattern was due in part to differences in the practices of submitting laboratories. Together, the Grubaugh Laboratory (authors) and the Centers for Disease Control and Prevention (CDC) contributed more than half of publicly available genomes in Connecticut in 2021 (Grubaugh = 29.4%, CDC = 34.7%). The mean time to submission only differed by 13.7 days for Mu and Delta genomes collected by the Grubaugh Laboratory (adjusted *P* < 0.05; supplementary Data, Supplementary Material online) but by 67.6 days for Mu genomes collected by the CDC (adjusted *P* < 0.05; supplementary Data, Supplementary Material online). Mean time to submission did not differ by comparable margins between labs for other VOCs. These data indicate that factors other than timing and resource availability delayed the publication of Mu genomes, and reporting delays occurred across geographic scales, ranging from continents to individual laboratories.

## Discussion

We present evidence that the timing of the WHO decision to designate Mu as a VOC was influenced by limited and delayed genomic data availability for this variant. The designation of the lineage B.1.621 as a VOC (Mu) occurred nearly a year after this variant likely emerged, and this delay may be attributed to significantly longer submission times to the public repository GISAID compared with other VOCs. By the end of August 2021, less than one quarter of the genomes from samples collected by that time in Colombia were made publicly available. This discrepancy was also observed in other countries reporting Mu genomes. Overall, time to submission decreased as the pandemic progressed, with Omicron genomes being submitted in the mean shortest amount of time, but global reporting patterns for Mu notably deviated from this trend. This observation demonstrates that reporting patterns for SARS-CoV-2 variants were nonuniform between variants and within submitting laboratories.

Initial challenges associated with publishing B.1.621 genome sequences on the public repository GISAID (gisaid.org) could have contributed to the delay in its designation. B.1.621 contains a 4-nucleotide deletion in ORF3a, which causes a frameshift mutation resulting in a premature stop codon and a truncated protein (supplementary fig. S1, Supplementary Material online). As a public repository, GISAID has necessarily stringent quality control measures to identify consensus genome sequences that may contain sequencing errors, and deletions causing frameshift mutations resulting in premature stop codons were considered to be sequencing and/or bioinformatic errors. Because genuine frameshift mutations were uncommon, they were flagged as sequencing errors, the submitters were notified, and the virus genome sequences in question were not made publicly available. It was therefore the responsibility of the submitter to review rejected sequences for possible errors before resubmitting their data. In overwhelmed or limited resource research settings, rejected genome sequences may not be reviewed for extended periods of time, thereby delaying the identification of new lineages with *bona fide* frameshift mutations.

The issue of deletions causing frameshift mutations resulting in premature stop codons did arise with B.1.621 and resulted in genomes being flagged and not made immediately publicly available. To fully address the impact of this delay, future studies should compare rejection rates across submitting laboratories for the duration of the pandemic. Although this may explain why time to submission of Mu genomes differed significantly than that of cocirculating VOCs even within submitting laboratories (e.g., CDC), other VOCs, notably Alpha, also contained premature stop codons (ORF 8). However, in the case of Alpha, the premature stop codon was due to a single nucleotide polymorphism (SNP) as opposed to a deletion. GISAID has subsequently streamlined the quality control process by adding multiple confirmation options allowing submitters to verify that consensus sequence genomes with frameshift mutations are genuine prior to submission. Similar but unanticipated challenges may arise with future variants, and it is critical that submitting laboratories monitor their data submission process to avoid introducing nonsystematic biases into publicly available data.

Addressing these biases is especially important if methods aimed at comparing emerging variants are to be successfully employed. The *R*_t_ estimation methods presented by Petrone et al. (2021), Earnest et al. (2021), and Figgins and Bedford (2022) are robust to systematic biases in data for cocirculating variants. However, our study demonstrates that nonsystematic biases should also be considered. Without this, the validation of *R*_t_ estimation methods using retrospective (i.e., complete) data sets may not accurately reflect their performance when prospective (i.e., incomplete) data sets are used in a “real-time” scenario. Relatedly, identifying the time at which prospectively collected data can be assumed to represent “true” variant dynamics will differ between variants and geographic locations. For example, the mean time to submission for Delta was 16.5 days in Europe, but 40.8 days in South America (fig. 3*b*). Therefore, comparative studies that assume uniform reporting delays between locations could result in inaccurate inferences.

Our study was not without limitations. The phylogeographic analysis presented comprised substantially downsampled data, potentially biasing our findings. This challenge is in part due to the retrospective nature of our study, and downsampling is less likely to be needed when a more recently emerged variant is the subject of analysis, but, for the reasons stated above, the conclusions may be less reliable. These data may also have been subject to nonsystematic reporting biases. Because of the challenges associated with reporting Mu genome sequences, our phylogeographic inference may overly weight countries with robust reporting of Mu infections. Additionally, time to submission estimates may be influenced by outliers in countries that reported relatively small numbers of Mu genome sequences (e.g., Dominican Republic). To mitigate this, we removed differences that were <0 (indicating an error in the collection date provided) and >365 days. The observation that time to submission of Mu genome sequences was consistently longer than that of Delta and Omicron in all locations supports the qualitative accuracy of these estimates.

In response to the pandemic, an unprecedented number of SARS-CoV-2 genomes have been sequenced and made publicly available. However, significant gaps and biases in these data persist, signifying vulnerabilities in the global health response system and presenting challenges for computational methods that leverage these data. Fortunately, Mu did not establish global circulation, but delays in the response to future variants may result in more severe consequences. Novel variants that circulate undetected in regions with limited genomic surveillance pose a particular threat. Improving the global health response to future emerging SARS-CoV-2 variants must involve providing additional support to underresourced areas. The genetic composition of SARS-CoV-2 lineages and rapid changes in their frequency are early indicators of an emerging VOC. As much as possible, genomic data should be made publicly available to facilitate the rapid detection of new variants. At the same time, we recognize that there are many valid reasons why some researchers are hesitant to share their data prior to publication (Brito et al. 2021; Maxmen 2021). Similarly, errors in sequencing data could obfuscate genomic warning signs in addition to introducing inaccuracies in phylogenetic analyses and *R*_t_ estimates. It is therefore of utmost importance that individuals generating genomic data do so with high levels of accuracy. The WHO has provided extensive quality control standards and implementation guidelines to ensure high-quality SARS-CoV-2 genomic data (World Health Organization, 2021). As the circulating SARS-CoV-2 population continues to evolve, evaluating the response to variants that came before can help us prepare for variants that will emerge in the future.

## Materials and Methods

### Ethics

This study was approved by the Yale Human Research Protection Program Institutional Review Board (IRB Protocol ID 2000028924). Informed consent was obtained from all enrolled vaccinated healthcare workers (HCWs).

### Sampling Mu Clade Genome Sequences

We first selected all complete Mu genome sequences available on GISAID collected through December 31, 2021 (*n* = 14,477), as of January 8, 2022. For countries or territories with ≥50 genome sequences during our study period, we began by tabulating the weekly incident cases for each using the Johns Hopkins dashboard (https://github.com/CSSEGISandData/COVID-19) between October 2020 and December 2021. We normalized weekly incident cases per 1 million persons in the population. Next, we calculated the proportion of cases each location contributed to the total per week. We then multiplied this proportion by the number of available genome sequences per week per location to obtain the total number of genome sequences to sample (*n* = 1,505). We did not downsample genome sequences from countries or territories with ≤50 genome sequences during our study period (*n* = 452). To improve our phylogenetic inference, we included genome sequences from closely related lineages (B.1.546, B.1.616, and B.1.625) as well as VOCs (Alpha, Beta, Gamma, Delta, and Omicron) (supplementary table S4, Supplementary Material online). For the former, we selected all complete genome sequences with high coverage collected during our study period available on GISAID. For the latter, we randomly selected one to two complete genome sequences. We rooted the tree using a reference genome sequence collected in Wuhan in 2019 (Wuhan/WH01/2019).

### Phylogenetic Tree Estimation

Once sequences were downloaded from GISAID (supplementary table S4, Supplementary Material online), we aligned our genome sequences with MAFFT using GenBank accession MN908947.3 as the reference sequence. We removed gaps and masked known problematic sites (De Maio et al. 2021). We estimated maximum likelihood trees (figs. 1*b* and 3*b*) using IQ-TREE v1.6.3 employing a GTR + Γ substitution model and 1,000 bootstrap replications. To obtain temporal resolution for figure 3*b*, we used the maximum likelihood tree and corresponding alignment as input for augur (TimeTree).

### Phylogenetic Tree Visualization

Both phylogenetic trees (figs. 1*b* and 3*b*) were visualized using “baltic” implemented in Python v.3.8.3.

### Measuring Variant Submission Lags

We measured the time elapsed between the date collected and date submitted (i.e., made publicly available) to GISAID for each variant category in each geographic location. Differences < 0 or >365 were assumed to be due to typographical errors in the data and were removed. We then calculated the mean, upper limit, and lower limit for each variant. To test for significance, we performed Welch two-sample *t*-test and used the Bonferroni adjustment to correct the resulting *P* values for multiple comparisons. All analyses were performed in R v.4.1.2.

### Phylogeographic Inference

We extracted the Mu clade from our time-resolved tree inferred with TimeTree (fig. 3*b*). For discrete phylogeographic reconstruction, we assigned tips to one of four categories: “North America,” “Central America,” “South America,” and “non-Americas.” Genome sequences assigned to “non-Americas” were primarily collected in Europe (*n* = 507). The remaining 31 were collected in Asia. Tips for which a complete date was not provided with the GISAID submission were assigned to the 15th of the month. We performed phylogeographic reconstruction implemented in BEAST v1.10.5 using an asymmetric CTMC model for discrete state reconstructions and running for 1 million states 32. We used Tracer v.1.7.1 to assess convergence and determined that all ESS values reached at least 200.

### Granger Causality Test

Using the data presented in supplementary figure S3, Supplementary Material online, we assessed Granger causality between counts and frequencies of Mu genomes. Combinations and precise *P* values are shown in supplementary table S3, Supplementary Material online. We performed this analysis in R v.4.1.2 with the package “lmtest.”

## Supplementary Material

evad052_Supplementary_Data

## Data Availability

All genomic data used in this manuscript are publicly available via GISAID. Accession numbers for all SARS-CoV-2 genomes used in this study can be found in the GISAID_Acknowledgement_Table.pdf file in the supplemental materials.
