# Supplementary material for: Nonsystematic Reporting Biases of the SARS-CoV-2 Variant Mu Could Impact Our Understanding of the Epidemiological Dynamics of Emerging Variants
Source: Genome Biol Evol. 2023 Mar 28;15(4):evad052. doi: 10.1093/gbe/evad052 (PMC10113931; doi:10.1093/gbe/evad052)
Supplement: evad052_Supplementary_Data [file evad052_supplementary_data.zip › Mu_supplement_v3.docx]

**
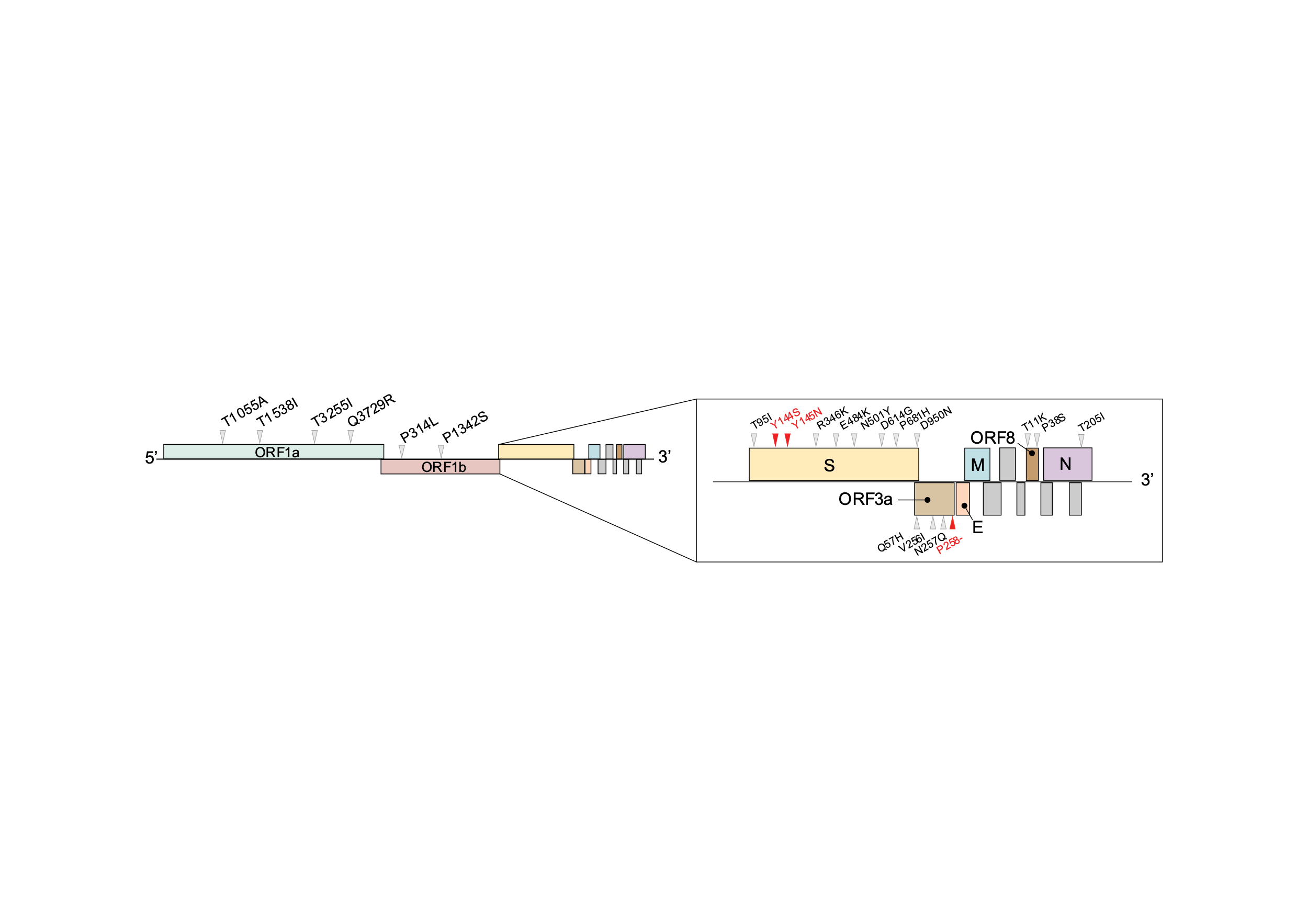
**

**Figure S1:** Schematic of SARS-CoV-2 genome with Mu clade defining mutations marked. The substitutions Y114S and Y145N (red) are the result of a 3bp insertion in the N-terminal domain. P258*, the premature stop codon resulting from a frameshift in ORF3a, is also shown in red.

**Table S1:** Publicly available Mu genomes for countries with the highest reported number of Mu genomes

| Country | # Mu genomes sequenced | # Mu genomes per 1M population | Week of peak reported Mu genome frequency (% total genomes) |
| --- | --- | --- | --- |
| USA | 6247 | 19.0 | 14/6/2021 (3.7) |
| Colombia | 5212 | 102.4 | 5/7/2021 (87) |
| Spain | 961 | 50.3 | 31/5/2021 (5.1) |
| Chile | 712 | 15.0 | 23/8/2021 (25) |
| Mexico | 491 | 27.8 | 5/7/2021 (6.9) |
| Ecuador | 443 | 3.4 | 9/8/2021 (45) |
| Canada | 271 | 8.2 | 9/8/2021 (0.7) |
| Peru | 163 | 4.3 | 2/8/2021 (7.3) |
| Dominican Republic | 129 | 11.9 | 21/6/2021 (89) |
| United Kingdom | 113 | 1.7 | 28/6/2021 (0.09) |


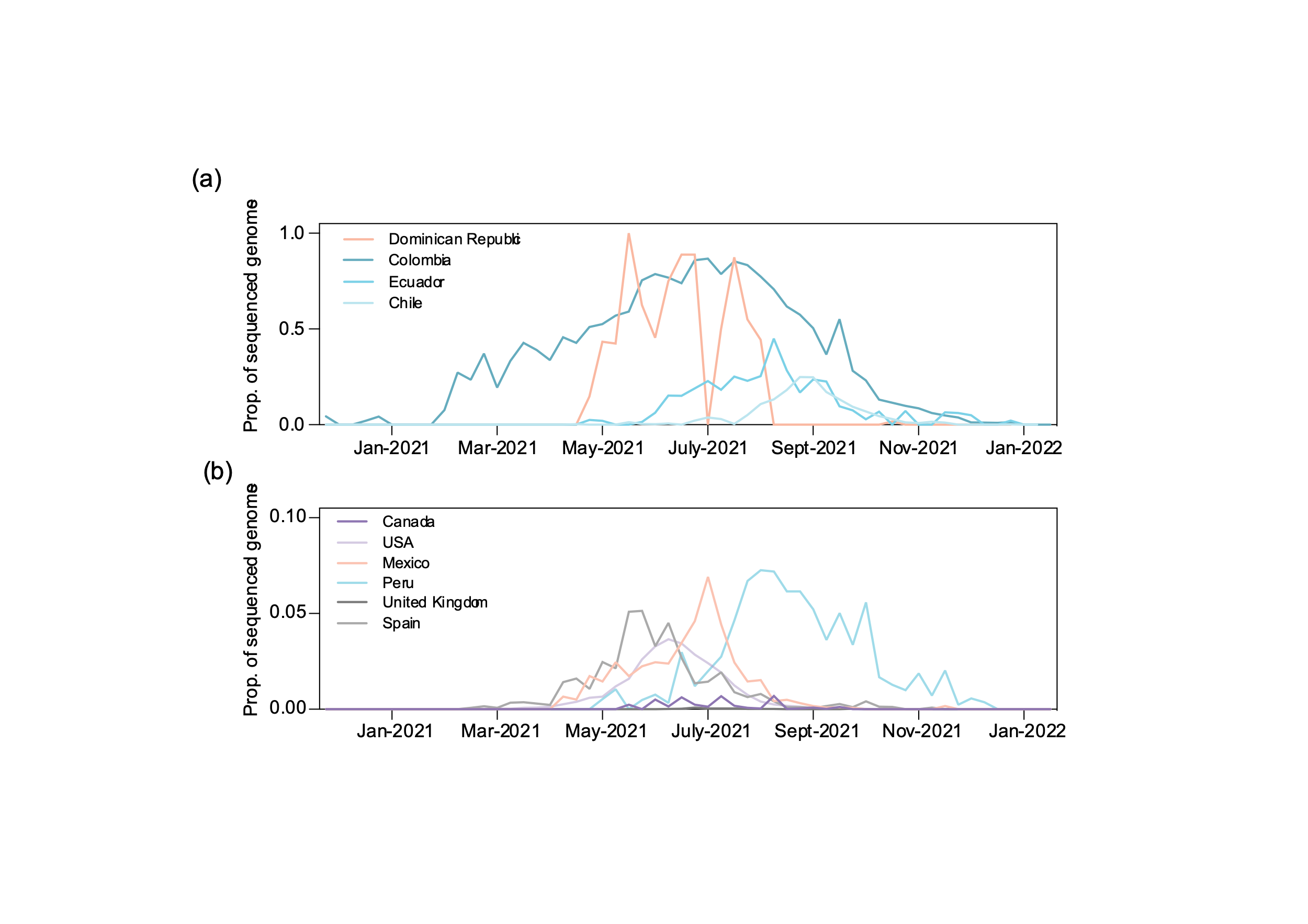


**Figure S2:** Weekly frequencies of reported Mu genomes in countries where Mu exceeded 10% of weekly sequenced genomes (a) and in countries where Mu did not exceed 10% of weekly sequenced genomes (b).

**Table S2: Percentage of Mu genomes made publicly available in South American countries reporting at least 100 Mu genomes**

| **Country** | **% total Mu genomes collected by August 31, 2021 that were publicly available on that date** |
| --- | --- |
| Colombia | 21.1 |
| Chile | 16.6 |
| Ecuador | 47.7 |
| Peru | 9.5 |

**Figure S3:** Delayed reporting of Mu genomes in Colombia. Genomic counts (a) and frequencies (b) of Mu genomes in Colombia based on data submitted to GISAID by month between June and December 2021.

**Table S3:** Results of Granger causality test of Mu genome counts and frequencies reported in Colombia. Significant p-values (p < 0.05) are italicized.

| **x** | **y** | **p-value** |
| --- | --- | --- |
| counts_June | counts_August | *0.003217* |
| counts_June | frequencies_August | 0.7526 |
| frequencies_June | counts_August | 0.7302 |
| frequencies_June | frequencies_August | 0.9109 |
| counts_June | counts_December | *0.005333* |
| counts_June | frequencies_December | 0.528 |
| frequencies_June | frequencies_December | 0.9726 |
| frequencies_June | counts_December | 0.385 |


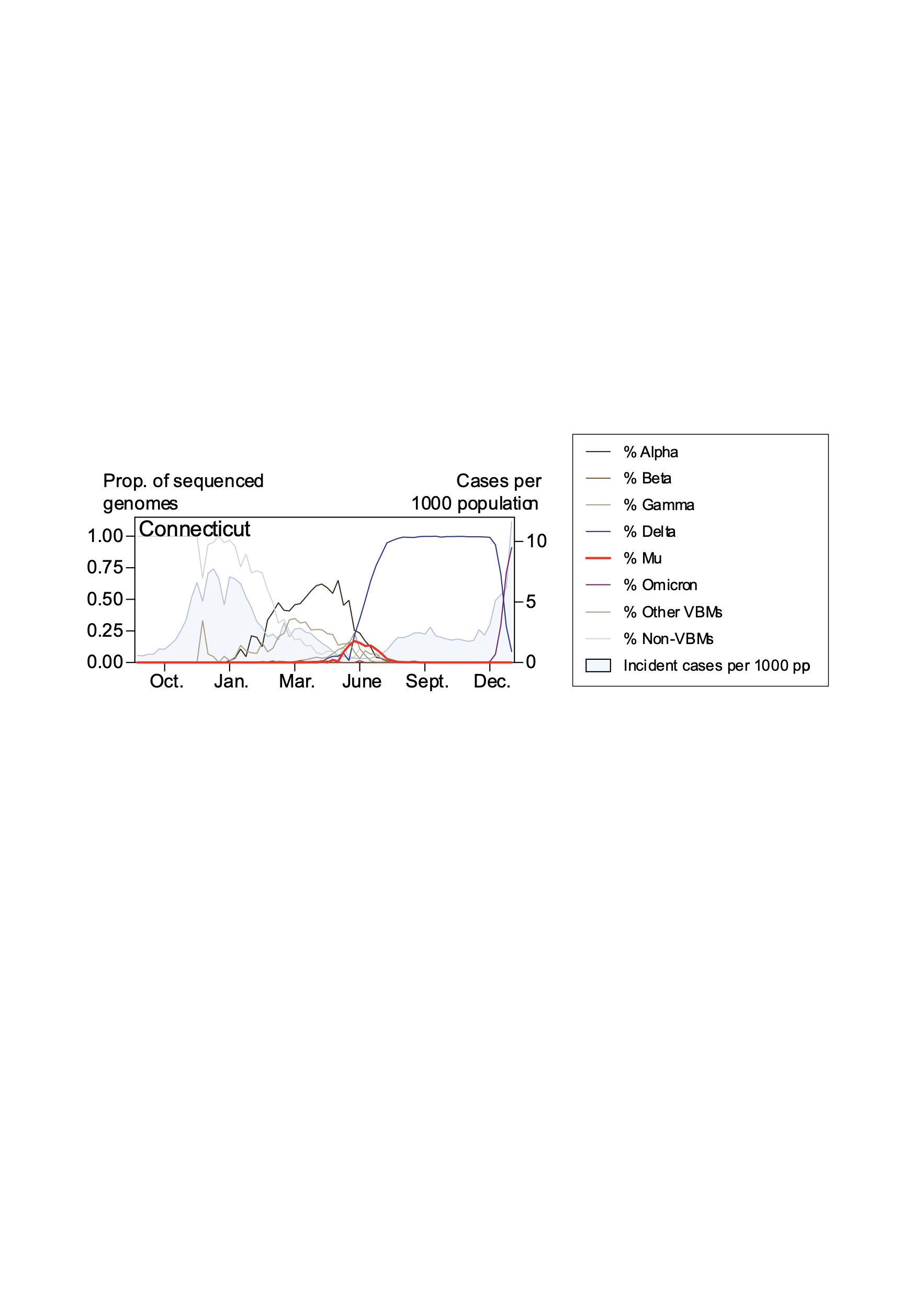


**Figure S4:** Genomic frequencies of variants in Connecticut, USA. Case data were retrieved from the Johns Hopkins COVID-19 Data Repository (<https://github.com/CSSEGISandData/COVID-19>). We downloaded all metadata that were submitted to GISAID by January 19, 2022. Entries for which only the collection year was provided were removed. Entries for which only the month were provided were assigned to the 15^th^. We further filtered our dataset to include genome sequences collected between October 1, 2020, and December 31, 2021. We tabulated the number of each variant category collected by week and reported frequencies as a proportion of the total number of genome sequences collected by week by location. All analyses were performed in R v.4.0.2.

**Figure S5:** Time to submission mean (filled circle), upper limit (top bar), and lower limit (bottom bar) for circulating variants in Connecticut.

**Table S4:** Lineage distribution for Figure 1b.

| Lineage | Count |
| --- | --- |
| Mu | 1957 |
| B.1.621 | 1373 |
| B.1.621.1 | 378 |
| B.1.621.2 | 62 |
| BB.2 | 144 |
| VOCs (Alpha, Beta, Gamma, Delta, Omicron) | 9 |
| B.1.546 | 211 |
| B.1.618 | 132 |
| B.1.625 | 167 |
| References | 2 |
| *Total* | *2478* |
