# Supplementary material for: Nonsystematic Reporting Biases of the SARS-CoV-2 Variant Mu Could Impact Our Understanding of the Epidemiological Dynamics of Emerging Variants
Source: Genome Biol Evol. 2023 Mar 28;15(4):evad052. doi: 10.1093/gbe/evad052 (PMC10113931; doi:10.1093/gbe/evad052)
Supplement: evad052_Supplementary_Data [file evad052_supplementary_data.zip › GISAID_Acknowledgement_Table.pdf]

We gratefully acknowledge the following Authors from the Originating laboratories responsible for obtaining the specimens, as well as the Submitting laboratories where the genome data were generated and shared via GISAID, on which this research is based.

All Submitters of data may be contacted directly via www.gisaid.org

Authors are sorted alphabetically.

Acknowledgement EPI_SET Identifier: EPI_SET_20220328ct

Accession ID	Originating Laboratory	Submitting Laboratory	Authors
EPI_ISL_7987914	4Cyte Pathology	NSW Health Pathology - Institute of Clinical Pathology and Medical Research; Westmead Hospital; University of Sydney	Arnott A.; Draper J.; Gall M.; Martinez E.; Rockett R.; Sintchenko V.; on behalf of ICPMR
EPI_ISL_2345429	AMBULATORIO DE ESPECIALIDADE V E MOGI MIRIM	Instituto Butantan / ESALQ-Piracicaba	Antonio Jorge Martins; Claudia Renata dos Santos Barros; David Schlesinger; Debora Botequio Moretti; Dimas Tadeu Covas; Elaine Cristina Marqueze; Elaine Vieira Santos; Evandra Strazza Rodrigues; Heidge Fukumasu; Jayme Augusto de Souza-Neto; José Salvatore Leister Patané; Luiz Alcantara; Luiz Lehmann Coutinho; Maria Carolina Elias; Maurício Lacerda Nogueira; Rafael dos Santos Bezerra; Raul Machado Neto; Rejane Maria Tommasini Grotto; Ricardo Haddad; Sandra Coccuzzo Sampaio Vessoni; Simone Kashima; Svetoslav Nanev Slavov; Vincent Louis Viala
EPI_ISL_1686547, EPI_ISL_1996455, EPI_ISL_2181206, EPI_ISL_2181383, EPI_ISL_3430367, EPI_ISL_3845629, EPI_ISL_3853010, EPI_ISL_4186127, EPI_ISL_4360363, EPI_ISL_4397844, EPI_ISL_8082202			
see above	Aegis Sciences Corporation	Centers for Disease Control and Prevention Division of Viral Diseases, Pathogen Discovery	Adrian Paskey; Alec Vest; Benjamin Rambo-Martin; Christopher Gulvick; Clinton Paden; Clinton R. Paden; Cyndi Clark; Dakota Howard; Darlene Wagner; Dhwani Batra; Dillon Nall; Duncan MacCannell; Erisa Sula; Ethan Sanders; Holly Houdeshell; Jason Caravas; Kara Moser; Kristine Lacek; Matthew Hardison; Matthew Schmerer; Ola Kvalvaag; Patrick Campbell; Peter Cook; Peter W. Cook; Rob Case; Scott Sammons; Shatavia Morrison; Shaun Westlund; Tymeckia Kendall; Victoria Caban Figueroa; Vikramsinha Ghorpade; Yvette Unoarumhi
EPI_ISL_5433572	Akademiska Sjukhuset, Clinical Microbiology and Hospital Hygiene	Uppsala University Hospital	Jonathan Haars; Julia Bergholm; Patrik Ellström; Rene Kaden; Steinar Mannsverk
EPI_ISL_1823038	Altius Institute	Seattle Flu Study	Alex Nguyen; Amanda Adler; Andrew Meuser; Barry R. Lutz; Benjamin Pelle; Caitlin R. Wolf; Chris D. Frazar; Clem Green; Daniel Bates; Deborah A. Nickerson; Elisabeth Brandstetter; Erica Ryke; Hannah Petersen; Helen Y. Chu; Jacob Rodriguez; Janet A. Englund; Jay Shendure; Jessica Halow; John Stamatoyannopoulos; Joshua Richards; Jover Lee; Julia Wald; Kairsten Fay; Kirsten Lacombe; Kneshay Harper; Lea M. Starita; Mark J. Rieder; Matt Hartman; Matthew Richardson; Matthew Thompson; Melissa Truong; Michael Boeckh; Michael Famulare; Misja Ilcisin; Muhammad Halimun; Olivia Waltner; Peter D. Han; Rebecca Bruders; Ryan Alexander; Sadie Patraw; Sofia Olsson; Stephanie DeBaun; Thomas R. Sibley; Tobias Ragoczy; Trevor Bedford; Truong Nguyen
EPI_ISL_2438065	Ayudas diagnosticas SURA	Universidad Nacional de Colombia - Laboratorio Genómico One Health	Andres F. Cardona-Rios; Carlos Franco-Muñoz; Carolina Muñoz-Arango; Celeny Ortiz; Daniel O. Maldonado-Perez; Diego A. Álvarez-Díaz; Hector Alejandro Ruiz-Moreno; Idabely Betancur Ortiz; Jorge E. Osorio; Juan P. Hernandez-Ortiz; Karl A Ciuoderis; Katherine Laiton-Donato; Laura Silvana Perez; Lina M. Hurtado; Marcela Mercado-Reyes; Maria Angélica Maya; Maria Stella López; Rita Almanza Payares; Sandra Ines Cano; Simón Villegas Velásquez
EPI_ISL_2528253, EPI_ISL_2721964	BCCDC Public Health Laboratory	BCCDC Public Health Laboratory	Ana Pacagnella; Corrinne Ng; Dan Fornika; John Tyson; Kim Macdonald; Kimia Kamelian; Linda Hoang; Loretta Janz; Mel Krajden; Prystajecky Natalie; Robert Azana; Shannon Russell
EPI_ISL_5648490, EPI_ISL_7779341	BIOFAST	Instituto Butantan	Antonio Jorge Martins; Claudia Renata dos Santos Barros; David Schlesinger; Debora Botequio Moretti; Dimas Tadeu Covas; Elaine Cristina Marqueze; Elaine Vieira Santos; Evandra Strazza Rodrigues; Heidge Fukumasu; Jayme Augusto de Souza-Neto; José Salvatore Leister Patané; Luiz Alcantara; Luiz Lehmann Coutinho; Maria Carolina Elias; Maurício Lacerda Nogueira; Rafael dos Santos Bezerra; Raul Machado Neto; Rejane Maria Tommasini Grotto; Ricardo Haddad; Sandra Coccuzzo Sampaio Vessoni; Simone Kashima; Svetoslav Nanev Slavov; Vincent Louis Viala
EPI_ISL_2933472	BIOMNIS LYON	CNR Virus des Infections Respiratoires - France SUD	Antonin Bal; Bruno Lina; Gregory Destras; Gwendolyne Burfin; Hadrien Regue; Laurence Josset; Martine Valette; Quentin Semanas
EPI_ISL_1157306, EPI_ISL_1357210	Bayerisches Landesamt für Gesundheit und Lebensmittelsicherheit (LGL)	Robert Koch Institute	
EPI_ISL_6619025, EPI_ISL_7893435, EPI_ISL_8086551	Berkshire and Surrey Pathology Services Lighthouse Laboratory	Wellcome Sanger Institute for the COVID-19 Genomics UK (COG-UK) Consortium	Berkshire and Surrey Pathology Services Lighthouse Laboratory and Alex Alderton; Cordelia Langford; David K. Jackson; Dominic Kwiatkowski; Ewan Harrison; Ian Johnston; Jeffrey Barrett; John Sillitoe on behalf of the Wellcome Sanger Institute COVID-19 Surveillance Team; Roberto Amato; Sonia Goncalves
EPI_ISL_6809523	BioneXt Lab	Laboratoire national de sante, Microbiology, Microbial Genomics Platform	Anke Wienecke-Baldacchino; Catherine Ragimbeau; Elodie Solarino; Fatu Djabi; Jessica Tapp; Lise Pignon; Raoul Salmon; Tamir Abdelrahman; Thibault Ferrandon; Virginie Jover
EPI_ISL_5502548	Botswana Harvard HIV Reference Laboratory	Botswana Harvard HIV Reference Laboratory	Boitumelo J. L Zuze; Botshelo Radibe; Dorcas Maruapula; Joseph Makhema; Keoratile Ntshambiwa; Kgomotso Moruisi; Legodile Kooepile; Letsibogo Gaoraelwe; Mosepele Mosepele; Mphaphi B. Mbulawa; Ontlametse T. Bareng; Pamela Smith-Lawrence; Roger Shapiro; Sefetogi Ramaologa; Shahin Lockman; Sikhulile Moyo; Simani Gaseitsiwe; Thela Tefelo; Thongbotho Mphoyakgosi; Wonderful T. Choga
EPI_ISL_3481365, EPI_ISL_3487617	British Columbia Centre For Disease Control	BCCDC Public Health Laboratory	Ana Pacagnella; Corrinne Ng; Dan Fornika; John Tyson; Kim Macdonald; Kimia Kamelian; Linda Hoang; Loretta Janz; Mel Krajden; Prystajecky Natalie; Robert Azana; Shannon Russell
EPI_ISL_3007338, EPI_ISL_8214111	Broad Institute Clinical Research Sequencing Platform	Infectious Disease Program, Broad Institute of Harvard and MIT	Adams, G.; B.L.; B.W.; Bauer, M.; Birren; Blumenstiel, B.; Brown, C.; Carter, A.; Chaluvadi, S.; D.J.; DeFelice, M.; DeRuff, K.; Dodge, S.; Gabriel, S.; Gallagher, G.; Gladden-Young, A.; Granger, B.; J.E.; K.J.; Lagerborg, K.; Larkin, K.; Lee, M.; Lemieux; Lennon, N.; Loreth, C.; Madoff, L.; McGovern, S.; Meldrim, J.; Normandin, E.; P.C.; Park; Pearlman, L.; Reilly, S.; Rudy, M.; Sabeti; Siddle; Smole, S.; Tomkins-Tinch, C.; Vicente, G.; and MacInnis
EPI_ISL_7747512	CAPRISA_HILLCREST	CERI, Centre for Epidemic Response and Innvoation, Stellenbosch University and KRISP, KZN Research Innovation and Sequencing Platform, UKZN.	Aida Sivro; Arisha Maharaj; Giandhari J; Naidoo Y; Natasha Samsunder; Pillay S; Ramphal U; Ramphal Y; San JE; Tegally H; Tshiabuila D; Wilkinson E; de Oliveira T
EPI_ISL_5016681, EPI_ISL_7130811, EPI_ISL_7335880, EPI_ISL_7703912	CDPH VBL	California Department of Public Health	Emily Smith on behalf of CDPH-COVIDNet
EPI_ISL_1628966	CERBALLIANCE	UMR PIMIT	Dr Camille Lebarbenchon; Dr David A Wilkinson; Dr Patrick Mavingui; Magali Turpin
EPI_ISL_4605097	CERBALLIANCE REUNION	CNR Virus des Infections Respiratoires - France SUD	Antonin Bal; Bruno Lina; Gregory Destras; Gwendolyne Burfin; Hadrien Regue; Laurence Josset; Martine Valette; Quentin Semanas
EPI_ISL_3347375	CT Department of Public Health	Grubaugh Lab - Yale School of Public Health	Anderson Brito; Annie Watkins; Chaney Kalinich; Chantal Vogels; Claire Pearson; Isabel Ott; Jessica Rothman; Joseph Fauver; Kendall Billig; Mallery Breban; Mary Petrone; Nathan Grubaugh; Tara Alpert; Tobias Koch; Tu N. Nguyen
EPI_ISL_2688697	Centre Hospitalier Eure Seine	Centre Hospitalier Universitaire de Rouen Laboratoire de Virologie	Alice Moisan; Fabienne De Oliveira; Marie Leoz
EPI_ISL_2467244, EPI_ISL_3242620, EPI_ISL_3242739	Clinica INDISA	"Facultad de Ciencias de la Vida, UNAB"	"Claudio Meneses; Ariel Orellana"; Claudio Olmos; Daniel Leon; Dayan Sanhueza; Eduardo Castro; Gonzalo Campaña; Macarena Bastias; Paola Pidal; Ricardo Yusta; Sebastian Wolter; Susana Saez; Víctor Monreal; Waldo Diaz
EPI_ISL_8206945	Clinical Microbiology Laboratory, Tel Aviv Sourasky Medical Center	Clinical Microbiology Laboratory, Tel Aviv Sourasky Medical Center	Alon Ziv; Amos Adler; Katya Levytskyi; Lior Handler; Ora Halutz
EPI_ISL_1664321	Clinical Molecular Microbiology Laboratory, UNC Hospitals	Jeremy Wang	Alexander Rubinsteyn; Colleen Rice; Corbin Jones; Jason Smedberg; Jeremy Wang; Melissa Miller; Robert Hagan; Shawn Hawken
EPI_ISL_3261639	Cliniques universitaires Saint-Luc	UCLouvain/IREC/MBLG	Benoit Kabamba Mukadi; Bertrand Bearzatto; Jean Ruelle
EPI_ISL_4634562	Colorado Department of Public Health and Environment	Colorado Department of Public Health and Environment	Alexandria Rossheim; Diana Ir; Emily A. Travanty; Laura Bankers; Mandy Waters; Michael Martin; Molly C. Hetherington-Rauth; Sarah Elizabeth Totten; Shannon R. Matzinger
EPI_ISL_1817733	Conville CDC wc CVC	NHLS/UCT	Arash Iranzadeh; Bruna Galvao; Carolyn Williamson; Deelan Doolabh; Diana Hardie; Innocent Mudau; Kruger Marais; Lynn Tyers; Marvin Hsiao; Stephen Korsman
EPI_ISL_4415852, EPI_ISL_4422568	DASA	DASA	Adriano Bonaldi; Angelica Hristov; Annelise Lopes; Bianca Cota; Cristina Oliveira; Jose Levi; Lidia Yamamoto; Paulo Pierry; Rodrigo Guarischi; Rodrigo Salazar
EPI_ISL_3053801, EPI_ISL_8136866, EPI_ISL_8171714	Department of Bacteria, Parasites and Fungi, Statens Serum Institut, Copenhagen, Denmark	Statens Serum Institut Bioinformatics and Microbial Genomics	Danish Covid-19 Genome Consortium
EPI_ISL_2811147, EPI_ISL_3872731	Department of Infectious Diseases, Kobe Institute of Health	Department of Infectious Diseases, Kobe Institute of Health	Kentaro Itokawa; Makoto Kuroda; Masanori Hashino; Noriko Nakanishi; Rina Tanaka; Ryohei Nomoto; Tomotada Iwamoto; Tsuyoshi Sekizuka
EPI_ISL_1310632	Department of Pathology, University of Cambridge	COVID-19 Genomics UK (COG-UK) Consortium	Aminu S. Jahun; Ian Goodfellow; Iliana Georgana; Martin D. Curran; Myra Hosmillo; Rhys Izuagbe; Surendra Parmar; William L. Hamilton; Yasmin Chaudhry
EPI_ISL_1876636	Department of Virus and Microbiological Special Diagnostics, Statens Serum Institut, Copenhagen, Denmark	Aalborg University	Danish Covid-19 Genome Consortium
EPI_ISL_1820249	Dept. of Medical Microbiology, Stavanger University Hospital, Helse Stavanger HF	Norwegian Institute of Public Health, Department of Virology	Atiya R Ali; Debech Nadia; Engebretsen Serina Beate; Garcia Llorente Ignacio; Hilde Elshaug; Hilde Vollan; Jon Bråte; Kamilla Heddeland Instefjord; Karoline Bragstad; Kathrine Stene-Johansen; Marie Paulsen Madsen; Olav Hungnes; Pedersen Benedikte Nevjen; Rasmus Riis Kopperud
EPI_ISL_1089695, EPI_ISL_1455691, EPI_ISL_1962271,	Dutch COVID-19 response team	National Institute for Public Health and the Environment (RIVM)	Adam Meijer; AnneMarie van den Brandt; Annelies Kroneman; Bas van der Veer; Chantal Reusken; Dennis Schmitz; Dirk Eggink; Euníce Then; Florian Zwagemaker; Harry Vennema; James Groot; Jeroen Cremer; Jolienke Hardeman; Karim Hajji; Kim Freriks; Linda van de Nes; Lisa Wijsman; Lynn Aarts; Melissa van Tuil; Robert Kohl; Ryanne Jaarsma; Sanne Bos; Sharon van den Brink; Sjoerd Kuiling; on behalf of the national COVID-19 response team

EPI_ISL_3057773			
EPI_ISL_2370486	ETHNIKO KENTRO AIMODOSIAS E.KE.A.	Greek Genome Center, Biomedical Research Foundation of the Academy of Athens (BRFAA)	Dimitrios Thanos; Efthimia Petinaki; Emmanouil Athanasiadis; Giannis Vatsellas; Katerina Zoi; Kostas Stamoulis; Theodoros Loupis
EPI_ISL_2477754	Edmonton Provincial Lab	Public Health Agency of Canada (PHAC) National Microbiology Laboratory	Buss; Croxen M; Deo A; Dieu P; E; Ferrato C; Gill K; Khan F; Koleva P; Li V; Lloyd C; Lynch T; Ma R; Murphy S; Pabbaraju K; Shokoples S; Thayer J; Tipples G; Whitehouse M; Wong A; Yu C; Zelyas N
EPI_ISL_1571313	Eurofins LifeCodexx GmbH	Robert Koch Institute	
EPI_ISL_4228722	Eurofins MVZ Labor Gelsenkirchen	Robert Koch Institute	
EPI_ISL_1663231	Fimlab Laboratories	Fimlab Laboratories	Bruno Luukinen; Leena Huhti; Mauri Keinänen; Minna Paloniemi; Sara Lehtinen; Tapio Seiskari
EPI_ISL_3100936, EPI_ISL_6049914	Florida Bureau of Public Health Laboratories	Florida Bureau of Public Health Laboratories	Jason Blanton; Namratha Tarigopula; Sarah Schmedes; Tiffany Splatt
EPI_ISL_1570873	Fraunhofer-Institut fÃ¼r Zelltherapie und Immunologie	Robert Koch Institute	
EPI_ISL_1667031, EPI_ISL_3305650, EPI_ISL_3609503, EPI_ISL_4369182, EPI_ISL_4369471, EPI_ISL_4371980, EPI_ISL_8081073			
see above	Fulgent Genetics	Centers for Disease Control and Prevention Division of Viral Diseases, Pathogen Discovery	Adrian Paskey; Becky Tsai; Benafsh Sapra; Benjamin Rambo-Martin; Christopher Gulvick; Clinton Paden; Clinton R. Paden; Dakota Howard; Darlene Wagner; Dhwani Batra; Doreen Ng; Duncan MacCannell; Erisa Sula; Harry Gao; James Xie; Jason Caravas; John Gao; Joseph Fierro; Kara Moser; Kristine Lacek; Matthew Schmerer; Mickey Li; Peter Cook; Peter W. Cook; Scott Sammons; Shatavia Morrison; Tymeckia Kendall; Victoria Caban Figueroa; Yan Meng; Yvette Unoarumhi
EPI_ISL_5797270	Fundacion Valle del Lili	Laboratorio de biotecnología, Universidad Icesi	"María I. Gutiérrez López; Adrián Camilo Rodríguez Ararat; Diana M. Florez Giraldo; Marcela Mercado; Maria F. Villegas Torres; Paola A. Caicedo Burbano"; Programa Nacional de Caracterización Genómica de SARS-CoV-2; Sara González Henao
EPI_ISL_4566162	Gencore - Universidad de los Andes	Gencore - Universidad de los Andes	Cristian Barrera; David González; Gabriela Ariza; Luisa Sacristan; Marcela Guevara; Marcela Mercado; Silvia Restrepo
EPI_ISL_406798	General Hospital of Central Theater Command of People's Liberation Army of China	BGI & Institute of Microbiology, Chinese Academy of Sciences & Shandong First Medical University & Shandong Academy of Medical Sciences & General Hospital of Central Theater Command of People's Liberation Army of China	Weifeng Shi and Zhenhong Hu; Weijun Chen; Yuhai Bi
EPI_ISL_1633527, EPI_ISL_2009197, EPI_ISL_2009624, EPI_ISL_2391368, EPI_ISL_2756096, EPI_ISL_2756124, EPI_ISL_2896151, EPI_ISL_3132696, EPI_ISL_3536127, EPI_ISL_4005118, EPI_ISL_4655970, EPI_ISL_5010817, EPI_ISL_5011403			
see above	Genetica Molecular and Subdepartamento de Virologia ISP Chile	Instituto de Salud Publica de Chile	Andres Castillo; Barbara Parra; Constanza Campano; Gisselle Barra; Jaime Lagos; Javier Tognarelli; Jorge Fernandez; Karen Orostica; Loredana Arata; Patricia Bustos; Rodrigo Fasce; Soledad Ulloa
EPI_ISL_2002482	Genomic Research Lab, BCSIR	Genomic Research Lab, BCSIR	Abu Sayeed Mohammad Mahmud; Barna Goswami; Eshrar Osman; Iffat Jahan; Md. Ahasan Habib; Md. Kamrul Islam; Md. Murshed Hasan Sarkar; Md. Saddam Hossain; Md. Salim Khan; Mohammad Mohi Uddin; Mohammad Samir Uzzaman; Shahina Akter; Tanjina Akhter Banu
EPI_ISL_5603127	Glencore Rhovan mine	National Institute for Communicable Diseases of the National Health Laboratory Service	Amoako DG; Bhiman JN; Everatt J; Ismail A; Mahlangu B; Mnguni A; Mohale T; Ntuli N; Scheepers C
EPI_ISL_5853660	H Divino Espirito Santo - Ponta Delgada	Instituto Nacional de Saude (INSA)	Borges et al
EPI_ISL_3102412	HOSPITAL E MATERNIDADE DRA ZILDA ARNS NEUMANN	Analytical Competence Molecular Epidemiology Lab/ACME, Oswaldo Cruz Foundation, Ceara (FIOCRUZ CE)	Cleber Furtado Aksenen; Fabio Miyajima; Fernando Braga Stehling; Francisco Eder de Moura Lopes; Jamille Maria Mendes Bezerra; Joaquim César do Nascimento Sousa Junior; Pedro Miguel Carneiro Jeronimo; Suzana Porto Almeida e Lucas Delerino; Thais Ferreira de Oliveira; Thais de Oliveira Costa; Ticiane Cavalcante de Souza; Veridiana Pessoa Miyajima
EPI_ISL_6481085	Helix	Centers for Disease Control and Prevention Division of Viral Diseases, Pathogen Discovery	Benjamin Rambo-Martin; Christopher Gulvick; Clinton Paden; Dakota Howard; Dhwani Batra; Duncan MacCannell; Erisa Sula; Helix CA; Jason Caravas; Kristine Lacek; Matthew Schmerer; Peter Cook; Scott Sammons; Shatavia Morrison; Tymeckia Kendall; Victoria Caban Figueroa; Yvette Unoarumhi
EPI_ISL_2320917, EPI_ISL_2599383, EPI_ISL_4346907, EPI_ISL_4347689	Helix/Illumina	Centers for Disease Control and Prevention Division of Viral Diseases, Pathogen Discovery	Adrian Paskey; Alexandre Bolze; Ary Ascencio; Benjamin Rambo-Martin; Brad Sickler; Charlotte Rivera-Garcia; Christine Tran; Christopher Gulvick; Chrstine Tran; Clinton Paden; Clinton R. Paden; Dakota Howard; Darlene Wagner; David Becker; Dhwani Batra; Duncan MacCannell; Efren Sandoval; Eileen De Feo; Eileen de Feo; Elizabeth Cirulli; Eric Allen; Geraint Levan; James Lu; Jan Antico; Jason Caravas; Jason Nguyen; Jimmy Ramirez; Jingtao Liu; Kara Moser; Kelly Barrett; Kelly Schiabor Barrett; Kim Gietzen; Kristine Lacek; Magnus Isaksson; Marc Laurent; Matthew Schmerer; Matthew Tolentino; Nicole L. Washington; Nicole Washington; Peter Cook; Peter W. Cook; Phil Febbo; Ryan Cho; Scott Sammons; Shannon Wickline; Shatavia Morrison; Sherry Wang; Simon White; Tyler Cassens; William Lee; Yvette Unoarumhi
EPI_ISL_3505986	Hospital General Universitario Gregorio Marañón	Hospital General Universitario Gregorio Marañón	Cristina Rodriguez-Grande; Darío García de Viedma; Julia Suárez; Laura Pérez-Lago; Marta Herranz Martin; Patricia Muñoz; Pedro Sola Campoy; Pilar Catalán; Sergio Buenestado Serrano; Victor Manuel de la Cueva
EPI_ISL_3216196	Hospital General Universitario de Alicante - Instituto de Investigación Sanitaria y Biomédica de Alicante	SeqCOVID-SPAIN consortium/IBV(CSIC)	Carmen Molina Pardines and SeqCOVID-SPAIN consortium; Maripaz Ventero Martín
EPI_ISL_2600860	Hospital Universitari i Politècnic La Fe de València	SeqCOVID-SPAIN consortium/IBV(CSIC)	Ana Gil Brusola; Eva González Barberá; José Luis López Hontangas and SeqCOVID-SPAIN consortium; María Dolores Gómez Ruiz; Salvador Giner Almaraz
EPI_ISL_7307141	Hospital Universitario San Ignacio	Centro de Investigaciones en Microbiología y Biotecnología-UR (CIMBIUR), Facultad de Ciencias Naturales, Universidad del Rosario, Bogotá, Colombia	Alberto Paniz-Mondolfi; Angie Ramírez; Beatriz Ariza; Camilo A. Correa-Cárdenas; Carlos Gómez-Restrepo; Claudia Cardozo-Romero; Claudia Méndez; David-Santiago Quevedo; Guido España; Hernando Díaz; Juan David Ramírez; Juliana Cuervo-Rojas; Julie Pérez; Luz H. Patiño; Manuel-Antonio Franco; Maria-Clara Duque; Marina Muñoz; Nathalia Ballesteros; Nicolas Luna; Sergio Castañeda; Zulma M. Cucunubá
EPI_ISL_1755806	Hôpital Avicenne	Department of Virology, Henri Mondor University Hospital, Assistance Publique Hôpitaux de Paris, Université Paris-Est Créteil, INSERM U955	Alexandre Soulier; Christophe Rodriguez; Elisabeth Trawinski; Guillaume Gricourt; Jean-Michel Pawlotsky; Melissa N'Debi; Slim Fourati; Vanessa Demontant
EPI_ISL_2637420	Hôpital Pitié-Salpêtrière	Department of Virology, Henri Mondor University Hospital, Assistance Publique Hôpitaux de Paris, Université Paris-Est Créteil, INSERM U955	Alexandre Soulier; Christophe Rodriguez; Elisabeth Trawinski; Guillaume Gricourt; Jean-Michel Pawlotsky; Melissa N'Debi; Slim Fourati; Vanessa Demontant
EPI_ISL_7314430	IDIME	Instituto Nacional de Salud- Dirección de Investigación en Salud Pública	Beatriz de Arco; Carlos Franco-Muñoz; Diego A. Álvarez-Díaz; Diego Andrés Prada; Dioselina Peláez-Carvajal; Gerardo Santamaría; Héctor Alejandro Ruiz-Moreno; Jhonnatan Reales-González; Jorge Rivera; Julián Naizaque; Katherine Laiton-Donato; Marcela Mercado-Reyes.; Martha Lucia Ospina Martínez; María T. Herrera-Sepúlveda; Paola Rojas-Estevez; Sheryll Corchuelo; Tatiana Cobos
EPI_ISL_7221763	INSACOG-IGIB and Kerala State SARS-COV-2 Genome Surveillance Programme (GENESCOV2)	INSACOG and GENESCOV2 at CSIR Institute of Genomics and Integrative Biology	INSACOG-IGIB and Kerala State SARS-COV-2 Genome Surveillance Programme (GENESCOV2)
EPI_ISL_5647578, EPI_ISL_5653077	INSIDE DIAGNOSTICOS	Instituto Butantan	Antonio Jorge Martins; Claudia Renata dos Santos Barros; David Schlesinger; Debora Botequio Moretti; Dimas Tadeu Covas; Elaine Cristina Marqueze; Elaine Vieira Santos; Evandra Strazza Rodrigues; Heidge Fukumasu; Jayme Augusto de Souza-Neto; José Salvatore Leister Patané; Luiz Alcantara; Luiz Lehmann Coutinho; Maria Carolina Elias; Maurício Lacerda Nogueira; Rafael dos Santos Bezerra; Raul Machado Neto; Rejane Maria Tommasini Grotto; Ricardo Haddad; Sandra Coccuzzo Sampaio Vessoni; Simone Kashima; Svetoslav Nanev Slavov; Vincent Louis Viala
EPI_ISL_6128872	INSPI-CRN DE INFLUENZA Y OTROS VIRUS RESPIRATORIOS	INSPI-CRN DE INFLUENZA Y OTROS VIRUS RESPIRATORIOS	Alfredo Bruno; Daniel Ramos; Domenica de Mora; Fernando Llerena; Jimmy Garcés; Lizbeth Patiño; Marcela Mejia; Maria Angelica Becerra; Maritza Olmedo; Michelle Páez; Ruben Armas
EPI_ISL_4497083	INSPI-CRN DE INFLUENZA Y OTROS VIRUS RESPIRATORIOS	NIC-INSPI	Alfredo Bruno; Carlos Chiluisa; Domenica de Mora.; Jimmy Garcés; Johanna Laines; Lizbeth Patiño; Manuel Gonzalez; Maritza Olmedo; Michelle Páez
EPI_ISL_8255195	Illinois Department of Public Health - Springfield Lab	Illinois Department of Public Health - Springfield Lab	Bryan Sim; Gordon McCall
EPI_ISL_2186784, EPI_ISL_4139714, EPI_ISL_4384506, EPI_ISL_8032846	Infinity Biologix	Centers for Disease Control and Prevention Division of Viral Diseases, Pathogen Discovery	Adrian Paskey; Benjamin Rambo-Martin; Chirayu Goswami; Christian Bixby; Christopher Gulvick; Clinton Paden; Clinton R. Paden; Dakota Howard; Darlene Wagner; Dhwani Batra; Duncan MacCannell; Erisa Sula; Jason Caravas; Jonathan Schultz; Kara Moser; Kristine Lacek; Matthew Schmerer; Peter Cook; Peter W. Cook; Robin Grimwood; Russ Hager; Scott Sammons; Shatavia Morrison; Tymeckia Kendall; Victoria Caban Figueroa; Yihe Wang; Yvette Unoarumhi
EPI_ISL_2828667	Instituo Adolfo Lutz - Reginal de Ribeirao Preto	Instituto Adolfo Lutz, Interdiciplinary Procedures Center, Strategic Laboratory	Caio Vinicius Dias Lopes; Claudia Regina Gonçalves; Claudio Tavares Sacchi; Erica Valessa Ramos Gomes; Karoline Rodrigues Campos
EPI_ISL_6779106	Institute for Infectious Diseases	Institute for Infectious Diseases	Alban Ramette; Christian Baumann; Cora Sägesser; Franziska Suter-Riniker; Loïc Borcard; Miguel A Terrazos Miani; Nicole Liechti; Pascal Bittel; Peter Keller; Sonja Gempeler; Stefan Neuenschwander; Stephen L Leib
EPI_ISL_7898712	Instituto Adolfo Lutz - Regional de Santo Andre	Instituto Adolfo Lutz, Interdiciplinary Procedures Center, Strategic Laboratory	Ariadne Ferreira Amarante; Caio Vinicius Dias Lopes; Claudia Regina Gonçalves; Claudio Tavares Sacchi; Karoline Rodrigues Campos; Leonardo Tadeu de Araujo; Marlon Benedito Nascimento Santos
EPI_ISL_2609537	Instituto Nacional de Investigação em Saúde	KRISP, KZN Research Innovation and Sequencing Platform	Afonso P; David K; Emmanuel SJ; Freitas RH; Giandhari J; Inglês L; Lutucuta S; Miranda J; Morais J; Mufinda M; Naidoo Y; Neto Z; Paulo A Carralero RR Paixão JP; Pereira A; Pillay S; Tegally H; Wilkinson E; de Oliveira T
EPI_ISL_2970402	Instituto Nacional de Medicina Genomica	Instituto Nacional de Medicina	Blancas S; Cedro-Tanda A; Cisneros- Villanueva M; Cisneros-Villanueva M; Escobar-Arrazola; Gonzalez-Barrera D; Herrera-Montalvo LA.; Hidalgo-Miranda A; M; Mendoza-Vargas A; Munguia-Garza P; Orjuela-Rodríguez M; Ramirez-Vega O; Rangel-DeLeon D; Reyes-Grajeda JP

		Genomica	
EPI_ISL_2295406	Instituto de Biotecnologia - UNESP-Botucatu-SP	Instituto de Biotecnologia - UNESP-Botucatu-SP	Cecília Artico Banho; Cíntia Bittar; Fábio Sossai Possebon; Guilherme Campos; Helena Lage Ferreira; Jorge A. Petroli Marchesi; João Pessoa Araújo Jr.; Leila Sabrina Ullmann; Lívia Sacchetto; Maisa C. Pereira Parra; Marília Moraes; Maurício L. Nogueira; Paula Rahal; Paulo Inacio da Costa
EPI_ISL_2636203	Israel Central Virology laboratory	Israel National Consortium for SARS-CoV-2 sequencing	Dana Bar-Ilan; Efrat Dahan Bucris; Efrat Glick-Saar; Ella Mendelson; Gideon Rechavi; Michal Mandelboim; Miranda Geva; Neta Zuckerman; Omri Nayshool; Oran Erster; Orna Mor
EPI_ISL_2331479	Johns Hopkins Hospital Department of Pathology	Johns Hopkins Hospital Department of Pathology	Adannaya Amadi; C. Paul Morris; Chun Huai Luo; Heba H. Mostafa; Matthew Schwartz; Nicholas Gallagher
EPI_ISL_1382279, EPI_ISL_2833494, EPI_ISL_2877058	KU Leuven, Rega Institute, Clinical and Epidemiological Virology	KU Leuven, Rega Institute, Clinical and Epidemiological Virology	Bert Vanmechelen; Joan Marti-Carerras; Piet Maes; Tony Wawina-Bokalanga
EPI_ISL_3825658	Kaiser Permanente NW Reginal Lab	OHSU MM Lab	Yun Wu
EPI_ISL_2802073	Klinika za infektivne bolesti "Dr. Fran Mihaljević"	Hrvatski zavod za javno zdravstvo	Irena Tabain; Ivana Ferenčak
EPI_ISL_5071120	LABORATORIO ALIFE HEALTH	Instituto Nacional de Salud- Dirección de Investigación en Salud Pública	Carlos Franco-Muñoz; Carmen Osorio; Diana Malo; Diego A. Álvarez-Díaz; Diego Andrés Prada; Gerardo Santamaría; Hector Alejandro Ruiz-Moreno; Jhonnatan Reales-González; Jorge Rivera; Juan Camilo Martinez; Julian Naizaque; Katherine Laiton-Donato; Lisseth Pardo; Magdalena Wiesner; Marcela Mercado-Reyes; Maria T. Herrera-Sepúlveda; Marta Lopez Blanco; Martha Lucia Ospina Martinez; Paola Rojas-Estevez; Sergio Gomez; Sheryll Corchuelo; Ángela Alarcon Cruz
EPI_ISL_3385839	LABORATORIO CLINICO CRISTIAN GRAM IPS SAS	Instituto Nacional de Salud- Dirección de Investigación en Salud Pública	Carlos Franco-Muñoz; Carmen Osorio; Diana Malo; Diego A. Álvarez-Díaz; Diego Andrés Prada; Gerardo Santamaría; Hector Alejandro Ruiz-Moreno; Jhonnatan Reales-González; Jorge Rivera; Juan Camilo Martinez; Julian Naizaque; Katherine Laiton-Donato; Lisseth Pardo; Magdalena Wiesner; Marcela Mercado-Reyes; Maria T. Herrera-Sepúlveda; Marta Lopez Blanco; Martha Lucia Ospina Martinez; Paola Rojas; Sergio Gomez; Sheryll Corchuelo; Ángela Alarcon Cruz
EPI_ISL_2345266	LABORATORIO DE FRANCA	Instituto Butantan / ESALQ-Piracicaba	Antonio Jorge Martins; Claudia Renata dos Santos Barros; David Schlesinger; Debora Botequio Moretti; Dimas Tadeu Covas; Elaine Cristina Marqueze; Elaine Vieira Santos; Evandra Strazza Rodrigues; Heidge Fukumasu; Jayme Augusto de Souza-Neto; José Salvatore Leister Patané; Luiz Alcantara; Luiz Lehmann Coutinho; Maria Carolina Elias; Maurício Lacerda Nogueira; Rafael dos Santos Bezerra; Raul Machado Neto; Rejane Maria Tommasini Grotto; Ricardo Haddad; Sandra Coccuzzo Sampaio Vessoni; Simone Kashima; Svetoslav Nanev Slavov; Vincent Louis Viala
EPI_ISL_5055230	LABORATORIO IMAT	Instituto Nacional de Salud- Dirección de Investigación en Salud Pública	Beatriz de Arco; Carlos Franco-Muñoz; Carmen Osorio; Diana Malo; Diego A. Álvarez-Díaz; Diego Andrés Prada; Gerardo Santamaría; Hector Alejandro Ruiz-Moreno; Jhonnatan Reales-González; Jorge Rivera; Juan Camilo Martinez; Julian Naizaque; Katherine Laiton-Donato; Lisseth Pardo; Magdalena Wiesner; Marcela Mercado-Reyes; Maria T. Herrera-Sepúlveda; Marta Lopez Blanco; Martha Lucia Ospina Martinez; Paola Rojas; Sergio Gomez; Sheryll Corchuelo; Tatiana Cobos; Ángela Alarcon Cruz
EPI_ISL_1643509	Labor Dr. Wisplinghoff - KÃ¶ln	Robert Koch Institute	
EPI_ISL_2390702	Labor ZOTZ|KLIMAS; MVZ Düsseldorf-Centrum	Robert Koch Institute	
EPI_ISL_2111154	Laborarztpraxis Dres. med. Walther Weindel & Kollegen	Robert Koch Institute	
EPI_ISL_2374084	Laboratoire Virologie Saint Louis APHP	Laboratoire Virologie Saint Louis APHP	Constance Delauguerre; Jérome Le Goff; Linda Feghoul; Marie Laure Chaix; Marie Laure Néré; Maud Salmona; Severine Mercier Delarue; Sophia Achaibou
EPI_ISL_2975479, EPI_ISL_7707101	Laboratoire de santé publique du Québec	Laboratoire de santé publique du Québec	Guillaume Bourque; Ioannis Ragoussis; Jesse Shapiro; Mark Lathrop and Judith Fafard on behalf of the CoVSeQ research group; Mark Lathrop and Michel Roger on behalf of the CoVSeQ research group; Sandrine Moreira
EPI_ISL_2401014	Laboratoire national de sante, Microbiology, Virology	Laboratoire national de sante, Microbiology, Microbial Genomics Platform	Anke Wienecke-Baldacchino; Catherine Ragimbeau; Fatu Djabi; Jessica Tapp; Lise Pignon; Raoul Salmon; Tamir Abdelrahman; Trung Nguyen Nguyen
EPI_ISL_3148352	Laboratoires d'analyses medicales - Ketterthill	Laboratoire national de sante, Microbiology, Microbial Genomics Platform	Anke Wienecke-Baldacchino; Caroline Scheiber; Catherine Ragimbeau; Elodie Solarino; Fatu Djabi; Jessica Tapp; Lise Pignon; Raoul Salmon; Serge Vedy; Tamir Abdelrahman; Virginie Jover
EPI_ISL_4946240	Laboratorio Angel (Synlab)	Instituto Nacional de Salud- Dirección de Investigación en Salud Pública	Carlos Franco-Muñoz; Carmen Osorio; Diana Malo; Diego A. Álvarez-Díaz; Diego Andrés Prada; Gerardo Santamaría; Hector Alejandro Ruiz-Moreno; Jhonnatan Reales-González; Jorge Rivera; Juan Camilo Martinez; Julian Naizaque; Katherine Laiton-Donato; Lisseth Pardo; Magdalena Wiesner; Marcela Mercado-Reyes; Maria T. Herrera-Sepúlveda; Marta Lopez Blanco; Martha Lucia Ospina Martinez; Paola Rojas; Sergio Gomez; Sheryll Corchuelo; Ángela Alarcon Cruz
EPI_ISL_2835946	Laboratorio Central de Epidemiología (LCE)	Centro de Investigación en Enfermedades Infecciosas (CIENI), Instituto Nacional de Enfermedades Respiratorias (INER)	; Alejandra Garcia-Gasca; Alejandra Hernández-Terán; Alejandro Sanchez-Flores; Alfredo Herrera-Estrella; Alicia Ocaña-Mondragón; Andreu Comas-García; Angel Gustavo Salas-Lais; Antonio Loza Román; Bernardo Martínez-Miguel; Blanca Taboada; Brenda Irasema Maldonado-Meza; Bruno Gomez-Gil; Carla Ivón Herrera-Najera; Carlos F. Arias; Celia Boukadida; Clara Esperanza Santacruz-Tinoco; Concepción Grajales-Muñiz; Consorcio Mexicano de Vigilancia Genómica (CoViGen-Mex). Authors (in alphabetical order): Julio Elias Alvarado-Yaah; Cristóbal Cháidez-Quiróz; Célida Duque Molina; Célida Martínez- Rodríguez; Daniel Fregoso-Rueda; Daniel Lira Morales; Eduardo Becerril-Vargas; Fernando Fontove-Herrera; Fidencio Mejía-Nepomuceno; Francisco Pulido; Gloria Elena Espinosa-Ayala; Gloria María Molina-Salinas; Gloria Vazquez; Hector Esteban Paz-Juárez; Hector Montoya-Fuentes; Helen Haydee Fernanda Ramírez-Plascencia; Irvin González-López; Jean Pierre González; Joel Armando Vázquez-Pérez.; Jorge Salas-Hernández; José Antonio Enciso-Moreno; José Arturo Martínez-Orozco; José Esteban Muñoz-Medina; José de Jesús Nuñez-Contreras; Juan Bautista Chale-Dzul; Julissa Enciso-Ibarra; Luis Alberto Ochoa-Carrera; Margarita Matías-Florentino; Mario Mújica-Sánchez; Marissa Perez-García; María Guadalupe Santiago-Mauricio; María Guadalupe de Jesús Mireles-Rivera; Nelly Sélem-Mojica; Pavel Isa; Ricardo Ciria Merce; Ricardo Grande; Rosa María Gutierrez Rios; Santiago Ávila-Ríos; Selene Zárate; Susana Lopez; Victor Eduardo Garcia-Arias; Víctor Hugo Borja-Aburto
EPI_ISL_3434899	Laboratorio Central de Saude Publica do Estado do Amapa (LACEN/AP)	Laboratory of Respiratory Viruses and Measles, Oswaldo Cruz Institute, FIOCRUZ	Agatha Cristinne Prudencio; Alice Sampaio Rocha; Ana Carolina Mendonca; Andreia Santos Costa; Anna Carolina Paixao; Anne Caroline da Silva Soledade; Elisa Cavalcante Pereira; Fernando Motta; Ighor Leonardo Arantes Gomes; Lindomar dos Anjos Silva; Luciana Appolinario; Marcia Socorro Pereira Cavalcante; Marilda Siqueira on behalf of the Fiocruz COVID-19 Genomic Surveillance Network; Paola Resende; Renata Serrano Lopes; Taina Venas
EPI_ISL_2196197	Laboratorio Central de Saude Publica do Estado do Rio de Janeiro (LACEN-RJ)	Laboratory of Respiratory Viruses and Measles, Oswaldo Cruz Institute, FIOCRUZ	Alice Sampaio Rocha; Ana Carolina Mendonca; Andrea Cony Cavalcanti; Anna Carolina Paixao; Elisa Cavalcante Pereira; Fernando Motta; Luciana Appolinario; Marilda Siqueira on behalf of the Fiocruz COVID-19 Genomic Surveillance Network; Paola Resende; Renata Serrano Lopes; Taina Venas
EPI_ISL_5604607	Laboratorio Masvida	Instituto Nacional de Salud- Dirección de Investigación en Salud Pública	Beatriz de Arco; Carlos Franco-Muñoz; Diego A. Álvarez-Díaz; Diego Andrés Prada; Dioselina Peláez-Carvajal; Gerardo Santamaría; Hector Alejandro Ruiz-Moreno; Jhonnatan Reales-González; Jorge Rivera; Julián Naizaque; Katherine Laiton-Donato; Marcela Mercado-Reyes.; Martha Lucia Ospina Martínez; María T. Herrera-Sepúlveda; Paola Rojas-Estevez; Sheryll Corchuelo; Tatiana Cobos
EPI_ISL_6703122	Laboratorio SYNLAB Colombia	CIAT, Laboratorio de Virologia	Ana M. Leiva; Diana Lopez-Alvarez; Programa Nacional de Caracterización Genómica de SARS-CoV-2; WilmerJ. Cuellar
EPI_ISL_7961350	Laboratorio de Biologia Molecular - Hospital Carlos Alberto Seguín Escobedo (ESSALUD)	Laboratorio de Genómica Microbiana, Universidad Peruana Cayetano Heredia	Brenes Hebleen; Camacho Erwin; Campos-Sánchez Rebeca; Ceballos Ana; Cordero Estela; Cristancho Marco; César Iván Chávez López; Diego Cuicapuza; Duarte Francisco; Erla Patricia Asencios Vales; Fernández-Do Porto Darío; Guillermo Salvatierra; Herrera-Estrella Alfredo; Iohana Nazarita Vera Núñez; Isabel Llerena Gamero; Janet Huancachoque / Fernando Sánchez Fragoso; Jiménez-Moraila Beatriz; Kreuze Jan; Lin Geraldin Zevallos Cuarite; Mireles-Rivera Guadalupe; Molina-Mora José; Muñoz-Medina José Esteban; Negri Tatiana; Nunes Gisele; Oliveira Guilherme; Oliveira Renato; Pedro E. Romero; Reales-González Jhonnatan; Remes-Lenicov Federico; Reyes Alejandro; Rosario Elsa Jesús; Segundo Fuentes; Sosa Ezequiel; Soto Claudio; Tatiana Shessira Chávez Arias; Tsukayama Pablo; Turjanski Adrián; Zúñiga Manrique; and Zayat Jonathan / Alejandra Dávila-Barclay
EPI_ISL_3376696	Laboratorio de Biología Molecular - Universidad del Magdalena	Centro de Genética y Biología Molecular - Universidad del Magdalena	Andrea M. Ramírez Hernandez; Angel Oviedo Marquez; Daniel Bautista; Lyda R. Castro; Maria Teresa Mojica-Ortiz
EPI_ISL_3707390	Laboratorio de Genómica Microbiana, Universidad Peruana Cayetano Heredia	Laboratorio de Genómica Microbiana, Universidad Peruana Cayetano Heredia	Alejandra Dávila-Barclay; Diego Cuicapuza; Guillermo Salvatierra; Janet Huancachoque; Luis González; Pablo Tsukayama; Pedro E. Romero; Pool Marcos
EPI_ISL_3133703, EPI_ISL_3758942	Laboratorio de Infectologia y Virologia Molecular	Laboratory of Molecular Virology, School of Medicine, Pontificia Universidad Catolica de Chile	Ana Maria Contreras; Andres E. Munoz-Marcos; Carlos Palma; Catalina Pardo-Roa; Constanza Maldonado; Constanza Martinez-Valdevenito; Eileen Serrano; Erick Salinas; Estefany Poblete; Francisco Melo; Jennifer Angulo; Jorge Levican; Leonardo I. Almonacid; M. Belen Leyton; Marcela Ferres; Maria Jose Avendano; Rafael A. Medina; Tamara Garcia-Salum
EPI_ISL_3537555, EPI_ISL_3537600, EPI_ISL_3547031, EPI_ISL_3670583, EPI_ISL_3769581, EPI_ISL_3987841, EPI_ISL_4036755, EPI_ISL_4080203, EPI_ISL_4080280, EPI_ISL_4417204, EPI_ISL_4417382, EPI_ISL_4417405, EPI_ISL_4635258, EPI_ISL_4740164, EPI_ISL_5115887, EPI_ISL_5504530, EPI_ISL_6574110			
see above	Laboratorio de Referencia Nacional de Virus Respiratorios. Centro Nacional de Salud Publica. Instituto Nacional de Salud Peru.	Laboratorio de Referencia Nacional de Virus Respiratorios. Centro Nacional de Salud Publica. Instituto Nacional de Salud Peru.	Alicia Nuñez Llanos; Carlos Padilla Rojas; Edwart Rogger Rivera Serrano; Henri Bailon Calderon; Iris Silva Molina; Joseph Huayra Niquen; Kelly Vanessa Izarra Rojas; Lely Solari Zerpa; Luis Barcena Flores; Marco Galarza Perez; Nancy Rojas Serrano; Nieves Sevilla Castañeda; Omar Caceres Rey; Orson Mestanza Millones; Princesa Medrano Alhuay; Priscila Lope Pari; Sandra Morales Ruiz; Sara Gordillo Vilchez; Steve Acedo Lazo; Veronica Hurtado Vela; Victor Jimenez Vasquez; Wendy Lizarraga Olivares
EPI_ISL_2536793, EPI_ISL_2536881, EPI_ISL_2921449, EPI_ISL_2921499, EPI_ISL_3023521, EPI_ISL_3375975, EPI_ISL_3376040			
see above	Laboratorio de Referencial Nacional de Virus Respiratorios	Laboratorio de Referencial Nacional de Virus Respiratorios	Carlos Padilla Rojas; Henri Bailon Calderon; Iris Silva Molina; Joseph Huayra Niquen; Lely Solari Zerpa; Luis Barcena Flores; Marco Galarza Perez; Nancy Rojas Serrano; Omar Caceres Rey; Orson Mestanza Millones; Priscila Lope Pari; Sandra Morales Ruiz; Steve Acedo Lazo; Veronica Hurtado Vela
EPI_ISL_1548411, EPI_ISL_2184764, EPI_ISL_3687413, EPI_ISL_4375598, EPI_ISL_4376018, EPI_ISL_4381627, EPI_ISL_6079425, EPI_ISL_7904286, EPI_ISL_8156279			
see above	Laboratory Corporation of America	Centers for Disease Control and Prevention Division of Viral Diseases, Pathogen Discovery	Adrian Paskey; Amanda Douglas; Amanda Suchanek; Andrea Throop; Ayla Burns; Benjamin Rambo-Martin; Bobbi Croy; Brian Krueger; Brian Norvell; Christopher Gulvick; Christos Petropoulos; Clinton Paden; Clinton R. Paden; Craig Lukasik; Dakota Howard; Darlene Wagner; Debbie Boles; Dhwani Batra; Duncan MacCannell; Eyad Almasri; Goran Stevovic; Howard Engler; Hrushikesh Deshmukh; Jake Humphrey; Jana Schroth; Jason Caravas; Joe Voshell; John Pruitt; Jonathan Meltzer; Jonathan Williams; Kara Moser; Kimberly Wagner; Kristine Lacek; Lax Iyer; Lisa Pfefferle; Lyndon Tilson; Manoj Jain; Marcia Eisenberg; Mary Ann Cristobal; Mary Cristobal; Mary Williamson; Matthew Robinson; Matthew Schmerer; Michael Levandoski; Mike Sapeta; Mindy Nye; Minoo Agarwal; Mohan Kolli; Nuthawin Charoensri; Oren Cohen; Peter Cook; Peter W. Cook; Prashant Gupta; Qian Zeng; Rama Ghatti; Scott Parker; Scott Ryan; Scott Sammons; Shatavia Morrison; Stanley Letovsky; Steven Ragan; Suresh Babu Selvaraju; Suresh Selvaraju; Susan Countryman; Susan Hicks; Suzanne Dale; Thomas Urban; Tim Kuphal; Tricia Zwiefelhofer; Tymeckia Kendall; Victoria Caban Figueroa; Vincent Drouillon; Yvette Unoarumhi
EPI_ISL_3050545, EPI_ISL_4516039	Laboratório Central de Saúde Pública do Amazonas - LACEN-AM	Laboratorio de Ecologia de Doencas Transmissiveis na Amazonia, Instituto Leonidas e Maria Deane - Fiocruz Amazonia	André Corado; Felipe Naveca; Fernanda Nascimento; George Silva; Karina Pessoa; Luciana Gonçalves; Maria Júlia Brandão; Matilde Mejía; Valdinete Nascimento; Victor Souza; Ágatha Costa
EPI_ISL_4543295	Laboratório de Biotecnologia Aplicada (LBA) - Laboratório de Biologia Molecular - Hospital das Clínicas, Faculdade de Medicina de Botucatu, Departamento de Bioprocessos e Biotecnologia - Faculdade de Ciências Agronômicas, UNESP – Botucatu/SP	Laboratory of Respiratory Viruses and Measles, Oswaldo Cruz Institute, FIOCRUZ	Alice Sampaio Rocha; Ana Carolina Mendonca; Anna Carolina Paixao; Elisa Cavalcante Pereira; Felipe Allan da Silva Costa; Fernando Motta; Jayme Augusto de Souza Neto; Leonardo Nazario de Moraes; Luciana Appolinario; Marilda Siqueira on behalf of the Fiocruz COVID-19 Genomic Surveillance Network; Paola Resende; Patricia Akemi Assato; Rejane Maria Tommasini; Renata Serrano Lopes; Taina Venas
EPI_ISL_5400838	Landesamt für Verbraucherschutz des Landes Sachsen-Anhalt	Robert Koch Institute	
EPI_ISL_796926, EPI_ISL_1330351, EPI_ISL_1414295, EPI_ISL_1700095, EPI_ISL_3368302, EPI_ISL_4010236, EPI_ISL_5134344, EPI_ISL_5974027, EPI_ISL_7893732, EPI_ISL_7992921, EPI_ISL_8048904, EPI_ISL_8237543			
see above	Lighthouse Lab in Alderley Park	Wellcome Sanger Institute for the COVID-19 Genomics UK (COG-UK) Consortium	Cordelia Langford; David K. Jackson; Dominic Kwiatkowski; Ewan Harrison; Ian Johnston; Jacquelyn Wynn; Jeffrey Barrett; John Sillitoe on behalf of the Wellcome Sanger Institute COVID-19 Surveillance Team; Mairead Hyland; Roberto Amato; Sonia Goncalves; The Lighthouse Lab in Alderley Park and Alex Alderton
EPI_ISL_7305684,	Lighthouse Lab in Glasgow	Wellcome Sanger Institute for the	Anna Dominiczak and Alex Alderton; Carol Clugston; Cordelia Langford; David Gray; David K. Jackson; Dominic Kwiatkowski; Ewan Harrison; Harper VanSteenhouse; Ian Johnston; Jeffrey Barrett; John Sillitoe on behalf of the Wellcome Sanger Institute COVID-19 Surveillance Team; Roberto Amato; Sonia

EPI_ISL_7630312, EPI_ISL_7867167		COVID-19 Genomics UK (COG-UK) Consortium	Goncalves; Yumi Kasai
EPI_ISL_873910, EPI_ISL_875020, EPI_ISL_2540099, EPI_ISL_3884142, EPI_ISL_5786901, EPI_ISL_6225882, EPI_ISL_7557476, EPI_ISL_7579656, EPI_ISL_7582935, EPI_ISL_7655401, EPI_ISL_7718518, EPI_ISL_7838025, EPI_ISL_7867480, EPI_ISL_7964979, EPI_ISL_7968329, EPI_ISL_7974757, EPI_ISL_8040219, EPI_ISL_8041334, EPI_ISL_8041571, EPI_ISL_8101978, EPI_ISL_8102137, EPI_ISL_8167447, EPI_ISL_8168656, EPI_ISL_8241909			
see above	Lighthouse Lab in Milton Keynes	Wellcome Sanger Institute for the COVID-19 Genomics UK (COG-UK) Consortium	Cordelia Langford; David K. Jackson; Dominic Kwiatkowski; Ewan Harrison; Ian Johnston; Jeffrey Barrett; John Sillitoe on behalf of the Wellcome Sanger Institute COVID-19 Surveillance Team; Roberto Amato; Sonia Goncalves; The Lighthouse Lab in Milton Keynes and Alex Alderton
EPI_ISL_7862511, EPI_ISL_7955308, EPI_ISL_8235063	Lighthouse Laboratory Plymouth	Wellcome Sanger Institute for the COVID-19 Genomics UK (COG-UK) Consortium	Cordelia Langford; David K. Jackson; Dominic Kwiatkowski; Ewan Harrison; Ian Johnston; Jeffrey Barrett; John Sillitoe on behalf of the Wellcome Sanger Institute COVID-19 Surveillance Team; Lighthouse Laboratory Plymouth and Alex Alderton; Roberto Amato; Sonia Goncalves
EPI_ISL_3152346	MD PHL	Maryland Department of Health Laboratories Administration	Ami Patel; Eric N. Keller; Jillian Loomis; Kwang Low; Terence L. Moore; and Robert Myers
EPI_ISL_5659977	MDI Limbach Berlin GmbH; MVZ Labor Berlin	Robert Koch Institute	
EPI_ISL_2689877	MS Public Health Laboratory	Centers for Disease Control and Prevention Division of Viral Diseases, Pathogen Discovery	Alison Laufer Halpin; Ben L. Rambo-Martin; Clinton R. Paden; Dakota Howard; Darlene Wagner; Dave Wentworth; Dhwani Batra; Jasmine Padilla; Justin Lee; Katie Dillon; Krista Queen; Kristen Knipe; Kristine Lacek; Mark Burroughs; Matthew Schmerer; Mili Sheth; Peter Cook; Sam Shepard; Sarah Nobles; Shoshona Le; Suxiang Tong; Vivien Dugan; Yvette Unoarumhi
EPI_ISL_3427956, EPI_ISL_8126798, EPI_ISL_8262424	Mako Medical	Centers for Disease Control and Prevention Division of Viral Diseases, Pathogen Discovery	Adrian Paskey; Benjamin Rambo-Martin; Christopher Gulvick; Clinton Paden; Clinton R. Paden; Dakota Howard; Darlene Wagner; Dhwani Batra; Duncan MacCannell; Erisa Sula; Jason Caravas; Kara Moser; Kristine Lacek; Lauren Moon; Matthew Schmerer; Matthew Tugwell; Peter Cook; Peter W. Cook; Scott Sammons; Shatavia Morrison; Tymeckia Kendall; Victoria Caban Figueroa; Yvette Unoarumhi
EPI_ISL_6963085	Malawi Liverpool Wellcome Trust Clinical Research Program	Malawi Liverpool Wellcome Trust Clinical Research Program	Ben Morton; Catherine Anscombe; Kondwani Jambo; Philip Ashton; Sam Lissauer
EPI_ISL_1367749, EPI_ISL_7495079	Michigan Department of Health and Human Services, Bureau of Laboratories	Michigan Department of Health and Human Services, Bureau of Laboratories	Blankenship HM; Riner D; Soehnlen MK
EPI_ISL_2140547	Microbiology Department, Laboratori Clínic Metropolitana Nord. Hospital Universitari Germans Trias i Pujol	Can Ruti SARS-CoV-2 Sequencing Hub (HUGTiP/IrsiCaixa/IGTP)	Alba Sánchez; Anna Not; Antoni E Bordoy; Bonaventura Clotet; Cristina Casañ; Cristina Esteban; Francesc Catala-Moll; Gemma Clara; Ignacio Blanco; Marc Noguera-Julian; Maria Casadellà; Mariona Parera; Mercedes Guerrero; Montserrat Giménez; Pere-Joan Cardona; Pilar Armengol; Roger Paredes; Verónica Saludes; and Elisa Martró on behalf of the Can Ruti SARS-CoV-2 Sequencing Hub.
EPI_ISL_5331932	Microbiology Department, University Hospital Donostia	Microbiology Department, University Hospital Donostia	Cilla G.; Gomez M; Marimon JM; Martin-Peñaranda T; Montes M; Piñeiro L; Sorarrain A
EPI_ISL_3470233	Microbiology Department. Complexo Hospitalario Universitario de Vigo	Microbiology Department. Complexo Hospitalario Universitario de Vigo	Alfaya N; Alonso I; Alvarez M; Cabrera JJ; Carballo R; Cores O; Cortizo S; Davina C; Martinez L; Mediero G; Perez S; Potel C; Regueiro B; Rey S; Vasallo FJ; del-Campo V
EPI_ISL_935074, EPI_ISL_1911806, EPI_ISL_3589012, EPI_ISL_7874529	Ministry of Health Turkey	Ministry of Health Turkey	Ayşe Başak Altaş; Fatma Bayrakdar; Gulay Korukluoglu; Gülay Korukluoğlu; Suleyman Yalcin; Süleyman Yalcin; Yasemin Cosgun; Yasemin Coşgun
EPI_ISL_2843164	Ministry of Public Health / Hamad Medical Corporation	Biomedical Research Center (BRC), Qatar University / Qatar Genome Project (QGP)	Asmaa A. Al-Thani. MOPH and HMC: Abdullatif Al-Khal; BRC: Fatiha M. Benslimane; Chadi Saad; Dalia Al-Batesh; Dina Elgakhlab QGP: Fatima H. Al-Kuwari; Einas A. E. Al-Kuwari; Hadi M. Yassine; Hamad E. Al-Romaihi; Hamda Alromaihi; Heba A. Al-Khatib; Mashael A. Al-Bader; Mohammed Al-Thani; Muna A. S. Al-Maslamani; Oal Al-Jamal; Peter V. Coyle; Reham A. El-Kahlout. QBB: Tasneem Al-Hamad; Roberto Bertollini; Salih Al-Marri
EPI_ISL_8208476	Montana Public Health Laboratory	Montana Public Health Laboratory	Ashley Ausman Deborah Gibson; Joy Goffena; Joy Ritter; Michelle Mozer
EPI_ISL_3704337	NUPIT/UFPE	WallauLab on behalf of Fiocruz COVID-19 Genomic Surveillance Network	Alexandre Freitas da Silva; Cassia Docena; Constância Flávia Junqueira Ayres; Filipe Zimmer Dezordi; Gabriel Luz Wallau; Gustavo Barbosa de Lima; Lais Ceschini Machado; Lilian Caroliny Amorim Silva; Maira Galdino da Rocha Pitta; Marcelo Henrique dos Santos Paiva; Matheus Filgueira Bezerra; Michelly Cristiny Pereira; Rômulo Pessoa e Silva; Sinval Pinto Brandão Filho
EPI_ISL_2180060	National Center of Infectious and Parasitic Diseases	National Center of Infectious and Parasitic Diseases	Alexiev et al
EPI_ISL_402125	National Institute for Communicable Disease Control and Prevention (ICDC) Chinese Center for Disease Control and Prevention (China CDC)	National Institute for Communicable Disease Control and Prevention (ICDC) Chinese Center for Disease Control and Prevention (China CDC)	Chen; Dai; F.-H.; Hu, Y.; J.-H.; J.-J.; J.-L. and Zhu; Liu, Y.; Pei; Q.-M.; She; Song; T.-Y.; Tao; Tian; Wang; Wang, W.; Wu, F.; Xu, L.; Y.-L.; Y.-M.; Y.-Y.; Y.-Z.; Yu, B.; Z.-G.; Z.-W.; Zhang; Zhao, S.; Zheng
EPI_ISL_1827589	National Institute for Communicable Diseases,National Health Laboratory Services, Gauteng, South Africa	National Institute for Communicable Diseases of the National Health Laboratory Service	Amoako DG; Bhiman JN; Ismail A; Mahlangu B; Mohale T; Ntuli N; Scheepers C
EPI_ISL_1665234	National Platform bis UMONS/Jolimont	National Platform bis UMONS/Jolimont	François Dufrasne; Gautier Detry; Guillaume Bayon-Vicente; Ruddy Wattiez
EPI_ISL_1317637, EPI_ISL_1317646	Nordland Hospital - Bodo, Laboratory Department, Molecular Biology Unit	Norwegian Institute of Public Health, Department of Virology	Atiya R Ali; Debech Nadia; Engebretsen Serina Beate; Garcia Llorente Ignacio; Hilde Elshaug; Hilde Vollan; Jon Bråte; Kamilla Heddeland Instefjord; Karoline Bragstad; Kathrine Stene-Johansen; Marie Paulsen Madsen; Olav Hungnes; Pedersen Benedikte Nevjen; Rasmus Riis Kopperud
EPI_ISL_2517869	Northumbria University / South Tees Hospitals NHS Foundation Trust / North Cumbria Integrated Care NHS Foundation Trust / North Tees and Hartlepool NHS Foundation Trust / Newcastle Hospitals NHS Foundation Trust	COVID-19 Genomics UK (COG-UK) Consortium	Andrew Nelson; Brendan Payne; Clive Graham; Darren L Smith; Debra Padgett; Edward Barton; Emma Swindells; Garren Scott; Gary Black; Gary Eltringham; Giles S Holt; Greg R Young; Jane Greenaway; Jennifer Collins; John Allan; Joshua Loh; Lynn Dover; Matthew Bashton; Mohammad A Tariq; Paul Baker; Sarah Essex; Steve Liggett; Wen C Yew; Yusri Taha
EPI_ISL_7976245	Northwestern Memorial Hospital	Northwestern University - Center for Pathogen Genomics and Microbial Evolution	Chad J. Achenbach; Chao Qi; Egon A. Ozer; Judd F. Hultquist; Lacy M. Simons; Lawrence J. Jennings; Michael G. Ison; Ramon Lorenzo-Redondo; Taylor J. Dean
EPI_ISL_2928905	Oregon State Public Health Laboratory	Oregon State Public Health Laboratory	Eugene Yeboah; John Fontana and Shane Sevey; Laura Tsaknaridis; Rafia Razzaque; Vanda Makris
EPI_ISL_6294609, EPI_ISL_7350045, EPI_ISL_7465200	Originating lab: Wales Specialist Virology Centre Sequencing lab: Pathogen Genomics Unit	Public Health Wales Microbiology Cardiff Wales Specialist Virology Centre	Alec Birchley; Alexander Adams; Amy Gaskin; Angela Marchbank; Bree Gatica-Wilcox; Catherine Moore; Jason Coombes; Joanne Watkins; Joel Southgate; Johnathan Evans; Laura Gifford; Lauren Gilbert; Lee Graham; Malorie Perry; Matthew Bull; Nicole Pacchiarini; Sally Corden; Sara Kumziene-Summerhayes; Sara Rey; Sarah Taylor; Simon Cottrell; Sophie Jones; Tom Connor
EPI_ISL_6002335	Orlickoustecka nemocnice	University Hospital Hradec Kralove	Helena Kovarikova; Lenka Rysava; Marketa Gancarcikova; Monika Berankova
EPI_ISL_4119464	Oslo University Hospital, Department of Microbiology	Norwegian Institute of Public Health, Department of Virology	Arvind Yegambaram Meenakshi Sundaram; Cathrine Fladeby; Garcia Llorente Ignacio; Gregor D. Gilfillan; Hilde Elshaug; Hilde Vollan; Jon Bråte; Kamilla Heddeland Instefjord; Karoline Bragstad; Kathrine Stene-Johansen; Line Victoria Moen; Lise Andresen; Mariann Nilsen; Mona Holberg-Petersen; Olav Hungnes; Pedersen Benedikte Nevjen; Pål Marius Bjørnstad; Rasmus Riis Kopperud; Teodora Plamenova Ribarska
EPI_ISL_3078829	Oxford Viromics, NDM, University of Oxford; Oxford University Hospitals; Basingstoke and North Hampshire Hospital	COVID-19 Genomics UK (COG-UK) Consortium	Alex Mobbs; Amy Trebes; Anita Justice; Catrin Moore; Christophe Fraser; David Bonsall; David Buck; Emma Wise; George Macintyre; Jessica Lynch; John Todd; Mariateresa de Cesare; Matilde Mori; Monique Andersson; Nathan Moore; Nick Cortes; Robert Shaw; Stephen Kidd; Tanya Golubchik; Timothy Peto
EPI_ISL_5646133	PSF DR ANTONIO PIRES DE ALMEIDA PORTO FELIZ	Instituto Butantan	Antonio Jorge Martins; Claudia Renata dos Santos Barros; David Schlesinger; Debora Botequio Moretti; Dimas Tadeu Covas; Elaine Cristina Marqueze; Elaine Vieira Santos; Evandra Strazza Rodrigues; Heidge Fukumasu; Jayme Augusto de Souza-Neto; José Salvatore Leister Patané; Luiz Alcantara; Luiz Lehmann Coutinho; Maria Carolina Elias; Maurício Lacerda Nogueira; Rafael dos Santos Bezerra; Raul Machado Neto; Rejane Maria Tommasini Grotto; Ricardo Haddad; Sandra Coccuzzo Sampaio Vessoni; Simone Kashima; Svetoslav Nanev Slavov; Vincent Louis Viala
EPI_ISL_3933315, EPI_ISL_4096617	Pandemic Response Lab - NYC	Pandemic Response Lab, R&D	Cybill del Castillo; Dylan Law; Haiping Hao; Henry Lee; Isabel Fernandez Escapa; Jon Laurent; Katharine Nelson; Melissa Hopkins; Michael Hammerling; Pradeep Bugga; Shinyoung Clair Kang; Sol Rey; William Ward
EPI_ISL_3197396	Pathogen Genomics Center, National Institute of Infectious Diseases	Pathogen Genomics Center, National Institute of Infectious Diseases	Kentaro Itokawa; Makoto Kuroda; Masanori Hashino; Rina Tanaka; Tsuyoshi Sekizuka
EPI_ISL_5306503	Pathology West - NSW Health Pathology	NSW Health Pathology - Institute of Clinical Pathology and Medical Research; Westmead Hospital; University of Sydney	Arnott A.; Draper J.; Gall M.; Martinez E.; Rockett R.; Sintchenko V.; on behalf of ICPMR
EPI_ISL_2188122	Philippine Red Cross Logistics and Multipurpose Center	Philippine Genome Center	Alethea R. de Guzman; Anna Ong-Lim; Arianne A. Zamora; Asia Louisa U. Chong; Benedict A. Maralit; Candice Francheska B. Tambaoan; Carlo M. Lapid; Celia Carlos; Devon Ray Pacial; Edsel Maurice Salvaña; El King D. Morado; Elcid Aaron R. Pangilinan; Eva Maria Cutiongco-de la Paz; Francis A. Tablizo; Irish Coleen A. Asin; Jaime C. Montoya; Jan Michael C. Yap; Jo-Hannah S. Llames; John Q. Wong; Joshua Gregor A. Dizon; Juan Antonio R. Magalang; Karol Sophia Agape R. Padilla; Kenneth M. Kim; Kris P. Punayan; Marc Edsel C. Ayes; Marc Jerrone R. Castro; Maria Rosario Singh-Vergeire and Cynthia P. Saloma; Maria Sofia L. Yangzon; Marissa Alejandria; Razel Nikka M. Hao; Rianna Patricia S. Cruz; Sheila Mae M. Araiza
EPI_ISL_5569914	Philippine Red Cross- Batangas Chapter Molecular Laboratory	Philippine Genome Center	Alethea R. de Guzman; Alyssa Joyce E. Telles; Anna Ong-Lim; Arianne A. Zamora; Benedict A. Maralit; Carlo M. Lapid; Celia Carlos; Cynthia P. Saloma; Devon Ray Pacial; Diomedes A. Cariño; Edsel Maurice Salvana; El King D. Morado; Elcid Aaron R. Pangilinan; Eva Maria Cutiongco-de la Paz; Francis A. Tablizo; Henrietta Marie Rodriguez; Jaime C. Montoya; Jan Michael C. Yap; Jarvin E. Nipales; Jo-Hannah S. Llames; John Michael Egana; John Q. Wong; Joshua Gregor A. Dizon; Joshua Jose Endozo; Juan Antonio R. Magalang; Karol Sophia Agape R. Padilla; Kris P. Punayan; Kristina Patriz Dela Cruz; Lindsay Clare D.L. Carandang; Ma. Exanil Plantig; Marc Edsel C. Ayes; Maria Rosario Singh-Vergeire; Maria Sofia L. Yangzon; Marielle M. Gamboa; Marissa Alejandria; Niña Francesca Bustamante; Razel Nikka M. Hao; Renato Jacinto Q. Mantaring; Rianna Patricia S. Cruz; Shiela Mae M. Araiza; Yvonne Valerie Austria; Zipporah Mariebelle R. Enriquez; Zyrel V. Mollejon
EPI_ISL_3391074	Plateau technique Biomer Metz Verriers	Department of Virology, Henri Mondor University Hospital, Assistance Publique Hôpitaux de Paris, Université	Alexandre Soulier; Christophe Rodriguez; Elisabeth Trawinski; Guillaume Gricourt; Jean-Michel Pawlotsky; Melissa N'Debi; Slim Fourati; Vanessa Demontant

		Paris-Est Créteil, INSERM U955	
EPI_ISL_1524087	Public Health Authority of the Slovak Republic	Laboratory of Genomics and Bioinformatics, Comenius University Science Park	Anna Gičová; Diana Rusňáková; Jaroslav Budiš; Miroslav Böhmer; Tatiana Sedláčková; Tomáš Szemes
EPI_ISL_2698835	Public Health Laboratory, Minnesota Department of Health	University of Minnesota Genomics Center	Corbin Dirkx; Daryl M. Gohl; Jaquelyn Kuriger-Laber; John Garbe; and Sean Wang
EPI_ISL_4369218, EPI_ISL_4372037, EPI_ISL_4372955, EPI_ISL_4373365	Quest Diagnostics Incorporated	Centers for Disease Control and Prevention Division of Viral Diseases, Pathogen Discovery	A. Gerasimova; A. Perez; B. Anderson; Benjamin Rambo-Martin; Christopher Gulvick; Clinton Paden; Dakota Howard; Dhwani Batra; Duncan MacCannell; Erisa Sula; F. Lacbawan; I. Shlyakhter; Jason Caravas; K. Livingston; Kristine Lacek; L. Bernstein; M. Hua; Matthew Schmerer; P. Tanpaiboon; Peter Cook; R. Kagan; R. Owen; R. Rolando; S. Rosenthal; Scott Sammons; Shatavia Morrison; Tymeckia Kendall; Victoria Caban Figueroa; Y. Liu; Yvette Unoarumhi
EPI_ISL_2893914	REUNILAB	CNR Virus des Infections Respiratoires - France SUD	Antonin Bal; Bruno Lina; Gregory Destras; Gwendolyne Burfin; Hadrien Regue; Laurence Josset; Martine Valette; Quentin Semanas
EPI_ISL_1628780	REUNILAB	UMR PIMIT	Dr Camille Lebarbenchon; Dr David A Wilkinson; Dr Patrick Mavingui; Magali Turpin
EPI_ISL_1409540	Randox Laboratories	Wellcome Sanger Institute for the COVID-19 Genomics UK (COG-UK) Consortium	Cordelia Langford; David K. Jackson; Dominic Kwiatkowski; Ewan Harrison; Ian Johnston; Jeffrey Barrett; John Sillitoe on behalf of the Wellcome Sanger Institute COVID-19 Surveillance Team; Randox Laboratories and Alex Alderton; Roberto Amato; Sonia Goncalves
EPI_ISL_2473932	Reditus Laboratories	Reditus Laboratories	Alexa Eichelberger; Cassy Philips; Joshua J. Geltz; M.S.; Ph.D.; Rex Dyer; Robert M. Sgambelluri
EPI_ISL_8039057	Regionalspital Emmental AG Burgdorf, Labor	Institute for Infectious Diseases, University of Bern	Alban Ramette; Christian Baumann; Cora Sägesser; Franziska Suter-Riniker; Loïc Borcard; Miguel A Terrazos Miani; Nicole Liechti; Pascal Bittel; Peter Keller; Sonja Gempeler; Stefan Neuenschwander; Stephen L Leib
EPI_ISL_2860081, EPI_ISL_4715142	Research Institute for Tropical Medicine, Inc. (RITM)	Philippine Genome Center	Alethea R. de Guzman; Anna Ong-Lim; Arianne A. Zamora; Benedict A. Maralit; Carlo M. Lapid; Celia Carlos; Cynthia P. Saloma; Devon Ray Pacial; Diomedes A. Carino; Diomedes A. Cariño; Edsel Maurice Salvana; Edsel Maurice Salvaña; El King D. Morado; Elcid Aaron R. Pangilinan; Eva Maria Cutiongco-de la Paz; Francis A. Tablizo; Henrietta Marie Rodriguez; Jaime C. Montoya; Jan Michael C. Yap; Jarvin E. Nipales; Jo-Hannah S. Llames; John Q. Wong; Joshua Gregor A. Dizon; Juan Antonio R. Magalang; Karol Sophia Agape R. Padilla; Kenneth M. Kim; Kris P. Punayan; Krisitna Patriz Dela Cruz; Kristina Patriz Dela Cruz; Lindsay Claire D.L. Carandang; Lindsay Clare D.L. Carandang; Ma. Exanil Plantig; Marc Edsel C. Ayes; Maria Rosario Singh-Vergeire; Maria Rosario Singh-Vergeire and Cynthia P. Saloma; Maria Sofia L. Yangzon; Marielle M Gamboa; Marielle M. Gamboa; Marissa Alejandria; Nina Francesca Bustamante; Niña Francesca Bustamante; Razel Nikka M. Hao; Renato Jacinto Q. Mantaring; Rianna Patricia S. Cruz; Sheila Mae M. Araiza; Shiela Mae M. Araiza; Yvonne Valerie Austria; Zipporah Mariebelle R. Enriquez; Zyrel V. Mollejon
EPI_ISL_3080448, EPI_ISL_4309658	Respiratory Virus Unit, Microbiology Services Colindale, Public Health England	COVID-19 Genomics UK (COG-UK) Consortium	PHE Covid Sequencing Team
EPI_ISL_7840805	Rosalind Franklin Laboratory	Wellcome Sanger Institute for the COVID-19 Genomics UK (COG-UK) Consortium	Cordelia Langford; David K. Jackson; Dominic Kwiatkowski; Donald Fraser; Ewan Harrison; Ian Johnston; Jeffrey Barrett; John Sillitoe on behalf of the Wellcome Sanger Institute COVID-19 Surveillance Team; Rob Howes; Roberto Amato; Sonia Goncalves; Suki Lee; The Rosalind Franklin Laboratory and Alex Alderton
EPI_ISL_2765174, EPI_ISL_3195724, EPI_ISL_8097004	SARS-CoV-2 testing team, National Institute of Infectious Diseases	Pathogen Genomics Center, National Institute of Infectious Diseases	Hazuka Y Furihata; Hiromizu Takahashi; Kentaro Itokawa; Makoto Kuroda; Masanori Hashino; Masumichi Saito; Naomi Nojiri; Nozomu Hanaoka; Rina Tanaka; Tsuguto Fujimoto; Tsuyoshi Sekizuka
EPI_ISL_3385800	SECRETARIA DE SALUD DE SAN ANDRES ISLA	Instituto Nacional de Salud- Dirección de Investigación en Salud Pública	Carlos Franco-Muñoz; Carmen Osorio; Diana Malo; Diego A. Álvarez-Díaz; Diego Andrés Prada; Gerardo Santamaría; Hector Alejandro Ruiz-Moreno; Jhonnatan Reales-González; Jorge Rivera; Juan Camilo Martinez; Julian Naizaque; Katherine Laiton-Donato; Lisseth Pardo; Magdalena Wiesner; Marcela Mercado-Reyes; Maria T. Herrera-Sepúlveda; Marta Lopez Blanco; Martha Lucia Ospina Martinez; Paola Rojas; Sergio Gomez; Sheryll Corchuelo; Ángela Alarcon Cruz
EPI_ISL_3255757	SK-Roy Romanow Provincial Laboratory	National Microbiology Laboratory (NML)	Alanna Senecal; Amanda Lang; Anna Majer; Anneliese Landgraff; CanCOGeN's metadata curation team; Darian Hole; Elsie Grudeski; Gary Van Domselaar; Grace Seo; Jennifer Tanner; Jessica Minion; Kara Loos; Keith MacKenzie; Kirsten Biggar; Madison Chapel; Meredith Faires; Morag Graham; Natalie Knox; Nathalie Bastien; Philip Mabon; Public Health Agency of Canada CanCOGeN team; Rachel DePaulo; Rhiannon Huzarewich; Russell Mandes; Ryan McDonald; Shari Tyson; Timothy Booth; Yan Li
EPI_ISL_1595690	SYNLAB	GIGA Medical Genomics	Bouchra Boujemla; Cécile Meex; Keith Durkin; Maria Artesi; Marie-Pierre Hayette; Nathalie Renotte; Pierrette Melin; Raphaël Boreux; Sébastien Bontems; Vincent Bours
EPI_ISL_5202214	SYNLAB	LSPSDS	Alejandro Gomez Lopez; Gabriela Delgado Murcia; Johana Hernandez Toloza; Marcela Castano Rodriguez; Marcela Mercado
EPI_ISL_4230406	SYNLAB MVZ Hamburg	Robert Koch Institute	
EPI_ISL_1849542, EPI_ISL_2130997	SYNLAB MVZ Heidelberg	Robert Koch Institute	
EPI_ISL_6467409	SYNLAB MVZ Leinfelden-Echterdingen	Robert Koch Institute	
EPI_ISL_2111707	SYNLAB MVZ Leverkusen	Robert Koch Institute	
EPI_ISL_6437701	Salud Digna	Instituto Nacional de Medicina Genomica	Abraham Campos-Romero; Cedro-Tanda A; Cruz-Islas Jazmin; Escobar-Arrazola; Gonzalez-Barrera D; Herrera-Montalvo LA.; Hidalgo-Miranda A; Luna-Ruiz Marco; M; Mendoza-Vargas A; Moreno-Camacho José Luis; Munguia-Garza P; Ramirez-Vega O; Rangel-DeLeon D; Reyes-Grajeda JP; Rodriguez-Gallegos Jorge
EPI_ISL_5986683	Scripps Medical Laboratory	Andersen lab at Scripps Research	Ellen Stefanski; Ian Mchardy; SEARCH Alliance San Diego with Michael Quigley
EPI_ISL_8185050	Sema4	Sema4	Angela Yannes; Bisram Deocharan; Feras Hantash; Matthew Capozziello; Rui Gu; Victoria Parkington; Zdenek Markovic
EPI_ISL_7751354, EPI_ISL_7752987, EPI_ISL_7753061	Servicio Virosis Respiratorias-Departamento Virologia-INEI	Instituto Nacional Enfermedades Infecciosas C.G.Malbran	Avaro M.; Baumeister E.; Benedetti E.; Campos J.; Cisterna D.; Dattero ME; De Belder D.; Haim MS.; Lorenzo F.; Molina V.; Perandones C.; Poklepovich T.; Pontoriero A.; Russo M.; Sanchez Loria J.; Tuduri E.
EPI_ISL_4083393, EPI_ISL_4083412, EPI_ISL_5159078, EPI_ISL_6694676, EPI_ISL_6694755	Servicio Virosis Respiratorias-Departamento Virología-INEI	Instituto Nacional Enfermedades Infecciosas C.G.Malbran	Avaro M.; Baumeister E.; Benedetti E.; Campos J.; Cisterna D.; Dattero ME; De Belder D.; Haim MS.; Lorenzo F.; Molina V.; Perandones C.; Poklepovich T.; Pontoriero A.; Russo M.; Sanchez Loria J.; Tuduri E.
EPI_ISL_2934733	Servicio de Microbiología Clínica (Complejo Hospitalario de Navarra, Pamplona)	Centro de Secuenciación NASERTIC	Ana Miqueleiz; Ana Navascués; Carmen Ezpeleta Baquedano
EPI_ISL_8136296	Shamir Medical Center (Asaf Harofe)	Shamir Medical Center (Asaf Harofe)	Abu Hamad Ramzia; Adina Bar Chaim; Anna Vishnevsky; Chen Weiner; Nir Rainy; Patricia Benveniste-Lekovitz; Reut Sorek Abramovich; Yevgeni Yegorov
EPI_ISL_1420584, EPI_ISL_1602954, EPI_ISL_1606093, EPI_ISL_1617543, EPI_ISL_2211128, EPI_ISL_2256570, EPI_ISL_2418725			
see above	Swedish national genomic surveillance program of SARS-CoV-2	The Public Health Agency of Sweden	Alma Brolund; Maria Lind Karlberg; Maximilian Riess; Swedish national genomic surveillance program of SARS-CoV-2
EPI_ISL_7952710	Synlab Medilab	Karolinska University Hospital Huddinge	Annika Tiveljung Lindell; Henning Onsbring; Jan Albert; Karina Hentrich; Lynda Eneh; Martin Ekman; Natalija Gerasimcik; Robert Dyrdak; Sandra Broddesson; Shambhu Ganeshappa Aralaguppe; Tanja Normark; Tobias Allander; Valtteri Wirta; Zhibing Yun
EPI_ISL_6327824	TSHEPONG LABORATORY	National Institute for Communicable Diseases of the National Health Laboratory Service	Amoako DG; Bhiman JN; Everatt J; Ismail A; Mahlangu B; Mnguni A; Mohale T; Ntuli N; Scheepers C
EPI_ISL_2678157, EPI_ISL_6250639	The Caribbean Public Health Agency	Carrington Lab, Department of Preclinical Sciences, Faculty of Medical Sciences, The University of the West Indies, St Augustine Campus	Anushka Ramjag; Arianne Brown-Jordan; Avery Hinds; Christine V. F. Carrington; Christopher Oura; Gabriel Escobar; Nikita S. D. Sahadeo; Nuno Faria; Oliver Pybus; Risha Singh; Roshan Parasram; Sarah Hill; Simone Keizer-Beache; SueMin Nathaniel; Vernie Ramkissoon
EPI_ISL_1581433	UAB InMedica	Vilnius University Hospital Santaros Klinikos, Center of Laboratory Medicine	Daniel Naumovas; Dovile Ezerskyte; Gytis Dudas; Ingrida Olendraite; Laimonas Griskevicius; Ligita Raugaite; Mindaugas Stoskus; Monika Katenaite; Rimvydas Norvilas
EPI_ISL_1966460	UBS IV GUARARAPES	Instituto Butantan / Mendelics	Antonio Jorge Martins; Bianca Cechetto Carlos. Mendelics: Bibiana Santos; Claudia Renata dos Santos Barros; Cíntia Bittar; David Schlesinger. Hemocentro Ribeirão Preto: Simone Kashima; Debora Botequio Moretti; Elaine Cristina Marqueze; Elaine Vieira dos Santos; Elisangela Chicaroni Mattos; Erika Freitas; Evandra Strazza Rodrigues; Felipe Allan da Silva da Costa; Flavia Aburjaile; Fábio Sossai Possebon; Guilherme Campos; Guilherme Targino Valente; Heidge Fukumasu. USP-Botucatu: Rejane Maria Tommasini Grotto; Helena Lage Ferreira; Instituto Butantan: Dimas Tadeu Covas; Jardelina de Souza Todao Bernardino; Jayme A. Souza-Neto; Jessika Cristina Chagas Lesbon; Jorge A. Petroli Marchesi; José Salvatore Leister Patané; João Paulo Kitajima; João Pessoa Araújo Jr.; Leila Sabrina Ullmann; Loyze Paola Oliveira de Lima; Luiz Aurelio de Campos Crispin. Centro de Genômica Funcional da ESALQ: Luiz Lehmann Coutinho; Luiz Carlos Junior de Alcantara; Lívia Sacchetto; Maisa C. Pereira Parra; Maria Carolina Elias; Marta Giovanetti; Marília Moraes; Maurício Lacerda Nogueira. Prefeitura de Sao Paulo: Melissa Palmieri.; Patricia Akemi Assato; Paula Rahal; Paulo Inacio da Costa; Rafael dos Santos Bezerra; Raquel de Lello Rocha Campos Cassano. NGS Soluções Genômicas: Pilar Drummond Sampaio Corrêa Mariani. FZEA-USP Pirassununga: Mirele Daiana Poleti; Raul Machado Neto; Ricardo Augusto Brassaloti; Ricardo Haddad; Rodrigo Tocantins Calado. FAMERP-SJRP: Cecília Artico Banho; Sandra Coccuzzo Sampaio; Svetoslav Nanev Slavov; Vagner Fonseca; Vincent Louis Viala
EPI_ISL_5646922	UNIDADE DE VIGILANCIA EM SAUDE	Instituto Butantan	Antonio Jorge Martins; Claudia Renata dos Santos Barros; David Schlesinger; Debora Botequio Moretti; Dimas Tadeu Covas; Elaine Cristina Marqueze; Elaine Vieira Santos; Evandra Strazza Rodrigues; Heidge Fukumasu; Jayme Augusto de Souza-Neto; José Salvatore Leister Patané; Luiz Alcantara; Luiz Lehmann Coutinho; Maria Carolina Elias; Maurício Lacerda Nogueira; Rafael dos Santos Bezerra; Raul Machado Neto; Rejane Maria Tommasini Grotto; Ricardo Haddad; Sandra Coccuzzo Sampaio Vessoni; Simone Kashima; Svetoslav Nanev Slavov; Vincent Louis Viala
EPI_ISL_5647737	UNIDADE RESPIRATORIA NOVA HORTOLANDIA	Instituto Butantan	Antonio Jorge Martins; Claudia Renata dos Santos Barros; David Schlesinger; Debora Botequio Moretti; Dimas Tadeu Covas; Elaine Cristina Marqueze; Elaine Vieira Santos; Evandra Strazza Rodrigues; Heidge Fukumasu; Jayme Augusto de Souza-Neto; José Salvatore Leister Patané; Luiz Alcantara; Luiz Lehmann Coutinho; Maria Carolina Elias; Maurício Lacerda Nogueira; Rafael dos Santos Bezerra; Raul Machado Neto; Rejane Maria Tommasini Grotto; Ricardo Haddad; Sandra Coccuzzo Sampaio Vessoni; Simone Kashima; Svetoslav Nanev Slavov; Vincent Louis Viala
EPI_ISL_2208904	UPA DR FRANCO DA ROCHA	Instituto Butantan / Mendelics	Antonio Jorge Martins; Claudia Renata dos Santos Barros; David Schlesinger; Debora Botequio Moretti; Dimas Tadeu Covas; Elaine Cristina Marqueze; Elaine Vieira Santos; Evandra Strazza Rodrigues; Heidge Fukumasu; Jayme Augusto de Souza-Neto; José Salvatore Leister Patané; Luiz Alcantara; Luiz Lehmann Coutinho; Maria Carolina Elias; Maurício Lacerda Nogueira; Rafael dos Santos Bezerra; Raul Machado Neto; Rejane Maria Tommasini Grotto; Ricardo Haddad; Sandra Coccuzzo Sampaio Vessoni; Simone Kashima; Svetoslav Nanev Slavov; Vincent Louis Viala
EPI_ISL_2693307	UW Virology Lab	UW Virology Lab	Alexander Greninger; Hong Xie; Keith R Jerome; Lasata Shrestha; Meei-Li Huang; Michelle Lin; Nathan Breit; Noah R. Baker; Patrick Mathias; Pavitra Roychoudhury; Ricardo Perez; Robert J. Livingston; Sean Ellis; Shah Mohamed Bakhash; Tien V. Nguyen
EPI_ISL_3568784	Unidad de Investigacion Medica de Yucatan (UIMY)	Unidad de Genomica Avanzada	; Alejandra Garcia-Gasca; Alejandra Hernandez-Teran; Alejandro Sanchez-Flores; Alfredo Herrera-Estrella; Alicia OcaÃ±a-Mondragon; Andreu Comas-Garcia; Angel Gustavo Salas-Lais; Antonio Loza Roman; Bernardo Martinez-Miguel; Blanca Taboada; Brenda Irasema Maldonado-Meza; Bruno Gomez-Gil; Carla Ivon Herrera-Najera; Carlos F. Arias; Celia Boukadida; Celida Duque Molina; Celida Martinez- Rodriguez; Clara Esperanza Santacruz-Tinoco; Concepcion Grajales-MuÃ±iz; Consorcio Mexicano de Vigilancia Genomica (CoViGen-Mex). Authors (in alphabetical order): Julio Elias Alvarado-Yaah; Cristobal Chaidez-Quiroz; Daniel Fregoso-Rueda; Daniel Lira Morales; Eduardo Becerril-Vargas; Fernando Fontove-Herrera; Fidencio Mejia-Nepomuceno; Francisco Pulido; Gloria Elena Espinosa-Ayala; Gloria Maria Molina-Salinas; Gloria Vazquez; Hector Esteban Paz-Juarez; Hector Montoya-Fuentes; Helen Haydee Fernanda Ramirez-Plascencia; Irvin Gonzalez-Lopez; Jean Pierre Gonzalez; Jesus Hernandez; Joel Armando Vazquez-Perez.; Jorge Salas-Hernandez; Jose Antonio Enciso-Moreno; Jose Arturo Martinez-Orozco; Jose Esteban MuÃ±oz-Medina; Jose de Jesus NuÃ±ez-Contreras; Juan Bautista Chale-Dzul; Julissa Enciso-Ibarra; Luis Alberto Ochoa-Carrera; Margarita Matias-Florentino; Maria Guadalupe Santiago-Mauricio; Maria Guadalupe de Jesus Mireles-Rivera; Mario Mujica-Sanchez; Marissa Perez-Garcia; Nelly Selem-Mojica; Pavel Isa; Ricardo Ciria Merce; Ricardo Grande; Rosa Maria Gutierrez Rios; Santiago avila-Rios; Selene Zarate; Susana Lopez; Veronica Mata-Haro; Victor Eduardo Garcia-Arias; Victor Hugo Borja-Aburto
EPI_ISL_7780468	Unidad de Investigación Médica de Yucatán (UIMY)	Centro de Investigación en Enfermedades Infecciosas (CIENI),	Alejandra García-Gasca; Alejandra Hernández-Terán; Alejandro Sánchez-Flores; Alfredo Herrera-Estrella; Alicia Ocaña-Mondragón; Andreu Comas-García; Angel Gustavo Salas-Lais; Antonio Loza Román; Bernardo Martínez-Miguel; Blanca Taboada; Brenda Irasema Maldonado-Meza; Bruno Gómez-Gil; Carla Ivón Herrera-Najera; Carlos F. Arias; Celia Boukadida; Clara Esperanza Santacruz-Tinoco; Concepción Grajales-Muñiz; Consorcio Mexicano de Vigilancia Genómica (CoViGen-Mex). Authors (in alphabetical order): Julio Elias Alvarado-Yaah; Cristóbal Cháidez-Quiróz; Célida Duque Molina; Célida

		Instituto Nacional de Enfermedades Respiratorias (INER)	Martínez-Rodríguez; Daniel Fregoso-Rueda; Daniel Lira Morales; Eduardo Becerril-Vargas; Eduardo Rivera-Martínez; Fernando Fontove-Herrera; Fidencio Mejía-Nepomuceno; Francisco Pulido; Gabriel Chavira-Trujillo; Gloria Elena Espinosa-Ayala; Gloria María Molina-Salinas; Gloria Vazquez; Hector Montoya-Fuentes; Helen Haydee Fernanda Ramírez-Plascencia; Irvin González-López; Jean Pierre González; Jesús Hernández; Joel Armando Vázquez-Pérez.; Jorge Salas-Hernández; José Antonio Enciso-Moreno; José Arturo Martínez-Orozco; José Esteban Muñoz-Medina; José de Jesús Nuñez-Contreras; Juan Bautista Chale-Dzul; Julissa Enciso-Ibarra; Kathia Elizabeth Tapia-Diaz; Luis Alberto Ochoa-Carrera; Margarita Matías-Florentino; Mario Mújica-Sánchez; Marissa Perez-Garcia; María Eugenia Jiménez-Corona; María Guadalupe Santiago-Mauricio; María Guadalupe de Jesús Mireles-Rivera; Nelly Sélem-Mojica; Pavel Isa; Ricardo Ciria Merce; Ricardo Grande; Rosa María Gutiérrez Rios; Rosario Vazquez-Larios; Santiago Ávila-Ríos; Selene Zárate; Susana Lopez; Verónica Mata-Haro; Victor Eduardo García-Arias; Víctor Hugo Borja-Aburto
EPI_ISL_2898445	Unilabs	Karolinska University Hospital	Annelie Bjerkner; Isak Sylvin; Jan Albert; Karolina Ininbergs; Lina Guerra Blomqvist; Lynda Eneh; Martin Ekman; Martina Wahlund; Robert Dyrdak; Sandra Broddesson; Tanja Normark; Tobias Allander; Valtteri Wirta; Zhibing Yun
EPI_ISL_3477572	Universidad del Valle, LDAB-Laboratorio de Diagnostico de Agentes Biologicos	Universidad del Valle, TAO-Lab, VIREM, NEAS Network	Andres Castillo; Beatriz Parra & COVID-19 Team Univalle; Diana López-Alvarez; Nelson Rivera Franco
EPI_ISL_983044	University Health Network/Mount Sinai Hospital Department of Microbiology	Ontario Institute for Cancer Research	Aimee Paterson; Allison McGeer; Angel Liu; Bernard Lam; Cassandra Bergwerff; Ilinca Lungu; Jared T. Simpson; Javier Hernandez; Jeff Wrana; Jessica Bourke; Kin Chan; Kuganya Nirmalarajah; Laurence Pelletier; Lawrence E. Heisler; Lubaina Kothari; Marc Mazzulli; Marie-Ming Aynaud; Michael Laszloffy; Patryk Aftanas; Paul Krzyzanowski; Richard de Borja; Samira Mubareka; Seda Barutcu; Tony Mazzulli
EPI_ISL_1241680	University of Liège COVID-19 testing center	GIGA Medical Genomics	Bouchra Boujemla; Cécile Meex; Keith Durkin; Maria Artesi; Marie-Pierre Hayette; Nathalie Renotte; Pierrette Melin; Raphaël Boreux; Sébastien Bontems; Vincent Bours
EPI_ISL_4742149	VITALAB Molecular Laboratory	Philippine Genome Center	Alethea R. de Guzman; Anna Ong-Lim; Arianne A. Zamora; Benedict A. Maralit; Carlo M. Lapid; Celia Carlos; Cynthia P. Saloma; Devon Ray Pacial; Diomedes A. Cariño; Edsel Maurice Salvana; El King D. Morado; Elcid Aaron R. Pangilinan; Eva Maria Cutiongco-de la Paz; Francis A. Tablizo; Henrietta Marie Rodriguez; Jaime C. Montoya; Jan Michael C. Yap; Jarvin E. Nipales; Jo-Hannah S. Llames; John Q. Wong; Joshua Gregor A. Dizon; Juan Antonio R. Magalang; Karol Sophia Agape R. Padilla; Kenneth M. Kim; Kris P. Punayan; Kristina Patriz Dela Cruz; Lindsay Clare D.L. Carandang; Ma. Exanil Plantig; Marc Edsel C. Ayes; Maria Rosario Singh-Vergeire; Maria Sofia L. Yangzon; Marielle M. Gamboa; Marissa Alejandria; Niña Francesca Bustamante; Razel Nikka M. Hao; Renato Jacinto Q. Mantaring; Rianna Patricia S. Cruz; Shiela Mae M. Araiza; Yvonne Valerie Austria; Zipporah Mariebelle R. Enriquez; Zyrel V. Mollejon
EPI_ISL_1371734	Virginia Division of Consolidated Laboratory Services	Virginia Division of Consolidated Laboratory Services	Virginia DCLS
EPI_ISL_6563125	WSSE w Gdansku	Wojewodzka Stacja Sanitarno-Epidemiologiczna w Olsztynie, Laboratorium Badan Epidemiologiczno-Klinicznych	Aleksandra Kobiatko; Barbara Dolinska; Emilia Tarabasz; Ewa Liszewska; Marta Lukian; Monika Czerminska; Patryk Bielecki; Paulina Rozycka; Sylwia Krzetowska; Tomasz Jakubczak
EPI_ISL_8209445	Washington State Department of Health Public Health Laboratories	Washington State Department of Health Public Health Laboratories	Alex Lathum; Ardizon Valdez; Avi Singh; Claire Howell; Denny Russell; Drew MacKellar; Holly Halstead; JohnAric Peterson; Kathryn Sickles; Kristin Roche; Lisa Jones; Philip Dykema; Rebecca Cao
EPI_ISL_2534262	Wichita State University - Molecular Diagnostics Lab	Kansas Health and Environmental Lab	Ben Olsen; Jonathan Barnell; Katherine Wiggins; Mike Grose; and Phil Adam
EPI_ISL_3556540	Wisconsin State Laboratory of Hygiene Communicable Disease Division	Wisconsin State Laboratory of Hygiene Communicable Disease Division	Abigail C. Shockey; Alicia J. Mooney; Erika M. Hanson; Kelsey R. Florek; Richard Griesser; Sara Wagner; Tonya Danz
EPI_ISL_2034966	Yale Clinical Virology Lab	Yale Center for Genomic Analysis	Brooke Sullivan; Curt Scharfe; Irina Tikhonova; Kaya Bilguvar; Shrikant Mane
EPI_ISL_2727263	ZARV/NHLS, Department Medical Virology, University of Pretoria	KRISP, KZN Research Innovation and Sequencing Platform	Adriano Mendes; Amy Strydom; Emmanuel SJ; Giandhari J; Lessells R; Micheala Davids; Naidoo Y; Pillay S; Ramphal U; Sim Mayaphi and Marietjie Venter; Tegally H; Wilkinson E; de Oliveira T
EPI_ISL_6666338	Zaklad Diagnostyki Laboratoryjnej Koagulologii i Mikrobiologii	Wojewodzka Stacja Sanitarno-Epidemiologiczna w Rzeszowie, Laboratorium Diagnostyki Medycznej	Anna Nowakowska; Karolina Ostrowska; Katarzyna Wilk; Marzena Baranowska
EPI_ISL_2360232	Zentrallabor Zürich	Institute of Medical Virology	Alexandra Trkola; Annette Audigé; Cyril Shah; Gabriela Ziltener; Guido Bloemberg; Jon Huder; Jürg Böni; Kevin Steiner; Maria Grünberg; Maryam Zaheri; Michael Huber; Riccarda Capaul; Stefan Schmutz; Verena Kufner
EPI_ISL_4295792	unknown	CNR Virus des Infections Respiratoires - France SUD	Antonin Bal; Bruno Lina; Gregory Destras; Gwendolyne Burfin; Hadrien Regue; Laurence Josset; Martine Valette; Quentin Semanas

We gratefully acknowledge the following Authors from the Originating laboratories responsible for obtaining the specimens, as well as the Submitting laboratories where the genome data were generated and shared via GISAID, on which this research is based.

All Submitters of data may be contacted directly via www.gisaid.org

Authors are sorted alphabetically.

Acknowledgement EPI_SET Identifier: EPI_SET_20220328ge

Accession ID	Originating Laboratory	Submitting Laboratory	Authors
EPI_ISL_3347206	ADILAB	Laboratorio Departamental de Salud Publica de Antioquia	Andres F. Cardona-Rios; Gloria Isabel Escobar; Idabely Betancur Ortiz; Juan P. Hernandez-Ortiz; Maria Stella López
EPI_ISL_3276536, EPI_ISL_3276539, EPI_ISL_3276543	ADILAB	Universidad Nacional de Colombia - Laboratorio Genómico One Health	Andres F. Cardona-Rios; Carlos Franco-Muñoz; Carolina Muñoz-Arango; Celeny Ortiz; Daniel O. Maldonado-Perez; Diego A. Álvarez-Díaz; Hector Alejandro Ruiz-Moreno; Idabely Betancur Ortiz; Jorge E. Osorio; Juan P. Hernandez-Ortiz; Karl A Ciuoderis; Katherine Laiton-Donato; Laura Silvana Perez; Lina M. Hurtado; Marcela Mercado-Reyes; Maria Angélica Maya; Maria Stella López; Rita Almanza Payares; Sandra Ines Cano; Simón Villegas Velásquez
EPI_ISL_3298351, EPI_ISL_3298358	AREA DE SALUD ACOSTA	Inciensa, Instituto Costarricense de Investigación y Enseñanza en Nutrición y Salud	Adriana Godínez; Claudio Soto-Garita; Estela Cordero; Francisco Duarte; Hebleen Porras; José Luis Vargas; Mariela Gutiérrez & Joselyn Prado; Melany Calderón
EPI_ISL_3274361, EPI_ISL_3274362	AREA DE SALUD ACOSTA [ACOSTA/SAN JOSE]	Inciensa, Instituto Costarricense de Investigación y Enseñanza en Nutrición y Salud	& Nazareth Ruiz; Adriana Godínez; Caterina Guzmán; Claudio Soto-Garita; Estela Cordero; Francisco Duarte; Hebleen Porras; José Luis Vargas; Mariela Gutiérrez Joselyn Prado; Melany Calderón
EPI_ISL_3274356	AREA DE SALUD ATENAS	Inciensa, Instituto Costarricense de Investigación y Enseñanza en Nutrición y Salud	Adriana Godínez; Caterina Guzmán; Claudio Soto-Garita; Estela Cordero; Francisco Duarte; Hebleen Porras; José Luis Vargas; Mariela Gutiérrez Joselyn Prado; Melany Calderón; Nazareth Ruiz & Benito Vega
EPI_ISL_3298360	AREA DE SALUD CORREDORES	Inciensa, Instituto Costarricense de Investigación y Enseñanza en Nutrición y Salud	Adriana Godínez; Claudio Soto-Garita; Estela Cordero; Francisco Duarte; Hebleen Porras; Joselyn Prado & Susana Mata; José Luis Vargas; Mariela Gutiérrez; Melany Calderón
EPI_ISL_3298355	AREA DE SALUD COTO BRUS	Inciensa, Instituto Costarricense de Investigación y Enseñanza en Nutrición y Salud	Adriana Godínez; Claudio Soto-Garita; Estela Cordero; Francisco Duarte; Hebleen Porras; José Luis Vargas; Mariela Gutiérrez & Joselyn Prado; Melany Calderón
EPI_ISL_3274353, EPI_ISL_3274354, EPI_ISL_3274360	AREA DE SALUD DESAMPARADOS 1 - CLINICA DR. MARCIAL FALLAS	Inciensa, Instituto Costarricense de Investigación y Enseñanza en Nutrición y Salud	& Nazareth Ruiz; Adriana Godínez; Caterina Guzmán; Claudio Soto-Garita; Estela Cordero; Francisco Duarte; Hebleen Porras; José Luis Vargas; Mariela Gutiérrez Joselyn Prado; Melany Calderón; Nazareth Ruiz & Juliana Mora Cortes
EPI_ISL_3274364	AREA DE SALUD DESAMPARADOS 1 - CLINICA DR. MARCIAL FALLAS [GRIFO ALTO/DESAMPARADOS/SAN JOSE]	Inciensa, Instituto Costarricense de Investigación y Enseñanza en Nutrición y Salud	& Nazareth Ruiz; Adriana Godínez; Caterina Guzmán; Claudio Soto-Garita; Estela Cordero; Francisco Duarte; Hebleen Porras; José Luis Vargas; Mariela Gutiérrez Joselyn Prado; Melany Calderón
EPI_ISL_3298371	AREA DE SALUD FORTUNA	Inciensa, Instituto Costarricense de Investigación y Enseñanza en Nutrición y Salud	Adriana Godínez; Claudio Soto-Garita; Estela Cordero; Francisco Duarte; Hebleen Porras; Joselyn Prado & Carolina Arrieta; José Luis Vargas; Mariela Gutiérrez; Melany Calderón
EPI_ISL_3638888	AREA DE SALUD GOICOECHEA 2 - CLINICA DR. JIMENEZ NUÑEZ	Inciensa, Instituto Costarricense de Investigación y Enseñanza en Nutrición y Salud	Adriana Godínez; Claudio Soto-Garita; Estela Cordero; Francisco Duarte; Hebleen Porras; Joselyn Prado & Juan Carlos Cartes; José Luis Vargas; Mariela Gutiérrez; Melany Calderón
EPI_ISL_3274365, EPI_ISL_3274367	AREA DE SALUD MATA REDONDA-HOSPITAL - CLINICA DR. MORENO CAÑAS [SAN JOSE/SAN JOSE]	Inciensa, Instituto Costarricense de Investigación y Enseñanza en Nutrición y Salud	& Nazareth Ruiz; Adriana Godínez; Caterina Guzmán; Claudio Soto-Garita; Estela Cordero; Francisco Duarte; Hebleen Porras; José Luis Vargas; Mariela Gutiérrez Joselyn Prado; Melany Calderón; Nazareth Ruiz & Ricardo González
EPI_ISL_3464535, EPI_ISL_3638898	AREA DE SALUD OREAMUNO-PACAYAS-TIERRA BLANCA	Inciensa, Instituto Costarricense de Investigación y Enseñanza en Nutrición y Salud	Adriana Godínez; Claudio Soto-Garita; Estela Cordero; Francisco Duarte; Hebleen Porras; Joselyn Prado & Carolina Loría; Joselyn Prado & Carolina Loría Acosta; José Luis Vargas; Mariela Gutiérrez; Melany Calderón
EPI_ISL_3638807	AREA DE SALUD PURISCAL-TURRUBARES	Inciensa, Instituto Costarricense de Investigación y Enseñanza en Nutrición y Salud	Adriana Godínez; Claudio Soto-Garita; Estela Cordero; Francisco Duarte; Hebleen Porras; José Luis Vargas; Mariela Gutiérrez & Joselyn Prado; Melany Calderón
EPI_ISL_3298349	AREA DE SALUD SIQUIRRES	Inciensa, Instituto Costarricense de Investigación y Enseñanza en Nutrición y Salud	Adriana Godínez; Claudio Soto-Garita; Estela Cordero; Francisco Duarte; Hebleen Porras; Joselyn Prado & Ileana Chaves; José Luis Vargas; Mariela Gutiérrez; Melany Calderón
EPI_ISL_3639064	AREA DE SALUD TIBAS-URUCA-MERCED - CLINICA DR. CLORITO PICADO	Inciensa, Instituto Costarricense de Investigación y Enseñanza en Nutrición y Salud	Adriana Godínez; Claudio Soto-Garita; Estela Cordero; Francisco Duarte; Hebleen Porras; Joselyn Prado & Johnny Villalobos; José Luis Vargas; Mariela Gutiérrez; Melany Calderón
EPI_ISL_3119719	AULSS 3 Venezia	Istituto Zooprofilattico Sperimentale delle Venezie	Adelaide Milani; Alessia Schivo; Alice Fusaro; Ambra Pastori; Annalisa Salviato; Antonia Ricci; Calogero Terregino; Edoardo Giussani; Elisa Palumbo; Erika Giorgia Quaranta; Isabella Monne; Luca Tassoni
EPI_ISL_3127787, EPI_ISL_3446799	AULSS 3 Venezia	UOSD Genetica e Citogenetica - Azienda ULSS 3 Serenissima; Istituto Zooprofilattico Sperimentale delle Venezie	Adelaide Milani; Alessia Schivo; Alice Fusaro; Ambra Pastori; Annalisa Salviato; Antonia Ricci; Calogero Terregino; Claudia Perini; Edoardo Giussani; Elisa Palumbo; Elisa Squarcina; Erika Giorgia Quaranta; Isabella Monne; Laura Bevilacqua; Laura Squarzon; Luca Sorino; Luca Tassoni; Mosé Favarato
EPI_ISL_3028236, EPI_ISL_3039672	AULSS 6 Euganea	Istituto Zooprofilattico Sperimentale delle Venezie	Adelaide Milani; Alessia Schivo; Alice Fusaro; Ambra Pastori; Annalisa Salviato; Antonia Ricci; Calogero Terregino; Edoardo Giussani; Elisa Palumbo; Erika Giorgia Quaranta; Isabella Monne; Luca Tassoni
EPI_ISL_3430113, EPI_ISL_3430351, EPI_ISL_3430547	Aegis Sciences Corporation	Centers for Disease Control and Prevention Division of Viral Diseases, Pathogen Discovery	Adrian Paskey; Alec Vest; Benjamin Rambo-Martin; Christopher Gulvick; Clinton R. Paden; Cyndi Clark; Dakota Howard; Darlene Wagner; Dhwani Batra; Dillon Nall; Duncan MacCannell; Ethan Sanders; Holly Houdeshell; Jason Caravas; Kara Moser; Matthew Hardison; Matthew Schmerer; Ola Kvalvaag; Patrick Campbell; Peter W. Cook; Rob Case; Scott Sammons; Shatavia Morrison; Shaun Westlund; Vikramsinha Ghorpade; Yvette Unoarumhi
EPI_ISL_3270474, EPI_ISL_3270475	Alaska State Virology Laboratory	Alaska State Virology Laboratory	Elva House; Jack Chen; Jacob Zidek; Lisa Smith; Ph.D.; Stephanie DeRonde
EPI_ISL_3386397	Arizona State University	Arizona State University	Ajeet Bains; Efrem S. Lim; Joshua LaBaer; LaRinda A. Holland; Matthew F. Smith; Nathaniel Johnson; Nicholas J. Mellor; Peter T. Skidmore; Rabia Maqsood; Vel Murugan
EPI_ISL_3276575	Ayudas Diagnosticas Sura	Universidad Nacional de Colombia - Laboratorio Genómico One Health	Andres F. Cardona-Rios; Carlos Franco-Muñoz; Carolina Muñoz-Arango; Celeny Ortiz; Daniel O. Maldonado-Perez; Diego A. Álvarez-Díaz; Hector Alejandro Ruiz-Moreno; Idabely Betancur Ortiz; Jorge E. Osorio; Juan P. Hernandez-Ortiz; Karl A Ciuoderis; Katherine Laiton-Donato; Laura Silvana Perez; Lina M. Hurtado; Marcela Mercado-Reyes; Maria Angélica Maya; Maria Stella López; Rita Almanza Payares; Sandra Ines Cano; Simón Villegas Velásquez
EPI_ISL_3167149	Basurto University Hospital: Clinical Microbiology Laboratory	Biocruces Bizkaia	Ana de la Hoz; Estibaliz Ugalde Zarraga; José Luis Díaz de Tuesta del Arco; Mikel Gallego Rodrigo; Mikel Urrutikoetxea-Gutiérrez; Mª Carmen Nieto Toboso
EPI_ISL_3042076, EPI_ISL_3043350, EPI_ISL_3109639, EPI_ISL_3169783, EPI_ISL_3225218	Berkshire and Surrey Pathology Services Lighthouse Laboratory	Wellcome Sanger Institute for the COVID-19 Genomics UK (COG-UK) Consortium	Berkshire and Surrey Pathology Services Lighthouse Laboratory and Alex Alderton; Cordelia Langford; David K. Jackson; Dominic Kwiatkowski; Ewan Harrison; Ian Johnston; Jeffrey Barrett; John Sillitoe on behalf of the Wellcome Sanger Institute COVID-19 Surveillance Team; Roberto Amato; Sonia Goncalves
EPI_ISL_3145700	BioneXt Lab	Laboratoire national de sante, Microbiology, Microbial Genomics Platform	Anke Wienecke-Baldacchino; Catherine Ragimbeau; Elodie Solarino; Fatu Djabi; Jessica Tapp; Lise Pignon; Raoul Salmon; Tamir Abdelrahman; Thibault Ferrandon; Virginie Jover
EPI_ISL_3602820	British Columbia Centre For Disease Control	BCCDC Public Health Laboratory	Ana Pacagnella; Corrinne Ng; Dan Fornika; John Tyson; Kim Macdonald; Kimia Kamelian; Linda Hoang; Loretta Janz; Mel Krajden; Prystajecky Natalie; Robert Azana; Shannon Russell
EPI_ISL_3407886, EPI_ISL_3525509	Broad Institute Clinical Research Sequencing Platform	Infectious Disease Program, Broad Institute of Harvard and MIT	Adams, G.; B.L.; B.W.; Bauer, M.; Birren; Blumenstiel, B.; Brown, C.; Carter, A.; Chaluvadi, S.; D.J.; DeFelice, M.; DeRuff, K.; Dodge, S.; Gabriel, S.; Gallagher, G.; Gladden-Young, A.; Granger, B.; J.E.; K.J.; Lagerborg, K.; Larkin, K.; Lee, M.; Lemieux; Lennon, N.; Loreth, C.; Madoff, L.; McGovern, S.; Meldrim, J.; Normandin, E.; P.C.; Park; Pearlman, L.; Reilly, S.; Rudy, M.; Sabeti; Siddle; Smole, S.; Tomkins-Tinch, C.; Vicente, G.; and MacInnis
EPI_ISL_3464547	CENTRO PENAL LUIS PAULINO MORA	Inciensa, Instituto Costarricense de Investigación y Enseñanza en Nutrición y Salud	Adriana Godínez; Claudio Soto-Garita; Estela Cordero; Francisco Duarte; Hebleen Porras; Joselyn Prado & Juan Carlos Villalobos Ugalde; José Luis Vargas; Mariela Gutiérrez; Melany Calderón
EPI_ISL_3061861, EPI_ISL_3061862,	CH VALENCIENNES	CHU Lille - Laboratoire de Virologie	AIT YAHYA Emilie; ALIDJINOU Enagnon Kazali; BOCKET Laurence; CREPIN Michel; DEMAY Christophe; ENGELMANN Ilka; GEFFROY Sandrine; GUIGON Aurélie; LAMBERT Valérie; LAZREK Mouna; NOBILLIAUX Florian; PREVOST Brigitte; TCHANTCHOU NJOSSE YANICK; THUILLIER Caroline; TINEZ Claire

EPI_ISL_3061863, EPI_ISL_3061864			
EPI_ISL_3432133	CHTMAD	Instituto Nacional de Saude (INSA)	Borges et al
EPI_ISL_3383406	CHU Purpan - Laboratoire de Virologie - Institut Fédératif de Biologie	CHU Purpan - Laboratoire de Virologie - Institut Fédératif de Biologie	Boyer P.; Carcenac R.; Ferrer V.; Harter A.; Izopet J.; Jeanne N.; Latour J.; Ranger N.; Tremeaux R.
EPI_ISL_3267818	CHUV	Laboratory of genomics and metagenomics	Claire Bertelli; Damien Jacot; Gilbert Greub; Sébastien Aeby; Trestan Pillonel
EPI_ISL_3385855	COLCAN	Instituto Nacional de Salud-Dirección de Investigación en Salud Pública	Carlos Franco-Muñoz; Carmen Osorio; Diana Malo; Diego A. Álvarez-Díaz; Diego Andrés Prada; Gerardo Santamaría; Hector Alejandro Ruiz-Moreno; Jhonnatan Reales-González; Jorge Rivera; Juan Camilo Martinez; Julian Naizaque; Katherine Laiton-Donato; Lisseth Pardo; Magdalena Wiesner; Marcela Mercado-Reyes; Maria T. Herrera-Sepúlveda; Marta Lopez Blanco; Martha Lucia Ospina Martinez; Paola Rojas; Sergio Gomez; Sheryll Corchuelo; Ángela Alarcon Cruz
EPI_ISL_3614327	COVID LAB	Cayman Islands Forensic Science Lab	Brittany Balcewich; Jonathan Smellie; Karla Montes; Tanisha Gilbert
EPI_ISL_3347367	CT Department of Public Health	Grubaugh Lab - Yale School of Public Health	Anderson Brito; Annie Watkins; Chaney Kalinich; Chantal Vogels; Claire Pearson; Isabel Ott; Jessica Rothman; Joseph Fauver; Kendall Billig; Mallery Breban; Mary Petrone; Nathan Grubaugh; Tara Alpert; Tobias Koch; Tu N. Nguyen
EPI_ISL_3355062	CT-Dr. Katherine A. Kelley State Public Health Lab	Centers for Disease Control and Prevention Division of Viral Diseases, Pathogen Discovery	Alex Burgin; Ben Rambo-Martin; Clinton Paden; Dakota Howard; Dave Wentworth; Dhwani Batra; Jasmine Padilla; Justin Lee; Krista Queen; Kristen Knipe; Kristine Lacek; Mark Burroughs; Matthew Schmerer; Meghan Bentz; Mili Sheth; Peter Cook; Sam Shepard; Sarah Nobles; Suxiang Tong; Vivien Dugan; Yvette Unoarumhi
EPI_ISL_3242482, EPI_ISL_3242489, EPI_ISL_3242732	Clinica INDISA	"Facultad de Ciencias de la Vida, UNAB"	"Claudio Meneses; Ariel Orellana"; Claudio Olmos; Daniel Leon; Dayan Sanhueza; Eduardo Castro; Gonzalo Campaña; Macarena Bastias; Paola Pidal; Ricardo Yusta; Sebastian Wolter; Susana Saez; Víctor Monreal; Waldo Diaz
EPI_ISL_3635412	DYOMEDEA-LABORATOIRE DE LA SAUVEGARDE	CNR Virus des Infections Respiratoires - France SUD	Antonin Bal; Bruno Lina; Gregory Destras; Gwendolyne Burfin; Hadrien Regue; Laurence Josset; Martine Valette; Quentin Semanas
EPI_ISL_3041379, EPI_ISL_3340126	Department of Bacteria, Parasites and Fungi, Statens Serum Institut, Copenhagen, Denmark	Statens Serum Institut Bioinformatics and Microbial Genomics	Danish Covid-19 Genome Consortium
EPI_ISL_3266210	Department of Public Health Sibiu	National Institute of Infectious Diseases-Prof. Dr. Matei Bals Molecular Diagnostics Laboratory	Corina Casangiu; Dan Otelea; Leontina Banica; Marius Surleac; Ovidiu Vlaicu; Petre Milu; Robert Hohan; Simona Paraschiv
EPI_ISL_3152109, EPI_ISL_3298382, EPI_ISL_3298383	Dipartimento di Medicina di Laboratorio, Azienda sanitaria universitaria Friuli Centrale (ASU FC)	Dipartimento di Medicina di Laboratorio, Azienda sanitaria universitaria Friuli Centrale (ASU FC)	Catia Mio; Chiara Dal Secco; Corrado Pipan; Francesco Curcio; Stefania Marzinotto
EPI_ISL_3052919	Dirección regional de salud del Callao (DIRESA-CALLAO)	Centro de Investigaciones Tecnológicas, Biomédicas y Medioambientales (CITBM)	B; Huaman; J. Alarcon; M. Cuellar; M. Ramirez; M. Sovero
EPI_ISL_3024839	Division of Emerging Infectious Diseases, Bureau of Infectious Diseases Diagnosis Control, Korea Disease Control and Prevention Agency	Division of Emerging Infectious Diseases, Bureau of Infectious Diseases Diagnosis Control, Korea Disease Control and Prevention Agency	Ae Kyung Park; Chae Young Lee; Eun-Jin Kim; Heui Man Kim; Il-Hwan Kim; Jeong-Ah Kim; Jeong-Min Kim
EPI_ISL_3389223, EPI_ISL_3578180, EPI_ISL_3578239, EPI_ISL_3578242	Dr. Risch Ostschweiz AG	Microbiology, Dr. Risch	Dominique Fabien Hilti; Faina Wehrli; Lorenz Risch; Martin Risch; Nadia Wohlwend; Sinem Kas; Thomas Bodmer
EPI_ISL_3148575	Dutch COVID-19 response team	Erasmus Medical Center	Anne van der Linden; Annemiek van der Eijk; Bas Oude Munnink; Corine GeurtsvanKessel; David Nieuwenhuijse; Emmanuelle Munger; Irina Chestakova; Marion Koopmans; Marjan Boter; Reina Sikkema; Richard Molenkamp; on behalf of the Dutch national COVID-19 response team.
EPI_ISL_3056750, EPI_ISL_3056849, EPI_ISL_3057362, EPI_ISL_3057834, EPI_ISL_3057842, EPI_ISL_3057849, EPI_ISL_3057855, EPI_ISL_3057864, EPI_ISL_3057871, EPI_ISL_3058099, EPI_ISL_3136585, EPI_ISL_3136617, EPI_ISL_3138350, EPI_ISL_3138360, EPI_ISL_3138368, EPI_ISL_3138370, EPI_ISL_3138405, EPI_ISL_3138419, EPI_ISL_3138424, EPI_ISL_3138429, EPI_ISL_3138430, EPI_ISL_3266858, EPI_ISL_3266859, EPI_ISL_3388928, EPI_ISL_3389111			
see above	Dutch COVID-19 response team	National Institute for Public Health and the Environment (RIVM)	Adam Meijer; AnneMarie van den Brandt; Annelies Kroneman; Bas van der Veer; Chantal Reusken; Dennis Schmitz; Dirk Eggink; Euníce Then; Florian Zwagemaker; Harry Vennema; Jeroen Cremer; Karim Hajji; Kim Freriks; Lisa Wijsman; Lynn Aarts; Melissa van Tuil; Ryanne Jaarsma; Sanne Bos; Sharon van den Brink; Stijn van Rossum; on behalf of the national COVID-19 response team
EPI_ISL_3491507	FL Bureau of Public Health Laboratories-Tampa	Centers for Disease Control and Prevention Division of Viral Diseases, Pathogen Discovery	Alex Burgin; Ben Rambo-Martin; Clinton Paden; Dakota Howard; Dave Wentworth; Dhwani Batra; Jasmine Padilla; Justin Lee; Krista Queen; Kristen Knipe; Kristine Lacek; Mark Burroughs; Matthew Schmerer; Meghan Bentz; Mili Sheth; Peter Cook; Sam Shepard; Sarah Nobles; Suxiang Tong; Vivien Dugan; Yvette Unoarumhi
EPI_ISL_3398814, EPI_ISL_3398815, EPI_ISL_3398819	FUNDACION VALLE DE LILI	Instituto Nacional de Salud	Carlos Franco-Muñoz; Carmen Osorio; Diana Malo; Diego A. Álvarez-Díaz; Diego Andrés Prada; Gerardo Santamaría; Hector Alejandro Ruiz-Moreno; Jhonnatan Reales-González; Jorge Rivera; Juan Camilo Martinez; Julian Naizaque; Katherine Laiton-Donato; Lisseth Pardo; Magdalena Wiesner; Marcela Mercado-Reyes; Maria T.Herrera-Sepúlveda; Marta Lopez Blanco; Martha Lucia Ospina Martinez; Paola Rojas; Sergio Gomez; Sheryll Corchuelo; Ángela Alarcon Cruz
EPI_ISL_3369934	FUNDACIÓN HOSPITAL SAN PEDRO	Corporacion CorpoGen-Universidad de los Andes-Universidad Central	Christian Romero; Jorge Duitama; Juan Manuel Anzola; Laura González; Maryam Chaib De Mares; María Mercedes Zambrano; Nelly Díaz; Patricia Del Portillo; Silvia Restrepo
EPI_ISL_3020727, EPI_ISL_3020836, EPI_ISL_3020842	Fimlab Laboratoriot Oy Tampere	Expert Microbiology, National Institute for Health and Welfare	Carita Savolainen-Kopra; Erika Lindh; Haider al-Hello; Jani Halkilahti; Kirsi Liitsola; Niina Ikonen; Olli Vapalahti; Pekka Ellonen; Phuoc Truong; Päivi Laurila; Ravi Kant; Sari Hannula; Soile Blomqvist; Teemu Smura
EPI_ISL_3304939, EPI_ISL_3305727, EPI_ISL_3305743, EPI_ISL_3460963, EPI_ISL_3609634	Fulgent Genetics	Centers for Disease Control and Prevention Division of Viral Diseases, Pathogen Discovery	Adrian Paskey; Becky Tsai; Benafsh Sapra; Benjamin Rambo-Martin; Christopher Gulvick; Clinton Paden; Clinton R. Paden; Dakota Howard; Darlene Wagner; Dhwani Batra; Doreen Ng; Duncan MacCannell; Harry Gao; James Xie; Jason Caravas; John Gao; Joseph Fierro; Kara Moser; Matthew Schmerer; Mickey Li; Peter Cook; Peter W. Cook; Scott Sammons; Shatavia Morrison; Yan Meng; Yvette Unoarumhi
EPI_ISL_3491809	Gencore - Universidad de los Andes	Gencore - Universidad de los Andes	Catherine Jaller; Cristian Barrera; David González; Luisa Sacristan; Marcela Guevara; Silvia Restrepo
EPI_ISL_3185499, EPI_ISL_3185623, EPI_ISL_3185661, EPI_ISL_3185751, EPI_ISL_3185773, EPI_ISL_3185774, EPI_ISL_3185775, EPI_ISL_3185802			
see above	Genetica Molecular and Subdepartamento de Virologia ISP Chile	Instituto de Salud Publica de Chile	Andres Castillo; Barbara Parra; Constanza Campano; Gisselle Barra; Javier Tognarelli; Jorge Fernandez; Karen Orostica; Loredana Arata; Patricia Bustos; Rodrigo Fasce; Soledad Ulloa
EPI_ISL_3464541	HOSPITAL CIUDAD NEILY	Inciensa, Instituto Costarricense de Investigación y Enseñanza en Nutrición y Salud	Adriana Godínez; Claudio Soto-Garita; Estela Cordero; Francisco Duarte; Hebleen Porras; Joselyn Prado & Susana Mata Guerrero; José Luis Vargas; Mariela Gutiérrez; Melany Calderón
EPI_ISL_3274351	HOSPITAL DR. CARLOS LUIS VALVERDE VEGA	Inciensa, Instituto Costarricense de Investigación y Enseñanza en Nutrición y Salud	Adriana Godínez; Caterina Guzmán; Claudio Soto-Garita; Estela Cordero; Francisco Duarte; Hebleen Porras; José Luis Vargas; Mariela Gutiérrez Joselyn Prado; Melany Calderón; Nazareth Ruiz & Charbel Vargas
EPI_ISL_3274363	HOSPITAL DR. CARLOS LUIS VALVERDE VEGA [JESUS MARIA/SAN RAMON/ALAJUELA]	Inciensa, Instituto Costarricense de Investigación y Enseñanza en Nutrición y Salud	Adriana Godínez; Caterina Guzmán; Claudio Soto-Garita; Estela Cordero; Francisco Duarte; Hebleen Porras; José Luis Vargas; Mariela Gutiérrez Joselyn Prado; Melany Calderón; Nazareth Ruiz & Charbel Vargas
EPI_ISL_3639001	HOSPITAL DR. FERNANDO ESCALANTE PRADILLA	Inciensa, Instituto Costarricense de Investigación y Enseñanza en Nutrición y Salud	Adriana Godínez; Claudio Soto-Garita; Estela Cordero; Francisco Duarte; Hebleen Porras; Joselyn Prado & María Fernanda Matamoros; José Luis Vargas; Mariela Gutiérrez; Melany Calderón
EPI_ISL_3274355, EPI_ISL_3298353	HOSPITAL METROPOLITANO	Inciensa, Instituto Costarricense de Investigación y Enseñanza en Nutrición y Salud	Adriana Godínez; Caterina Guzmán; Claudio Soto-Garita; Estela Cordero; Francisco Duarte; Hebleen Porras; Joselyn Prado & Margarita Lee-Lui; José Luis Vargas; Mariela Gutiérrez; Mariela Gutiérrez Joselyn Prado; Melany Calderón; Nazareth Ruiz & Margarita Lee
EPI_ISL_3274358, EPI_ISL_3274359, EPI_ISL_3298342	HOSPITAL SAN FRANCISCO DE ASIS	Inciensa, Instituto Costarricense de Investigación y Enseñanza en Nutrición y Salud	Adriana Godínez; Caterina Guzmán; Claudio Soto-Garita; Estela Cordero; Francisco Duarte; Hebleen Porras; Joselyn Prado & Adrián Fallas; José Luis Vargas; Mariela Gutiérrez; Mariela Gutiérrez Joselyn Prado; Melany Calderón; Nazareth Ruiz & Adrian Fallas Mora
EPI_ISL_3502573	HOSPITAL UNIVERSITARIO 12 DE OCTUBRE	HOSPITAL UNIVERSITARIO 12 DE OCTUBRE	Carmen Martín-Higuera; Esther Viedma; Irene Múñoz-Gallego; M.ª Dolores Folgueira; Mar Aguilera; Noelia Moral; Rafael Delgado; Sagrario Zurita
EPI_ISL_3459404, EPI_ISL_3462744	HOSPITAL UNIVERSITARIO HERNANDO MONCALEANO PERDOMO (HUHMP	Instituto Nacional de Salud	Carlos Franco-Muñoz; Carmen Osorio; Diana Malo; Diego A. Álvarez-Díaz; Diego Andrés Prada; Gerardo Santamaría; Hector Alejandro Ruiz-Moreno; Jhonnatan Reales-González; Jorge Rivera; Juan Camilo Martinez; Julian Naizaque; Katherine Laiton-Donato; Lisseth Pardo; Magdalena Wiesner; Marcela Mercado-Reyes; Maria T. Herrera-Sepúlveda; Marta Lopez Blanco; Martha Lucia Ospina Martinez; Paola Rojas; Sergio Gomez; Sheryll Corchuelo; Ángela Alarcon Cruz

EPI_ISL_3276551	HPTU	Universidad Nacional de Colombia - Laboratorio Genómico One Health	Andres F. Cardona-Rios; Carlos Franco-Muñoz; Carolina Muñoz-Arango; Celeny Ortiz; Daniel O. Maldonado-Perez; Diego A. Álvarez-Díaz; Hector Alejandro Ruiz-Moreno; Idabely Betancur Ortiz; Jorge E. Osorio; Juan P. Hernandez-Ortiz; Karl A Ciuoderis; Katherine Laiton-Donato; Laura Silvana Perez; Lina M. Hurtado; Marcela Mercado-Reyes; Maria Angélica Maya; Maria Stella López; Rita Almanza Payares; Sandra Ines Cano; Simón Villegas Velásquez
EPI_ISL_3320721, EPI_ISL_3320726	Hemato Oncologos	Instituto Nacional de Salud	Carlos Franco-Muñoz; Carmen Osorio; Diana Malo; Diego A. Álvarez-Díaz; Diego Andrés Prada; Gerardo Santamaría; Hector Alejandro Ruiz-Moreno; Jhonnatan Reales-González; Jorge Rivera; Juan Camilo Martinez; Julian Naizaque; Katherine Laiton-Donato; Lisseth Pardo; Magdalena Wiesner; Marcela Mercado-Reyes; Maria T. Herrera-Sepúlveda; Marta Lopez Blanco; Martha Lucia Ospina Martinez; Paola Rojas; Sergio Gomez; Sheryll Corchuelo; Ángela Alarcon Cruz
EPI_ISL_3061858, EPI_ISL_3061859	Hospital	National Reference Center for Viruses of Respiratory Infections, Institut Pasteur, Paris	Alexandra Ducancelle; Angela Brisebarre; Camille Capel; Christophe Malabat; Corinne Maufrais; Etienne Simon-Lorière; Frédéric Lemoine; Hub de Bioinformatique et Biostatistique; Louise Lefrançois; Marion Barbet; Maud Vanpeene; Méline Bizard; Sylvie Behillil; Sylvie Van der Werf; Vincent Enouf
EPI_ISL_3050294	Hospital Clínico Universitario Virgen de la Arrixaca	Hospital Clínico Universitario Virgen de la Arrixaca	Laura Moreno and Luis Gil-Gallardo; Marina Simón
EPI_ISL_3188867, EPI_ISL_3505956	Hospital General Universitario Gregorio Marañón	Hospital General Universitario Gregorio Marañón	Cristina Rodriguez-Grande; Darío García de Viedma; Julia Suárez; Laura Pérez-Lago; Marta Herranz Martin; Patricia Muñoz; Pedro Sola Campoy; Pilar Catalán; Sergio Buenestado Serrano; Victor Manuel de la Cueva
EPI_ISL_3216188, EPI_ISL_3216194, EPI_ISL_3216216	Hospital General Universitario de Alicante - Instituto de Investigación Sanitaria y Biomédica de Alicante	SeqCOVID-SPAIN consortium/IBV(CSIC)	Carmen Molina Pardines and SeqCOVID-SPAIN consortium; Maripaz Ventero Martín
EPI_ISL_3161803	Hospital Universitari Vall d'Hebron - Vall d'Hebron Institut de Recerca	Hospital Universitari Vall d'Hebron - Vall d'Hebron Institut de Recerca	Alejandra González-Sánchez; Andrés Antón; Ariadna Rando; Carla Castillo; Cristina Andrés; Damir Garcia-Cehic; Josep Quer; Juliana Esperalba; Karen García; Maria Carmen Martin; Maria Gema Codina; Maria Piñana; Rodrigo Vásquez; Tomàs Pumarola
EPI_ISL_3276628, EPI_ISL_3276629	ICMT-Apartado	Universidad Nacional de Colombia - Laboratorio Genómico One Health	Andres F. Cardona-Rios; Carlos Franco-Muñoz; Carolina Muñoz-Arango; Celeny Ortiz; Daniel O. Maldonado-Perez; Diego A. Álvarez-Díaz; Hector Alejandro Ruiz-Moreno; Idabely Betancur Ortiz; Jorge E. Osorio; Juan P. Hernandez-Ortiz; Karl A Ciuoderis; Katherine Laiton-Donato; Laura Silvana Perez; Lina M. Hurtado; Marcela Mercado-Reyes; Maria Angélica Maya; Maria Stella López; Rita Almanza Payares; Sandra Ines Cano; Simón Villegas Velásquez
EPI_ISL_3391981	IMAT S.A.S.	Instituto Nacional de Salud-Dirección de Investigación en Salud Pública	Carlos Franco-Muñoz; Carmen Osorio; Diana Malo; Diego A. Álvarez-Díaz; Diego Andrés Prada; Gerardo Santamaría; Hector Alejandro Ruiz-Moreno; Jhonnatan Reales-González; Jorge Rivera; Juan Camilo Martinez; Julian Naizaque; Katherine Laiton-Donato; Lisseth Pardo; Magdalena Wiesner; Marcela Mercado-Reyes; Maria T. Herrera-Sepúlveda; Marta Lopez Blanco; Martha Lucia Ospina Martinez; Paola Rojas; Sergio Gomez; Sheryll Corchuelo; Ángela Alarcon Cruz
EPI_ISL_3104821, EPI_ISL_3104824, EPI_ISL_3579331	INSIDE DIAGNÓSTICOS	Instituto Butantan	Antonio Jorge Martins; Claudia Renata dos Santos Barros; David Schlesinger; Debora Botequio Moretti; Dimas Tadeu Covas; Elaine Cristina Marqueze; Elaine Vieira Santos; Evandra Strazza Rodrigues; Heidge Fukumasu; Jayme Augusto de Souza-Neto; José Salvatore Leister Patané; Luiz Alcantara; Luiz Lehmann Coutinho; Maria Carolina Elias; Maurício Lacerda Nogueira; Rafael dos Santos Bezerra; Raul Machado Neto; Rejane Maria Tommasini Grotto; Ricardo Haddad; Sandra Coccuzzo Sampaio Vessoni; Simone Kashima; Svetoslav Nanev Slavov; Vincent Louis Viala
EPI_ISL_3031564	INSIDE DIAGNÓSTICOS - UPA TATUAPÉ	Instituto Butantan	Antonio Jorge Martins; Claudia Renata dos Santos Barros; David Schlesinger; Debora Botequio Moretti; Dimas Tadeu Covas; Elaine Cristina Marqueze; Elaine Vieira Santos; Evandra Strazza Rodrigues; Heidge Fukumasu; Jayme Augusto de Souza-Neto; José Salvatore Leister Patané; Luiz Alcantara; Luiz Lehmann Coutinho; Maria Carolina Elias; Maurício Lacerda Nogueira; Rafael dos Santos Bezerra; Raul Machado Neto; Rejane Maria Tommasini Grotto; Ricardo Haddad; Sandra Coccuzzo Sampaio Vessoni; Simone Kashima; Svetoslav Nanev Slavov; Vincent Louis Viala
EPI_ISL_3133197, EPI_ISL_3274538	INSPI-CRN DE INFLUENZA Y OTROS VIRUS RESPIRATORIOS	NIC-INSPI	Alfredo Bruno; Daniel Ramos; Domenica de Mora.; Jimmy Garcés; Johanna Laines; Lizbeth Patiño; Manuel Gonzalez; Maria Angelica Becerra; Maritza Olmedo; Mayra Wilca; Michelle Páez
EPI_ISL_3298687, EPI_ISL_3298689	IVIC	Laboratorio de Virología Molecular	Carmen L Loureiro; CoViMol Group; Domingo J Garzaro; Esmeralda Vizzi; Flor H Pujol; Héctor R Rangel; José Luis Zambrano; Lieska Rodríguez; Mariana Hidalgo; Pierina D´Angelo; Rossana C Jaspe; Víctor Alarcón; Yoneira Sulbaran; Zoila Moros
EPI_ISL_3610849	Infinity Biologix	Centers for Disease Control and Prevention Division of Viral Diseases, Pathogen Discovery	Adrian Paskey; Benjamin Rambo-Martin; Chirayu Goswami; Christian Bixby; Christopher Gulvick; Clinton Paden; Dakota Howard; Darlene Wagner; Dhwani Batra; Duncan MacCannell; Jason Caravas; Jonathan Schultz; Kara Moser; Matthew Schmerer; Peter Cook; Robin Grimwood; Russ Hager; Scott Sammons; Shatavia Morrison; Yihe Wang; Yvette Unoarumhi
EPI_ISL_3132358	Institute of Microbiology, Universidad San Francisco de Quito	Institute of Microbiology, Universidad San Francisco de Quito	Alexandra Gonzalez; Belén Prado-Vivar; Bernardo Gutiérrez; Betty Angulo; Erika Muñoz; Fernanda Zurita; Francisco Guerra; Gabriel Trueba; Hugo Vergara; Jeaninna Peña; Juan José Guadalupe; Liu Yuqian; Luis Fuenmayor; Luz-Angelica Castillo; Marco Viteri Yanez; Michelle Grunauer; Michelle Jacome; Monica Becerra-Wong; Nancy Flores Lastra; Natalia Parra; Patricio Rojas-Silva; Paúl Cárdenas; Ronny Javier Pibaque; Stephanie Espín-Arroba; Sully Márquez; Valeria Armijos; Verónica Barragán
EPI_ISL_3316300	Instituto Adolfo Lutz - Regional de Rio Claro	Instituto Adolfo Lutz, Interdiciplinary Procedures Center, Strategic Laboratory	Caio Vinicius Dias Lopes; Claudia Regina Gonçalves; Claudio Tavares Sacchi; Karoline Rodrigues Campos; Leonardo Tadeu de Araujo; Marlon Benedito Nascomento Santos
EPI_ISL_3507367, EPI_ISL_3507369, EPI_ISL_3507370	Instituto Venezolano de Investigaciones Científicas	Laboratorio de Virología Molecular	Carmen L Loureiro; CoViVen Group; Domingo J Garzaro; Flor H Pujol; Héctor R Rangel; José Luis Zambrano; Lieska Rodríguez; Mariana Hidalgo; Pierina D´Angelo; Rossana C Jaspe; Víctor Alarcón; Yoneira Sulbaran; Zoila Moros
EPI_ISL_3188597, EPI_ISL_3188598, EPI_ISL_3188599, EPI_ISL_3188600, EPI_ISL_3188608, EPI_ISL_3188609, EPI_ISL_3236440, EPI_ISL_3236441, EPI_ISL_3236444, EPI_ISL_3236445, EPI_ISL_3236446, EPI_ISL_3236448, EPI_ISL_3236450, EPI_ISL_3236451			
see above	Instituto de Medicina Tropical & Salud Global Universidad Iberoamericana	Grubaugh Lab - Yale School of Public Health	Alejandaro Vallejo Degaudenzi; Anderson Brito; Annie Watkins; Chaney Kalinich; Chantal Vogels; Elisa Contreras; Esperanza Mendoza; Isabel Ott; Jessica Rothman; Joseph Fauver; Kendall Billig; Mallery Breban; Mary Petrone; Nathan Grubaugh; Robert Paulino-Ramirez; Tara Alpert; Tobias Koch; Victor Virgilio Calderon
EPI_ISL_3071067	Istituto Ortopedico Galeazzi	Laboratory of Clinical Microbiology, Virology and Bioemergencies, ASST Fatebenefratelli Sacco - Sacco Hospital	Valeria Micheli
EPI_ISL_3373121	Johns Hopkins Hospital Department of Pathology	Johns Hopkins Hospital Department of Pathology	C. Paul Morris; Chun Huai Luo; David Gaston; Heba H. Mostafa; Julie M. Norton; Matthew Schwartz
EPI_ISL_3085775	Karolinska University Hospital Solna	Karolinska University Hospital	Annelie Bjerkner; Isak Sylvin; Jan Albert; Karolina Ininbergs; Lina Guerra Blomqvist; Lynda Eneh; Martin Ekman; Martina Wahlund; Robert Dyrdak; Sandra Broddesson; Tanja Normark; Tobias Allander; Valtteri Wirta; Zhibing Yun
EPI_ISL_3462746	LABORATORIO CLINICO BIO-TEST	Instituto Nacional de Salud	Carlos Franco-Muñoz; Carmen Osorio; Diana Malo; Diego A. Álvarez-Díaz; Diego Andrés Prada; Gerardo Santamaría; Hector Alejandro Ruiz-Moreno; Jhonnatan Reales-González; Jorge Rivera; Juan Camilo Martinez; Julian Naizaque; Katherine Laiton-Donato; Lisseth Pardo; Magdalena Wiesner; Marcela Mercado-Reyes; Maria T. Herrera-Sepúlveda; Marta Lopez Blanco; Martha Lucia Ospina Martinez; Paola Rojas; Sergio Gomez; Sheryll Corchuelo; Ángela Alarcon Cruz
EPI_ISL_3385791, EPI_ISL_3385842	LABORATORIO CLINICO CRISTIAN GRAM IPS SAS	Instituto Nacional de Salud-Dirección de Investigación en Salud Pública	Carlos Franco-Muñoz; Carmen Osorio; Diana Malo; Diego A. Álvarez-Díaz; Diego Andrés Prada; Gerardo Santamaría; Hector Alejandro Ruiz-Moreno; Jhonnatan Reales-González; Jorge Rivera; Juan Camilo Martinez; Julian Naizaque; Katherine Laiton-Donato; Lisseth Pardo; Magdalena Wiesner; Marcela Mercado-Reyes; Maria T. Herrera-Sepúlveda; Marta Lopez Blanco; Martha Lucia Ospina Martinez; Paola Rojas; Sergio Gomez; Sheryll Corchuelo; Ángela Alarcon Cruz
EPI_ISL_3274357	LABORATORIO CLINICO LABIN	Inciensa, Instituto Costarricense de Investigación y Enseñanza en Nutrición y Salud	Adriana Godínez; Caterina Guzmán; Claudio Soto-Garita; Estela Cordero; Francisco Duarte; Hebleen Porras; José Luis Vargas; Mariela Gutiérrez Joselyn Prado; Melany Calderón; Nazareth Ruiz & Pei Chan
EPI_ISL_3274352	LABORATORIO CLINICO UNIVERSIDAD DE COSTA RICA (NUCLEO)	Inciensa, Instituto Costarricense de Investigación y Enseñanza en Nutrición y Salud	Adriana Godínez; Caterina Guzmán; Claudio Soto-Garita; Estela Cordero; Francisco Duarte; Hebleen Porras; José Luis Vargas; Mariela Gutiérrez Joselyn Prado; Melany Calderón; Nazareth Ruiz & Dimelsa Suarez
EPI_ISL_3246157	LATE - Laboratório de Técnicas Especiais - Hospital Israelita Albert Einstein	LATE - Laboratório de Técnicas Especiais - Hospital Israelita Albert Einstein	Alexandre Hideaki Takara; Ana Paula Moreira Salles; Anelisie da Silva Santos; Deyvid Amgarten; Erick Gustavo Dorlass; Fernanda de Mello Malta; João Renato Rebello Pinho; Marcio Anunciacao Menezes; Pedro Henrique Sebe Rodrigues; Raquel Riyuzo
EPI_ISL_3459398	LDSP CAQUETA	Instituto Nacional de Salud	Carlos Franco-Muñoz; Carmen Osorio; Diana Malo; Diego A. Álvarez-Díaz; Diego Andrés Prada; Gerardo Santamaría; Hector Alejandro Ruiz-Moreno; Jhonnatan Reales-González; Jorge Rivera; Juan Camilo Martinez; Julian Naizaque; Katherine Laiton-Donato; Lisseth Pardo; Magdalena Wiesner; Marcela Mercado-Reyes; Maria T. Herrera-Sepúlveda; Marta Lopez Blanco; Martha Lucia Ospina Martinez; Paola Rojas; Sergio Gomez; Sheryll Corchuelo; Ángela Alarcon Cruz
EPI_ISL_3459391	LDSP CUNDINAMARCA	Instituto Nacional de Salud	Carlos Franco-Muñoz; Carmen Osorio; Diana Malo; Diego A. Álvarez-Díaz; Diego Andrés Prada; Gerardo Santamaría; Hector Alejandro Ruiz-Moreno; Jhonnatan Reales-González; Jorge Rivera; Juan Camilo Martinez; Julian Naizaque; Katherine Laiton-Donato; Lisseth Pardo; Magdalena Wiesner; Marcela Mercado-Reyes; Maria T. Herrera-Sepúlveda; Marta Lopez Blanco; Martha Lucia Ospina Martinez; Paola Rojas; Sergio Gomez; Sheryll Corchuelo; Ángela Alarcon Cruz
EPI_ISL_3127426	Laboratoires d'analyses medicales - Ketterthill	Laboratoire national de sante, Microbiology, Microbial Genomics Platform	Anke Wienecke-Baldacchino; Caroline Scheiber; Catherine Ragimbeau; Elodie Solarino; Fatu Djabi; Jessica Tapp; Lise Pignon; Raoul Salmon; Serge Vedy; Tamir Abdelrahman; Virginie Jover
EPI_ISL_3045422	Laboratorio Nacional de Salud Pública Dr. Defilló - LNSPDD	Laboratory of Respiratory Viruses and Measles, Oswaldo Cruz Institute, FIOCRUZ	Alice Sampaio Rocha; Ana Carolina Mendonca; Anna Carolina Paixao; Elisa Cavalcante Pereira; Fernando Motta; Grey Benoit Vasquez; Isaac Miguel Sanchez; Ivonne Imbert; Lucia de la Cruz; Luciana Appolinario; Marilda Siqueira on behalf of the Fiocruz COVID-19 Genomic Surveillance Network; Nurys de Castro; Paola Resende; Renata Serrano Lopes; Ronald Skewes; Taina Venas
EPI_ISL_3320742, EPI_ISL_3320747	Laboratorio Olimpus	Instituto Nacional de Salud	Carlos Franco-Muñoz; Carmen Osorio; Diana Malo; Diego A. Álvarez-Díaz; Diego Andrés Prada; Gerardo Santamaría; Hector Alejandro Ruiz-Moreno; Jhonnatan Reales-González; Jorge Rivera; Juan Camilo Martinez; Julian Naizaque; Katherine Laiton-Donato; Lisseth Pardo; Magdalena Wiesner; Marcela Mercado-Reyes; Maria T. Herrera-Sepúlveda; Marta Lopez Blanco; Martha Lucia Ospina Martinez; Paola Rojas; Sergio Gomez; Sheryll Corchuelo; Ángela Alarcon Cruz
EPI_ISL_3376685, EPI_ISL_3376686, EPI_ISL_3376687, EPI_ISL_3376690, EPI_ISL_3376697, EPI_ISL_3376703, EPI_ISL_3376707, EPI_ISL_3376708, EPI_ISL_3376711, EPI_ISL_3376712, EPI_ISL_3376714, EPI_ISL_3376717, EPI_ISL_3376718, EPI_ISL_3376723, EPI_ISL_3376724, EPI_ISL_3376727			
see above	Laboratorio de Biología Molecular - Universidad del Magdalena	Centro de Genética y Biología Molecular - Universidad del Magdalena	Andrea M. Ramírez Hernandez; Angel Oviedo Marquez; Daniel Bautista; Lyda R. Castro; Maria Teresa Mojica-Ortiz
EPI_ISL_3321115, EPI_ISL_3321116, EPI_ISL_3321309, EPI_ISL_3535716, EPI_ISL_3535748	Laboratorio de Infectologia y Virologia Molecular	Laboratory of Molecular Virology, School of Medicine, Pontificia Universidad Catolica de Chile	Alejandro Bhrun; Ana Maria Contreras; Andres E. Munoz-Marcos; Carlos Palma; Catalina Pardo-Roa; Constanza Maldonado; Constanza Martinez-Valdevenito; Eileen Serrano; Erick Salinas; Estefany Poblete; Francisco Melo; Jennifer Angulo; Jorge Levican; Leonardo I. Almonacid; M. Belen Leyton; Magdalena Vera; Marcela Ferres; Maria Jose Avendano; Rafael A. Medina; Tamara Garcia-Salum
EPI_ISL_3654103, EPI_ISL_3654104, EPI_ISL_3654105, EPI_ISL_3654106, EPI_ISL_3654107, EPI_ISL_3655700	Laboratorio de Referencia Nacional de Virus Respiratorios. Centro Nacional de Salud Publica. Instituto Nacional de Salud Peru.	Laboratorio de Referencia Nacional de Virus Respiratorios. Centro Nacional de Salud Publica. Instituto Nacional de Salud Peru.	Carlos Padilla Rojas; Henri Bailon Calderon; Iris Silva Molina; Joseph Huayra Niquen; Lely Solari Zerpa; Luis Barcena Flores; Marco Galarza Perez; Nancy Rojas Serrano; Nieves Sevilla Castañeda; Omar Caceres Rey; Orson Mestanza Millones; Princesa Medrano Alhuay; Priscila Lope Pari; Sandra Morales Ruiz; Sara Gordillo Vilchez; Steve Acedo Lazo; Veronica Hurtado Vela; Victor Jimenez Vasquez; Wendy Lizarraga Olivares

EPI_ISL_3055549	Laboratorio de salud publica Puebla	LABOPAT	Cynthia Penaloza; Luis Mendoza; Silvia Montilla
EPI_ISL_3430864, EPI_ISL_3431052, EPI_ISL_3510253, EPI_ISL_3512112, EPI_ISL_3514445, EPI_ISL_3518226, EPI_ISL_3605427			
see above	Laboratory Corporation of America	Centers for Disease Control and Prevention Division of Viral Diseases, Pathogen Discovery	Adrian Paskey; Amanda Douglas; Amanda Suchanek; Andrea Throop; Ayla Burns; Benjamin Rambo-Martin; Bobbi Croy; Brian Krueger; Brian Norvell; Christopher Gulvick; Christos Petropoulos; Clinton Paden; Clinton R. Paden; Craig Lukasik; Dakota Howard; Darlene Wagner; Debbie Boles; Dhwani Batra; Duncan MacCannell; Eyad Almasri; Goran Stevovic; Howard Engler; Hrushikesh Deshmukh; Jake Humphrey; Jana Schroth; Jason Caravas; Joe Voshell; John Pruitt; Jonathan Meltzer; Jonathan Williams; Kara Moser; Kimberly Wagner; Lax Iyer; Lisa Pfefferle; Lyndon Tilson; Manoj Jain; Marcia Eisenberg; Mary Ann Cristobal; Mary Cristobal; Mary Williamson; Matthew Robinson; Matthew Schmerer; Michael Levandoski; Mike Sapeta; Mindy Nye; Minoo Agarwal; Mohan Kolli; Nuthawin Charoensri; Oren Cohen; Peter Cook; Peter W. Cook; Prashant Gupta; Qian Zeng; Rama Ghatti; Scott Parker; Scott Ryan; Scott Sammons; Shatavia Morrison; Stanley Letovsky; Steven Ragan; Suresh Babu Selvaraju; Suresh Selvaraju; Susan Countryman; Susan Hicks; Suzanne Dale; Thomas Urban; Tim Kuphal; Tricia Zwiefelhofer; Vincent Drouillon; Yvette Unoarumhi
EPI_ISL_3583516	Lighthouse Lab in Glasgow	Wellcome Sanger Institute for the COVID-19 Genomics UK (COG-UK) Consortium	Anna Dominiczak and Alex Alderton; Carol Clugston; Cordelia Langford; David Gray; David K. Jackson; Dominic Kwiatkowski; Ewan Harrison; Harper VanSteenhouse; Ian Johnston; Jeffrey Barrett; John Sillitoe on behalf of the Wellcome Sanger Institute COVID-19 Surveillance Team; Roberto Amato; Sonia Goncalves; Yumi Kasai
EPI_ISL_3042294, EPI_ISL_3095213, EPI_ISL_3095666, EPI_ISL_3096103, EPI_ISL_3108676, EPI_ISL_3171897	Lighthouse Lab in Milton Keynes	Wellcome Sanger Institute for the COVID-19 Genomics UK (COG-UK) Consortium	Cordelia Langford; David K. Jackson; Dominic Kwiatkowski; Ewan Harrison; Ian Johnston; Jeffrey Barrett; John Sillitoe on behalf of the Wellcome Sanger Institute COVID-19 Surveillance Team; Roberto Amato; Sonia Goncalves; The Lighthouse Lab in Milton Keynes and Alex Alderton
EPI_ISL_3152228	MD PHL	Maryland Department of Health Laboratories Administration	Ami Patel; Eric N. Keller; Jillian Loomis; Kwang Low; Terence L. Moore; and Robert Myers
EPI_ISL_3472780	MIRIALIS CLUSES BECHET	CNR Virus des Infections Respiratoires - France SUD	Antonin Bal; Bruno Lina; Gregory Destras; Gwendolyne Burfin; Hadrien Regue; Laurence Josset; Martine Valette; Quentin Semanas
EPI_ISL_3085879, EPI_ISL_3086039	Maine Health and Environmental Testing Laboratory	Tewhey Lab, The Jackson Laboratory	Barter, M.; Dewey, H.; H. and Tewhey, R.; Iosue, F.; Lynch, R.; Matluk, N.; Munger
EPI_ISL_3603488	Mako Medical	Centers for Disease Control and Prevention Division of Viral Diseases, Pathogen Discovery	Adrian Paskey; Benjamin Rambo-Martin; Christopher Gulvick; Clinton Paden; Dakota Howard; Darlene Wagner; Dhwani Batra; Duncan MacCannell; Jason Caravas; Kara Moser; Lauren Moon; Matthew Schmerer; Matthew Tugwell; Peter Cook; Scott Sammons; Shatavia Morrison; Yvette Unoarumhi
EPI_ISL_3062529, EPI_ISL_3549736	Maryland Genomics, Institute for Genome Sciences, University of Maryland School of Medicine	Maryland Genomics, Institute for Genome Sciences, University of Maryland School of Medicine	Claire M; Fraser; Hazen; Holly; Humphrys; Ivette; Jacques; Jonathan; Kranthi; Lim; Lisa D; Luke J; Mike; Ott; Ravel; Roussey; Sadzewicz; Sandra; Santana-Cruz; Tallon; Tracy; Vavikolanu
EPI_ISL_3332862	Medyczne Laboratoria Diagnostyczne INVICTA	Wojewodzka Stacja Sanitarno-Epidemiologiczna w Lodzi, Oddzial Laboratoryjny Mikrobiologii i Parazytologii	Agnieszka Karnowska; Aleksandra Krawczyk; Danuta Bartczak; Elian Terpo; Katarzyna Kosowska; Lucyna Kochanowska; Marta Gawronska; Martyna Matera; Szymon Konkolewski
EPI_ISL_3251568	Medyczne Laboratoria Diagnostyczne INVICTA	Wojewodzka Stacja Sanitarno-Epidemiologiczna w Rzeszowie, Laboratorium Diagnostyki Medycznej	Anna Nowakowska; Karolina Ostrowska; Katarzyna Wilk; Marzena Baranowska
EPI_ISL_3637925	Michigan Department of Health and Human Services, Bureau of Laboratories	Michigan Department of Health and Human Services, Bureau of Laboratories	Blankenship HM; Riner D; Soehnlen MK
EPI_ISL_3241825, EPI_ISL_3470152, EPI_ISL_3470193, EPI_ISL_3470201	Microbiology Department. Complexo Hospitalario Universitario de Vigo	Microbiology Department. Complexo Hospitalario Universitario de Vigo	Alfaya N; Alonso I; Alvarez M; Cabrera JJ; Carballo R; Cores O; Cortizo S; Davina C; Martinez L; Mediero G; Perez S; Potel C; Regueiro B; Rey S; Vasallo FJ; del-Campo V
EPI_ISL_3410989, EPI_ISL_3410990	Ministry of Health Turkey	Ministry of Health Turkey	Fatma Bayrakdar; Gulay Korukluoglu; Suleyman Yalcin; Yasemin Cosgun
EPI_ISL_3319115	NORTHWELL HEALTH LABORATORIES	Wadsworth Center, New York State Department of Health	Alexis Russell; Catharine Prussing; Daryl M. Lamson; Erasmus Schneider; Erica Lasek-Nesselquist; John Kelly; Jonathan Plitnick; Kirsten St. George; Matthew Shudt; Melissa A Leisner; Navjot Singh
EPI_ISL_3045698	National Virus Reference Laboratory	National Virus Reference Laboratory	Charlene Bennett; Cillian F De Gascun; Gabriel Gonzalez; Jonathan Dean; Michael Carr; Zoe Yandle
EPI_ISL_3045076	New Mexico Department of Health Scientific Laboratory	New Mexico Department of Health Scientific Laboratory	Anastacia Griego-Fisher; D'eldra Malone; Ellie Johnson; Jennifer Benoit; Linda Salazar; Ratheesh Rajan
EPI_ISL_3447229	OLVZ Aalst	OLVZ Aalst	Astrid Holderbeke
EPI_ISL_3189600	Orebro University Hospital, Dept Laboratory Medicine, Clinical Microbiology	Orebro University Hospital	Sundqvist M et al
EPI_ISL_3120108	Ospedale di Vimercate	Laboratory of Clinical Microbiology, Virology and Bioemergencies, ASST Fatebenefratelli Sacco - Sacco Hospital	Valeria Micheli
EPI_ISL_3459407	PREVIS - IPS	Instituto Nacional de Salud	Carlos Franco-Muñoz; Carmen Osorio; Diana Malo; Diego A. Álvarez-Díaz; Diego Andrés Prada; Gerardo Santamaría; Hector Alejandro Ruiz-Moreno; Jhonnatan Reales-González; Jorge Rivera; Juan Camilo Martinez; Julian Naizaque; Katherine Laiton-Donato; Lisseth Pardo; Magdalena Wiesner; Marcela Mercado-Reyes; Maria T. Herrera-Sepúlveda; Marta Lopez Blanco; Martha Lucia Ospina Martinez; Paola Rojas; Sergio Gomez; Sheryll Corchuelo; Ángela Alarcon Cruz
EPI_ISL_3188507, EPI_ISL_3339534, EPI_ISL_3394195	Pandemic Response Lab - NYC	Pandemic Response Lab, R&D	Alex Carpio; Cybill del Castillo; Dylan Law; Haiping Hao; Henry Lee; Isabel Fernandez Escapa; Jon Laurent; Melissa Hopkins; Michael Hammerling; Pradeep Bugga; Shinyoung Clair Kang; Sol Rey; William Ward
EPI_ISL_3534151	Pennsylvania Department of Health Bureau of Laboratories	Pennsylvania Department of Health Bureau of Laboratories	Dongxiang Xia
EPI_ISL_3233768	Public Health Ontario Laboratory	Public Health Ontario Laboratory	Aimin Li; Alireza Eshaghi; Andre Villegas; Ashleigh Sullivan; Christine Frantz; Dean Maxwell; Esha Joshi; Jared Simpson; Jennifer L Guthrie; Jonathan B Gubbay; Karthikeyan Sivaraman; Lawrence Heisler; Matthew Watson; Michael CY Li; Michael Laszloffy; Nahuel Fittipaldi; Philip Banh; Richard de Borja; Samir N Patel; Sandeep Nagra; Sandra Zittermann; Sarah Teatero; Vanessa G Allen; Yao Chen; Yogi Sundaravadanam
EPI_ISL_3305019, EPI_ISL_3396975	Quest Diagnostics Incorporated	Centers for Disease Control and Prevention Division of Viral Diseases, Pathogen Discovery	A. Gerasimova; A. Perez; Adrian Paskey; B. Anderson; Benjamin Rambo-Martin; Christopher Gulvick; Clinton R. Paden; Dakota Howard; Darlene Wagner; Dhwani Batra; Duncan MacCannell; F. Lacbawan; I. A. Shlyakhter; Jason Caravas; K.E. Livingston; Kara Moser; L.E. Bernstein; M. Hua; Matthew Schmerer; P. Tanpaiboon; Peter W. Cook; R. M. Kagan; R. Owen; R. V. Rolando; S. H. Rosenthal; Scott Sammons; Shatavia Morrison; Y. Liu; Yvette Unoarumhi
EPI_ISL_3080350, EPI_ISL_3080965, EPI_ISL_3081128, EPI_ISL_3179560, EPI_ISL_3179619, EPI_ISL_3291244, EPI_ISL_3291275, EPI_ISL_3291363, EPI_ISL_3291371, EPI_ISL_3291394, EPI_ISL_3291513, EPI_ISL_3292319, EPI_ISL_3292337, EPI_ISL_3292509, EPI_ISL_3292999, EPI_ISL_3573331, EPI_ISL_3573820, EPI_ISL_3573839, EPI_ISL_3573990, EPI_ISL_3574030, EPI_ISL_3574031, EPI_ISL_3574292, EPI_ISL_3574474, EPI_ISL_3574614			
see above	Respiratory Virus Unit, Microbiology Services Colindale, Public Health England	COVID-19 Genomics UK (COG-UK) Consortium	PHE Covid Sequencing Team
EPI_ISL_3646351, EPI_ISL_3646354, EPI_ISL_3646358	SARS-CoV-2 Sequencing Castilla y Leon-Spain Consortium	SARS-CoV-2 Sequencing Castilla y Leon-Spain Consortium	Antonio Orduña-Domingo; Carlos Fuster Foz; Carmen Aldea-Mansilla; Carmen Gimeno Crespo; David Abad; Gabriel March Rosello; Gregoria Megías Lobón; Jose María Eiros Bouza; M. Isabel Fernandez-Natal; Marta Dominguez-Gil; Marta Hernandez; María Antonia García Castro; Mª Fe Brezmes-Valdivieso; Noelia Arenal Andrés; Silvia Rojo; Sonsoles Garcinuño Pérez
EPI_ISL_3342048	SC (UCO) Igiene e Sanità Pubblica, ASUGI, Trieste	ARGO Laboratorio Genomica ed Epigenomica	Barbone F; Braida C; Busetti M; D'Agaro P; Dal Monego S; Degasperi M; Licastro D; Maggiore A; Marcello A; Piscianz E; Segat L
EPI_ISL_3579329, EPI_ISL_3579330	SECRETARIA MUNCIPAL DE SAUDE DE SANTA BARBARA D OESTE	Instituto Butantan	Antonio Jorge Martins; Claudia Renata dos Santos Barros; David Schlesinger; Debora Botequio Moretti; Dimas Tadeu Covas; Elaine Cristina Marqueze; Elaine Vieira Santos; Evandra Strazza Rodrigues; Heidge Fukumasu; Jayme Augusto de Souza-Neto; José Salvatore Leister Patané; Luiz Alcantara; Luiz Lehmann Coutinho; Maria Carolina Elias; Maurício Lacerda Nogueira; Rafael dos Santos Bezerra; Raul Machado Neto; Rejane Maria Tommasini Grotto; Ricardo Haddad; Sandra Coccuzzo Sampaio Vessoni; Simone Kashima; Svetoslav Nanev Slavov; Vincent Louis Viala
EPI_ISL_3369920, EPI_ISL_3369921, EPI_ISL_3369931, EPI_ISL_3369943	SYNLAB - ANGEL DIAGNOSTICA	Corporacion CorpoGen-Universidad de los Andes-Universidad Central	Christian Romero; Jorge Duitama; Juan Manuel Anzola; Laura González; Maryam Chaib De Mares; María Mercedes Zambrano; Nelly Díaz; Patricia Del Portillo; Silvia Restrepo
EPI_ISL_3162242	Salud Digna	Instituto Nacional de Medicina Genomica	Abraham Campos-Romero; Blancas S; Cedro-Tanda A; Cisneros-Villanueva M; Escobar-Arrazola; Gonzalez-Barrera D; Herrera-Montalvo LA.; Hidalgo-Miranda A; Luna-Ruiz Marco; M; Mendoza-Vargas A; Moreno-Camacho José Luis; Munguia-Garza P; Ramirez-Vega O; Rangel-DeLeon D; Reyes-Grajeda JP; Rodriguez-Gallegos Jorge
EPI_ISL_3373987	Salud Digna, A.C	Andersen lab at Scripps Research	Abraham Garcia Gil; Jose Luis Moreno Camacho; Marco Antonio Luna Ruiz-Esparza; Miguel A. Fernandez Rojas; SEARCH Alliance with Abraham Campos Romero
EPI_ISL_3261726	Scripps Medical Laboratory	Andersen lab at Scripps Research	Ellen Stefanski; Ian Mchardy; SEARCH Alliance San Diego with Michael Quigley
EPI_ISL_3391987	Secretaria De Salud Del Guaviare	Instituto Nacional de Salud-Dirección de Investigación en Salud Pública	Carlos Franco-Muñoz; Carmen Osorio; Diana Malo; Diego A. Álvarez-Díaz; Diego Andrés Prada; Gerardo Santamaría; Hector Alejandro Ruiz-Moreno; Jhonnatan Reales-González; Jorge Rivera; Juan Camilo Martinez; Julian Naizaque; Katherine Laiton-Donato; Lisseth Pardo; Magdalena Wiesner; Marcela Mercado-Reyes; Maria T. Herrera-Sepúlveda; Marta Lopez Blanco; Martha Lucia Ospina Martinez; Paola Rojas; Sergio Gomez; Sheryll Corchuelo; Ángela Alarcon Cruz

EPI_ISL_3098720, EPI_ISL_3098721, EPI_ISL_3098722, EPI_ISL_3098723, EPI_ISL_3098724, EPI_ISL_3098725, EPI_ISL_3098726, EPI_ISL_3098727			
see above	Section of Microbiology Department of Molecular and Translational Medicine, University of Brescia	Section of Microbiology Department of Molecular and Translational Medicine, University of Brescia	Alberto Zani; Anna Bertelli; Arnaldo Caruso; Francesca Caccuri; Giovanni Campisi; Serena Messali
EPI_ISL_3154873	Shamir Medical Center (Asaf Harofe)	Shamir Medical Center (Asaf Harofe)	Abu Hamad Ramzia; Adina Bar Chaim; Anna Vishnevsky; Chen Weiner; Nir Rainy; Patricia Benveniste-Lekovitz; Reut Sorek Abramovich; Yevgeni Yegorov
EPI_ISL_3261400, EPI_ISL_3373781	Sharp HealthCare Laboratory	Andersen lab at Scripps Research	Art Mendoza; Cathy Woerle; Jacquelyn Berumen; Liam McGinnis; Omid Bakhtar; SEARCH Alliance San Diego with Aaron Harding
EPI_ISL_3045364, EPI_ISL_3231558, EPI_ISL_3231561, EPI_ISL_3259958, EPI_ISL_3425894	Stadtspital Triemli	Institute of Medical Virology	Alexandra Trkola; Annette Audigé; Catharine Aquino; Cyril Shah; Daniel Ehrsam; Gabriela Ziltener; Guido Bloemberg; Hubert Rehrauer; Isabel Stürmer; Joel Wirz; Jon Huder; Jürg Böni; Kevin Steiner; Maria Grünberg; Maryam Zaheri; Michael Huber; Riccarda Capaul; Stefan Schmutz; Verena Kufner; Weihong Qi
EPI_ISL_3326053	TGen North	TGen North	Brett Van Tassel; Chris French; Darrin Lemmer; Dave Engelthaler; Hayley Yaglom; Heather Centner; Jolene Bowers
EPI_ISL_3383994, EPI_ISL_3384016	TXDSHS	TXDSHS	Anita Pokharel; Bonnie Oh; Chun Wang; Grace Kubin; Jenny Zhang; Karen Bobier; Lorraine Rodriguez; Maliha Rahman; Mayela Pedrueza; Myong Koag; Rachel Lee; Rashmi Tuladhar
EPI_ISL_3373118	The Jackson Laboratory	The Jackson Laboratory	Adams M; Kelly K; Li L; Long J; Omerza G; Renzette N
EPI_ISL_3321312, EPI_ISL_3370451	UC-Christus Clinical Hospital Laboratory	Laboratory of Molecular Virology, School of Medicine, Pontificia Universidad Catolica de Chile	Ana Maira Guzman; Andres E. Munoz-Marcos; Catalina Pardo-Roa; Eileen Serrano; Erick Salinas; Estefany Poblete; Francisco Melo; Jorge Levican; Leonardo I. Almonacid; Maria Jose Avendano; Maria Patricia Vega; Rafael A. Medina; Ricardo Enrique de la Barra; Tamara Garcia-Salum
EPI_ISL_3105520	UCC	Grubaugh Lab - Yale School of Public Health	Anderson Brito; Andrea Firpo; Annie Watkins; Chaney Kalinich; Chantal Vogels; David Rivera Aponte; Ernesto Vazquez; Fabiola Cruz-Lopez; Fabiola Fontanet; Isabel Ott; Jessica Rothman; Joseph Fauver; Karolane Gonzalez; Kendall Billig; Mallery Breban; Mary Petrone; Natalie Machargo; Nathan Grubaugh; Patricia Serrano; Tania Mitwalli; Tara Alpert; Tobias Koch; Vianca Aponte
EPI_ISL_3188574, EPI_ISL_3188576, EPI_ISL_3188577, EPI_ISL_3188578, EPI_ISL_3188591, EPI_ISL_3188612, EPI_ISL_3188613, EPI_ISL_3188614, EPI_ISL_3188615, EPI_ISL_3188618, EPI_ISL_3188619, EPI_ISL_3188620, EPI_ISL_3236562, EPI_ISL_3369783, EPI_ISL_3369786, EPI_ISL_3369787, EPI_ISL_3369788, EPI_ISL_3500991, EPI_ISL_3500998			
see above	UCC (Universidad Central del Caribe)	Grubaugh Lab - Yale School of Public Health	Anderson Brito; Andrea Firpo; Annie Watkins; Chaney Kalinich; Chantal Vogels; David Rivera Aponte; Ernesto Vazquez; Fabiola Cruz-Lopez; Fabiola Fontanet; Isabel Ott; Jessica Rothman; Joseph Fauver; Karolane Gonzalez; Kendall Billig; Mallery Breban; Mary Petrone; Natalie Machargo; Nathan Grubaugh; Patricia Serrano; Tania Mitwalli; Tara Alpert; Tobias Koch; Vianca Aponte
EPI_ISL_3071377	ULS Castelo Branco	Instituto Nacional de Saude (INSA)	Borges et al
EPI_ISL_3369936	UNIVERSIDAD DEL QUINDIO	Corporacion CorpoGen-Universidad de los Andes-Universidad Central	Christian Romero; Jorge Duitama; Juan Manuel Anzola; Laura González; Maryam Chaib De Mares; María Mercedes Zambrano; Nelly Díaz; Patricia Del Portillo; Silvia Restrepo
EPI_ISL_3236599, EPI_ISL_3412244	UW Virology Lab	UW Virology Lab	Alexander Greninger; Hong Xie; Keith R Jerome; Maria Lukes; Meei-Li Huang; Nathan Breit; Patrick Mathias; Pavitra Roychoudhury; Ricardo Perez; Robert J. Livingston; Sean Ellis; Shah Mohamed Bakhash; Tien V. Nguyen
EPI_ISL_3277390, EPI_ISL_3277391	Unidad de Investigaciones Moleculares (UNIMOL) - Universidad de Cartagena	Centro de Genética y Biología Molecular - Universidad del Magdalena	Andrea M. Ramírez Hernandez; Angel Oviedo Marquez; Daniel Bautista; Lyda R. Castro; Maria Teresa Mojica-Ortiz
EPI_ISL_3072495	Unidade de apoio ao diagnostico da COVID - UNADIG	Bioinformatics Laboratory / LNCC	Alessandra P Lamarca; Alexandra L Gerber; Amilcar Tanuri; Ana Paula de C Guimaraes; Ana Tereza R Vasconcelos; Andrea Cony Cavalcanti; Caio Luiz Pereira Ribeiro; Cassia Alves; Cintia Policarpo; Claudia Maria Braga de Mello; Cristiane Gomes da Silva; Diana Mariani; Douglas Terra Machado; Flavio Dias da Silva; Gleidson da Silva de Oliveira; Leandro Magalhaes de Souza; Liliane Cavalcante; Luiz G P de Almeida; Marcio Henrique de Oliveira Garcia; Mario Sergio Ribeiro; Ronaldo da Silva F Jr; Silvia Carvalho
EPI_ISL_3505613	Universidad Estatal de Bolivar	Institute of Microbiology, Universidad San Francisco de Quito	Anita García; Belén Prado-Vivar; Bernardo Gutiérrez; Carlos Julio Tobar; Erika B. Muñoz; Fernanda Zurita; Gabriel Trueba; Juan José Guadalupe; Karina Barragán; Michelle Grunauer; Monica Becerra-Wong; Patricio Rojas-Silva; Paúl Cárdenas; René Bracho; Stephanie Arregui; Sully Márquez; Verónica Barragán
EPI_ISL_3276649, EPI_ISL_3276650	Universidad Nacional de Colombia - Laboratorio Genómico One Health	Universidad Nacional de Colombia - Laboratorio Genómico One Health	Andres F. Cardona-Rios; Carlos Franco-Muñoz; Carolina Muñoz-Arango; Celeny Ortiz; Daniel O. Maldonado-Perez; Diego A. Álvarez-Díaz; Hector Alejandro Ruiz-Moreno; Idabely Betancur Ortiz; Jorge E. Osorio; Juan P. Hernandez-Ortiz; Karl A Ciuoderis; Katherine Laiton-Donato; Laura Silvana Perez; Lina M. Hurtado; Marcela Mercado-Reyes; Maria Angélica Maya; Maria Stella López; Rita Almanza Payares; Sandra Ines Cano; Simón Villegas Velásquez
EPI_ISL_3032253, EPI_ISL_3118424	University of Liège COVID-19 testing center	GIGA Medical Genomics	Bouchra Boujemla; Claire Gourzonès; Cécile Meex; Keith Durkin; Laurent Gillet; Maria Artesi; Marie-Pierre Hayette; Nadine Cambisano; Nathalie Renotte; Olivier Ek; Sébastien Bontems; Vincent Bours
EPI_ISL_3259950	Universität Zürich	Institute of Medical Virology	Alexandra Trkola; Annette Audigé; Catharine Aquino; Cyril Shah; Daniel Ehrsam; Gabriela Ziltener; Guido Bloemberg; Hubert Rehrauer; Isabel Stürmer; Joel Wirz; Jon Huder; Jürg Böni; Kevin Steiner; Maria Grünberg; Maryam Zaheri; Michael Huber; Riccarda Capaul; Stefan Schmutz; Verena Kufner; Weihong Qi
EPI_ISL_3236501, EPI_ISL_3347353, EPI_ISL_3369777, EPI_ISL_3369779, EPI_ISL_3369780, EPI_ISL_3369785	VIDOH (Virgin Islands Department of Health)	Grubaugh Lab - Yale School of Public Health	Anderson Brito; Annie Watkins; Brett Ellis; Chaney Kalinich; Chantal Vogels; Esther Ellis; Isabel Ott; Jendai Richards; Jessica Rothman; Joseph Fauver; Kendall Billig; Mallery Breban; Marlon Lawrence; Mary Petrone; Nathan Grubaugh; TaLesa Aderohunmu; Tara Alpert; Tobias Koch
EPI_ISL_3123367, EPI_ISL_3123368	WSSE w Gdansku	Wojewodzka Stacja Sanitarno-Epidemiologiczna w Olsztynie, Laboratorium Badan Epidemiologiczno-Klinicznych	Aleksandra Kobiatko; Barbara Dolinska; Emilia Tarabasz; Ewa Liszewska; Marta Lukian; Monika Czerminska; Patryk Bielecki; Paulina Rozycka; Sylwia Krzetowska; Tomasz Jakubczak
EPI_ISL_3587050	Wisconsin State Laboratory of Hygiene Communicable Disease Division	Wisconsin State Laboratory of Hygiene Communicable Disease Division	Abigail C. Shockey; Alicia J. Mooney; Erika M. Hanson; Kelsey R. Florek; Richard Griesser; Sara Wagner; Tonya Danz
EPI_ISL_3188596	Yale Clinical Virology Lab	Grubaugh Lab - Yale School of Public Health	Anderson Brito; Annie Watkins; Chaney Kalinich; Chantal Vogels; Isabel Ott; Jessica Rothman; Joseph Fauver; Kendall Billig; Mallery Breban; Marie L. Landry; Mary Petrone; Nathan Grubaugh; Tara Alpert; Tobias Koch
EPI_ISL_3539208	ZLC Zentrallabor Chur	Microbiology, Dr. Risch	Dominique Fabien Hilti; Faina Wehrli; Lorenz Risch; Martin Risch; Nadia Wohlwend; Sinem Kas; Thomas Bodmer
EPI_ISL_3115647, EPI_ISL_3115672	ZOTZ KLIMAS MVZ Düsseldorf-Centrum GbR ÜBAG für Labormedizin, Genetik, Zytologie, Pathologie	Center of Medical Microbiology, Virology, and Hospital Hygiene, University of Duesseldorf	Alexander Dilthey; Andreas Walker; Daniel Strelow; Jessica Nicolai; Jörg Timm; Katrin Hoffmann; Klaus Pfeffer; Lisanna Hülse; Malte Kohns Vasconcelos; Maximilian Damagnez; Nadine Lübke; Patrick Finzer; Rainer Zotz; Tobias Wienemann; Torsten Houwaart

We gratefully acknowledge the following Authors from the Originating laboratories responsible for obtaining the specimens, as well as the Submitting laboratories where the genome data were generated and shared via GISAID, on which this research is based.

All Submitters of data may be contacted directly via www.gisaid.org

Authors are sorted alphabetically.

Acknowledgement EPI_SET Identifier: EPI_SET_20220328wg

Accession ID	Originating Laboratory	Submitting Laboratory	Authors
EPI_ISL_3710325	A. Krumbholz, Labor Dr. Krause und Kollegen MVZ GmbH, Kiel	Charité Universitätsmedizin Berlin, Institut für Virologie	Barbara Mühlemann; Christian Drosten; Julia Schneider; Jörn Beheim-Schwarzbach; Talitha Veith; Terry Jones; Victor M Corman
EPI_ISL_3878175	AFIP	Instituto Butantan	Antonio Jorge Martins; Claudia Renata dos Santos Barros; David Schlesinger; Debora Botequio Moretti; Dimas Tadeu Covas; Elaine Cristina Marqueze; Elaine Vieira Santos; Evandra Strazza Rodrigues; Heidge Fukumasu; Jayme Augusto de Souza-Neto; José Salvatore Leister Patané; Luiz Alcantara; Luiz Lehmann Coutinho; Maria Carolina Elias; Maurício Lacerda Nogueira; Rafael dos Santos Bezerra; Raul Machado Neto; Rejane Maria Tommasini Grotto; Ricardo Haddad; Sandra Coccuzzo Sampaio Vessoni; Simone Kashima; Svetoslav Nanev Slavov; Vincent Louis Viala
EPI_ISL_4258609	AREA DE SALUD ATENAS	Inciensa, Instituto Costarricense de Investigación y Enseñanza en Nutrición y Salud	Adriana Godínez; Claudio Soto-Garita; Estela Cordero; Francisco Duarte; Hebleen Porras; Joselyn Prado & Marilyn Alfaro Segura; José Luis Vargas; Mariela Gutiérrez; Melany Calderón
EPI_ISL_4258613	AREA DE SALUD CORRALILLO	Inciensa, Instituto Costarricense de Investigación y Enseñanza en Nutrición y Salud	Adriana Godínez; Claudio Soto-Garita; Estela Cordero; Francisco Duarte; Hebleen Porras; Joselyn Prado & Natasha Tames Robles; José Luis Vargas; Mariela Gutiérrez; Melany Calderón
EPI_ISL_3948514	AREA DE SALUD CORREDORES	Inciensa, Instituto Costarricense de Investigación y Enseñanza en Nutrición y Salud	Adriana Godínez; Claudio Soto-Garita; Estela Cordero; Francisco Duarte; Hebleen Porras; Joselyn Prado & Raul Zeledon; José Luis Vargas; Mariela Gutiérrez; Melany Calderón
EPI_ISL_3948546	AREA DE SALUD DESAMPARADOS 2 (COOPESALUD)	Inciensa, Instituto Costarricense de Investigación y Enseñanza en Nutrición y Salud	Adriana Godínez; Claudio Soto-Garita; Estela Cordero; Francisco Duarte; Hebleen Porras; Joselyn Prado & Juliana Mora; José Luis Vargas; Mariela Gutiérrez; Melany Calderón
EPI_ISL_3948532	AREA DE SALUD HEREDIA-VIRILLA	Inciensa, Instituto Costarricense de Investigación y Enseñanza en Nutrición y Salud	Adriana Godínez; Claudio Soto-Garita; Estela Cordero; Francisco Duarte; Hebleen Porras; José Luis Vargas; Mariela Gutiérrez & Joselyn Prado; Melany Calderón
EPI_ISL_3948547	AREA DE SALUD PAVAS (COOPESALUD)	Inciensa, Instituto Costarricense de Investigación y Enseñanza en Nutrición y Salud	Adriana Godínez; Claudio Soto-Garita; Estela Cordero; Francisco Duarte; Hebleen Porras; Joselyn Prado & Juliana Mora; José Luis Vargas; Mariela Gutiérrez; Melany Calderón
EPI_ISL_3948539	AREA DE SALUD PEREZ ZELEDON	Inciensa, Instituto Costarricense de Investigación y Enseñanza en Nutrición y Salud	Adriana Godínez; Claudio Soto-Garita; Estela Cordero; Francisco Duarte; Hebleen Porras; Joselyn Prado & Maria Fernanda Matamoros; José Luis Vargas; Mariela Gutiérrez; Melany Calderón
EPI_ISL_3753039, EPI_ISL_3816035, EPI_ISL_3819770, EPI_ISL_3819821, EPI_ISL_3819866, EPI_ISL_3820061, EPI_ISL_3820574, EPI_ISL_3820602, EPI_ISL_3822426, EPI_ISL_3822966, EPI_ISL_3843163, EPI_ISL_3843310, EPI_ISL_3844481, EPI_ISL_3849009, EPI_ISL_3851821, EPI_ISL_3852069, EPI_ISL_3852100, EPI_ISL_3855473, EPI_ISL_3857415, EPI_ISL_3857693, EPI_ISL_3860382, EPI_ISL_3860905, EPI_ISL_3863969, EPI_ISL_4186127, EPI_ISL_4186503, EPI_ISL_4186511, EPI_ISL_4360230, EPI_ISL_4360318, EPI_ISL_4361298, EPI_ISL_4361343, EPI_ISL_4361511, EPI_ISL_4361546, EPI_ISL_4361931, EPI_ISL_4362047, EPI_ISL_4362325, EPI_ISL_4362892, EPI_ISL_4362925, EPI_ISL_4363609, EPI_ISL_4364033, EPI_ISL_4364034, EPI_ISL_4364841, EPI_ISL_4365036, EPI_ISL_4365285, EPI_ISL_4365669, EPI_ISL_4366082, EPI_ISL_4366486			
see above	Aegis Sciences Corporation	Centers for Disease Control and Prevention Division of Viral Diseases, Pathogen Discovery	Adrian Paskey; Alec Vest; Benjamin Rambo-Martin; Christopher Gulvick; Clinton Paden; Cyndi Clark; Dakota Howard; Darlene Wagner; Dhwani Batra; Dillon Nall; Duncan MacCannell; Erisa Sula; Ethan Sanders; Holly Houdeshell; Jason Caravas; Kara Moser; Kristine Lacek; Matthew Hardison; Matthew Schmerer; Ola Kvalvaag; Patrick Campbell; Peter Cook; Rob Case; Scott Sammons; Shatavia Morrison; Shaun Westlund; Tymeckia Kendall; Victoria Caban Figueroa; Vikramsinha Ghorpade; Yvette Unoarumhi
EPI_ISL_3668671, EPI_ISL_4072194, EPI_ISL_4199029	Alaska State Virology Laboratory	Alaska State Virology Laboratory	Elva House; Jack Chen; Jacob Zidek; Lisa Smith; Ph.D.; Stephanie DeRonde
EPI_ISL_3721582	Ayudas Diagnosticas Sura	Universidad Nacional de Colombia - Laboratorio Genómico One Health	Andres F. Cardona-Rios; Carlos Franco-Muñoz; Carolina Muñoz-Arango; Celeny Ortiz; Daniel O. Maldonado-Perez; Diego A. Álvarez-Díaz; Hector Alejandro Ruiz-Moreno; Idabely Betancur Ortiz; Jorge E. Osorio; Juan P. Hernandez-Ortiz; Karl A Ciuoderis; Katherine Laiton-Donato; Laura Silvana Perez; Lina M. Hurtado; Marcela Mercado-Reyes; Maria Angélica Maya; Maria Stella López; Rita Almanza Payares; Sandra Ines Cano; Simón Villegas Velásquez
EPI_ISL_3724153, EPI_ISL_4094266	Broad Institute Clinical Research Sequencing Platform	Infectious Disease Program, Broad Institute of Harvard and MIT	Adams, G.; B.L.; B.W.; Bauer, M.; Birren; Blumenstiel, B.; Brown, C.; Carter, A.; Chaluvadi, S.; D.J.; DeFelice, M.; DeRuff, K.; Dodge, S.; Gabriel, S.; Gallagher, G.; Gladden-Young, A.; Granger, B.; J.E.; K.J.; Lagerborg, K.; Larkin, K.; Lee, M.; Lemieux; Lennon, N.; Loreth, C.; Madoff, L.; McGovern, S.; Meldrim, J.; Normandin, E.; P.C.; Park; Pearlman, L.; Reilly, S.; Rudy, M.; Sabeti; Siddle; Smole, S.; Tomkins-Tinch, C.; Vicente, G.; and MacInnis
EPI_ISL_3737595	CHR Verviers	GIGA Medical Genomics	Bouchra Boujemla; Claire Gourzonès; Cécile Meex; Keith Durkin; Laurent Gillet; Maria Artesi; Marie-Pierre Hayette; Nadine Cambisano; Nathalie Renotte; Olivier Ek; Sébastien Bontems; Vincent Bours
EPI_ISL_3838194, EPI_ISL_3838195, EPI_ISL_3838196	CHU NIMES	CHU NIMES	Agathe Boudet; Marie-Josée Carles; Sophie Bravo; Stephan Robin
EPI_ISL_3667141	COVID LAB	Cayman Islands Forensic Science Lab	Brittany Balcewich; Jonathan Smellie; Karla Montes; Tanisha Gilbert
EPI_ISL_4299725	Centro de Investigación Biomédica de Occidente (CIBO)	Centro de Investigación en Enfermedades Infecciosas (CIENI), Instituto Nacional de Enfermedades Respiratorias (INER)	Alejandra García-Gasca; Alejandra Hernández-Terán; Alejandro Sánchez-Flores; Alfredo Herrera-Estrella; Alicia Ocaña-Mondragón; Andreu Comas-García; Angel Gustavo Salas-Lais; Antonio Loza Román; Bernardo Martínez-Miguel; Blanca Taboada; Brenda Irasema Maldonado-Meza; Bruno Gómez-Gil; Carla Ivón Herrera-Najera; Carlos F. Arias; Celia Boukadida; Clara Esperanza Santacruz-Tinoco; Concepción Grajales-Muñiz; Consorcio Mexicano de Vigilancia Genómica (CoViGen-Mex). Authors (in alphabetical order): Julio Elias Alvarado-Yaah; Cristóbal Cháidez-Quiróz; Célida Duque Molina; Célida Martínez-Rodríguez; Daniel Fregoso-Rueda; Daniel Lira Morales; Eduardo Becerril-Vargas; Eduardo Rivera-Martínez; Fernando Fontove-Herrera; Fidencio Mejía-Nepomuceno; Francisco Pulido; Gabriel Chavira-Trujillo; Gloria Elena Espinosa-Ayala; Gloria María Molina-Salinas; Gloria Vazquez; Hector Montoya-Fuentes; Helen Haydee Fernanda Ramírez-Plascencia; Irvin González-López; Jean Pierre González; Jesús Hernández; Joel Armando Vázquez-Pérez.; Jorge Salas-Hernández; José Antonio Enciso-Moreno; José Arturo Martínez-Orozco; José Esteban Muñoz-Medina; José de Jesús Nuñez-Contreras; Juan Bautista Chale-Dzul; Julissa Enciso-Ibarra; Kathia Elizabeth Tapia-Diaz; Luis Alberto Ochoa-Carrera; Margarita Matías-Florentino; Mario Mújica-Sánchez; Marissa Perez-Garcia; María Eugenia Jiménez-Corona; María Guadalupe Santiago-Mauricio; María Guadalupe de Jesús Mireles-Rivera; Nelly Sélem-Mojica; Pavel Isa; Ricardo Ciria Merce; Ricardo Grande; Rosa María Gutiérrez Rios; Rosario Vazquez-Larios; Santiago Ávila-Ríos; Selene Zárate; Susana Lopez; Verónica Mata-Haro; Victor Eduardo García-Arias; Víctor Hugo Borja-Aburto
EPI_ISL_4006620, EPI_ISL_4006667	Centro de Investigación Biomédica de Occidente (CIBO)	Instituto de Biotecnología de la UNAM	; Alejandra García-Gasca; Alejandra Hernández-Terán; Alejandro Sánchez-Flores; Alfredo Herrera-Estrella; Alicia Ocaña-Mondragón; Andreu Comas-García; Angel Gustavo Salas-Lais; Antonio Loza Román; Bernardo Martínez-Miguel; Blanca Taboada; Brenda Irasema Maldonado-Meza; Bruno Gómez-Gil; Carla Ivón Herrera-Najera; Carlos F. Arias; Celia Boukadida; Clara Esperanza Santacruz-Tinoco; Concepción Grajales-Muñiz; Consorcio Mexicano de Vigilancia Genómica (CoViGen-Mex). Authors (in alphabetical order): Julio Elias Alvarado-Yaah; Cristóbal Cháidez-Quiróz; Célida Duque Molina; Célida Martínez- Rodríguez; Daniel Fregoso-Rueda; Daniel Lira Morales; Eduardo Becerril-Vargas; Fernando Fontove-Herrera; Fidencio Mejía-Nepomuceno; Francisco Pulido; Gloria Elena Espinosa-Ayala; Gloria María Molina-Salinas; Gloria Vazquez; Hector Esteban Paz-Juárez; Hector Montoya-Fuentes; Helen Haydee Fernanda Ramírez-Plascencia; Irvin González-López; Jean Pierre González; Jesús Hernández; Joel Armando Vázquez-Pérez).; Jorge Salas-Hernández; José Antonio Enciso-Moreno; José Arturo Martínez-Orozco; José Esteban Muñoz-Medina; José de Jesús Nuñez-Contreras; Juan Bautista Chale-Dzul; Julissa Enciso-Ibarra; Kathia Elizabeth Tapia-Diaz; Luis Alberto Ochoa-Carrera; Margarita Matías-Florentino; Mario Mújica-Sánchez; Marissa Perez-Garcia; María Guadalupe Santiago-Mauricio; María Guadalupe de Jesús Mireles-Rivera; Nelly Sélem-Mojica; Pavel Isa; Ricardo Ciria Merce; Ricardo Grande; Rosa María Gutiérrez Rios; Santiago Ávila-Ríos; Selene Zárate; Susana Lopez; Verónica Mata-Haro; Victor Eduardo García-Arias; Víctor Hugo Borja-Aburto
EPI_ISL_4006308	Centro de Investigación Biomédica del Noreste (CIBIN)	Instituto de Biotecnología de la UNAM	; Alejandra García-Gasca; Alejandra Hernández-Terán; Alejandro Sánchez-Flores; Alfredo Herrera-Estrella; Alicia Ocaña-Mondragón; Andreu Comas-García; Angel Gustavo Salas-Lais; Antonio Loza Román; Bernardo Martínez-Miguel; Blanca Taboada; Brenda Irasema Maldonado-Meza; Bruno Gómez-Gil; Carla Ivón Herrera-Najera; Carlos F. Arias; Celia Boukadida; Clara Esperanza Santacruz-Tinoco; Concepción Grajales-Muñiz; Consorcio Mexicano de Vigilancia Genómica (CoViGen-Mex). Authors (in alphabetical order): Julio Elias Alvarado-Yaah; Cristóbal Cháidez-Quiróz; Célida Duque Molina; Célida Martínez- Rodríguez; Daniel Fregoso-Rueda; Daniel Lira Morales; Eduardo Becerril-Vargas; Fernando Fontove-Herrera; Fidencio Mejía-Nepomuceno; Francisco Pulido; Gloria Elena Espinosa-Ayala; Gloria María Molina-Salinas; Gloria Vazquez; Hector Esteban Paz-Juárez; Hector Montoya-Fuentes; Helen Haydee Fernanda Ramírez-Plascencia; Irvin González-López; Jean Pierre González; Jesús Hernández; Joel Armando Vázquez-Pérez).; Jorge Salas-Hernández; José Antonio Enciso-Moreno; José Arturo Martínez-Orozco; José Esteban Muñoz-Medina; José de Jesús Nuñez-Contreras; Juan Bautista Chale-Dzul; Julissa Enciso-Ibarra; Kathia Elizabeth Tapia-Diaz; Luis Alberto Ochoa-Carrera; Margarita Matías-Florentino; Mario Mújica-Sánchez; Marissa Perez-Garcia; María Guadalupe Santiago-Mauricio; María Guadalupe de Jesús Mireles-Rivera; Nelly Sélem-Mojica; Pavel Isa; Ricardo Ciria Merce; Ricardo Grande; Rosa María Gutiérrez Rios; Santiago Ávila-Ríos; Selene Zárate; Susana Lopez; Verónica Mata-Haro; Victor Eduardo García-Arias; Víctor Hugo Borja-Aburto
EPI_ISL_4056032	City of Milwaukee Health Department Laboratory	City of Milwaukee Health Department Laboratory	Amy Bauer; Manjeet Khubbar; Nandu Balakrishnan; Samantha Scott; Sanjib Bhattacharyya
EPI_ISL_3709760, EPI_ISL_3709761,	Department of Bacteria, Parasites and	Statens Serum Institut Bioinformatics and	Danish Covid-19 Genome Consortium

EPI_ISL_3709765	Fungi, Statens Serum Institut, Copenhagen, Denmark	Microbial Genomics	
EPI_ISL_4006772	Diagnóstico CIB	Corporación para Investigaciones Biológicas-CIB	Jeanneth Mosquera Rendon; Katterine Molina Hoyos; Marcela Mercado Reyes; Uriel A. Hurtado Paez
EPI_ISL_3771724, EPI_ISL_3771736, EPI_ISL_3771754, EPI_ISL_3771761, EPI_ISL_3771763, EPI_ISL_3771765, EPI_ISL_3771767, EPI_ISL_3771769, EPI_ISL_3771771, EPI_ISL_3771774, EPI_ISL_3771776, EPI_ISL_3917534, EPI_ISL_3917537, EPI_ISL_3917539, EPI_ISL_4076724, EPI_ISL_4077161, EPI_ISL_4077321, EPI_ISL_4077325			
see above	Dutch COVID-19 response team	National Institute for Public Health and the Environment (RIVM)	Adam Meijer; AnneMarie van den Brandt; Annelies Kroneman; Bas van der Veer; Chantal Reusken; Dennis Schmitz; Dirk Eggink; Euníce Then; Florian Zwagemaker; Harry Vennema; Ivo van Walle; Jeroen Cremer; Karim Hajji; Kim Freriks; Lisa Wijsman; Lynn Aarts; Ryanne Jaarsma; Sanne Bos; Sharon van den Brink; Stijn van Rossum; on behalf of the national COVID-19 response team
EPI_ISL_3941383, EPI_ISL_3941388, EPI_ISL_3941390, EPI_ISL_3941391, EPI_ISL_3941392, EPI_ISL_3941393, EPI_ISL_3941394, EPI_ISL_3941395, EPI_ISL_3941396, EPI_ISL_3941397, EPI_ISL_3941402, EPI_ISL_3941405, EPI_ISL_3941406, EPI_ISL_3941407, EPI_ISL_3941409, EPI_ISL_3941413, EPI_ISL_3941418, EPI_ISL_3941422, EPI_ISL_3941424, EPI_ISL_3941425, EPI_ISL_3941426			
see above	Edmonton Provincial Lab	Alberta Precision Labs (APL)	Buss; Croxen M; Deo A; Dieu P; E; Ferrato C; Gill K; Khan F; Koleva P; Li V; Lloyd C; Lynch T; Ma R; Murphy S; Pabbaraju K; Shokoples S; Thayer J; Tipples G; Whitehouse M; Wong A; Yu C; Zelyas N
EPI_ISL_3661081, EPI_ISL_3661421, EPI_ISL_3719766, EPI_ISL_4160689, EPI_ISL_4184283, EPI_ISL_4358179, EPI_ISL_4366972, EPI_ISL_4367467, EPI_ISL_4367935, EPI_ISL_4368833, EPI_ISL_4369438, EPI_ISL_4369471, EPI_ISL_4370473, EPI_ISL_4371167, EPI_ISL_4371547			
see above	Fulgent Genetics	Centers for Disease Control and Prevention Division of Viral Diseases, Pathogen Discovery	Adrian Paskey; Becky Tsai; Benafsh Sapra; Benjamin Rambo-Martin; Christopher Gulvick; Clinton Paden; Dakota Howard; Darlene Wagner; Dhwani Batra; Doreen Ng; Duncan MacCannell; Erisa Sula; Harry Gao; James Xie; Jason Caravas; John Gao; Joseph Fierro; Kara Moser; Kristine Lacek; Matthew Schmerer; Mickey Li; Peter Cook; Scott Sammons; Shatavia Morrison; Tymeckia Kendall; Victoria Caban Figueroa; Yan Meng; Yvette Unoarumhi
EPI_ISL_4196877, EPI_ISL_4198483, EPI_ISL_4198494, EPI_ISL_4198499, EPI_ISL_4198500	Gencore - Universidad de los Andes	Gencore - Universidad de los Andes	Cristian Barrera; David González; Gabriela Ariza; Luisa Sacristan; Marcela Guevara; Silvia Restrepo
EPI_ISL_3987681	Genetica Molecular and Subdepartamento de Virologia ISP Chile	Instituto de Salud Publica de Chile	Andres Castillo; Barbara Parra; Constanza Campano; Gisselle Barra; Javier Tognarelli; Jorge Fernandez; Karen Orostica; Loredana Arata; Patricia Bustos; Rodrigo Fasce; Soledad Ulloa
EPI_ISL_3799749	Gibraltar Health Authority Lab	Gibraltar Health Authority Covid-19 Laboratory	Dr Daniel Cassaglia; Dr Martyn Bell; Dr Nicholas Cortes; Dr Zoe Vincent
EPI_ISL_3761809, EPI_ISL_3761810	HOSPITAL NACIONAL DE NIÑOS	Inciensa, Instituto Costarricense de Investigación y Enseñanza en Nutrición y Salud	Cristian Pérez-Corrales & Valeria Peralta-Barquero
EPI_ISL_4206000	HOSPITAL UNIVERSITARIO 12 DE OCTUBRE	HOSPITAL UNIVERSITARIO 12 DE OCTUBRE	Carmen Martín-Higuera; Esther Viedma; Irene Múñoz-Gallego; M.ª Dolores Folgueira; Mar Aguilera; Noelia Moral; Rafael Delgado; Sagrario Zurita
EPI_ISL_3834651, EPI_ISL_3834652	HOSPITAL UNIVERSITARIO CENTRAL DE ASTURIAS	Laboratorio de Virología HUCA	; Alba L; Alvarez-Arguelles ME; Boga JA; Costales I; Coto E; González-Alba JM; Gómez de Oña J; Martín-Rodríguez G; Melón S; Perez-Martínez Z; Rojo S; Sandoval M
EPI_ISL_4345910, EPI_ISL_4346277, EPI_ISL_4346509, EPI_ISL_4347185, EPI_ISL_4347267, EPI_ISL_4347631	Helix/Illumina	Centers for Disease Control and Prevention Division of Viral Diseases, Pathogen Discovery	Adrian Paskey; Alexandre Bolze; Ary Ascencio; Benjamin Rambo-Martin; Brad Sickler; Charlotte Rivera-Garcia; Christopher Gulvick; Chrstine Tran; Clinton Paden; Dakota Howard; Darlene Wagner; David Becker; Dhwani Batra; Duncan MacCannell; Efren Sandoval; Eileen De Feo; Elizabeth Cirulli; Eric Allen; Geraint Levan; James Lu; Jan Antico; Jason Caravas; Jason Nguyen; Jimmy Ramirez; Jingtao Liu; Kara Moser; Kelly Barrett; Kim Gietzen; Kristine Lacek; Magnus Isaksson; Marc Laurent; Matthew Schmerer; Matthew Tolentino; Nicole Washington; Peter Cook; Phil Febbo; Ryan Cho; Scott Sammons; Shannon Wickline; Shatavia Morrison; Sherry Wang; Simon White; Tyler Cassens; William Lee; Yvette Unoarumhi
EPI_ISL_3671012, EPI_ISL_3671057	Hospital Fundacion San Vicente de Paul	Universidad Nacional de Colombia - Laboratorio Genómico One Health	Andres F. Cardona-Rios; Carlos Franco-Muñoz; Carolina Muñoz-Arango; Celeny Ortiz; Daniel O. Maldonado-Perez; Diego A. Álvarez-Díaz; Hector Alejandro Ruiz-Moreno; Idabely Betancur Ortiz; Jorge E. Osorio; Juan P. Hernandez-Ortiz; Karl A Ciuoderis; Katherine Laiton-Donato; Laura Silvana Perez; Lina M. Hurtado; Marcela Mercado-Reyes; Maria Angélica Maya; Maria Stella López; Rita Almanza Payares; Sandra Ines Cano; Simón Villegas Velásquez
EPI_ISL_4351984	Hospital General Universitario Gregorio Marañón	Hospital General Universitario Gregorio Marañón	Cristina Rodriguez-Grande; Darío García de Viedma; Julia Suárez; Laura Pérez-Lago; Marta Herranz Martin; Patricia Muñoz; Pedro Sola Campoy; Pilar Catalán; Sergio Buenestado Serrano; Victor Manuel de la Cueva
EPI_ISL_3671065	Hospital Pablo Tobon	Universidad Nacional de Colombia - Laboratorio Genómico One Health	Andres F. Cardona-Rios; Carlos Franco-Muñoz; Carolina Muñoz-Arango; Celeny Ortiz; Daniel O. Maldonado-Perez; Diego A. Álvarez-Díaz; Hector Alejandro Ruiz-Moreno; Idabely Betancur Ortiz; Jorge E. Osorio; Juan P. Hernandez-Ortiz; Karl A Ciuoderis; Katherine Laiton-Donato; Laura Silvana Perez; Lina M. Hurtado; Marcela Mercado-Reyes; Maria Angélica Maya; Maria Stella López; Rita Almanza Payares; Sandra Ines Cano; Simón Villegas Velásquez
EPI_ISL_4112985	Hospital Universitari Arnau de Vilanova	Hospital Universitari Vall d'Hebron - Vall d'Hebron Institut de Recerca	Alejandra González-Sánchez; Andrés Antón; Ariadna Rando; Carla Castillo; Cristina Andrés; Damir Garcia-Cehic; Josep Quer; Juliana Esperalba; Karen García; Maria Carmen Martin; Maria Gema Codina; Maria Piñana; Rodrigo Vásquez; Tomàs Pumarola
EPI_ISL_3983941, EPI_ISL_3984119	Hospital Universitari Vall d'Hebron - Vall d'Hebron Institut de Recerca	Hospital Universitari Vall d'Hebron - Vall d'Hebron Institut de Recerca	Alejandra González-Sánchez; Andrés Antón; Ariadna Rando; Carla Castillo; Cristina Andrés; Damir Garcia-Cehic; Josep Quer; Juliana Esperalba; Karen García; Maria Carmen Martin; Maria Gema Codina; Maria Piñana; Rodrigo Vásquez; Tomàs Pumarola
EPI_ISL_3721580, EPI_ISL_3721606	ICMT-Apartado	Universidad Nacional de Colombia - Laboratorio Genómico One Health	Andres F. Cardona-Rios; Carlos Franco-Muñoz; Carolina Muñoz-Arango; Celeny Ortiz; Daniel O. Maldonado-Perez; Diego A. Álvarez-Díaz; Hector Alejandro Ruiz-Moreno; Idabely Betancur Ortiz; Jorge E. Osorio; Juan P. Hernandez-Ortiz; Karl A Ciuoderis; Katherine Laiton-Donato; Laura Silvana Perez; Lina M. Hurtado; Marcela Mercado-Reyes; Maria Angélica Maya; Maria Stella López; Rita Almanza Payares; Sandra Ines Cano; Simón Villegas Velásquez
EPI_ISL_4198473	IDIME	Gencore - Universidad de los Andes	Cristian Barrera; David González; Gabriela Ariza; Luisa Sacristan; Marcela Guevara; Marcela Mercado; Silvia Restrepo
EPI_ISL_4344406	Infinity Biologix	Centers for Disease Control and Prevention Division of Viral Diseases, Pathogen Discovery	Benjamin Rambo-Martin; Chirayu Goswami; Christian Bixby; Christopher Gulvick; Clinton Paden; Dakota Howard; Dhwani Batra; Duncan MacCannell; Erisa Sula; Jason Caravas; Jonathan Schultz; Kristine Lacek; Matthew Schmerer; Peter Cook; Robin Grimwood; Russ Hager; Scott Sammons; Shatavia Morrison; Tymeckia Kendall; Victoria Caban Figueroa; Yihe Wang; Yvette Unoarumhi
EPI_ISL_3721577	Instituto Colombiano Medicina Tropical ICMT - Sede Sabaneta	Universidad Nacional de Colombia - Laboratorio Genómico One Health	Andres F. Cardona-Rios; Carlos Franco-Muñoz; Carolina Muñoz-Arango; Celeny Ortiz; Daniel O. Maldonado-Perez; Diego A. Álvarez-Díaz; Hector Alejandro Ruiz-Moreno; Idabely Betancur Ortiz; Jorge E. Osorio; Juan P. Hernandez-Ortiz; Karl A Ciuoderis; Katherine Laiton-Donato; Laura Silvana Perez; Lina M. Hurtado; Marcela Mercado-Reyes; Maria Angélica Maya; Maria Stella López; Rita Almanza Payares; Sandra Ines Cano; Simón Villegas Velásquez
EPI_ISL_3945527	Instituto Nacional de Ciencias Médicas y Nutrición Salvador Zubirán (INCMNSZ)	Instituto Nacional de Medicina Genomica	Abraham Campos-Romero; Blancas S; Cedro-Tanda A; Cisneros-Villanueva M; Escobar-Arrazola MA; Herrera-Montalvo LA.; Hidalgo-Miranda A; Luna-Ruiz Marco; Mendoza-Vargas A; Moreno-Camacho José Luis; Ramirez-Vega O; Rangel-DeLeon D; Reyes-Grajeda JP; Rodriguez-Gallegos Jorge; Yair Alfaro-Mora
EPI_ISL_3982798	Instituto Nacional de Enfermedades Respiratorias (INER)	Centro de Investigación en Enfermedades Infecciosas (CIENI), Instituto Nacional de Enfermedades Respiratorias (INER)	Alejandra García-Gasca; Alejandra Hernández-Terán; Alejandro Sánchez-Flores; Alfredo Herrera-Estrella; Alicia Ocaña-Mondragón; Andreu Comas-García; Angel Gustavo Salas-Lais; Antonio Loza Román; Bernardo Martínez-Miguel; Blanca Taboada; Brenda Irasema Maldonado-Meza; Bruno Gómez-Gil; Carla Ivón Herrera-Najera; Carlos F. Arias; Celia Boukadida; Clara Esperanza Santacruz-Tinoco; Concepción Grajales-Muñiz; Consorcio Mexicano de Vigilancia Genómica (CoViGen-Mex). Authors (in alphabetical order): Julio Elias Alvarado-Yaah; Cristóbal Cháidez-Quiróz; Célida Duque Molina; Célida Martínez-Rodríguez; Daniel Fregoso-Rueda; Daniel Lira Morales; Eduardo Becerril-Vargas; Eduardo Rivera-Martínez; Fernando Fontove-Herrera; Fidencio Mejía-Nepomuceno; Francisco Pulido; Gabriel Chavira-Trujillo; Gloria Elena Espinosa-Ayala; Gloria María Molina-Salinas; Gloria Vazquez; Hector Montoya-Fuentes; Helen Haydee Fernanda Ramírez-Plascencia; Irvin González-López; Jean Pierre González; Jesús Hernández; Joel Armando Vázquez-Pérez).; Jorge Salas-Hernández; José Antonio Enciso-Moreno; José Arturo Martínez-Orozco; José Esteban Muñoz-Medina; José de Jesús Nuñez-Contreras; Juan Bautista Chale-Dzul; Julissa Enciso-Ibarra; Kathia Elizabeth Tapia-Diaz; Luis Alberto Ochoa-Carrera; Margarita Matías-Florentino; Mario Mújica-Sánchez; Marissa Perez-Garcia; María Eugenia Jiménez-Corona; María Guadalupe Santiago-Mauricio; María Guadalupe de Jesús Mireles-Rivera; Nelly Sélem-Mojica; Pavel Isa; Ricardo Ciria Merce; Ricardo Grande; Rosa María Gutiérrez Rios; Rosario Vazquez-Larios; Santiago Ávila-Ríos; Selene Zárate; Susana Lopez; Verónica Mata-Haro; Victor Eduardo García-Arias; Víctor Hugo Borja-Aburto
EPI_ISL_4071007, EPI_ISL_4107171	Instituto Nacional de Medicina Genomica	Instituto Nacional de Medicina Genomica	Blancas S; Cedro-Tanda A; Cisneros-Villanueva M; Escobar-Arrazola MA; Herrera-Montalvo LA.; Hidalgo-Miranda A; Mendoza-Vargas A; Ramirez-Vega O; Rangel-DeLeon D; Reyes-Grajeda JP; Yair Alfaro-Mora
EPI_ISL_3827499, EPI_ISL_3827558, EPI_ISL_3827767	Instituto de Medicina Tropical & Salud Global, Universidad Iberoamericana (UNIBE)	Instituto de Medicina Tropical & Salud Global, Universidad Iberoamericana (UNIBE)	Alejandro; Calderon; Paulino-Ramirez; Robert; Vallejo Degaudenzi; Victor V.
EPI_ISL_3978876	Integrated Covid Hub North East	Wellcome Sanger Institute for the COVID-19 Genomics UK (COG-UK) Consortium	Cordelia Langford; David K. Jackson; Dominic Kwiatkowski; Ewan Harrison; Ian Johnston; Integrated Covid Hub North East and Alex Alderton; Jeffrey Barrett; John Sillitoe on behalf of the Wellcome Sanger Institute COVID-19 Surveillance Team; Roberto Amato; Sonia Goncalves
EPI_ISL_4007284, EPI_ISL_4008105, EPI_ISL_4008108, EPI_ISL_4348705, EPI_ISL_4348711, EPI_ISL_4354278, EPI_ISL_4358318, EPI_ISL_4365206			
see above	KU Leuven, Rega Institute, Clinical and	KU Leuven, Rega Institute, Clinical and	Bert Vanmechelen; Joan Marti-Carerras; Piet Maes; Tony Wawina-Bokalanga

	Epidemiological Virology	Epidemiological Virology	
EPI_ISL_3921333, EPI_ISL_3921335, EPI_ISL_3921336, EPI_ISL_3921341, EPI_ISL_3921343, EPI_ISL_3921357, EPI_ISL_3921365, EPI_ISL_3921376, EPI_ISL_3921379, EPI_ISL_3921386, EPI_ISL_3921389, EPI_ISL_3921391, EPI_ISL_3921395			
see above	LDSP SAN ANDRES ISLA	Instituto Nacional de Salud- Dirección de Investigación en Salud Pública	Carlos Franco-Muñoz; Carmen Osorio; Diana Malo; Diego A. Álvarez-Díaz; Diego Andrés Prada; Gerardo Santamaría; Hector Alejandro Ruiz-Moreno; Jhonnatan Reales-González; Jorge Rivera; Juan Camilo Martinez; Julian Naizaque; Katherine Laiton-Donato; Lisseth Pardo; Magdalena Wiesner; Marcela Mercado-Reyes; Maria T. Herrera-Sepúlveda; Marta Lopez Blanco; Martha Lucia Ospina Martinez; Paola Rojas-Estevez; Sergio Gomez; Sheryll Corchuelo; Ángela Alarcon Cruz
EPI_ISL_4199478, EPI_ISL_4199479	LESP Aguascalientes	Instituto de Diagnostico y Referencia Epidemiologicos (INDRE)	Abril Rodriguez-Maldonado; Ariadna Medina-Benitez; Claudia Wong-Arambula; Ernesto Ramirez-Gonzalez.; Gisela Barrera-Badillo; Irma Lopez-Martinez; Joaquin Quiroz-Mercado; Lucia Hernandez-Rivas; Maribel Gonzalez-Villa; Natividad Cruz-Ortiz; Sergio Rangel-Guerrero; Tatiana Nunez-Garcia; Vanessa Rivero-Arredondo
EPI_ISL_4299038	LESP Michoacan	Instituto de Diagnostico y Referencia Epidemiologicos (INDRE)	Abril Rodriguez-Maldonado; Ariadna Medina-Benitez; Claudia Wong-Arambula; Ernesto Ramirez-Gonzalez.; Gisela Barrera-Badillo; Irma Lopez-Martinez; Joaquin Quiroz-Mercado; Lucia Hernandez-Rivas; Maribel Gonzalez-Villa; Natividad Cruz-Ortiz; Sergio Rangel-Guerrero; Tatiana Nunez-Garcia; Vanessa Rivero-Arredondo
EPI_ISL_4199471	LESP Queretaro	Instituto de Diagnostico y Referencia Epidemiologicos (INDRE)	Abril Rodriguez-Maldonado; Ariadna Medina-Benitez; Claudia Wong-Arambula; Ernesto Ramirez-Gonzalez.; Gisela Barrera-Badillo; Irma Lopez-Martinez; Joaquin Quiroz-Mercado; Lucia Hernandez-Rivas; Maribel Gonzalez-Villa; Natividad Cruz-Ortiz; Sergio Rangel-Guerrero; Tatiana Nunez-Garcia; Vanessa Rivero-Arredondo
EPI_ISL_4196885, EPI_ISL_4196888, EPI_ISL_4196901, EPI_ISL_4212131, EPI_ISL_4212144, EPI_ISL_4212166	LSP DISTRITAL BOGOTA	Gencore - Universidad de los Andes	Alejandro Gomez; Cristian Barrera; Gabriela Ariza; Gabriela Delgado; Johana Hernandez; Luisa Sacristan; Marcela Guevara; Marcela Mercado; Silvia Restrepo
EPI_ISL_3868377	Lab voor klinische biologie	Lab voor klinische biologie	Bruno Verhasselt; Hannelore Hamerlinck; Marija Janevska
EPI_ISL_3998159	Lab. Virología y Genética Universidad Simón Bolívar	Laboratorio de Biologia Molecular, Universidad Cooperativa de Colombia, Santa Marta	Andrew S. Muñoz-Gamba; Antonio Acosta; Daniel B. Ramírez-Osorio; José A. Usme-Ciro; Paula A. Quintero-Cortés; Yesid Bello
EPI_ISL_3801473, EPI_ISL_3999861	Labo Analyses Med	National Reference Center for Viruses of Respiratory Infections, Institut Pasteur, Paris	Angela Brisebarre; Camille Capel; Christophe Malabat; Corinne Maufrais; Etienne Simon-Lorière; Frédéric Lemoine; Hub de Bioinformatique et Biostatistique; Julien Fumey; Louise Lefrançois; Marion Barbet; Maud Vanpeene; Méline Bizard; Slim El-Khiari; Sylvie Behillil; Sylvie Van der Werf; Vincent Enouf; Vincent Vieillefond
EPI_ISL_3947362	Labor Dr. Fenner und Kollegen	Heinrich Pette Institute, Leibniz Institute for Experimental Virology	Adam Grundhoff; Alexis Robitaille; Johannes Knobloch; Martin Aepfelbacher; Nicole Fischer; Thomas Günther
EPI_ISL_4028962, EPI_ISL_4028964, EPI_ISL_4028966, EPI_ISL_4028973, EPI_ISL_4028977, EPI_ISL_4028981	Laboratoire National de Santé Publique – LNSP	Genomics and Proteomics Departament, Gorgas Memorial Institute For Health Studies	Alexander A Martinez; Ambar Moreno; Claudia Estrada; Claudia Gonzalez V; César Roberto Conde Pereira; Jessica Gondola; Leyda Abrego; Marlene Castillo; Melissa Gaitan; Oris Chavarria
EPI_ISL_4006547	Laboratorio Central de Epidemiología (LCE)	Instituto de Biotecnología de la UNAM	; Alejandra García-Gasca; Alejandra Hernández-Terán; Alejandro Sánchez-Flores; Alfredo Herrera-Estrella; Alicia Ocaña-Mondragón; Andreu Comas-García; Angel Gustavo Salas-Lais; Antonio Loza Román; Bernardo Martínez-Miguel; Blanca Taboada; Brenda Irasema Maldonado-Meza; Bruno Gómez-Gil; Carla Ivón Herrera-Najera; Carlos F. Arias; Celia Boukadida; Clara Esperanza Santacruz-Tinoco; Concepción Grajales-Muñiz; Consorcio Mexicano de Vigilancia Genómica (CoViGen-Mex). Authors (in alphabetical order): Julio Elias Alvarado-Yaah; Cristóbal Cháidez-Quiróz; Célida Duque Molina; Célida Martínez- Rodríguez; Daniel Fregoso-Rueda; Daniel Lira Morales; Eduardo Becerril-Vargas; Fernando Fontove-Herrera; Fidencio Mejía-Nepomuceno; Francisco Pulido; Gloria Elena Espinosa-Ayala; Gloria María Molina-Salinas; Gloria Vazquez; Hector Esteban Paz-Juárez; Hector Montoya-Fuentes; Helen Haydee Fernanda Ramírez-Plascencia; Irvin González-López; Jean Pierre González; Jesús Hernández; Joel Armando Vázquez-Pérez).; Jorge Salas-Hernández; José Antonio Enciso-Moreno; José Arturo Martínez-Orozco; José Esteban Muñoz-Medina; José de Jesús Nuñez-Contreras; Juan Bautista Chale-Dzul; Julissa Enciso-Ibarra; Kathia Elizabeth Tapia-Diaz; Luis Alberto Ochoa-Carrera; Margarita Matías-Florentino; Mario Mújica-Sánchez; Marissa Perez-Garcia; María Guadalupe Santiago-Mauricio; María Guadalupe de Jesús Mireles-Rivera; Nelly Sélem-Mojica; Pavel Isa; Ricardo Ciria Merce; Ricardo Grande; Rosa María Gutiérrez Rios; Santiago Ávila-Ríos; Selene Zárate; Susana Lopez; Verónica Mata-Haro; Victor Eduardo García-Arias; Víctor Hugo Borja-Aburto
EPI_ISL_3671014, EPI_ISL_3671015	Laboratorio Centrolab	Universidad Nacional de Colombia - Laboratorio Genómico One Health	Andres F. Cardona-Rios; Carlos Franco-Muñoz; Carolina Muñoz-Arango; Celeny Ortiz; Daniel O. Maldonado-Perez; Diego A. Álvarez-Díaz; Hector Alejandro Ruiz-Moreno; Idabely Betancur Ortiz; Jorge E. Osorio; Juan P. Hernandez-Ortiz; Karl A Ciuoderis; Katherine Laiton-Donato; Laura Silvana Perez; Lina M. Hurtado; Marcela Mercado-Reyes; Maria Angélica Maya; Maria Stella López; Rita Almanza Payares; Sandra Ines Cano; Simón Villegas Velásquez
EPI_ISL_4006785	Laboratorio Clínico Citisalud	Corporación para Investigaciones Biológicas-CIB	Jeanneth Mosquera Rendon; Katterine Molina Hoyos; Marcela Mercado Reyes; Uriel A. Hurtado Paez
EPI_ISL_3998147, EPI_ISL_3998151	Laboratorio Clínico Cristiam Gram	Laboratorio de Biologia Molecular, Universidad Cooperativa de Colombia, Santa Marta	Andrew S. Muñoz-Gamba; Daniel B. Ramírez-Osorio; Gloria A. Cuello; José A. Usme-Ciro; Katty Mindiola; Paula A. Quintero-Cortés; Yulieth L. Quiroz
EPI_ISL_4297520	Laboratorio Clínico Nancy Florez García	Centro de Genética y Biología Molecular - Universidad del Magdalena	Andrea M. Ramírez Hernandez; Angel Oviedo Marquez; Daniel Bautista; Edison Lea-Ch; Lyda R. Castro; Maria Teresa Mojica-Ortiz
EPI_ISL_4297539, EPI_ISL_4297546	Laboratorio Cristian Gram	Centro de Genética y Biología Molecular - Universidad del Magdalena	Andrea M. Ramírez Hernandez; Angel Oviedo Marquez; Daniel Bautista; Edison Lea-Ch; Lyda R. Castro; Maria Teresa Mojica-Ortiz
EPI_ISL_4219522, EPI_ISL_4219540, EPI_ISL_4219545	Laboratorio IMAT Instituto médico de alta tecnología S.A.S	Molecular Genetics and Antimicrobial Resistance - UGRA, Universidad El Bosque	Catalina Espitia; Jinnethe Reyes; Lorena Diaz; Marcela Mercado; Mauricio Pacheco; Rafael Rios; Valentina Martinez
EPI_ISL_3671008, EPI_ISL_3671051	Laboratorio Las Americas	Universidad Nacional de Colombia - Laboratorio Genómico One Health	Andres F. Cardona-Rios; Carlos Franco-Muñoz; Carolina Muñoz-Arango; Celeny Ortiz; Daniel O. Maldonado-Perez; Diego A. Álvarez-Díaz; Hector Alejandro Ruiz-Moreno; Idabely Betancur Ortiz; Jorge E. Osorio; Juan P. Hernandez-Ortiz; Karl A Ciuoderis; Katherine Laiton-Donato; Laura Silvana Perez; Lina M. Hurtado; Marcela Mercado-Reyes; Maria Angélica Maya; Maria Stella López; Rita Almanza Payares; Sandra Ines Cano; Simón Villegas Velásquez
EPI_ISL_3998114	Laboratorio Lorena Vejarano sede Barranquilla	Laboratorio de Biologia Molecular, Universidad Cooperativa de Colombia, Santa Marta	Andrew S. Muñoz-Gamba; Daniel B. Ramírez-Osorio; Danis Lora; José A. Usme-Ciro; Lissette M. Lopez; Paula A. Quintero-Cortés
EPI_ISL_4297483, EPI_ISL_4297485	Laboratorio Masvida de la Costa	Centro de Genética y Biología Molecular - Universidad del Magdalena	Andrea M. Ramírez Hernandez; Angel Oviedo Marquez; Daniel Bautista; Edison Lea-Ch; Lyda R. Castro; Maria Teresa Mojica-Ortiz
EPI_ISL_4220349, EPI_ISL_4220352, EPI_ISL_4220353, EPI_ISL_4220354, EPI_ISL_4220355, EPI_ISL_4220363, EPI_ISL_4220369, EPI_ISL_4220372, EPI_ISL_4220377, EPI_ISL_4220378, EPI_ISL_4220379			
see above	Laboratorio Nacional de Salud Pública Dr. Defilló - LNSPDD	Laboratory of Respiratory Viruses and Measles, Oswaldo Cruz Institute, FIOCRUZ	; Alice Sampaio Rocha; Ana Beatriz Machado Lima; Ana Carolina Mendonca; Anna Carolina Paixao; Elisa Cavalcante Pereira; Fernando Motta; Grey Benoit Vasquez; Isaac Miguel Sanchez; Ivonne Imbert; Lucia de la Cruz; Luciana Appolinario; Luiz Fernando Lopez Tort; Marilda Siqueira on behalf of the Fiocruz COVID-19 Genomic Surveillance Network; Mia Ferreira de Araujo; Natália Valente da Silva; Nurys de Castro; Paola Resende; Renata Serrano Lopes; Ronald Skewes; Taina Venas; Thayssa Keren Santos da Silva Neves
EPI_ISL_4029328, EPI_ISL_4029329	Laboratorio Nacional de Salud, Ministerio de Salud Publica y Asistencia Social	Genomics and Proteomics Departament, Gorgas Memorial Institute For Health Studies	Alexander A Martinez; Ambar Moreno; Claudia Estrada; Claudia Gonzalez V; César Roberto Conde Pereira; Jessica Gondola; Leyda Abrego; Marlene Castillo; Melissa Gaitan; Oris Chavarria
EPI_ISL_4297505	Laboratorio Rey-Fals	Centro de Genética y Biología Molecular - Universidad del Magdalena	Andrea M. Ramírez Hernandez; Angel Oviedo Marquez; Daniel Bautista; Edison Lea-Ch; Lyda R. Castro; Maria Teresa Mojica-Ortiz
EPI_ISL_3769570, EPI_ISL_3769571, EPI_ISL_3769572, EPI_ISL_3769573, EPI_ISL_3769576, EPI_ISL_3769577, EPI_ISL_3769581, EPI_ISL_3998033, EPI_ISL_3998034, EPI_ISL_3998035, EPI_ISL_3998038, EPI_ISL_3998180, EPI_ISL_3998184, EPI_ISL_3998195, EPI_ISL_3998199, EPI_ISL_3998201, EPI_ISL_3998202, EPI_ISL_4148466, EPI_ISL_4148467, EPI_ISL_4148476, EPI_ISL_4148484, EPI_ISL_4148485, EPI_ISL_4198084, EPI_ISL_4198096, EPI_ISL_4198098			
see above	Laboratorio de Referencia Nacional de	Laboratorio de Referencia Nacional de	Carlos Padilla Rojas; Henri Bailon Calderon; Iris Silva Molina; Joseph Huayra Niquen; Lely Solari Zerpa; Luis Barcena Flores; Marco Galarza Perez; Nancy Rojas Serrano; Nieves Sevilla Castañeda; Omar Caceres Rey; Orson Mestanza Millones; Princesa Medrano Alhuay; Priscila Lope Pari; Sandra Morales Ruiz; Sara Gordillo Vilchez; Steve Acedo Lazo; Veronica Hurtado Vela; Victor Jimenez Vasquez; Wendy Lizarraga Olivares

	Virus Respiratorios. Centro Nacional de Salud Publica. Instituto Nacional de Salud Peru.	Virus Respiratorios. Centro Nacional de Salud Publica. Instituto Nacional de Salud Peru.	
EPI_ISL_3667219	Laboratorio de Virología HUCA	Laboratorio de Virología HUCA	; Alba L; Alvarez-Arguelles ME; Boga JA; Costales I; Coto E; González-Alba JM; Gómez de Oña J; Martín-Rodríguez G; Melón S; Perez-Martínez Z; Rojo S; Sandoval M
EPI_ISL_4196918, EPI_ISL_4197212, EPI_ISL_4212167, EPI_ISL_4212171	Laboratorio de la Clínica Los Nogales	Gencore - Universidad de los Andes	Cristian Barrera; David González; Gabriela Ariza; Luisa Sacristan; Marcela Guevara; Marcela Mercado; Silvia Restrepo
EPI_ISL_3684751, EPI_ISL_3684971, EPI_ISL_3686276, EPI_ISL_3686480, EPI_ISL_3743419, EPI_ISL_3745925, EPI_ISL_3746461, EPI_ISL_4149236, EPI_ISL_4173053, EPI_ISL_4173473, EPI_ISL_4178156, EPI_ISL_4180800, EPI_ISL_4335425			
see above	Laboratory Corporation of America	Centers for Disease Control and Prevention Division of Viral Diseases, Pathogen Discovery	Adrian Paskey; Amanda Douglas; Amanda Suchanek; Andrea Throop; Ayla Burns; Benjamin Rambo-Martin; Bobbi Croy; Brian Krueger; Brian Norvell; Christopher Gulvick; Christos Petropoulos; Clinton Paden; Craig Lukasik; Dakota Howard; Darlene Wagner; Debbie Boles; Dhwani Batra; Duncan MacCannell; Eyad Almasri; Goran Stevovic; Howard Engler; Hrushikesh Deshmukh; Jake Humphrey; Jana Schroth; Jason Caravas; Joe Voshell; John Pruitt; Jonathan Meltzer; Jonathan Williams; Kara Moser; Kimberly Wagner; Kristine Lacek; Lax Iyer; Lisa Pfefferle; Lyndon Tilson; Manoj Jain; Marcia Eisenberg; Mary Cristobal; Mary Williamson; Matthew Robinson; Matthew Schmerer; Michael Levandoski; Mike Sapeta; Mindy Nye; Minoo Agarwal; Mohan Kolli; Nuthawin Charoensri; Oren Cohen; Peter Cook; Prashant Gupta; Qian Zeng; Rama Ghatti; Scott Parker; Scott Ryan; Scott Sammons; Shatavia Morrison; Stanley Letovsky; Steven Ragan; Suresh Selvaraju; Susan Countryman; Susan Hicks; Suzanne Dale; Thomas Urban; Tim Kuphal; Tricia Zwiefelhofer; Tymeckia Kendall; Victoria Caban Figueroa; Vincent Drouillon; Yvette Unoarumhi
EPI_ISL_4060322	MD DOH Laboratories Administration	Centers for Disease Control and Prevention Division of Viral Diseases, Pathogen Discovery	Alex Burgin; Ben Rambo-Martin; Clinton Paden; Dakota Howard; Dave Wentworth; Dhwani Batra; Jasmine Padilla; Justin Lee; Krista Queen; Kristen Knipe; Kristine Lacek; Mark Burroughs; Matthew Schmerer; Meghan Bentz; Mili Sheth; Peter Cook; Sam Shepard; Sarah Nobles; Suxiang Tong; Vivien Dugan; Yvette Unoarumhi
EPI_ISL_4083973	Maine Health and Environmental Testing Laboratory	Tewhey Lab, The Jackson Laboratory	Barter, M.; Dewey, H.; H. and Tewhey, R.; Iosue, F.; Lynch, R.; Matluk, N.; Munger
EPI_ISL_4167559, EPI_ISL_4347742, EPI_ISL_4347822, EPI_ISL_4348028, EPI_ISL_4348090, EPI_ISL_4348107, EPI_ISL_4348124			
see above	Mako Medical	Centers for Disease Control and Prevention Division of Viral Diseases, Pathogen Discovery	Adrian Paskey; Benjamin Rambo-Martin; Christopher Gulvick; Clinton Paden; Dakota Howard; Darlene Wagner; Dhwani Batra; Duncan MacCannell; Erisa Sula; Jason Caravas; Kara Moser; Kristine Lacek; Lauren Moon; Matthew Schmerer; Matthew Tugwell; Peter Cook; Scott Sammons; Shatavia Morrison; Tymeckia Kendall; Victoria Caban Figueroa; Yvette Unoarumhi
EPI_ISL_4178770	Maryland Genomics, Institute for Genome Sciences, University of Maryland School of Medicine	Maryland Genomics, Institute for Genome Sciences, University of Maryland School of Medicine	Claire M; Fraser; Hazen; Holly; Humphrys; Ivette; Jacques; Jonathan; Kranthi; Lim; Lisa D; Luke J; Mike; Ott; Ravel; Roussey; Sadzewicz; Sandra; Santana-Cruz; Tallon; Tracy; Vavikolanu
EPI_ISL_4213081	Michael E. DeBakery VA Medical Center	Genomics and Discovery, Respiratory Viruses Branch, Division of Viral Diseases, Centers for Disease Control and Prevention	Adam Retchless; Anna Kelleher; Anna Uehara; Brian Lynch; Clinton R. Paden; Dhwani Batra; Haibin Wang; Han Jia Justin Ng; Jasmine Padilla; Jing Zhang; Justin Lee; Mark Burroughs; Mili Sheth; Morgan Davis; Peter Cook; Rachel Marine; Sarah Nobles; Suxiang Tong; Tara Coalter; Yan Li; Ying Tao
EPI_ISL_3771045	Microbiologia CATLAB	Can Ruti SARS-CoV-2 Sequencing Hub (HUGTiP/IrsiCaixa/IGTP)	Alba Sánchez; Alexia París; Anna Not; Antoni E Bordoy; Bonaventura Clotet; Cristina Casañ; David Panisello; Francesc Catala-Moll; Gemma Clara; Ignacio Blanco; Laia Soler; Lauro Sumoy; Marc Noguera-Julian; Maria Casadellà; Mariona Parera; Mercedes Guerrero; Montserrat Giménez; Pere-Joan Cardona; Pilar Armengol; Roger Paredes; Verónica Saludes; and Elisa Martró on behalf of the Can Ruti SARS-CoV-2 Sequencing Hub
EPI_ISL_3761813, EPI_ISL_4137465	Microbiology Department, University Hospital Donostia	Microbiology Department, University Hospital Donostia	Cilla G.; Gomez M; Marimon JM; Martin-Peñaranda T; Montes M; Piñeiro L; Sorarrain A
EPI_ISL_4185684	Microbiology Department. Complexo Hospitalario Universitario de Vigo	Microbiology Department. Complexo Hospitalario Universitario de Vigo	Alvarez M; Cabrera JJ; Carballo R; Cores O; Cortizo S; Davina C; Martinez L; Mediero G; Pena I; Perez S; Potel C; Regueiro B; Rey S; Vasallo FJ; del-Campo V
EPI_ISL_3914609	Montana Public Health Laboratory	Montana Public Health Laboratory	Carrie Biskupiak; Deborah Gibson; Joy Ritter; Michael Dills; Michelle Mozer
EPI_ISL_4060407	NYC Department of Health and Mental Hygiene	Centers for Disease Control and Prevention Division of Viral Diseases, Pathogen Discovery	Alex Burgin; Ben Rambo-Martin; Clinton Paden; Dakota Howard; Dave Wentworth; Dhwani Batra; Jasmine Padilla; Justin Lee; Krista Queen; Kristen Knipe; Kristine Lacek; Mark Burroughs; Matthew Schmerer; Meghan Bentz; Mili Sheth; Peter Cook; Sam Shepard; Sarah Nobles; Suxiang Tong; Vivien Dugan; Yvette Unoarumhi
EPI_ISL_3845461	Naval Infectious Diseases Diagnostic Laboratory	Naval Medical Research Center Biological Defense Research Directorate	Andrea E. Luquette; Andrew J. Bennett; Catherine E. Arnold; Francisco Malagon; Gregory K. Rice; Kimberly A. Bishop-Lilly; Kyle A. Long; Lindsay A. Glang; Logan J. Voegtly; Megan A. Schilling; Michael V. Deschenes; Regina Z. Cer; Robin H. Miller; Victor A. Sugiharto
EPI_ISL_3825164	Nevada State Public Health Laboratory	Nevada State Public Health Laboratory	Andrew Gorzalski; Mark Pandori
EPI_ISL_4221926	Orange County Public Health Laboratory	Fulgent Genetics	Becky Tsai; Benafsh Sapra; Doreen Ng; Harry Gao; James Xie; John Gao; Joseph Fierro; Mickey Li; Yan Meng
EPI_ISL_3916157	Oregon State Public Health Laboratory	Oregon State Public Health Laboratory	Eugene Yeboah; John Fontana and Shane Sevey; Laura Tsaknaridis; Rafia Razzaque; Vanda Makris
EPI_ISL_3933263, EPI_ISL_3933391, EPI_ISL_4096771	Pandemic Response Lab - NYC	Pandemic Response Lab, R&D	Cybill del Castillo; Dylan Law; Haiping Hao; Henry Lee; Isabel Fernandez Escapa; Jon Laurent; Katharine Nelson; Melissa Hopkins; Michael Hammerling; Pradeep Bugga; Shinyoung Clair Kang; Sol Rey; William Ward
EPI_ISL_4182870	Public Health Ontario Laboratory	Public Health Ontario Laboratory	Aimin Li; Alireza Eshaghi; Andre Villegas; Ashleigh Sullivan; Christine Frantz; Dean Maxwell; Esha Joshi; Jared Simpson; Jennifer L Guthrie; Jonathan B Gubbay; Karthikeyan Sivaraman; Lawrence Heisler; Matthew Watson; Michael CY Li; Michael Laszloffy; Nahuel Fittipaldi; Philip Banh; Richard de Borja; Samir N Patel; Sandeep Nagra; Sandra Zittermann; Sarah Teatero; Vanessa G Allen; Yao Chen; Yogi Sundaravadanam
EPI_ISL_4366811, EPI_ISL_4368810, EPI_ISL_4368977, EPI_ISL_4369780, EPI_ISL_4371143, EPI_ISL_4371445	Quest Diagnostics Incorporated	Centers for Disease Control and Prevention Division of Viral Diseases, Pathogen Discovery	A. Gerasimova; A. Perez; B. Anderson; Benjamin Rambo-Martin; Christopher Gulvick; Clinton Paden; Dakota Howard; Dhwani Batra; Duncan MacCannell; Erisa Sula; F. Lacbawan; I. Shlyakhter; Jason Caravas; K. Livingston; Kristine Lacek; L. Bernstein; M. Hua; Matthew Schmerer; P. Tanpaiboon; Peter Cook; R. Kagan; R. Owen; R. Rolando; S. Rosenthal; Scott Sammons; Shatavia Morrison; Tymeckia Kendall; Victoria Caban Figueroa; Y. Liu; Yvette Unoarumhi
EPI_ISL_4060244	Regional Hospital Liberec	Regional Hospital Liberec	Iva Dolinova; Katerina Arientova; Katerina Stillerova; Martin Kracik; Tomas Zajic
EPI_ISL_3775827, EPI_ISL_3777185	Respiratory Virus Unit, Microbiology Services Colindale, Public Health England	COVID-19 Genomics UK (COG-UK) Consortium	PHE Covid Sequencing Team
EPI_ISL_3881334, EPI_ISL_3881812, EPI_ISL_3881832, EPI_ISL_3881841	SARS-CoV-2 Sequencing Castilla y Leon-Spain Consortium	SARS-CoV-2 Sequencing Castilla y Leon-Spain Consortium	Antonio Orduña-Domingo; Carlos Fuster Foz; Carmen Aldea-Mansilla; Carmen Gimeno Crespo; David Abad; Gabriel March Rosello; Gregoria Megías Lobón; Jose María Eiros Bouza; M. Isabel Fernandez-Natal; Marta Dominguez-Gil; Marta Hernandez; María Antonia García Castro; Mª Fe Brezmes-Valdivieso; Noelia Arenal Andrés; Silvia Rojo; Sonsoles Garcinuño Pérez
EPI_ISL_3691311	SYNLAB	GIGA Medical Genomics	Bouchra Boujemla; Claire Gourzonès; Cécile Meex; Keith Durkin; Laurent Gillet; Maria Artesi; Marie-Pierre Hayette; Nadine Cambisano; Nathalie Renotte; Olivier Ek; Sébastien Bontems; Vincent Bours
EPI_ISL_4071008, EPI_ISL_4071009, EPI_ISL_4199632	Salud Digna	Instituto Nacional de Medicina Genomica	Abraham Campos-Romero; Blancas S; Cedro-Tanda A; Cisneros-Villanueva M; Cruz-Islas Jazmin; Escobar-Arrazola MA; Garnica-Lopez Dora; Herrera-Montalvo LA.; Hidalgo-Miranda A; Luna-Ruiz Marco; Mendoza-Vargas A; Moreno-Camacho José Luis; Ramirez-Vega O; Rangel-DeLeon D; Reyes-Grajeda JP; Rodriguez-Gallegos Jorge; Yair Alfaro-Mora
EPI_ISL_3913027	Scripps Medical Laboratory	Andersen lab at Scripps Research	Ellen Stefanski; Ian Mchardy; SEARCH Alliance San Diego with Michael Quigley
EPI_ISL_4237131	Stanford Health Care	Stanford University School of Medicine, Clinical Virology Laboratory	Becky Jiang; James Zehnder; Malaya K. Sahoo; Selamawit Bihon; and Benjamin A. Pinsky
EPI_ISL_3792632	Swedish national genomic surveillance	The Public Health Agency of Sweden	Alma Brolund; Maria Lind Karlberg; Maximilian Riess; Swedish national genomic surveillance program of SARS-CoV-2

	program of SARS-CoV-2		
EPI_ISL_4060851	TXDSHS	TXDSHS	Anita Pokharel; Bonnie Oh; Chun Wang; Grace Kubin; Jenny Zhang; Karen Bobier; Lorraine Rodriguez; Maliha Rahman; Mayela Pedrueza; Myong Koag; Rachel Lee; Rashmi Tuladhar
EPI_ISL_3673913, EPI_ISL_3673914, EPI_ISL_3673915, EPI_ISL_3673916, EPI_ISL_3673917, EPI_ISL_3673918, EPI_ISL_3673919, EPI_ISL_3673920, EPI_ISL_3673921, EPI_ISL_3673922, EPI_ISL_3673923, EPI_ISL_3673924, EPI_ISL_3673925, EPI_ISL_3673926, EPI_ISL_3673927, EPI_ISL_3673928, EPI_ISL_3673929, EPI_ISL_3673930, EPI_ISL_3673931, EPI_ISL_3673932, EPI_ISL_3673933, EPI_ISL_3688253			
see above	The Caribbean Public Health Agency	Carrington Lab, Department of Preclinical Sciences, Faculty of Medical Sciences, The University of the West Indies, St Augustine Campus	Anushka Ramjag; Arianne Brown-Jordan; Avery Hinds; Christine V. F. Carrington; Christopher Oura; Gabriel Escobar; Irad Potter; Nadia Ashwood; Nikita S. D. Sahadeo; Nuno Faria; Oliver Pybus; Risha Singh; Sarah Hill; SueMin Nathaniel; Vernie Ramkissoon
EPI_ISL_4169304	US Air Force School of Aerospace Medicine	US Air Force School of Aerospace Medicine	Anthony Fries; Carol Garrett; Clarise Starr; Deanna Muehleman; Elizabeth Macias; Jennifer Meyer; Kelsey Lanter; Sarah Purves; William Gruner
EPI_ISL_4084898	USC Clinical Lab	Los Angeles County Public Health Laboratories	P. Hemarajata et al.
EPI_ISL_3942173	UW Virology Lab	UW Virology Lab	Alexander Greninger; Hong Xie; Keith R Jerome; Maria Lukes; Meei-Li Huang; Nathan Breit; Patrick Mathias; Pavitra Roychoudhury; Ricardo Perez; Robert J. Livingston; Sean Ellis; Shah Mohamed Bakhash; Tien V. Nguyen
EPI_ISL_4297514	Universidad Simón Bolivar	Centro de Genética y Biología Molecular - Universidad del Magdalena	Andrea M. Ramírez Hernandez; Angel Oviedo Marquez; Daniel Bautista; Edison Lea-Ch; Lyda R. Castro; Maria Teresa Mojica-Ortiz
EPI_ISL_3721599	Universidad de Caldas	Universidad Nacional de Colombia - Laboratorio Genómico One Health	Andres F. Cardona-Rios; Carlos Franco-Muñoz; Carolina Muñoz-Arango; Celeny Ortiz; Daniel O. Maldonado-Perez; Diego A. Álvarez-Díaz; Hector Alejandro Ruiz-Moreno; Idabely Betancur Ortiz; Jorge E. Osorio; Juan P. Hernandez-Ortiz; Karl A Ciuoderis; Katherine Laiton-Donato; Laura Silvana Perez; Lina M. Hurtado; Marcela Mercado-Reyes; Maria Angélica Maya; Maria Stella López; Rita Almanza Payares; Sandra Ines Cano; Simón Villegas Velásquez
EPI_ISL_3671047	Universidad de Sucre	Universidad Nacional de Colombia - Laboratorio Genómico One Health	Andres F. Cardona-Rios; Carlos Franco-Muñoz; Carolina Muñoz-Arango; Celeny Ortiz; Daniel O. Maldonado-Perez; Diego A. Álvarez-Díaz; Hector Alejandro Ruiz-Moreno; Idabely Betancur Ortiz; Jorge E. Osorio; Juan P. Hernandez-Ortiz; Karl A Ciuoderis; Katherine Laiton-Donato; Laura Silvana Perez; Lina M. Hurtado; Marcela Mercado-Reyes; Maria Angélica Maya; Maria Stella López; Rita Almanza Payares; Sandra Ines Cano; Simón Villegas Velásquez
EPI_ISL_3998105, EPI_ISL_3998154	Universidad del Atlántico Laboratorio de Investigación en Biología Molecular	Laboratorio de Biologia Molecular, Universidad Cooperativa de Colombia, Santa Marta	Andrew S. Muñoz-Gamba; Daniel B. Ramírez-Osorio; José A. Usme-Ciro; Paula A. Quintero-Cortés; Roberto Garcia
EPI_ISL_3914569, EPI_ISL_3914571, EPI_ISL_3914572	Universidad del Valle, LDAB-Laboratorio de Diagnostico de Agentes Biologicos	Universidad del Valle	Programa Nacional de Caracterización Genómica de SARS-CoV-2
EPI_ISL_3981839, EPI_ISL_3981840	University of Liège COVID-19 testing center	GIGA Medical Genomics	Bouchra Boujemla; Claire Gourzonès; Cécile Meex; Keith Durkin; Laurent Gillet; Maria Artesi; Marie-Pierre Hayette; Nadine Cambisano; Nathalie Renotte; Olivier Ek; Sébastien Bontems; Vincent Bours
EPI_ISL_4348482	Usansolo-Galdakao University Hospital	Cruces University Hospital	Ana Belén de la Hoz; Ana Gual-de-Torrella; Izaskun Alejo-Cancho; Mikel Gallego
EPI_ISL_3743387, EPI_ISL_3745405, EPI_ISL_4029470, EPI_ISL_4029482, EPI_ISL_4029605, EPI_ISL_4208554, EPI_ISL_4208573			
see above	Utah Public Health Laboratory	Utah Public Health Laboratory	Erin L. Young; John Arnn; Kelly F. Oakeson; Olinto Linares-Perdomo; Pooja Gupta
EPI_ISL_3717248	Valais Hospital, Central Institute	Valais Hospital, Central Institute	Alexis Dumoulin; Cedric Howald; Deborah Penet; Henri Pegeot; Ioannis Xenarios; Keith Harshman; Lorenzo Cerutti; Melyssa Elies
EPI_ISL_3673830	Washington State Department of Health Public Health Laboratories	Washington State Department of Health Public Health Laboratories	Avi Singh; Darren Lucas; Denny Russell; Drew MacKellar; Geoff Melly; Hannah Gray; Joenice Gonzalez; JohnAric Peterson; Philip Dykema; Rebecca Cao; Vanessa De Los Santos
EPI_ISL_3742030	Wyoming Public Health Laboratory	Wyoming Public Health Laboratory	Ashley Norberg; Brian Dominguez; Cari Sloma; Channing Weber; Chayse Rowley; Elliot Thomasson; Jim Mildenberger; Marley Goetz; Robert Petit; Sam Britz; Taylor Fearing; and Rob Christensen
EPI_ISL_4197547, EPI_ISL_4197999	Yale Clinical Virology Lab	Grubaugh Lab - Yale School of Public Health	Anderson Brito; Annie Watkins; Chaney Kalinich; Chantal Vogels; Isabel Ott; Jessica Rothman; Joseph Fauver; Kendall Billig; Mallery Breban; Marie L. Landry; Mary Petrone; Nathan Grubaugh; Tara Alpert; Tobias Koch

We gratefully acknowledge the following Authors from the Originating laboratories responsible for obtaining the specimens, as well as the Submitting laboratories where the genome data were generated and shared via GISAID, on which this research is based.

All Submitters of data may be contacted directly via www.gisaid.org

Authors are sorted alphabetically.

Acknowledgement EPI_SET Identifier: EPI_SET_20220328zg

Accession ID	Originating Laboratory	Submitting Laboratory	Authors
EPI_ISL_5058060	"SYNLAB ANGEL DIAGNOSTICA"	"Laboratorio de biotecnología, Universidad Icesi"	"María I. Gutiérrez López; Adrián Camilo Rodríguez Ararat; Diana M. Florez Giraldo; Marcela Mercado; Maria F. Villegas Torres; Paola A. Caicedo Burbano"; Programa Nacional de Caracterización Genómica de SARS-CoV-2; Sara González Henao
EPI_ISL_5058034, EPI_ISL_5058035, EPI_ISL_5058069	"Unidad de Diagnóstico Hemato Oncológico - UDHO"	"Laboratorio de biotecnología, Universidad Icesi"	"María I. Gutiérrez López; Adrián Camilo Rodríguez Ararat; Diana M. Florez Giraldo; Marcela Mercado; Maria F. Villegas Torres; Paola A. Caicedo Burbano"; Programa Nacional de Caracterización Genómica de SARS-CoV-2; Sara González Henao
EPI_ISL_5797245	"Unidad de Diagnóstico Hemato Oncológico - UDHO"	Laboratorio de biotecnología, Universidad Icesi	"María I. Gutiérrez López; Adrián Camilo Rodríguez Ararat; Diana M. Florez Giraldo; Marcela Mercado; Maria F. Villegas Torres; Paola A. Caicedo Burbano"; Programa Nacional de Caracterización Genómica de SARS-CoV-2; Sara González Henao
EPI_ISL_4960541, EPI_ISL_4984496, EPI_ISL_4984513, EPI_ISL_5428495	ADILAB	Laboratorio Departamental de Salud Publica de Antioquia	Ana Victoria Valencia Duarte; Andres F. Cardona-Rios; Cristian Arbey Velarde Hoyos; Gloria Isabel Escobar; Idabely Betancur Ortiz; Juan P. Hernandez-Ortiz; Juan Pablo Isaza Agudelo; Maria Stella López
EPI_ISL_5914875	AREA DE SALUD BARVA (COOPESIBA)	Inciensa, Instituto Costarricense de Investigación y Enseñanza en Nutrición y Salud	Adriana Godínez; Claudio Soto-Garita; Estela Cordero; Francisco Duarte; Hebleen Porras; José Luis Vargas; Mariela Gutiérrez; Melany Calderón; Sofia Herrera & Angélica Espinoza Fontana
EPI_ISL_4659127	AUSTRAL-omics, UACh	AUSTRAL-omics, UACh	Andrea Silva; Carolina Encina; Cristian Molina; Daniela Plaza; Luis Guzmán; Suany Quesada
EPI_ISL_4850558, EPI_ISL_4850560, EPI_ISL_4850566, EPI_ISL_4850567	AYUDAS DIAGNOSTICAS SURA	Instituto Nacional de Salud-Dirección de Investigación en Salud Pública	Carlos Franco-Muñoz; Carmen Osorio; Diana Malo; Diego A. Álvarez-Díaz; Diego Andrés Prada; Gerardo Santamaría; Hector Alejandro Ruiz-Moreno; Jhonnatan Reales-González; Jorge Rivera; Juan Camilo Martinez; Julian Naizaque; Katherine Laiton-Donato; Lisseth Pardo; Magdalena Wiesner; Marcela Mercado-Reyes; Maria T. Herrera-Sepúlveda; Marta Lopez Blanco; Martha Lucia Ospina Martinez; Paola Rojas; Sergio Gomez; Sheryll Corchuelo; Ángela Alarcon Cruz
EPI_ISL_5803646	AYUDAS DIAGNOSTICAS SURA	Universidad Nacional de Colombia - Laboratorio Genómico One Health	Andres F. Cardona-Rios; Carlos Franco-Muñoz; Carolina Muñoz-Arango; Celeny Ortiz; Daniel O. Maldonado-Perez; Diego A. Álvarez-Díaz; Hector Alejandro Ruiz-Moreno; Idabely Betancur Ortiz; Jorge E. Osorio; Juan P. Hernandez-Ortiz; Karl A Ciuoderis; Katherine Laiton-Donato; Laura Silvana Perez; Lina M. Hurtado; Marcela Mercado-Reyes; Maria Angélica Maya; Maria Stella López; Rita Almanza Payares; Sandra Ines Cano; Simón Villegas Velásquez
EPI_ISL_4376769, EPI_ISL_5108824, EPI_ISL_5215412, EPI_ISL_5229610, EPI_ISL_5860239, EPI_ISL_6164570	Aegis Sciences Corporation	Centers for Disease Control and Prevention Division of Viral Diseases, Pathogen Discovery	Alec Vest; Benjamin Rambo-Martin; Christopher Gulvick; Clinton Paden; Cyndi Clark; Dakota Howard; Dhwani Batra; Dillon Nall; Duncan MacCannell; Erisa Sula; Ethan Sanders; Holly Houdeshell; Jason Caravas; Kristine Lacek; Matthew Hardison; Matthew Schmerer; Ola Kvalvaag; Patrick Campbell; Peter Cook; Rob Case; Scott Sammons; Shatavia Morrison; Shaun Westlund; Tymeckia Kendall; Victoria Caban Figueroa; Vikramsinha Ghorpade; Yvette Unoarumhi
EPI_ISL_4574480	Alaska State Virology Laboratory	Alaska State Virology Laboratory	Elva House; Jack Chen; Jacob Zidek; Lisa Smith; Ph.D.; Stephanie DeRonde
EPI_ISL_4960533	BIORREFERENCIA	Laboratorio Departamental de Salud Publica de Antioquia	Cristian Arbey Velarde Hoyos; Gloria Isabel Escobar; Idabely Betancur Ortiz; Juan P. Hernandez-Ortiz; Maria Stella López
EPI_ISL_4520797	Bienestar IPS	Universidad Nacional de Colombia - Laboratorio Genómico One Health	Andres F. Cardona-Rios; Carlos Franco-Muñoz; Carolina Muñoz-Arango; Celeny Ortiz; Daniel O. Maldonado-Perez; Diego A. Álvarez-Díaz; Hector Alejandro Ruiz-Moreno; Idabely Betancur Ortiz; Jorge E. Osorio; Juan P. Hernandez-Ortiz; Karl A Ciuoderis; Katherine Laiton-Donato; Laura Silvana Perez; Lina M. Hurtado; Marcela Mercado-Reyes; Maria Angélica Maya; Maria Stella López; Rita Almanza Payares; Sandra Ines Cano; Simón Villegas Velásquez
EPI_ISL_6572162	BioMoLab	Molecular Genetics Laboratory, Instituto de Investigaciones Químicas, Universidad Mayor de San Andrés	Aneth Vasquez Michel; Carmen Delgado Barrera; Oscar M. Rollano-Peñaloza; Sandra Miranda Sardon
EPI_ISL_5152759, EPI_ISL_5152792	CARVAJAL_LABORATORIO_IPS_SAS	UNAL-BOGOTA	Andrés Pinzón; Cristian Nicolás Rodríguez Pava; Emiliano Barreto Hernandez; Johana hernández Tolosa; Juan Germán Rodríguez Castillo; Lorena Alexandra Argoty Chamorro; Luis Fernando Cadavid; Marcela Castaño Rodríguez; Maria Andrea Angarita Rodriguez; Martha Isabel Murcia; María Irene Cerezo Cortes; Mishelle Cuello Mejia; Nicole Osorio Certuche
EPI_ISL_4509642, EPI_ISL_4510708, EPI_ISL_4511035, EPI_ISL_4511165, EPI_ISL_4680649, EPI_ISL_4959005, EPI_ISL_5015966, EPI_ISL_5016156, EPI_ISL_6175235, EPI_ISL_6175289			
see above	CDPH VBL	California Department of Public Health	Emily Smith on behalf of CDPH-COVIDNet
EPI_ISL_6124117	CHU Pontchaillou	CHU Pontchaillou	DE TAYRAC Marie; DENOUAL Florent; ETCHEVERRY Amandine; FEBREAU Christine; GALIBERT Marie Dominique; GROLHIER Claire; JAGLINE Steven; PRONIER Charlotte; QUENET Benjamin; SASSI Mohamed; THIBAULT Vincent
EPI_ISL_5071147, EPI_ISL_5071161, EPI_ISL_5071201, EPI_ISL_5072601	COLCAN	Instituto Nacional de Salud-Dirección de Investigación en Salud Pública	Carlos Franco-Muñoz; Carmen Osorio; Diana Malo; Diego A. Álvarez-Díaz; Diego Andrés Prada; Gerardo Santamaría; Hector Alejandro Ruiz-Moreno; Jhonnatan Reales-González; Jorge Rivera; Juan Camilo Martinez; Julian Naizaque; Katherine Laiton-Donato; Lisseth Pardo; Magdalena Wiesner; Marcela Mercado-Reyes; Maria T. Herrera-Sepúlveda; Marta Lopez Blanco; Martha Lucia Ospina Martinez; Paola Rojas-Estevez; Sergio Gomez; Sheryll Corchuelo; Ángela Alarcon Cruz
EPI_ISL_5054472, EPI_ISL_5054477	COLCAN - INS	Gencore - Universidad de los Andes	Cristian Barrera; David González; Gabriela Ariza; Luisa Sacristan; Marcela Guevara; Marcela Mercado; Silvia Restrepo
EPI_ISL_5071127	CORPORACION CLINICA PRIMAVERA DE VILLAVICENCIO	Instituto Nacional de Salud-Dirección de Investigación en Salud Pública	Carlos Franco-Muñoz; Carmen Osorio; Diana Malo; Diego A. Álvarez-Díaz; Diego Andrés Prada; Gerardo Santamaría; Hector Alejandro Ruiz-Moreno; Jhonnatan Reales-González; Jorge Rivera; Juan Camilo Martinez; Julian Naizaque; Katherine Laiton-Donato; Lisseth Pardo; Magdalena Wiesner; Marcela Mercado-Reyes; Maria T. Herrera-Sepúlveda; Marta Lopez Blanco; Martha Lucia Ospina Martinez; Paola Rojas-Estevez; Sergio Gomez; Sheryll Corchuelo; Ángela Alarcon Cruz
EPI_ISL_5152809	CPC-Synlab_Colombia	UNAL-BOGOTA	Andrés Pinzón; Cristian Nicolás Rodríguez Pava; Emiliano Barreto Hernandez; Johana hernández Tolosa; Juan Germán Rodríguez Castillo; Lorena Alexandra Argoty Chamorro; Luis Fernando Cadavid; Marcela Castaño Rodríguez; Maria Andrea Angarita Rodriguez; Martha Isabel Murcia; María Irene Cerezo Cortes; Mishelle Cuello Mejia; Nicole Osorio Certuche
EPI_ISL_5062464	Centre Hospitalier Universitaire de Rouen Laboratoire de Virologie	Centre Hospitalier Universitaire de Rouen Laboratoire de Virologie	Alice Moisan; Fabienne De Oliveira; Marie Leoz
EPI_ISL_4560893	Centro de Investigación Biomédica de La Rioja - Hospital San Pedro Logroño	SeqCOVID-SPAIN consortium/IBV(CSIC)	José Manuel Azcona Gutiérrez; María Pilar Bea Escudero; María de Toro; Miriam Blasco Alberdi and SeqCOVID-SPAIN consortium
EPI_ISL_5779882	Clinique Saint-Pierre Ottignies	UCLouvain/IREC/MBLG	Benoit Kabamba Mukadi; Bertrand Bearzatto; Jean Ruelle
EPI_ISL_5603315, EPI_ISL_5603329	Clínica Imbanaco	Laboratorio de Biología Molecular y Biotecnología - Universidad Tecnológica de Pereira	Augusto Zuluaga-Velez; Fredy A. Tabares-Villa; Juan C. Sepulveda; Juan D. Anacona-Montilla; Marcela Orjuela-Rodriguez
EPI_ISL_5264656	Colorado Department of Public Health and Environment	Colorado Department of Public Health and Environment	Alexandria Rossheim; Diana Ir; Emily A. Travanty; Laura Bankers; Mandy Waters; Michael Martin; Molly C. Hetherington-Rauth; Sarah Elizabeth Totten; Shannon R. Matzinger
EPI_ISL_5603780	Compensar Calle 26	LSPSDS	Alejandro Gomez Lopez; Gabriela Delgado Murcia; Johana Hernandez Toloza; Marcela Castano Rodriguez; Marcela Mercado
EPI_ISL_5522795	Curative Labs	Curative Labs	Elias L. Salfati; Eugenia Khorosheva; George Way; J.Cesar Ignacio-Espinoza; Janet Chen; Mikhail Hanewich-Hollatz; Nabjot Sandhu; Sophia Quasem; Vladimir Slepnev; Zhiyi Xie
EPI_ISL_4413569, EPI_ISL_4424795	DASA	DASA	Adriano Bonaldi; Angelica Hristov; Annelise Lopes; Bianca Cota; Cristina Oliveira; Jose Levi; Lidia Yamamoto; Paulo Pierry; Rodrigo Guarischi; Rodrigo Salazar
EPI_ISL_4891956	Diagnostyka. Laboratoria Medyczne.	1. ViroGenetics - BSL3 Laboratory of Virology, Małopolska Centre of Biotechnology, Jagiellonian University; 2. genXone SA, Research & Development Laboratory	Aleksandra Gidlewicz; Anna Brylak; Gromowski, T.; Grzegorz Nowicki; Jakub Grabowski; Kowalski, M.; Labaj; Maciej Sykulski; Mazur-Panasiuk, N.; Michał Kaszuba; Natalia Drweska-Matelska; P.P.; Pyrc, K.; Ruslan Herasymenko; Sylwia Januszczak; Szulc, P.; Wydmanski, W.; Łukasz Krych
EPI_ISL_5645353, EPI_ISL_5645484, EPI_ISL_5645515, EPI_ISL_5645516, EPI_ISL_5645519	Dirección Regional de Salud (DIRESA) Amazonas	Instituto de Enfermedades Tropicales - UNTRM	Alejandra Dávila Barclay; Carla Montenegro; Cecilia Pajuelo; Christian Campos; Diego Cuicapuza; Guillermo Salvatierra; Luis Rojas; Pablo Tsukayama; Pedro E. Romero; Rafael Tapia; Stella Chenet
EPI_ISL_4401998, EPI_ISL_4401999, EPI_ISL_4402000, EPI_ISL_4839317,	Dutch COVID-19 response team	National Institute for Public Health and the Environment (RIVM)	Adam Meijer; AnneMarie van den Brandt; Annelies Kroneman; Bas van der Veer; Chantal Reusken; Dennis Schmitz; Dirk Eggink; Euníce Then; Florian Zwagemaker; Harry Vennema; Ivo van Walle; Jeroen Cremer; Karim Hajji; Kim Freriks; Lisa Wijsman; Lynn Aarts; Ryanne Jaarsma; Sanne Bos; Sharon van den Brink; Stijn van Rossum; on behalf of the national COVID-19 response team

EPI_ISL_4846094			
EPI_ISL_6512558	ESCUELA DE MICROBIOLOGIA UNIVERSIDAD DE ANTIOQUIA	Laboratorio Departamental de Salud Publica de Antioquia	Ana Victoria Valencia Duarte; Cristian Arbey Velarde Hoyos; Gloria Isabel Escobar; Idabely Betancur Ortiz; Juan P. Hernandez-Ortiz; Juan Pablo Isaza Agudelo; Maria Stella López
EPI_ISL_5934856	Fimlab Laboratoriot Oy Tampere	Expert Microbiology, National Institute for Health and Welfare	Carita Savolainen-Kopra; Erika Lindh; Haider al-Hello; Jani Halkilahti; Kirsi Liitsola; Niina Ikonen; Olli Vapalahti; Pekka Ellonen; Phuoc Truong; Päivi Laurila; Ravi Kant; Sari Hannula; Soile Blomqvist; Teemu Smura
EPI_ISL_5541948, EPI_ISL_5541982, EPI_ISL_5541997, EPI_ISL_5542170, EPI_ISL_5866225, EPI_ISL_5925008, EPI_ISL_5925291, EPI_ISL_5925292, EPI_ISL_5925297, EPI_ISL_6367519			
see above	Florida Bureau of Public Health Laboratories	Florida Bureau of Public Health Laboratories	Jason Blanton; Namratha Tarigopula; Sarah Schmedes; Tiffany Splatt
EPI_ISL_4371757, EPI_ISL_4372077, EPI_ISL_5232587, EPI_ISL_5609058	Fulgent Genetics	Centers for Disease Control and Prevention Division of Viral Diseases, Pathogen Discovery	Becky Tsai; Benafsh Sapra; Benjamin Rambo-Martin; Christopher Gulvick; Clinton Paden; Dakota Howard; Dhwani Batra; Doreen Ng; Duncan MacCannell; Erisa Sula; Harry Gao; James Xie; Jason Caravas; John Gao; Joseph Fierro; Kristine Lacek; Matthew Schmerer; Mickey Li; Peter Cook; Scott Sammons; Shatavia Morrison; Tymeckia Kendall; Victoria Caban Figueroa; Yan Meng; Yvette Unoarumhi
EPI_ISL_5736039, EPI_ISL_5797256, EPI_ISL_5797260, EPI_ISL_5797266	Fundacion Valle del Lili	Laboratorio de biotecnología, Universidad Icesi	"María I. Gutiérrez López; Adrián Camilo Rodríguez Ararat; Diana M. Florez Giraldo; Marcela Mercado; Maria F. Villegas Torres; Paola A. Caicedo Burbano"; Programa Nacional de Caracterización Genómica de SARS-CoV-2; Sara González Henao
EPI_ISL_5365919	Fundación Cardioinfantil	Centro de Investigaciones en Microbiología y Biotecnología-UR (CIMBIUR), Facultad de Ciencias Naturales, Universidad del Rosario, Bogotá, Colombia	Angie Ramírez; Juan David Ramírez; Luz H. Patiño; Marcela Mercado-Reyes; Marina Muñoz; Nathalia Ballesteros; Nicolas Niño; Sergio Castañeda
EPI_ISL_4566164, EPI_ISL_4566175, EPI_ISL_4566198, EPI_ISL_4740144, EPI_ISL_4740247, EPI_ISL_4740255, EPI_ISL_4740265, EPI_ISL_4891921			
see above	Gencore - Universidad de los Andes	Gencore - Universidad de los Andes	Cristian Barrera; David González; Felipe Báez; Gabriela Ariza; Luisa Sacristan; Marcela Guevara; Marcela Mercado; Silvia Restrepo
EPI_ISL_4628790, EPI_ISL_4632314, EPI_ISL_4635740, EPI_ISL_4649141, EPI_ISL_4659826, EPI_ISL_5011668, EPI_ISL_5880326, EPI_ISL_6052412, EPI_ISL_6052417, EPI_ISL_6054318, EPI_ISL_6054328, EPI_ISL_6453513, EPI_ISL_6453958, EPI_ISL_6453978, EPI_ISL_6453989, EPI_ISL_6453993, EPI_ISL_6455293, EPI_ISL_6456070, EPI_ISL_6456092, EPI_ISL_6568026, EPI_ISL_6568041, EPI_ISL_6568043, EPI_ISL_6568047			
see above	Genetica Molecular and Subdepartamento de Virologia ISP Chile	Instituto de Salud Publica de Chile	Andres Castillo; Barbara Parra; Constanza Campano; Gisselle Barra; Javier Tognarelli; Jorge Fernandez; Karen Orostica; Loredana Arata; Patricia Bustos; Rodrigo Fasce; Soledad Ulloa
EPI_ISL_5196317, EPI_ISL_5196318, EPI_ISL_5196319, EPI_ISL_5196320, EPI_ISL_5196321, EPI_ISL_5196322, EPI_ISL_5196323, EPI_ISL_5196324, EPI_ISL_5196325, EPI_ISL_5196326, EPI_ISL_5196327, EPI_ISL_5196328, EPI_ISL_5196329, EPI_ISL_5196330, EPI_ISL_5196331			
see above	Gorgas Memorial Institute of Health Studies	Gorgas Memorial Institute of Health Studies	Castillo Jorge; Chen Maria; Franco Danilo; Gonzalez Claudia; Jessica Gondola; Leyda Abrego; Lopez-Verges Sandra; Marlenne Castillo; Martinez Alexander; Menacho Abdiel; Moreno Ambar; Moreno Brechla; Oris Chavarria; Ortiz Alma; Salazar Jacqueline
EPI_ISL_4744620	Gorgas Memorial Laboratory of Health Studies	Gorgas Memorial Laboratory of Health Studies	Castillo Jorge; Chen Maria; Franco Danilo; Gonzalez Claudia; Jessica Gondola; Leyda Abrego; Lopez-Verges Sandra; Marlenne Castillo; Martinez Alexander; Menacho Abdiel; Moreno Ambar; Moreno Brechla; Oris Chavarria; Ortiz Alma; Salazar Jacqueline
EPI_ISL_5315111	Gyncentrum	Gyncentrum	Adam Pudełko; Agnieszka Polak; Aleksandra Skubis-Sikora; Celina Kruszniewska-Rajs; Emilia Morawiec; Jolanta Bartosiewicz- Wąsik; Magdalena Samul; Maria Maklasińska-Majdanik; Paweł Czerwiński; Robert Wojtyczka; Tomasz Wąsik
EPI_ISL_5072527	HOSPITAL DEPARTAMENTAL DE VILLAVICENCIO	Instituto Nacional de Salud-Dirección de Investigación en Salud Pública	Carlos Franco-Muñoz; Carmen Osorio; Diana Malo; Diego A. Álvarez-Díaz; Diego Andrés Prada; Gerardo Santamaría; Hector Alejandro Ruiz-Moreno; Jhonnatan Reales-González; Jorge Rivera; Juan Camilo Martinez; Julian Naizaque; Katherine Laiton-Donato; Lisseth Pardo; Magdalena Wiesner; Marcela Mercado-Reyes; Maria T. Herrera-Sepúlveda; Marta Lopez Blanco; Martha Lucia Ospina Martinez; Paola Rojas-Estevez; Sergio Gomez; Sheryll Corchuelo; Ángela Alarcon Cruz
EPI_ISL_4659468, EPI_ISL_6226805	HOSPITAL UNIVERSITARIO 12 DE OCTUBRE	HOSPITAL UNIVERSITARIO 12 DE OCTUBRE	Carmen Martín-Higuera; Esther Viedma; Irene Múñoz-Gallego; M.ª Dolores Folgueira; Mar Aguilera; Noelia Moral; Rafael Delgado; Sagrario Zurita
EPI_ISL_5886781	HOSPITAL UNIVERSITARIO CENTRAL DE ASTURIAS	Laboratorio de Virología HUCA	; Alba L; Alvarez-Arguelles ME; Boga JA; Costales I; Coto E; González-Alba JM; Gómez de Oña J; Martín-Rodríguez G; Melón S; Perez-Martínez Z; Rojo S; Sandoval M
EPI_ISL_5263913	HOSPITAL UNIVERSITARIO VIRGEN DE LA ARRIXACA	Instituto de Salud Carlos III	A. Monzón; F. Casas; I. Jiménez; I.MORENO PARRADO; LAURA; M. Sandonís; P. Zaballos; S. Cuesta; S. Iglesias-Caballero; S. Pozo; S. Varona; V. Camarero; Vázquez-Morón
EPI_ISL_4960508, EPI_ISL_4984735	HPTU	Laboratorio Departamental de Salud Publica de Antioquia	Andres F. Cardona-Rios; Cristian Arbey Velarde Hoyos; Gloria Isabel Escobar; Idabely Betancur Ortiz; Juan P. Hernandez-Ortiz; Maria Stella López
EPI_ISL_5858260	Hackensack Medical Center	New York Genome Center	Amy Baldwin; Andre Corvelo; Barry Kreiswirth; David Perlin; Dayna M. Oschwald; Jose Mediavilla; Kaelea Composto; Kar Chow; Liang Chen; Marcus Cunningham; Michael Zody; Samantha Fennessey; Tom Maniatis
EPI_ISL_5522122, EPI_ISL_5659018, EPI_ISL_5947562, EPI_ISL_6479524, EPI_ISL_6481761, EPI_ISL_6484453, EPI_ISL_6485137, EPI_ISL_6485232			
see above	Helix	Centers for Disease Control and Prevention Division of Viral Diseases, Pathogen Discovery	Benjamin Rambo-Martin; Christopher Gulvick; Clinton Paden; Dakota Howard; Dhwani Batra; Duncan MacCannell; Erisa Sula; Helix CA; Jason Caravas; Kristine Lacek; Matthew Schmerer; Peter Cook; Scott Sammons; Shatavia Morrison; Tymeckia Kendall; Victoria Caban Figueroa; Yvette Unoarumhi
EPI_ISL_4962237	Helix/Illumina	Centers for Disease Control and Prevention Division of Viral Diseases, Pathogen Discovery	Adrian Paskey; Alexandre Bolze; Ary Ascencio; Benjamin Rambo-Martin; Brad Sickler; Charlotte Rivera-Garcia; Christopher Gulvick; Chrstine Tran; Clinton Paden; Dakota Howard; Darlene Wagner; David Becker; Dhwani Batra; Duncan MacCannell; Efren Sandoval; Eileen De Feo; Elizabeth Cirulli; Eric Allen; Geraint Levan; James Lu; Jan Antico; Jason Caravas; Jason Nguyen; Jimmy Ramirez; Jingtao Liu; Kara Moser; Kelly Barrett; Kim Gietzen; Kristine Lacek; Magnus Isaksson; Marc Laurent; Matthew Schmerer; Matthew Tolentino; Nicole Washington; Peter Cook; Phil Febbo; Ryan Cho; Scott Sammons; Shannon Wickline; Shatavia Morrison; Sherry Wang; Simon White; Tyler Cassens; William Lee; Yvette Unoarumhi
EPI_ISL_4946245	Hospital Civil Ipiales	Instituto Nacional de Salud-Dirección de Investigación en Salud Pública	Carlos Franco-Muñoz; Carmen Osorio; Diana Malo; Diego A. Álvarez-Díaz; Diego Andrés Prada; Gerardo Santamaría; Hector Alejandro Ruiz-Moreno; Jhonnatan Reales-González; Jorge Rivera; Juan Camilo Martinez; Julian Naizaque; Katherine Laiton-Donato; Lisseth Pardo; Magdalena Wiesner; Marcela Mercado-Reyes; Maria T. Herrera-Sepúlveda; Marta Lopez Blanco; Martha Lucia Ospina Martinez; Paola Rojas; Sergio Gomez; Sheryll Corchuelo; Ángela Alarcon Cruz
EPI_ISL_5587611, EPI_ISL_5587623	Hospital General Plaza de la Salud	Instituto de Medicina Tropical & Salud Global, Universidad Iberoamericana (UNIBE)	A; Anel; Ann; Calderon; Cuevas; Cuevas Lantigua; Gabriella; Gilda; Guzman Marte; Jabier; Maridania; Paula; Paulino-Ramirez; Rita.; Robert; Rojas Fermin; Sanchez Marmolejos; Tolari Jacobo; Vallejo Degaudenzi; Victor Virgilio
EPI_ISL_5587633, EPI_ISL_5587645, EPI_ISL_5587651, EPI_ISL_5621195, EPI_ISL_5621494, EPI_ISL_5621499, EPI_ISL_5621502, EPI_ISL_5687696, EPI_ISL_5687945			
see above	Hospital General Plaza de la Salud, Infectious Disease Department	Instituto de Medicina Tropical & Salud Global, Universidad Iberoamericana (UNIBE)	A; Anel; Ann; Calderon; Cuevas; Cuevas Lantigua; Gabriella; Gilda; Guzman Marte; Jabier; Maridania; Paula; Paulino-Ramirez; Rita.; Robert; Rojas Fermin; Sanchez Marmolejos; Tolari Jacobo; Vallejo Degaudenzi; Victor Virgilio
EPI_ISL_5587617	Hospital General Plaza de la Salud, Infectious Disease Department	Instituto de Medicina Tropical & Salud Global, Universidad Iberoamericana (UNIBE), Santo Domingo, 22333, Dominican Republic	A; Anel; Ann; Calderon; Cuevas; Cuevas Lantigua; Gabriella; Gilda; Guzman Marte; Jabier; Maridania; Paula; Paulino-Ramirez; Rita.; Robert; Rojas Fermin; Sanchez Marmolejos; Tolari Jacobo; Vallejo Degaudenzi; Victor Virgilio
EPI_ISL_4987001, EPI_ISL_4987029, EPI_ISL_6135444, EPI_ISL_6135499, EPI_ISL_6135500, EPI_ISL_6135501, EPI_ISL_6157320			
see above	Hospital General Universitario Gregorio Marañón	Hospital General Universitario Gregorio Marañón	Cristina Rodriguez-Grande; Darío García de Viedma; Jorge Rodríguez-Grande; Julia Suárez; Laura Pérez-Lago; Marta Herranz Martin; Patricia Muñoz; Pedro Sola Campoy; Pilar Catalán; Sergio Buenestado Serrano; Victor Manuel de la Cueva
EPI_ISL_4560905, EPI_ISL_4560920, EPI_ISL_4560973, EPI_ISL_4560982	Hospital General Universitario de Alicante - Instituto de Investigación Sanitaria y Biomédica de Alicante	SeqCOVID-SPAIN consortium/IBV(CSIC)	Carmen Molina Pardines and SeqCOVID-SPAIN consortium; Maripaz Ventero Martín
EPI_ISL_5926925	Hospital Herrera Llerandi	Asociación de Salud Integral / Clínica Familiar "Luis Ángel García"	Ana S. Gonzalez-Reiche; Claudia Rangel; Danicela Mercado; Eduardo Arathon; Hilda Ruiz; Luis Aguirre; Luis Rivas; Narda Medina; Oscar Bonilla; Osmar Gamboa.
EPI_ISL_4885353, EPI_ISL_4885355, EPI_ISL_4885356, EPI_ISL_6026625	Hospital Universitari Joan XXIII	Hospital Universitari Joan XXIII	Carla Martín; Clara Benavent; Cristina Gutiérrez; Ester Picó; Gemma Recio; Margarida Terrón
EPI_ISL_5340043, EPI_ISL_5340961, EPI_ISL_5341127, EPI_ISL_5341826, EPI_ISL_5344149, EPI_ISL_5375470	Houston Methodist Hospital	Houston Methodist Hospital	Ilya J. Finkelstein; James J. Davis; Jessica Cambric; Jimmy Gollihar; Kristina Reppond; Layne Pruitt; Madison N. Shyer; Marcus Nguyen; Matthew Ojeda Saavedra; Paul A. Christensen; Prasanti Yerramilli; Randall J. Olsen; Robert Olson; Ryan Gadd; S. Wesley Long; Sishir Subedi; and James M. Musser
EPI_ISL_4984530	ICMT APARTADO	Laboratorio Departamental de Salud Publica de Antioquia	Andres F. Cardona-Rios; Cristian Arbey Velarde Hoyos; Gloria Isabel Escobar; Idabely Betancur Ortiz; Juan P. Hernandez-Ortiz; Maria Stella López
EPI_ISL_5778137	INCMNSZ	Instituto Nacional de Medicina Genomica	Cedro-Tanda A; Cruz-Islas J; Escobar-Arrazola MA; Herrera-Montalvo LA.; Hidalgo-Miranda A; Mendoza-Vargas A; Ramirez-Vega O; Rangel-DeLeon D; Reyes-Grajeda JP; Yair Alfaro-Mora
EPI_ISL_5650473	INSIDE DIAGNOSTICOS	Instituto Butantan	Antonio Jorge Martins; Claudia Renata dos Santos Barros; David Schlesinger; Debora Botequio Moretti; Dimas Tadeu Covas; Elaine Cristina Marqueze; Elaine Vieira Santos; Evandra Strazza Rodrigues; Heidge Fukumasu; Jayme Augusto de Souza-Neto; José Salvatore Leister Patané; Luiz Alcantara; Luiz Lehmann

Coutinho; Maria Carolina Elias; Maurício Lacerda Nogueira; Rafael dos Santos Bezerra; Raul Machado Neto; Rejane Maria Tommasini Grotto; Ricardo Haddad; Sandra Coccuzzo Sampaio Vessoni; Simone Kashima; Svetoslav Nanev Slavov; Vincent Louis Viala

EPI_ISL_6129002, EPI_ISL_6129027, EPI_ISL_6208167, EPI_ISL_6208176, EPI_ISL_6208197, EPI_ISL_6208214, EPI_ISL_6208237, EPI_ISL_6208247, EPI_ISL_6208263, EPI_ISL_6208293, EPI_ISL_6208306, EPI_ISL_6208311, EPI_ISL_6208319, EPI_ISL_6208320, EPI_ISL_6507608, EPI_ISL_6507610, EPI_ISL_6507611, EPI_ISL_6507612, EPI_ISL_6507613, EPI_ISL_6507614, EPI_ISL_6507691, EPI_ISL_6526278, EPI_ISL_6526284, EPI_ISL_6526285, EPI_ISL_6569586, EPI_ISL_6569593, EPI_ISL_6569599, EPI_ISL_6569609, EPI_ISL_6569625, EPI_ISL_6569673

see above	INSPI-CRN DE INFLUENZA Y OTROS VIRUS RESPIRATORIOS	INSPI-CRN DE INFLUENZA Y OTROS VIRUS RESPIRATORIOS	Alfredo Bruno; Carlos Chiluiza; Daniel Ramos; Domenica de Mora; Fernando Llerena; Jimmy Garcés; Lizbeth Patiño; Marcela Mejia; Maria Angelica Becerra; Maritza Olmedo; Michelle Páez; Ruben Armas
EPI_ISL_4497025	INSPI-CRN DE INFLUENZA Y OTROS VIRUS RESPIRATORIOS	NIC-INSPI	Alfredo Bruno; Domenica de Mora.; Jimmy Garcés; Johanna Laines; Lizbeth Patiño; Manuel Gonzalez; Maritza Olmedo; Michelle Páez
EPI_ISL_4382923, EPI_ISL_4384702, EPI_ISL_4386123, EPI_ISL_4387287, EPI_ISL_6102799	Infinity Biologix	Centers for Disease Control and Prevention Division of Viral Diseases, Pathogen Discovery	Benjamin Rambo-Martin; Chirayu Goswami; Christian Bixby; Christopher Gulvick; Clinton Paden; Dakota Howard; Dhwani Batra; Duncan MacCannell; Erisa Sula; Jason Caravas; Jonathan Schultz; Kristine Lacek; Matthew Schmerer; Peter Cook; Robin Grimwood; Russ Hager; Scott Sammons; Shatavia Morrison; Tymeckia Kendall; Victoria Caban Figueroa; Yihe Wang; Yvette Unoarumhi
EPI_ISL_4740197, EPI_ISL_5094648, EPI_ISL_6368039	Institute of Microbiology, Universidad San Francisco de Quito	Institute of Microbiology, Universidad San Francisco de Quito	Belén Prado-Vivar; Bernardo Gutiérrez; Daniela Villalva; Erika B. Muñoz; Fernanda Zurita; Gabriel Trueba; Hugo Navarrete; Jaime Costales; Juan José Guadalupe; Mateo Carvajal; Michelle Grunauer; Monica Becerra-Wong; Nelson Montalvan; Patricio Rojas-Silva; Paúl Cárdenas; Sully Márquez; Verónica Barragán
EPI_ISL_5778127	Instituto Nacional de Medicina Genomica	Instituto Nacional de Medicina Genomica	Cedro-Tanda A; Cruz-Islas J; Escobar-Arrazola MA; Herrera-Montalvo LA.; Hidalgo-Miranda A; Mendoza-Vargas A; Ramirez-Vega O; Rangel-DeLeon D; Reyes-Grajeda JP; Yair Alfaro-Mora
EPI_ISL_5393528, EPI_ISL_5424306	KU Leuven, Rega Institute, Clinical and Epidemiological Virology	KU Leuven, Rega Institute, Clinical and Epidemiological Virology	Bert Vanmechelen; Joan Marti-Carerras; Piet Maes; Tony Wawina-Bokalanga
EPI_ISL_5421700	Kaiser Permanente NW Reginal Lab	OHSU MM Lab	Amber Halse; Jeannine Lama; Xuan Qin; Yun Wu
EPI_ISL_5071132, EPI_ISL_5071190, EPI_ISL_5072505, EPI_ISL_5072515, EPI_ISL_5072592	LABORATORIO ALIFE HEALTH	Instituto Nacional de Salud-Dirección de Investigación en Salud Pública	Carlos Franco-Muñoz; Carmen Osorio; Diana Malo; Diego A. Álvarez-Díaz; Diego Andrés Prada; Gerardo Santamaría; Hector Alejandro Ruiz-Moreno; Jhonnatan Reales-González; Jorge Rivera; Juan Camilo Martinez; Julian Naizaque; Katherine Laiton-Donato; Lisseth Pardo; Magdalena Wiesner; Marcela Mercado-Reyes; Maria T. Herrera-Sepúlveda; Marta Lopez Blanco; Martha Lucia Ospina Martinez; Paola Rojas-Estevez; Sergio Gomez; Sheryll Corchuelo; Ángela Alarcon Cruz
EPI_ISL_5055255, EPI_ISL_5055261, EPI_ISL_5055262, EPI_ISL_5055269, EPI_ISL_5055281, EPI_ISL_5055300	LABORATORIO CLINICO COMPENSAR	Instituto Nacional de Salud-Dirección de Investigación en Salud Pública	Beatriz de Arco; Carlos Franco-Muñoz; Carmen Osorio; Diana Malo; Diego A. Álvarez-Díaz; Diego Andrés Prada; Gerardo Santamaría; Hector Alejandro Ruiz-Moreno; Jhonnatan Reales-González; Jorge Rivera; Juan Camilo Martinez; Julian Naizaque; Katherine Laiton-Donato; Lisseth Pardo; Magdalena Wiesner; Marcela Mercado-Reyes; Maria T. Herrera-Sepúlveda; Marta Lopez Blanco; Martha Lucia Ospina Martinez; Paola Rojas; Sergio Gomez; Sheryll Corchuelo; Tatiana Cobos; Ángela Alarcon Cruz
EPI_ISL_4960511, EPI_ISL_4960512	LABORATORIO CLINICO HEMATOLOGICO	Laboratorio Departamental de Salud Publica de Antioquia	Cristian Arbey Velarde Hoyos; Gloria Isabel Escobar; Idabely Betancur Ortiz; Juan P. Hernandez-Ortiz; Maria Stella López
EPI_ISL_5334482	LABORATORIO CLINICO HIGUERA ESCALANTE	Instituto Nacional de Salud-Dirección de Investigación en Salud Pública	Beatriz de Arco; Carlos Franco-Muñoz; Diego A. Álvarez-Díaz; Diego Andrés Prada; Dioselina Peláez-Carvajal; Gerardo Santamaría; Héctor Alejandro Ruiz-Moreno; Jhonnatan Reales-González; Jorge Rivera; Julián Naizaque; Katherine Laiton-Donato; Marcela Mercado-Reyes.; Martha Lucia Ospina Martínez; María T. Herrera-Sepúlveda; Paola Rojas; Sheryll Corchuelo; Tatiana Cobos
EPI_ISL_4984816, EPI_ISL_5428461	LABORATORIO ECHAVARRIA	Laboratorio Departamental de Salud Publica de Antioquia	Ana Victoria Valencia Duarte; Andres F. Cardona-Rios; Cristian Arbey Velarde Hoyos; Gloria Isabel Escobar; Idabely Betancur Ortiz; Juan P. Hernandez-Ortiz; Juan Pablo Isaza Agudelo; Maria Stella López
EPI_ISL_5055131, EPI_ISL_5055132, EPI_ISL_5055141, EPI_ISL_5055174, EPI_ISL_5055216	LABORATORIO IMAT	Instituto Nacional de Salud-Dirección de Investigación en Salud Pública	Beatriz de Arco; Carlos Franco-Muñoz; Carmen Osorio; Diana Malo; Diego A. Álvarez-Díaz; Diego Andrés Prada; Gerardo Santamaría; Hector Alejandro Ruiz-Moreno; Jhonnatan Reales-González; Jorge Rivera; Juan Camilo Martinez; Julian Naizaque; Katherine Laiton-Donato; Lisseth Pardo; Magdalena Wiesner; Marcela Mercado-Reyes; Maria T. Herrera-Sepúlveda; Marta Lopez Blanco; Martha Lucia Ospina Martinez; Paola Rojas; Sergio Gomez; Sheryll Corchuelo; Tatiana Cobos; Ángela Alarcon Cruz
EPI_ISL_5055268	LABORATORIO LORENA VEJARANO S.A.S.	Instituto Nacional de Salud-Dirección de Investigación en Salud Pública	Beatriz de Arco; Carlos Franco-Muñoz; Carmen Osorio; Diana Malo; Diego A. Álvarez-Díaz; Diego Andrés Prada; Gerardo Santamaría; Hector Alejandro Ruiz-Moreno; Jhonnatan Reales-González; Jorge Rivera; Juan Camilo Martinez; Julian Naizaque; Katherine Laiton-Donato; Lisseth Pardo; Magdalena Wiesner; Marcela Mercado-Reyes; Maria T. Herrera-Sepúlveda; Marta Lopez Blanco; Martha Lucia Ospina Martinez; Paola Rojas; Sergio Gomez; Sheryll Corchuelo; Tatiana Cobos; Ángela Alarcon Cruz
EPI_ISL_4965717, EPI_ISL_4965728, EPI_ISL_4965734, EPI_ISL_4965742	LDSP	Laboratorio Departamental de Salud Publica de Antioquia	Andres F. Cardona-Rios; Cristian Arbey Velarde Hoyos; Gloria Isabel Escobar; Idabely Betancur Ortiz; Juan P. Hernandez-Ortiz; Maria Stella López
EPI_ISL_5055250	LDSP DEL CESAR	Instituto Nacional de Salud-Dirección de Investigación en Salud Pública	Beatriz de Arco; Carlos Franco-Muñoz; Carmen Osorio; Diana Malo; Diego A. Álvarez-Díaz; Diego Andrés Prada; Gerardo Santamaría; Hector Alejandro Ruiz-Moreno; Jhonnatan Reales-González; Jorge Rivera; Juan Camilo Martinez; Julian Naizaque; Katherine Laiton-Donato; Lisseth Pardo; Magdalena Wiesner; Marcela Mercado-Reyes; Maria T. Herrera-Sepúlveda; Marta Lopez Blanco; Martha Lucia Ospina Martinez; Paola Rojas; Sergio Gomez; Sheryll Corchuelo; Tatiana Cobos; Ángela Alarcon Cruz
EPI_ISL_4984610	LDSP de Antioquia	Laboratorio Departamental de Salud Publica de Antioquia	Andres F. Cardona-Rios; Cristian Arbey Velarde Hoyos; Gloria Isabel Escobar; Idabely Betancur Ortiz; Juan P. Hernandez-Ortiz; Maria Stella López
EPI_ISL_4917852	LESP Tabasco	Instituto de Diagnostico y Referencia Epidemiologicos (INDRE)	Abril Rodriguez-Maldonado; Ariadna Medina-Benitez; Claudia Wong-Arambula; Ernesto Ramirez-Gonzalez.; Fernando Gonzalez-Dominguez; Gisela Barrera-Badillo; Irma Lopez-Martinez; Joaquin Quiroz-Mercado; Lucia Hernandez-Rivas; Maribel Gonzalez-Villa; Natividad Cruz-Ortiz; Sergio Rangel-Guerrero; Tatiana Nunez-Garcia; Vanessa Rivero-Arredondo
EPI_ISL_4984862, EPI_ISL_4984875, EPI_ISL_5923355	LIME	Laboratorio Departamental de Salud Publica de Antioquia	Ana Victoria Valencia Duarte; Andres F. Cardona-Rios; Cristian Arbey Velarde Hoyos; Gloria Isabel Escobar; Idabely Betancur Ortiz; Juan P. Hernandez-Ortiz; Juan Pablo Isaza Agudelo; Maria Stella López
EPI_ISL_5020128	LSP Valle del Cauca	Molecular Genetics and Antimicrobial Resistance - UGRA, Universidad El Bosque	Catalina Espitia; Jinnethe Reyes; Lorena Diaz; Marcela Mercado; Mauricio Pacheco; Rafael Rios; Valentina Martinez
EPI_ISL_4417574, EPI_ISL_4417588, EPI_ISL_4417653	LSP de Atlantico	Centro de Investigaciones en Microbiología y Biotecnología-UR (CIMBIUR), Facultad de Ciencias Naturales, Universidad del Rosario, Bogotá, Colombia	Angie Ramírez; Juan David Ramírez; Luz H. Patiño; Marcela Mercado-Reyes; Marina Muñoz; Nathalia Ballesteros; Nicolas Niño; Sergio Castañeda
EPI_ISL_4419177	Lab Christus Sinergia Salud - Clinica Farallones	Universidad del Valle	Programa Nacional de Caracterización Genómica de SARS-CoV-2
EPI_ISL_5887479	Lab. Hospital clinico Universidad de Chile	"Facultad de Ciencias de la Vida, UNAB"	"Claudio Meneses; Ariel Orellana"; Claudio Olmos; Daniel Leon; Dayan Sanhueza; Eduardo Castro; Gonzalo Campaña; Macarena Bastias; Paola Pidal; Ricardo Yusta; Sebastian Wolter; Susana Saez; Víctor Monreal; Waldo Diaz
EPI_ISL_4412226, EPI_ISL_4412229, EPI_ISL_4412231	Labo Analyses Med	National Reference Center for Viruses of Respiratory Infections, Institut Pasteur, Paris	Angela Brisebarre; Camille Capel; Christophe Malabat; Corinne Chauvet; Corinne Maufrais; Etienne Simon-Lorière; Frédéric Lemoine; Julien Fumey; Louise Lefrançois; Marion Barbet; Maud Vanpeene; Méline Bizard; Slim El Khiari; Sylvie Behillil; Sylvie Van der Werf; Vincent Enouf
EPI_ISL_5422240, EPI_ISL_5897949, EPI_ISL_5897950, EPI_ISL_5897953, EPI_ISL_5898098	Laboratoire de santé publique du Québec	Laboratoire de santé publique du Québec	Guillaume Bourque; Ioannis Ragoussis; Jesse Shapiro; Mark Lathrop and Michel Roger on behalf of the CoVSeQ research group; Sandrine Moreira
EPI_ISL_5395466	Laboratoires Reunis	Laboratoire national de sante, Microbiology, Microbial Genomics Platform	Anke Wienecke-Baldacchino; Bernard Weber; Catherine Ragimbeau; Elodie Solarino; Fatu Djabi; Jessica Tapp; Lise Pignon; Raoul Salmon; Tamir Abdelrahman; Virginie Jover
EPI_ISL_4634221	Laboratori de Referencia de Catalunya	Laboratori de Referencia de Catalunya	Bellosillo B.; Canal M.; Hernandez JJ.; Padilla E.; Ramirez A.; Vilas A.
EPI_ISL_4946220, EPI_ISL_4946239, EPI_ISL_4946241	Laboratorio Angel (Synlab)	Instituto Nacional de Salud-Dirección de Investigación en Salud Pública	Carlos Franco-Muñoz; Carmen Osorio; Diana Malo; Diego A. Álvarez-Díaz; Diego Andrés Prada; Gerardo Santamaría; Hector Alejandro Ruiz-Moreno; Jhonnatan Reales-González; Jorge Rivera; Juan Camilo Martinez; Julian Naizaque; Katherine Laiton-Donato; Lisseth Pardo; Magdalena Wiesner; Marcela Mercado-Reyes; Maria T. Herrera-Sepúlveda; Marta Lopez Blanco; Martha Lucia Ospina Martinez; Paola Rojas; Sergio Gomez; Sheryll Corchuelo; Ángela Alarcon Cruz
EPI_ISL_6062547, EPI_ISL_6155758, EPI_ISL_6155762, EPI_ISL_6155769, EPI_ISL_6155775	Laboratorio Central, Ministerio de Salud Córdoba	Instituto de Patologia Vegetal (CIAP-INTA) on behalf of 'Proyecto Argentino Interinstitucional de genomica de SARS-CoV-2' (PAIS Consortium)	A; Amadio; Barbas, G.; Castro, G.; Debat; Debat, HJ.; FD; Fernández; Irazoqui; M; M.B.; Marquez, N.; Pisano; Re, V.

EPI_ISL_4417560, EPI_ISL_4417604, EPI_ISL_4417606, EPI_ISL_4417608, EPI_ISL_4417609, EPI_ISL_4417610, EPI_ISL_4417619, EPI_ISL_4417628, EPI_ISL_4417642, EPI_ISL_4417645, EPI_ISL_4417648

see above	Laboratorio Clinica Iberoamericana (Sanitas)	Centro de Investigaciones en Microbiología y Biotecnología-UR (CIMBIUR), Facultad de Ciencias Naturales, Universidad del Rosario, Bogotá, Colombia	Angie Ramírez; Juan David Ramírez; Luz H. Patiño; Marcela Mercado-Reyes; Marina Muñoz; Nathalia Ballesteros; Nicolas Niño; Sergio Castañeda
EPI_ISL_4703869, EPI_ISL_5395975	Laboratorio Clinico CITISALUD	Laboratorio de Biologia Molecular, Universidad Cooperativa de Colombia, Santa Marta	Andrew S. Muñoz-Gamba; Camila Gonzalez; Daniel B. Ramírez-Osorio; Johana Zúñiga; José A. Usme-Ciro; Maria J. Capmartin; Paula A. Quintero-Cortés; Robinson Alvarez
EPI_ISL_5103737	Laboratorio Clínico BioReferencia	Corporación para Investigaciones Biológicas-CIB	Jeanneth Mosquera Rendon; Jenny Santiago Cuesta; Marcela Mercado Reyes; Uriel A. Hurtado Paez
EPI_ISL_4878266, EPI_ISL_4878270, EPI_ISL_4878303	Laboratorio Direccion Sanidad Policia Nacional	Instituto Nacional de Salud-Dirección de Investigación en Salud Pública	Carlos Franco-Muñoz; Carmen Osorio; Diana Malo; Diego A. Álvarez-Díaz; Diego Andrés Prada; Gerardo Santamaría; Hector Alejandro Ruiz-Moreno; Jhonnatan Reales-González; Jorge Rivera; Juan Camilo Martinez; Julian Naizaque; Katherine Laiton-Donato; Lisseth Pardo; Magdalena Wiesner; Marcela Mercado-Reyes; Maria T. Herrera-Sepúlveda; Marta Lopez Blanco; Martha Lucia Ospina Martinez; Paola Rojas; Sergio Gomez; Sheryll Corchuelo; Ángela Alarcon Cruz
EPI_ISL_5063050, EPI_ISL_5063065	Laboratorio Direccion Sanidad Policia Nacional	Centro de Investigaciones en Microbiología y Biotecnología-UR (CIMBIUR), Facultad de Ciencias Naturales, Universidad del Rosario, Bogotá, Colombia	Angie Ramírez; Juan David Ramírez; Luz H. Patiño; Marcela Mercado-Reyes; Marina Muñoz; Nathalia Ballesteros; Nicolas Niño; Sergio Castañeda
EPI_ISL_4703599	Laboratorio Las Americas	Laboratorio de Biologia Molecular, Universidad Cooperativa de Colombia, Santa Marta	Adriana Espitia; Andrew S. Muñoz-Gamba; Daniel B. Ramírez-Osorio; Javier Martinez; José A. Usme-Ciro; Paula A. Quintero-Cortés
EPI_ISL_5103733, EPI_ISL_5103746, EPI_ISL_5103747, EPI_ISL_5103765, EPI_ISL_5103773, EPI_ISL_5103784, EPI_ISL_5103787, EPI_ISL_5103823, EPI_ISL_5103846			
see above	Laboratorio SYNLAB Colombia	Corporación para Investigaciones Biológicas-CIB	Jeanneth Mosquera Rendon; Jenny Santiago Cuesta; Marcela Mercado Reyes; Uriel A. Hurtado Paez
EPI_ISL_5199330, EPI_ISL_5199349	Laboratorio SYNLAB Colombia	Instituto Nacional de Salud-Dirección de Investigación en Salud Pública	Beatriz de Arco; Carlos Franco-Muñoz; Carmen Osorio; Diana Malo; Diego A. Álvarez-Díaz; Diego Andrés Prada; Gerardo Santamaría; Hector Alejandro Ruiz-Moreno; Jhonnatan Reales-González; Jorge Rivera; Juan Camilo Martinez; Julian Naizaque; Katherine Laiton-Donato; Lisseth Pardo; Magdalena Wiesner; Marcela Mercado-Reyes; Maria T. Herrera-Sepúlveda; Marta Lopez Blanco; Martha Lucia Ospina Martinez; Paola Rojas; Sergio Gomez; Sheryll Corchuelo; Tatiana Cobos; Ángela Alarcon Cruz
EPI_ISL_5020133	Laboratorio clínico Angeles Biología molecular	Molecular Genetics and Antimicrobial Resistance - UGRA, Universidad El Bosque	Catalina Espitia; Jinnethe Reyes; Lorena Diaz; Marcela Mercado; Mauricio Pacheco; Rafael Rios; Valentina Martinez
EPI_ISL_4703643, EPI_ISL_4703763, EPI_ISL_4703808	Laboratorio de Biologia Molecular, Universidad Cooperativa de Colombia, Santa Marta	Laboratorio de Biologia Molecular, Universidad Cooperativa de Colombia, Santa Marta	Andres Rojas-Gulloso; Andrew S. Muñoz-Gamba; Beatriz Maestre; Camila Gonzalez; Daniel B. Ramírez-Osorio; Emy S. Torres; Johanna Zúñiga; José A. Usme-Ciro; Paula A. Quintero-Cortés
EPI_ISL_5065711, EPI_ISL_5065721, EPI_ISL_5065730	Laboratorio de Biología Molecular - Universidad del Magdalena	Centro de Genética y Biología Molecular - Universidad del Magdalena	Andrea M. Ramírez Hernandez; Angel Oviedo Marquez; Daniel Bautista; Edison Lea-Ch; Lyda R. Castro; Maria Teresa Mojica-Ortiz
EPI_ISL_4578878, EPI_ISL_4578886	Laboratorio de Biología molecular del Hospital General de Agudos Dr. Carlos G. Durand	Área de Secuenciación del Laboratorio de Virología del Hospital de Niños Dr. Ricardo Gutierrez on behalf of 'Proyecto Argentino Interinstitucional de genomica de SARS-CoV-2' (PAIS Consortium)	A; Acuña; Anzorena; B; C; Carrón; Castro; Cañellas; Chiussi; Colina; Costa; D; Dahinten; Dima; Domínguez; E; Elsegood; F; Frisone; G; Gatica; Goya; Irrazábal; Jurado; L; LE; Leivas; Loayza; Lusso; M; MB; MF; MI; MS; Marchissio; Marina; Matillas; N; Nabaes Jodar; Narduzzi; Natale; Notaristéfano; R; Rivarola; Rodríguez Cardozo; Rodríguez Saá; Rozo; S; Theaux; Valinotto; Viegas, M.; W; Warszatska; Y; Yaunguzian
EPI_ISL_4890344, EPI_ISL_4890345, EPI_ISL_4890346, EPI_ISL_4890359	Laboratorio de Biología molecular y Biotecnología UTP	Laboratorio de Biología Molecular y Biotecnología - Universidad Tecnológica de Pereira	Augusto Zuluaga-Velez; Fredy A. Tabares-Villa; Juan C. Sepulveda; Juan D. Anacona-Montilla; Marcela Orjuela-Rodriguez
EPI_ISL_5147062	Laboratorio de Diagnóstico e Investigación BIOSCIENCE SRL	Laboratorio de Genómica Microbiana, Universidad Peruana Cayetano Heredia	Alejandra Dávila-Barclay; Diego Cuicapuza; Esdenka Pérez; Freddy Tinajeros; Guillermo Salvatierra; Janet Huancachoque; Luis González; Mauricio Prado; Pablo Tsukayama; Paula Carballo; Pedro E. Romero; Pool Marcos
EPI_ISL_5490873, EPI_ISL_5490878, EPI_ISL_5490886	Laboratorio de Ecologia de Doencas Transmissiveis na Amazonia, Instituto Leonidas e Maria Deane - Fiocruz Amazonia	Laboratorio de Ecologia de Doencas Transmissiveis na Amazonia, Instituto Leonidas e Maria Deane - Fiocruz Amazonia	Adele Schwartz Benzaken; André Corado; Felipe Naveca; Fernanda Nascimento; George Silva; José Joaquín Carvajal Cortés; Juan Camilo Grisales Nieto; Kelly Natalia Romero Vesga; Luciana Gonçalves; Maria Júlia Brandão; Matilde Mejía; Valdinete Nascimento; Victor Souza
EPI_ISL_6208403, EPI_ISL_6208578, EPI_ISL_6208579, EPI_ISL_6208610, EPI_ISL_6208698, EPI_ISL_6574128	Laboratorio de Referencia Nacional de Virus Respiratorios. Centro Nacional de Salud Publica. Instituto Nacional de Salud Peru.	Laboratorio de Referencia Nacional de Virus Respiratorios. Centro Nacional de Salud Publica. Instituto Nacional de Salud Peru.	Alicia Nuñez Llanos; Carlos Padilla Rojas; Edwart Rogger Rivera Serrano; Henri Bailon Calderon; Iris Silva Molina; Joseph Huayra Niquen; Kelly Vanessa Izarra Rojas; Lely Solari Zerpa; Luis Barcena Flores; Marco Galarza Perez; Nancy Rojas Serrano; Nieves Sevilla Castañeda; Omar Caceres Rey; Orson Mestanza Millones; Princesa Medrano Alhuay; Priscila Lope Pari; Sara Gordillo Vilchez; Steve Acedo Lazo; Veronica Hurtado Vela; Victor Jimenez Vasquez; Wendy Lizarraga Olivares
EPI_ISL_4578879, EPI_ISL_4578882	Laboratorio de Virología del Hospital de Niños Dr. Ricardo Gutierrez	Área de Secuenciación del Laboratorio de Virología del Hospital de Niños Dr. Ricardo Gutierrez on behalf of 'Proyecto Argentino Interinstitucional de genomica de SARS-CoV-2' (PAIS Consortium)	AS; Acevedo; Acuña; Alexay; Alvarez Lopez; C; D; E; Goya; Gravis; Jacques; LE; Lusso; ME; MI; MS; Mistchenko; Nabaes Jodar; Natale; O; S; Valinotto; Viegas, M.
EPI_ISL_6368859	Laboratorio nacional de salud publica	Viral Special Pathogens Branch, Centers for Disease Control and Prevention	Emir Talundzic; Isaac Miguel; Joel Montgomery; John Klena; Justin Lee; Melissa Mobley; Ronald Skewes-Ramm; Shannon Whitmer
EPI_ISL_4372847, EPI_ISL_4373371, EPI_ISL_4373672, EPI_ISL_4375408, EPI_ISL_4375456, EPI_ISL_4376827, EPI_ISL_4377577, EPI_ISL_4379164, EPI_ISL_4381338, EPI_ISL_4381935, EPI_ISL_4382021, EPI_ISL_4382170, EPI_ISL_4484580, EPI_ISL_4488844, EPI_ISL_4488845, EPI_ISL_4811830, EPI_ISL_4814264, EPI_ISL_4963077, EPI_ISL_4965554, EPI_ISL_4966220, EPI_ISL_5214209, EPI_ISL_5222609, EPI_ISL_5223528, EPI_ISL_5223668, EPI_ISL_5223669, EPI_ISL_5223812, EPI_ISL_5223907, EPI_ISL_5562250, EPI_ISL_5573377, EPI_ISL_6079093			
see above	Laboratory Corporation of America	Centers for Disease Control and Prevention Division of Viral Diseases, Pathogen Discovery	Amanda Douglas; Amanda Suchanek; Andrea Throop; Ayla Burns; Benjamin Rambo-Martin; Bobbi Croy; Brian Krueger; Brian Norvell; Christopher Gulvick; Christos Petropoulos; Clinton Paden; Craig Lukasik; Dakota Howard; Debbie Boles; Dhwani Batra; Duncan MacCannell; Eyad Almasri; Goran Stevovic; Howard Engler; Hrushikesh Deshmukh; Jake Humphrey; Jana Schroth; Jason Caravas; Joe Voshell; John Pruitt; Jonathan Meltzer; Jonathan Williams; Kimberly Wagner; Kristine Lacek; Lax Iyer; Lisa Pfefferle; Lyndon Tilson; Manoj Jain; Marcia Eisenberg; Mary Cristobal; Mary Williamson; Matthew Robinson; Matthew Schmerer; Michael Levandoski; Mike Sapeta; Mindy Nye; Minoo Agarwal; Mohan Kolli; Nuthawin Charoensri; Oren Cohen; Peter Cook; Prashant Gupta; Qian Zeng; Rama Ghatti; Scott Parker; Scott Ryan; Scott Sammons; Shatavia Morrison; Stanley Letovsky; Steven Ragan; Suresh Selvaraju; Susan Countryman; Susan Hicks; Suzanne Dale; Thomas Urban; Tim Kuphal; Tricia Zwiefelhofer; Tymeckia Kendall; Victoria Caban Figueroa; Vincent Drouillon; Yvette Unoarumhi
EPI_ISL_4516039	Laboratório Central de Saúde Pública do Amazonas - LACEN-AM	Laboratorio de Ecologia de Doencas Transmissiveis na Amazonia, Instituto Leonidas e Maria Deane - Fiocruz Amazonia	André Corado; Felipe Naveca; Fernanda Nascimento; George Silva; Karina Pessoa; Luciana Gonçalves; Maria Júlia Brandão; Matilde Mejía; Valdinete Nascimento; Victor Souza
EPI_ISL_6492839, EPI_ISL_6493133	MSHS Clinical Microbiology Laboratories	MSHS Pathogen Surveillance Program	Adolfo García-Sastre; Adriana van de Guchte; Ajay Obla; Alberto Paniz-Mondolfi; Ana S. Gonzalez-Reiche; Angela Amoako; Ashley Salimbangon; Betsaida Salom Melo; Bremy Alburquerque; Brianne Ciferri; Charles Gleason; Daniel Floda; Deena R. Altman; Denise Jurczyszak; Emilia Mia Sordillo; Gintaras Deikus; Giulio Kleiner; Gopi Patel; Hala Alshammary; Harm van Bakel; Irina Oussenko; Jayeeta Dutta; Juan Soto; Julia Matthews; Katherine Beach; Kathryn Twyman; Kayla Russo; Komal Srivastava; Levy Sominsky; Mahmoud Awawda; Marta Luksza; Matthew M. Hernandez; Melissa Gitman; Michael D. Nowak; Mitchell J. Sullivan; Nancy Francoeur; Robert Sebra; Sarah Schaefer; Shelcie Fabre; Shwetha Hara Sridhar; Viviana Simon; Ying-Chih Wang; Zain Khalil; Zenab Khan
EPI_ISL_5471871	Maasstad Ziekenhuis	Maasstad ziekenhuis	Pieter Smit
EPI_ISL_5366551	Maine Health and Environmental Testing Laboratory	Tewhey Lab, The Jackson Laboratory	Barter, M.; Dewey, H.; H. and Tewhey, R.; Iosue, F.; Lynch, R.; Matluk, N.; Munger
EPI_ISL_4463015, EPI_ISL_4463016	Maryland Genomics, Institute for Genome Sciences, University of Maryland School of Medicine	Maryland Genomics, Institute for Genome Sciences, University of Maryland School of Medicine	Claire M; Fraser; Hazen; Holly; Humphrys; Ivette; Jacques; Jonathan; Kranthi; Lim; Lisa D; Luke J; Mike; Ott; Ravel; Roussey; Sadzewicz; Sandra; Santana-Cruz; Tallon; Tracy; Vavikolanu
EPI_ISL_5199359, EPI_ISL_5438634	Massachusetts State Public Health Laboratory	Massachusetts State Public Health Laboratory	Andrew Lang; Glen Gallagher; Sandra Smole; Timelia Fink
EPI_ISL_4552962	Michigan Department of Health and Human Services, Bureau of Laboratories	Michigan Department of Health and Human Services, Bureau of Laboratories	Blankenship HM; Riner D; Soehnlen MK
EPI_ISL_4458487	Microbiology Department. Complexo Hospitalario Universitario de Vigo	Microbiology Department. Complexo Hospitalario Universitario de Vigo	Alvarez M; Cabrera JJ; Carballo R; Cores O; Cortizo S; Davina C; Martinez L; Mediero G; Pena I; Perez S; Potel C; Regueiro B; Rey S; Vasallo FJ; del-Campo V

EPI_ISL_4936740	NJDOH, Public Health and Environmental Laboratories	NJ PHEL	Byeong Jeong; Chelsea San Filippo; Dana Woell; Lindsey Bodnar; Maria-Magdalene Pugliese; Mohammad M. Ali; Ryan Pachucki; Shiv K. Verma
EPI_ISL_5429195	National Public Health Laboratory, Ministry of Health & Wellness	Viral Special Pathogens Branch, Centers for Disease Control and Prevention	Antoni Comrie; Carl Bruce; Emir Talundzic; Joel Montgomery; John Klena; Justin Lee; Karen Webster-Kerr; Michelle Brown; Michelle Hamilton; Monica Smikle; Nathlee McMorris; Sandra Jackson; Shannon Whitmer; Suwanie Lewis
EPI_ISL_4984903, EPI_ISL_4984954, EPI_ISL_4984955	PECET UDEA	Laboratorio Departamental de Salud Publica de Antioquia	Andres F. Cardona-Rios; Cristian Arbey Velarde Hoyos; Gloria Isabel Escobar; Idabely Betancur Ortiz; Juan P. Hernandez-Ortiz; Maria Stella López
EPI_ISL_4458156	PR Public Health Lab	Centers for Disease Control and Prevention Division of Viral Diseases, Pathogen Discovery	Becky Tsai; Benafsh Sapra; Benjamin Rambo-Martin; Christopher Gulvick; Clinton Paden; Dakota Howard; Dhwani Batra; Doreen Ng; Duncan MacCannell; Erisa Sula; Harry Gao; James Xie; Jason Caravas; John Gao; Joseph Fierro; Kristine Lacek; Matthew Schmerer; Mickey Li; Peter Cook; Scott Sammons; Shatavia Morrison; Tymeckia Kendall; Victoria Caban Figueroa; Yan Meng; Yvette Unoarumhi
EPI_ISL_5540381, EPI_ISL_6037383	Pandemic Response Lab - NYC	Pandemic Response Lab, R&D	Alex Carpio; Cybill del Castillo; Dylan Law; Haiping Hao; Henry Lee; Isabel Fernandez Escapa; Jon Laurent; Melissa Hopkins; Michael Hammerling; Pradeep Bugga; Shinyoung Clair Kang; Sol Rey; William Ward
EPI_ISL_4473154	Public Health Authority of the Slovak Republic	Public Health Authority of the Slovak Republic	Anna Gičová; Barbora Kotvasová; Elena Tichá; Lucia Ševčíková; Miroslav Böhmer; Pavol Mišenko; Terézia Vrabľová; Tomáš Szemes
EPI_ISL_4409524, EPI_ISL_4409728, EPI_ISL_4410095, EPI_ISL_4564064, EPI_ISL_4564065, EPI_ISL_6339154	Public Health Ontario Laboratory	Public Health Ontario Laboratory	Aimin Li; Alireza Eshaghi; Andre Villegas; Ashleigh Sullivan; Christine Frantz; Dean Maxwell; Esha Joshi; Jared Simpson; Jennifer L Guthrie; Jonathan B Gubbay; Karthikeyan Sivaraman; Lawrence Heisler; Matthew Watson; Michael CY Li; Michael Laszloffy; Nahuel Fittipaldi; Philip Banh; Richard de Borja; Samir N Patel; Sandeep Nagra; Sandra Zittermann; Sarah Teatero; Vanessa G Allen; Yao Chen; Yogi Sundaravadanam
EPI_ISL_5104527	Puerto Rico Department of Health	Centers for Disease Control and Prevention, Dengue Branch	Betzabel Flores; Gabriela Paz-Bailey; Gilberto A. Santiago; Glenda Gonzalez; Jorge L. Munoz-Jordan; Keyla Charriez
EPI_ISL_4372352, EPI_ISL_4372365, EPI_ISL_4372480, EPI_ISL_4372681, EPI_ISL_4372933, EPI_ISL_4372986, EPI_ISL_4373162, EPI_ISL_5068063, EPI_ISL_5846287, EPI_ISL_5929268			
see above	Quest Diagnostics Incorporated	Centers for Disease Control and Prevention Division of Viral Diseases, Pathogen Discovery	A. Gerasimova; A. Perez; B. Anderson; Benjamin Rambo-Martin; Christopher Gulvick; Clinton Paden; Dakota Howard; Dhwani Batra; Duncan MacCannell; Erisa Sula; F. Lacbawan; I. Shlyakhter; Jason Caravas; K. Livingston; Kristine Lacek; L. Bernstein; M. Hua; Matthew Schmerer; P. Tanpaiboon; Peter Cook; R. Kagan; R. Owen; R. Rolando; S. Rosenthal; Scott Sammons; Shatavia Morrison; Tymeckia Kendall; Victoria Caban Figueroa; Y. Liu; Yvette Unoarumhi
EPI_ISL_4531131, EPI_ISL_5810934	Respiratory Virus Unit, Microbiology Services Colindale, Public Health England	COVID-19 Genomics UK (COG-UK) Consortium	PHE Covid Sequencing Team
EPI_ISL_4520808	Rey Fals	Universidad Nacional de Colombia - Laboratorio Genómico One Health	Andres F. Cardona-Rios; Carlos Franco-Muñoz; Carolina Muñoz-Arango; Celeny Ortiz; Daniel O. Maldonado-Perez; Diego A. Álvarez-Díaz; Hector Alejandro Ruiz-Moreno; Idabely Betancur Ortiz; Jorge E. Osorio; Juan P. Hernandez-Ortiz; Karl A Ciuoderis; Katherine Laiton-Donato; Laura Silvana Perez; Lina M. Hurtado; Marcela Mercado-Reyes; Maria Angélica Maya; Maria Stella López; Rita Almanza Payares; Sandra Ines Cano; Simón Villegas Velásquez
EPI_ISL_4542967	Rush University Medical Center	RIPHL at Rush University Medical Center	Diane Springer; Edith Perez; Felix Araujo Perez; Joyce Houlihan; Kevin Kunstman; Laura Furtado; Marieta Hyde; Mary Hayden; Sofiya Bobrovska; Stefan Green
EPI_ISL_5031027	SALUD DIGNA	Instituto Nacional de Medicina Genomica	Abraham Campos-Romero; Cedro-Tanda A; Cruz-Islas Jazmin; Escobar-Arrazola MA; Garnica-Lopez Dora; Herrera-Montalvo LA.; Hidalgo-Miranda A; Luna-Ruiz Marco; Mendoza-Vargas A; Moreno-Camacho José Luis; Ramirez-Vega O; Rangel-DeLeon D; Reyes-Grajeda JP; Rodriguez-Gallegos Jorge; Yair Alfaro-Mora
EPI_ISL_5143473, EPI_ISL_5143483, EPI_ISL_5143486, EPI_ISL_5143505, EPI_ISL_6512535	SOMER	Laboratorio Departamental de Salud Publica de Antioquia	Ana Victoria Valencia Duarte; Cristian Arbey Velarde Hoyos; Gloria Isabel Escobar; Idabely Betancur Ortiz; Juan P. Hernandez-Ortiz; Juan Pablo Isaza Agudelo; Maria Stella López
EPI_ISL_4740147	SURA	Gencore - Universidad de los Andes	Cristian Barrera; Felipe Báez; Gabriela Ariza; Luisa Sacristan; Marcela Guevara; Marcela Mercado; Silvia Restrepo
EPI_ISL_4965818, EPI_ISL_4984622, EPI_ISL_5143457, EPI_ISL_5143458, EPI_ISL_5143504	SURA	Laboratorio Departamental de Salud Publica de Antioquia	Andres F. Cardona-Rios; Cristian Arbey Velarde Hoyos; Gloria Isabel Escobar; Idabely Betancur Ortiz; Juan P. Hernandez-Ortiz; Maria Stella López
EPI_ISL_4878272	SURA Antioquia	Instituto Nacional de Salud-Dirección de Investigación en Salud Pública	Carlos Franco-Muñoz; Carmen Osorio; Diana Malo; Diego A. Álvarez-Díaz; Diego Andrés Prada; Gerardo Santamaría; Hector Alejandro Ruiz-Moreno; Jhonnatan Reales-González; Jorge Rivera; Juan Camilo Martinez; Julian Naizaque; Katherine Laiton-Donato; Lisseth Pardo; Magdalena Wiesner; Marcela Mercado-Reyes; Maria T. Herrera-Sepúlveda; Marta Lopez Blanco; Martha Lucia Ospina Martinez; Paola Rojas; Sergio Gomez; Sheryll Corchuelo; Ángela Alarcon Cruz
EPI_ISL_4878283	SURA Bogota	Instituto Nacional de Salud-Dirección de Investigación en Salud Pública	Carlos Franco-Muñoz; Carmen Osorio; Diana Malo; Diego A. Álvarez-Díaz; Diego Andrés Prada; Gerardo Santamaría; Hector Alejandro Ruiz-Moreno; Jhonnatan Reales-González; Jorge Rivera; Juan Camilo Martinez; Julian Naizaque; Katherine Laiton-Donato; Lisseth Pardo; Magdalena Wiesner; Marcela Mercado-Reyes; Maria T. Herrera-Sepúlveda; Marta Lopez Blanco; Martha Lucia Ospina Martinez; Paola Rojas; Sergio Gomez; Sheryll Corchuelo; Ángela Alarcon Cruz
EPI_ISL_5152646, EPI_ISL_5152661	SYNLAB	CIAT, Laboratorio de Virologia	Ana M. Leiva; Diana Lopez; Programa Nacional de Caracterización Genómica de SARS/CoV/2; WilmerJ. Cuellar
EPI_ISL_4878281, EPI_ISL_4878293	SYNLAB	Instituto Nacional de Salud-Dirección de Investigación en Salud Pública	Carlos Franco-Muñoz; Carmen Osorio; Diana Malo; Diego A. Álvarez-Díaz; Diego Andrés Prada; Gerardo Santamaría; Hector Alejandro Ruiz-Moreno; Jhonnatan Reales-González; Jorge Rivera; Juan Camilo Martinez; Julian Naizaque; Katherine Laiton-Donato; Lisseth Pardo; Magdalena Wiesner; Marcela Mercado-Reyes; Maria T. Herrera-Sepúlveda; Marta Lopez Blanco; Martha Lucia Ospina Martinez; Paola Rojas; Sergio Gomez; Sheryll Corchuelo; Ángela Alarcon Cruz
EPI_ISL_5202113, EPI_ISL_5202128, EPI_ISL_5202151, EPI_ISL_5202240, EPI_ISL_5202241, EPI_ISL_5202248, EPI_ISL_5202354, EPI_ISL_5202382, EPI_ISL_5202421, EPI_ISL_5202610, EPI_ISL_5202611, EPI_ISL_5202693			
see above	SYNLAB	LSPSDS	Alejandro Gomez Lopez; Gabriela Delgado Murcia; Johana Hernandez Toloza; Marcela Castano Rodriguez; Marcela Mercado
EPI_ISL_4984548, EPI_ISL_4984717, EPI_ISL_4984831	SYNLAB	Laboratorio Departamental de Salud Publica de Antioquia	Andres F. Cardona-Rios; Cristian Arbey Velarde Hoyos; Gloria Isabel Escobar; Idabely Betancur Ortiz; Juan P. Hernandez-Ortiz; Maria Stella López
EPI_ISL_4878527	SYNLAB	Universidad Nacional de Colombia - Laboratorio Genómico One Health	Andres F. Cardona-Rios; Carlos Franco-Muñoz; Carolina Muñoz-Arango; Celeny Ortiz; Daniel O. Maldonado-Perez; Diego A. Álvarez-Díaz; Hector Alejandro Ruiz-Moreno; Idabely Betancur Ortiz; Jorge E. Osorio; Juan P. Hernandez-Ortiz; Karl A Ciuoderis; Katherine Laiton-Donato; Laura Silvana Perez; Lina M. Hurtado; Marcela Mercado-Reyes; Maria Angélica Maya; Maria Stella López; Rita Almanza Payares; Sandra Ines Cano; Simón Villegas Velásquez
EPI_ISL_4410699	Salud Digna	Instituto Nacional de Medicina Genomica	Abraham Campos-Romero; Cedro-Tanda A; Cisneros-Villanueva M; Cruz-Islas Jazmin; Escobar-Arrazola MA; Garnica-Lopez Dora; Herrera-Montalvo LA.; Hidalgo-Miranda A; Luna-Ruiz Marco; Mendoza-Vargas A; Moreno-Camacho José Luis; Ramirez-Vega O; Rangel-DeLeon D; Reyes-Grajeda JP; Rodriguez-Gallegos Jorge; Yair Alfaro-Mora
EPI_ISL_4920267	Servicio Microbiología H.U. Dr. Negrín	Servicio Microbiología H.U. Dr. Negrín	Ana Bordes Benítez; Bartolomé Gómez Arroyo; Francisco Javier Chamizo López
EPI_ISL_5655844	Servicio de Microbiología Clínica (Complejo Hospitalario de Navarra, Pamplona)	Centro de Secuenciación NASERTIC	Ana Miqueleiz; Ana Navascués; Carmen Ezpeleta Baquedano
EPI_ISL_6080358	Shamir Medical Center (Asaf Harofe)	Shamir Medical Center (Asaf Harofe)	Abu Hamad Ramzia; Adina Bar Chaim; Anna Vishnevsky; Chen Weiner; Nir Rainy; Patricia Benveniste-Lekovitz; Reut Sorek Abramovich; Yevgeni Yegorov
EPI_ISL_6402371	TGen North	TGen North	Brett Van Tassel; Chris French; Darrin Lemmer; Dave Engelthaler; Hayley Yaglom; Heather Centner; Jolene Bowers
EPI_ISL_5334484	UNIDAD HEMATOLOGICA ESPECIALIZADA	Instituto Nacional de Salud-Dirección de Investigación en Salud Pública	Beatriz de Arco; Carlos Franco-Muñoz; Diego A. Álvarez-Díaz; Diego Andrés Prada; Dioselina Peláez-Carvajal; Gerardo Santamaría; Héctor Alejandro Ruiz-Moreno; Jhonnatan Reales-González; Jorge Rivera; Julián Naizaque; Katherine Laiton-Donato; Marcela Mercado-Reyes.; Martha Lucia Ospina Martínez; María T. Herrera-Sepúlveda; Paola Rojas; Sheryll Corchuelo; Tatiana Cobos
EPI_ISL_6012788	UNILABS	Instituto Nacional de Saude (INSA)	Borges et al
EPI_ISL_5055405	UNIVERSIDAD DE ANTIOQUIA - LIME	Instituto Nacional de Salud-Dirección de Investigación en Salud Pública	Beatriz de Arco; Carlos Franco-Muñoz; Carmen Osorio; Diana Malo; Diego A. Álvarez-Díaz; Diego Andrés Prada; Gerardo Santamaría; Hector Alejandro Ruiz-Moreno; Jhonnatan Reales-González; Jorge Rivera; Juan Camilo Martinez; Julian Naizaque; Katherine Laiton-Donato; Lisseth Pardo; Magdalena Wiesner; Marcela Mercado-Reyes; Maria T. Herrera-Sepúlveda; Marta Lopez Blanco; Martha Lucia Ospina Martinez; Paola Rojas; Sergio Gomez; Sheryll Corchuelo; Tatiana Cobos; Ángela Alarcon Cruz
EPI_ISL_4472792	UW Virology Lab	UW Virology Lab	Alexander Greninger; Hong Xie; Keith R Jerome; Maria Lukes; Meei-Li Huang; Nathan Breit; Patrick Mathias; Pavitra Roychoudhury; Ricardo Perez; Robert J. Livingston; Sean Ellis; Shah Mohamed Bakhash; Tien V. Nguyen
EPI_ISL_4879059	Unidad de Investigación Médica de Yucatán (UIMY)	Instituto de Biotecnología de la UNAM	; Alejandra García-Gasca; Alejandra Hernández-Terán; Alejandro Sánchez-Flores; Alfredo Herrera-Estrella; Alicia Ocaña-Mondragón; Andreu Comas-García; Angel Gustavo Salas-Lais; Antonio Loza Román; Bernardo Martínez-Miguel; Blanca Taboada; Brenda Irasema Maldonado-Meza; Bruno Gómez-Gil; Carla Ivón Herrera-Najera; Carlos F. Arias; Celia Boukadida; Clara Esperanza Santacruz-Tinoco; Concepción Grajales-Muñiz; Consorcio Mexicano de Vigilancia Genómica (CoViGen-Mex). Authors (in alphabetical order): Julio Elias Alvarado-Yaah; Cristóbal Cháidez-Quiróz; Célida Duque Molina; Célida Martínez- Rodríguez; Daniel Fregoso-Rueda; Daniel Lira Morales; Eduardo Becerril-Vargas; Fernando Fontove-Herrera; Fidencio Mejía-Nepomuceno; Francisco Pulido; Gloria Elena Espinosa-Ayala; Gloria María Molina-Salinas; Gloria Vazquez; Hector Esteban Paz-Juárez; Hector Montoya-Fuentes; Helen Haydee Fernanda Ramírez-Plascencia; Irvin González-López; Jean Pierre González; Jesús Hernández; Joel Armando Vázquez-Pérez).; Jorge Salas-Hernández; José Antonio Enciso-Moreno; José Arturo Martínez-Orozco; José Esteban Muñoz-Medina; José de Jesús Nuñez-Contreras; Juan Bautista Chale-Dzul; Julissa Enciso-Ibarra; Kathia Elizabeth Tapia-Diaz; Luis Alberto Ochoa-Carrera; Margarita Matías-Florentino; Mario Mújica-Sánchez; Marissa Perez-Garcia; María Guadalupe Santiago-Mauricio; María Guadalupe de Jesús Mireles-Rivera; Nelly Sélem-Mojica; Pavel Isa; Ricardo Ciria Merce; Ricardo Grande; Rosa María Gutiérrez Rios; Santiago Ávila-Ríos; Selene Zárate; Susana Lopez; Verónica Mata-Haro; Victor Eduardo García-Arias; Víctor Hugo Borja-Aburto
EPI_ISL_5887477	Universidad San Sebastian	"Facultad de Ciencias de la Vida, UNAB"	"Claudio Meneses; Ariel Orellana"; Claudio Olmos; Daniel Leon; Dayan Sanhueza; Eduardo Castro; Gonzalo Campaña; Macarena Bastias; Paola Pidal; Ricardo Yusta; Sebastian Wolter; Susana Saez; Víctor Monreal; Waldo Diaz
EPI_ISL_5065684, EPI_ISL_5065695, EPI_ISL_5065713,	Universidad del Sinú - Sede Cartagena	Centro de Genética y Biología Molecular - Universidad del Magdalena	Andrea M. Ramírez Hernandez; Angel Oviedo Marquez; Daniel Bautista; Edison Lea-Ch; Lyda R. Castro; Maria Teresa Mojica-Ortiz

EPI_ISL_5065725			
EPI_ISL_4419161, EPI_ISL_4419165, EPI_ISL_4419174	Universidad del Valle, LDAB-Laboratorio de Diagnostico de Agentes Biologicos	Universidad del Valle	Programa Nacional de Caracterización Genómica de SARS-CoV-2
EPI_ISL_5397463	Universitätsklinikum Köln; Institut für Virologie	Robert Koch Institute	
EPI_ISL_5421948	Usansolo-Galdakao University Hospital	Cruces University Hospital	Ana Belén de la Hoz; Ana Gual-de-Torrella; Izaskun Alejo-Cancho; Mikel Gallego
EPI_ISL_4494213, EPI_ISL_5334261, EPI_ISL_5334279	Utah Public Health Laboratory	Utah Public Health Laboratory	Erin L. Young; John Arnn; Kelly F. Oakeson; Olinto Linares-Perdomo; Pooja Gupta
EPI_ISL_4760533	V20	Y20	Amy Bauer; Jenny Lentz; Manjeet Khubbar; Nandhukumar Balakrishnan; Samantha Scott; Sanjib Bhattacharyya
EPI_ISL_5511723	Wyoming Public Health Laboratory	Wyoming Public Health Laboratory	Ashley Norberg; Brian Dominguez; Cari Sloma; Channing Weber; Chayse Rowley; Elliot Thomasson; Jim Mildenberger; Marley Goetz; Robert Petit; Sam Britz; Taylor Fearing; and Rob Christensen
EPI_ISL_4520825	Yamina Cumplido Romero	Universidad Nacional de Colombia - Laboratorio Genómico One Health	Andres F. Cardona-Rios; Carlos Franco-Muñoz; Carolina Muñoz-Arango; Celeny Ortiz; Daniel O. Maldonado-Perez; Diego A. Álvarez-Díaz; Hector Alejandro Ruiz-Moreno; Idabely Betancur Ortiz; Jorge E. Osorio; Juan P. Hernandez-Ortiz; Karl A Ciuoderis; Katherine Laiton-Donato; Laura Silvana Perez; Lina M. Hurtado; Marcela Mercado-Reyes; Maria Angélica Maya; Maria Stella López; Rita Almanza Payares; Sandra Ines Cano; Simón Villegas Velásquez

We gratefully acknowledge the following Authors from the Originating laboratories responsible for obtaining the specimens, as well as the Submitting laboratories where the genome data were generated and shared via GISAID, on which this research is based.

All Submitters of data may be contacted directly via www.gisaid.org

Authors are sorted alphabetically.

Acknowledgement EPI_SET Identifier: EPI_SET_20220328wc

Accession ID	Originating Laboratory	Submitting Laboratory	Authors
EPI_ISL_7660301	ADILAB	Laboratorio Departamental de Salud Publica de Antioquia	Ana Victoria Valencia Duarte; Cristian Arbey Velarde Hoyos; Gloria Isabel Escobar; Idabely Betancur Ortiz; Juan P. Hernandez-Ortiz; Juan Pablo Isaza Agudelo; Maria Stella López
EPI_ISL_7809039	AFIP	Instituto Butantan	Antonio Jorge Martins; Claudia Renata dos Santos Barros; David Schlesinger; Debora Botequio Moretti; Dimas Tadeu Covas; Elaine Cristina Marqueze; Elaine Vieira Santos; Evandra Strazza Rodrigues; Heidge Fukumasu; Jayme Augusto de Souza-Neto; José Salvatore Leister Patané; Luiz Alcantara; Luiz Lehmann Coutinho; Maria Carolina Elias; Maurício Lacerda Nogueira; Rafael dos Santos Bezerra; Raul Machado Neto; Rejane Maria Tommasini Grotto; Ricardo Haddad; Sandra Coccuzzo Sampaio Vessoni; Simone Kashima; Svetoslav Nanev Slavov; Vincent Louis Viala
EPI_ISL_6649094, EPI_ISL_6786693	Aegis Sciences Corporation	Centers for Disease Control and Prevention Division of Viral Diseases, Pathogen Discovery	Alec Vest; Benjamin Rambo-Martin; Christopher Gulvick; Clinton Paden; Cyndi Clark; Dakota Howard; Dhwani Batra; Dillon Nall; Duncan MacCannell; Erisa Sula; Ethan Sanders; Holly Houdeshell; Jason Caravas; Kristine Lacek; Matthew Hardison; Matthew Schmerer; Ola Kvalvaag; Patrick Campbell; Peter Cook; Rob Case; Scott Sammons; Shatavia Morrison; Shaun Westlund; Tymeckia Kendall; Victoria Caban Figueroa; Vikramsinha Ghorpade; Yvette Unoarumhi
EPI_ISL_7711703	Area De Salud Cartago	Universidad de Costa Rica e Instituto Costarricense de Investigacion y Ensenanza en Nutricion y Salud	Brenes Hebleen; Camacho Erwin; Campos-Sanchez Rebeca; Ceballos Ana; Cordero Estela; Cristancho Marco; Duarte Francisco; Fernandez-Do Porto Dario; Herrera-Estrella Alfredo; Jimenez-Moraila Beatriz; Kreuze Jan; Mireles-Rivera Guadalupe; Molina-Mora Jose; Munoz-Medina Jose Esteban; Negri Tatiana; Nunes Gisele; Oliveira Guilherme; Oliveira Renato; Reales-Gonzalez Jhonnatan; Remes-Lenicov Federico; Reyes Alejandro; Sosa Ezequiel; Soto Claudio; Tsukayama Pablo; Turjanski Adrian; and Zayat Jonathan
EPI_ISL_7711707	Area De Salud Heredia Cubujuchi	Universidad de Costa Rica e Instituto Costarricense de Investigacion y Ensenanza en Nutricion y Salud	Brenes Hebleen; Camacho Erwin; Campos-Sanchez Rebeca; Ceballos Ana; Cordero Estela; Cristancho Marco; Duarte Francisco; Fernandez-Do Porto Dario; Herrera-Estrella Alfredo; Jimenez-Moraila Beatriz; Kreuze Jan; Mireles-Rivera Guadalupe; Molina-Mora Jose; Munoz-Medina Jose Esteban; Negri Tatiana; Nunes Gisele; Oliveira Guilherme; Oliveira Renato; Reales-Gonzalez Jhonnatan; Remes-Lenicov Federico; Reyes Alejandro; Sosa Ezequiel; Soto Claudio; Tsukayama Pablo; Turjanski Adrian; and Zayat Jonathan
EPI_ISL_8482343, EPI_ISL_8482347, EPI_ISL_8482372, EPI_ISL_8482721, EPI_ISL_8482723, EPI_ISL_8482739, EPI_ISL_8482740, EPI_ISL_8482746, EPI_ISL_8482750, EPI_ISL_8482922, EPI_ISL_8482926, EPI_ISL_8482928, EPI_ISL_8482929, EPI_ISL_8482930, EPI_ISL_8482931, EPI_ISL_8482933, EPI_ISL_8482939, EPI_ISL_8482941, EPI_ISL_8482944, EPI_ISL_8482952, EPI_ISL_8482955, EPI_ISL_8482957, EPI_ISL_8482958, EPI_ISL_8482965, EPI_ISL_8482966, EPI_ISL_8482974, EPI_ISL_8482977, EPI_ISL_8482979, EPI_ISL_8482997			
see above	Biotechnology Core Facility Branch, Centers for Disease Control and Prevention	Centers for Disease Control and Prevention Division of Viral Diseases, Pathogen Discovery	; Alex Burgin; Alison Nicholson; Anir Enkhbat; Antoni Comrie; Ariuntuya Ganbat; Battur Lkhagvaa; Ben Rambo-Martin; Bilegtsaikhan Sukhee; Carl Bruce; Clinton Paden; Dakota Howard; Davaasukh Battumur; Dave Wentworth; Dhwani Batra; Ganbold Dalantai; Gulnar Genden; Jasmine Padilla; Joseph Madden; Justin Lee; Karen Webster-Kerr; Kristen Knipe; Kristine Lacek; Mark Burroughs; Matthew Schmerer; Meghan Bentz; Michelle Brown; Michelle Hamilton; Mili Sheth; Nathlee McMorris; Peter Cook; Sam Shepard; Sandra Jackson; Sarah Nobles; Suwanie Lewis; Vivien Dugan; Yvette Unoarumhi
EPI_ISL_7308809, EPI_ISL_8395542, EPI_ISL_8395951, EPI_ISL_8396009, EPI_ISL_8396231, EPI_ISL_8397336	CDPH VBL	California Department of Public Health	CDPH-COVIDNet; Emily Smith on behalf of CDPH-COVIDNet
EPI_ISL_7507357	Cedars-Sinai Medical Center, Molecular Pathology Laboratory of Department of Pathology & Laboratory Medicine and Genomic Core	Cedars-Sinai Medical Center, Molecular Pathology Laboratory of Department of Pathology & Laboratory Medicine and Genomic Core	Brian Davis; Eric Vail; Jasmine T Plummer; Jorge Mario Sincuir Martinez; Stephanie Chen; Wenjuan Zhang
EPI_ISL_7809634	Colorado Department of Public Health and Environment	Colorado Department of Public Health and Environment	Alexandria Rossheim; Diana Ir; Emily A. Travanty; Laura Bankers; Mandy Waters; Michael A. Martin; Molly C. Hetherington-Rauth; Sarah Elizabeth Totten; Shannon R. Matzinger
EPI_ISL_8357381, EPI_ISL_8357385, EPI_ISL_8357389	Compensar Calle 26	LSPSDS	Alejandro Gomez Lopez; Gabriela Delgado Murcia; Johana Hernandez Toloza; Marcela Mercado
EPI_ISL_8357369	Compensar Calle 63	LSPSDS	Alejandro Gomez Lopez; Gabriela Delgado Murcia; Johana Hernandez Toloza; Marcela Mercado
EPI_ISL_6941250, EPI_ISL_7039253	DASA	DASA	Adriano Bonaldi; Angelica Hristov; Annelise Lopes; Bianca Cota; Camila Romano; Cristina Oliveira; Jose Levi; Lidia Yamamoto; Luciane Sussuchi; Paulo Pierry; Rodrigo Guarischi; Rodrigo Salazar
EPI_ISL_7808713	DeRisi Lab, University of California, San Francisco	DeRisi Lab, University of California, San Francisco	Amy Kistler; Anthea Mitchell; Carina Marquez; Diane Havlir; Diane Jones; Douglas Black; Eric Chow; Gabriel Chamie; Genay Pilarowski; Grace Wang; IDseq Team; Jackie Martinez; James Peng; Jamin Liu; John Schrom; Jon Jacobo; Lucy Li; Luis Rubio; Manu Vanaerschot; Matthew Laurie; Matthias Hapte-Selassie; Maya Petersen; Patrick Ayscue; Sabrina Mann; Sara Sunshine; Susana Rojas; Susy Rojas; Valerie Tulier-Laiwa; and Joseph DeRisi
EPI_ISL_7908699, EPI_ISL_7912756	Department of Bacteria, Parasites and Fungi, Statens Serum Institut, Copenhagen, Denmark	Statens Serum Institut Bioinformatics and Microbial Genomics	Danish Covid-19 Genome Consortium
EPI_ISL_7747681	Emory Molecular Diagnostics Laboratory, Emory Healthcare	Piantadosi Lab, Emory Department of Pathology	Ahmed Babiker; Anne Piantadosi; Dara Khosravi
EPI_ISL_7319328, EPI_ISL_7319464	Florida Bureau of Public Health Laboratories	Florida Bureau of Public Health Laboratories	Jason Blanton; Namratha Tarigopula; Sarah Schmedes; Tiffany Splatt
EPI_ISL_6620229	Fulgent Genetics	Centers for Disease Control and Prevention Division of Viral Diseases, Pathogen Discovery	Becky Tsai; Benafsh Sapra; Benjamin Rambo-Martin; Christopher Gulvick; Clinton Paden; Dakota Howard; Dhwani Batra; Doreen Ng; Duncan MacCannell; Erisa Sula; Harry Gao; James Xie; Jason Caravas; John Gao; Joseph Fierro; Kristine Lacek; Matthew Schmerer; Mickey Li; Peter Cook; Scott Sammons; Shatavia Morrison; Tymeckia Kendall; Victoria Caban Figueroa; Yan Meng; Yvette Unoarumhi
EPI_ISL_6675476, EPI_ISL_6675503, EPI_ISL_6675612, EPI_ISL_6675615, EPI_ISL_6675619, EPI_ISL_6675620, EPI_ISL_6675624, EPI_ISL_6675673, EPI_ISL_6675694, EPI_ISL_6675857, EPI_ISL_7778190, EPI_ISL_7778208, EPI_ISL_8187693			
see above	Genetica Molecular and Subdepartamento de Virologia ISP Chile	Instituto de Salud Publica de Chile	Andres Castillo; Barbara Parra; Constanza Campano; Gisselle Barra; Javier Tognarelli; Jorge Fernandez; Karen Orostica; Loredana Arata; Patricia Bustos; Rodrigo Fasce; Soledad Ulloa
EPI_ISL_7307270, EPI_ISL_7476228, EPI_ISL_7476314, EPI_ISL_7476330, EPI_ISL_7476368, EPI_ISL_7476423, EPI_ISL_7476449, EPI_ISL_7476483, EPI_ISL_7476510, EPI_ISL_7476523, EPI_ISL_7476597, EPI_ISL_7476651, EPI_ISL_7476741, EPI_ISL_7476758, EPI_ISL_7476784, EPI_ISL_7476794, EPI_ISL_7476796, EPI_ISL_7476801			
see above	Grupo de Investigación en Enfermedades Tropicales del Ejército (GINETEJ), Laboratorio de Referencia e Investigación, Dirección de Sanidad Ejército, Bogotá, Colombia	Centro de Investigaciones en Microbiología y Biotecnología-UR (CIMBIUR), Facultad de Ciencias Naturales, Universidad del Rosario, Bogotá, Colombia	Alberto E. Paniz-Mondolfi; Alberto Paniz-Mondolfi; Angie L. Ramírez; Angie Ramírez; Beatriz Ariza; Camilo A. Correa-Cárdenas; Camilo Correa-Cardenas; Carlos Gómez-Restrepo; Claudia Cardozo-Romero; Claudia Mendez; Claudia Méndez; David-Santiago Quevedo; Enzo Guerrero-Araya; Guido España; Hernando Díaz; Juan David Ramírez; Juliana Cuervo-Rojas; Julie Perez; Julie Pérez; Luz H. Patiño; Manuel-Antonio Franco; Maria Clara Duque; Maria-Clara Duque; Marina Muñoz; Nathalia Ballesteros; Nicolas Luna; Nicolás Luna; Sergio Castañeda; Zulma M. Cucunubá
EPI_ISL_6945580	HOSPITAL UNIVERSITARIO SAN IGNACIO	Centro de Genética y Biología Molecular - Universidad del Magdalena	Andrea M. Ramírez Hernandez; Angel Oviedo Marquez; Daniel Bautista; Edison Lea-Ch; Lyda R. Castro; Maria Teresa Mojica-Ortiz
EPI_ISL_8181151	Helix	Centers for Disease Control and Prevention Division of Viral Diseases, Pathogen Discovery	Benjamin Rambo-Martin; Christopher Gulvick; Clinton Paden; Dakota Howard; Dhwani Batra; Duncan MacCannell; Erisa Sula; Helix CA; Jason Caravas; Kristine Lacek; Matthew Schmerer; Peter Cook; Scott Sammons; Shatavia Morrison; Tymeckia Kendall; Victoria Caban Figueroa; Yvette Unoarumhi
EPI_ISL_7711705	Hospital Dr. Carlos Luis Valverde Vega	Universidad de Costa Rica e Instituto Costarricense de Investigacion y Ensenanza en Nutricion y Salud	Brenes Hebleen; Camacho Erwin; Campos-Sanchez Rebeca; Ceballos Ana; Cordero Estela; Cristancho Marco; Duarte Francisco; Fernandez-Do Porto Dario; Herrera-Estrella Alfredo; Jimenez-Moraila Beatriz; Kreuze Jan; Mireles-Rivera Guadalupe; Molina-Mora Jose; Munoz-Medina Jose Esteban; Negri Tatiana; Nunes Gisele; Oliveira Guilherme; Oliveira Renato; Reales-Gonzalez Jhonnatan; Remes-Lenicov Federico; Reyes Alejandro; Sosa Ezequiel; Soto Claudio; Tsukayama Pablo; Turjanski Adrian; and Zayat Jonathan
EPI_ISL_7711704	Hospital Mexico	Universidad de Costa Rica e Instituto Costarricense de Investigacion y Ensenanza en Nutricion y Salud	Brenes Hebleen; Camacho Erwin; Campos-Sanchez Rebeca; Ceballos Ana; Cordero Estela; Cristancho Marco; Duarte Francisco; Fernandez-Do Porto Dario; Herrera-Estrella Alfredo; Jimenez-Moraila Beatriz; Kreuze Jan; Mireles-Rivera Guadalupe; Molina-Mora Jose; Munoz-Medina Jose Esteban; Negri Tatiana; Nunes Gisele; Oliveira Guilherme; Oliveira Renato; Reales-Gonzalez Jhonnatan; Remes-Lenicov Federico; Reyes Alejandro; Sosa Ezequiel; Soto Claudio; Tsukayama Pablo; Turjanski Adrian; and Zayat Jonathan
EPI_ISL_7606117, EPI_ISL_7606119	Hospital Nacional de Niños	Inciensa, Instituto Costarricense de Investigación y Enseñanza en Nutrición y Salud	Cristian Pérez-Corrales
EPI_ISL_7711706	Hospital San Francisco De Asis [San Isidro/Grecia/Alajuela]	Universidad de Costa Rica e Instituto Costarricense de Investigacion y Ensenanza en Nutricion y Salud	Brenes Hebleen; Camacho Erwin; Campos-Sanchez Rebeca; Ceballos Ana; Cordero Estela; Cristancho Marco; Duarte Francisco; Fernandez-Do Porto Dario; Herrera-Estrella Alfredo; Jimenez-Moraila Beatriz; Kreuze Jan; Mireles-Rivera Guadalupe; Molina-Mora Jose; Munoz-Medina Jose Esteban; Negri Tatiana; Nunes Gisele; Oliveira Guilherme; Oliveira Renato; Reales-Gonzalez Jhonnatan; Remes-Lenicov Federico; Reyes Alejandro; Sosa Ezequiel; Soto Claudio; Tsukayama Pablo; Turjanski Adrian; and Zayat Jonathan
EPI_ISL_7211378	Hospital Universitari Dr. Josep Trueta	Institut d'Investigació Biomèdica	Bernat del Olmo; Mel·lina Pinsach; Meritxell Deulofeu; Nuria Esther Neto; Paula Costa

		de Girona | Hospital Universitari Dr. Josep Trueta	
EPI_ISL_7307162, EPI_ISL_7307166, EPI_ISL_7307182, EPI_ISL_7307194, EPI_ISL_7307198, EPI_ISL_7307202, EPI_ISL_7307215, EPI_ISL_7307223, EPI_ISL_7307227, EPI_ISL_7307233, EPI_ISL_7307280, EPI_ISL_7307292, EPI_ISL_7339454, EPI_ISL_7339455, EPI_ISL_7339458, EPI_ISL_7339470, EPI_ISL_7829667			
see above	Hospital Universitario San Ignacio	Centro de Investigaciones en Microbiología y Biotecnología-UR (CIMBIUR), Facultad de Ciencias Naturales, Universidad del Rosario, Bogotá, Colombia	Alberto Paniz-Mondolfi; Angie Ramírez; Beatriz Ariza; Beatriz Elena Ariza; Camilo A. Correa-Cárdenas; Carlos Gómez-Restrepo; Claudia Cardozo-Romero; Claudia Cecilia Cardozo; Claudia Méndez; David-Santiago Quevedo; Guido España; Hernando Díaz; Juan David Ramírez; Juliana Cuervo-Rojas; Juliana Maria Cuervo; Julie Pérez; Luz H. Patiño; Manuel Antonio Franco; Manuel-Antonio Franco; Maria-Clara Duque; Marina Muñoz; Nathalia Ballesteros; Nicolas Luna; Sandra Liliana Valderrama; Sergio Castañeda; Zulma M. Cucunubá; Zulma Milena Cucunubá
EPI_ISL_8033762	IHU Mediterranee Infection	IHU Mediterranee Infection	Bernard La Scola; Philippe Colson; et al.
EPI_ISL_8097176, EPI_ISL_8097183, EPI_ISL_8097185	IIPSI SOL WAYUU	Instituto Nacional de Salud-Dirección de Investigación en Salud Pública	Beatriz de Arco; Carlos Franco-Muñoz; Diego A. Álvarez-Díaz; Diego Andrés Prada; Dioselina Peláez-Carvajal; Gerardo Santamaría; Héctor Alejandro Ruiz-Moreno; Jhonnatan Reales-González; Jorge Rivera; Julián Naizaque; Katherine Laiton-Donato; Marcela Mercado-Reyes.; Martha Lucia Ospina Martínez; María T. Herrera-Sepúlveda; Paola Rojas-Estevez; Sheryll Corchuelo; Tatiana Cobos
EPI_ISL_6976265, EPI_ISL_6989642, EPI_ISL_6990137, EPI_ISL_6990142, EPI_ISL_7829649, EPI_ISL_7829658, EPI_ISL_7831126, EPI_ISL_7831406, EPI_ISL_7831409			
see above	IVIC	Laboratorio de Virología Molecular	Carmen L Loureiro; CoViMol Group; CoViVen Group; Domingo J Garzaro; Flor H Pujol; Héctor R Rangel; José Luis Zambrano; Lieska Rodríguez; Mariana Hidalgo; Pierina D´Angelo; Rossana C Jaspe; Víctor Alarcón; Yoneira Sulbaran; Zoila Moros
EPI_ISL_7709464, EPI_ISL_7709473, EPI_ISL_7709474, EPI_ISL_8183444, EPI_ISL_8183483	Institute of Microbiology, Universidad San Francisco de Quito	Institute of Microbiology, Universidad San Francisco de Quito	Belén Prado-Vivar; Bernardo Gutiérrez; Erika B. Muñoz; Fernanda Zurita; Gabriel Trueba; Juan Jose Villacís; Juan José Guadalupe; Mateo Carvajal; Michelle Grunauer; Monica Becerra-Wong; Nelson Montalvan; Patricio Rojas-Silva; Paúl Cárdenas; Sully Márquez; Verónica Barragán; Verónica Pacheco
EPI_ISL_7813104	Instituto Mexicano del Seguro Social (IMSS)	CABANA: Unidad de Genómica Avanzada del Centro de Investigación y de Estudios Avanzados (UGA-LANGEBIO, CINVESTAV)	Adrián Turjanski; Alejandro Reyes; Alfredo Herrera-Estrella; Ana Ceballos; Beatriz Jiménez-Moraila; Claudio Soto; Darío Fernández-Do Porto; Erwin Camacho; Estela Cordero; Ezequiel Sosa; Federico Remes-Lenicov; Francisco Duarte; Gisele Nunes; Guadalupe Mireles-Rivera; Guilherme Oliveira; Hebleen Brenes; Jan Kreuze; Jhonnatan Reales-González; Jonathan Zayat; José Esteban Muñoz-Medina; José Molina-Mora; Marco Cristancho; Pablo Tsukayama; Rebeca Campos-Sánchez; Renato Oliveira; Tatiana Negri
EPI_ISL_7747261	Instituto Nacional de Enfermedades Respiratorias (INER)	Centro de Investigación en Enfermedades Infecciosas (CIENI), Instituto Nacional de Enfermedades Respiratorias (INER)	Alejandra García-Gasca; Alejandra Hernández-Terán; Alejandro Sánchez-Flores; Alfredo Herrera-Estrella; Alicia Ocaña-Mondragón; Andreu Comas-García; Angel Gustavo Salas-Lais; Antonio Loza Román; Bernardo Martínez-Miguel; Blanca Taboada; Brenda Irasema Maldonado-Meza; Bruno Gómez-Gil; Carla Ivón Herrera-Najera; Carlos F. Arias; Celia Boukadida; Clara Esperanza Santacruz-Tinoco; Concepción Grajales-Muñiz; Consorcio Mexicano de Vigilancia Genómica (CoViGen-Mex). Authors (in alphabetical order): Julio Elias Alvarado-Yaah; Cristóbal Cháidez-Quiróz; Célida Duque Molina; Célida Martínez-Rodríguez; Daniel Fregoso-Rueda; Daniel Lira Morales; Eduardo Becerril-Vargas; Eduardo Rivera-Martínez; Fernando Fontove-Herrera; Fidencio Mejía-Nepomuceno; Francisco Pulido; Gabriel Chavira-Trujillo; Gloria Elena Espinosa-Ayala; Gloria María Molina-Salinas; Gloria Vazquez; Hector Montoya-Fuentes; Helen Haydee Fernanda Ramírez-Plascencia; Irvin González-López; Jean Pierre González; Jesús Hernández; Joel Armando Vázquez-Pérez.; Jorge Salas-Hernández; José Antonio Enciso-Moreno; José Arturo Martínez-Orozco; José Esteban Muñoz-Medina; José de Jesús Nuñez-Contreras; Juan Bautista Chale-Dzul; Julissa Enciso-Ibarra; Kathia Elizabeth Tapia-Diaz; Luis Alberto Ochoa-Carrera; Margarita Matías-Florentino; Mario Mújica-Sánchez; Marissa Perez-Garcia; María Eugenia Jiménez-Corona; María Guadalupe Santiago-Mauricio; María Guadalupe de Jesús Mireles-Rivera; Nelly Sélem-Mojica; Pavel Isa; Ricardo Ciria Merce; Ricardo Grande; Rosa María Gutiérrez Rios; Rosario Vazquez-Larios; Santiago Ávila-Ríos; Selene Zárate; Susana Lopez; Verónica Mata-Haro; Victor Eduardo García-Arias; Víctor Hugo Borja-Aburto
EPI_ISL_7951686	LABORATORIO CLINICO COMPENSAR LOS COBOS	Instituto Nacional de Salud-Dirección de Investigación en Salud Pública	Beatriz de Arco; Carlos Franco-Muñoz; Diego A. Álvarez-Díaz; Diego Andrés Prada; Dioselina Peláez-Carvajal; Gerardo Santamaría; Héctor Alejandro Ruiz-Moreno; Jhonnatan Reales-González; Jorge Rivera; Julián Naizaque; Katherine Laiton-Donato; Marcela Mercado-Reyes.; Martha Lucia Ospina Martínez; María T. Herrera-Sepúlveda; Paola Rojas-Estevez; Sheryll Corchuelo; Tatiana Cobos
EPI_ISL_8357393, EPI_ISL_8357394, EPI_ISL_8357396, EPI_ISL_8357403	LABORATORIO LORENA VEJARANO SAS	LSPSDS	Alejandro Gomez Lopez; Gabriela Delgado Murcia; Johana Hernandez Toloza; Marcela Mercado
EPI_ISL_8357466	LSP Distrital Bogota	LSPSDS	Alejandro Gomez Lopez; Gabriela Delgado Murcia; Johana Hernandez Toloza; Marcela Mercado
EPI_ISL_6769061	Lab. Virología y Genética Universidad Simón Bolívar	Laboratorio de Biologia Molecular, Universidad Cooperativa de Colombia, Santa Marta	Andrew S. Muñoz-Gamba; Antonio Acosta; Camila Gonzalez; Daniel B. Ramírez-Osorio; Johana Zuñiga; José A. Usme-Ciro; Paula A. Quintero-Cortés; Yesid Bello
EPI_ISL_8178768	Laboratoire de Recherche et d'Analyses Médicales de la Gendarmerie Royale	Laboratoire de Recherche et d'Analyses Médicales de la Gendarmerie Royale	Amal SOUIRI; Hajar LEMRISS; Mohamed LABIOUI; Mohamed Mouatakid; Nabil LEMZAOUI; Sanaâ LEMRISS; Saâd EL KABBAJ
EPI_ISL_7662410, EPI_ISL_7662503, EPI_ISL_7662506, EPI_ISL_7664072, EPI_ISL_7664074, EPI_ISL_7664076, EPI_ISL_7664078			
see above	Laboratoire de santé publique du Québec	Laboratoire de santé publique du Québec	Guillaume Bourque; Ioannis Ragoussis; Jesse Shapiro; Mark Lathrop and Judith Fafard on behalf of the CoVSeQ research group; Sandrine Moreira
EPI_ISL_8408362, EPI_ISL_8408363, EPI_ISL_8408365, EPI_ISL_8408370, EPI_ISL_8408371, EPI_ISL_8408373	Laboratorio Central, Ministerio de Salud Córdoba	Fundacion para el Progreso de la Medicina and Instituto de Patologia Vegetal (IPAVE-CIAP-INTA) on behalf of 'Proyecto Argentino Interinstitucional de genomica de SARS-CoV-2' (PAIS Consortium)	Barbas, G.; Castro, G.; Debat, H.; F.D.; Fernández; Goya, S.; Lucca, A.; M.B.; Marquez, N.; Pisano; Re, V.; Sicilia, P.; Zeballos, M.
EPI_ISL_7961381, EPI_ISL_7961386	Laboratorio Referencial Regional en Salud Pública - GERESA Lambayeque	Laboratorio de Genómica Microbiana, Universidad Peruana Cayetano Heredia	Alejandra Dávila-Barclay; Cristian Díaz Vélez; Diego Cuicapuza; Guillermo Salvatierra; Gustavo Adolfo Sandoval Peña; Janet Huancachoque; Luis Miguel Serquén Lopez; Pablo Tsukayama; Richard Montalvo Aguirre; Ronald Milian Pérez; Sergio Luis Aguilar Martínez
EPI_ISL_8217527	Laboratorio SYNLAB Colombia	"Laboratorio de biotecnología, Universidad Icesi"	"María I. Gutiérrez López; Adrián Camilo Rodríguez Ararat; Diana M. Florez Giraldo; Marcela Mercado; Maria F. Villegas Torres; Paola A. Caicedo Burbano"; Programa Nacional de Caracterización Genómica de SARS-CoV-2
EPI_ISL_6703229, EPI_ISL_6703248	Laboratorio SYNLAB Colombia	CIAT, Laboratorio de Virologia	Ana M. Leiva; Diana Lopez-Alvarez; Programa Nacional de Caracterización Genómica de SARS-CoV-2; WilmerJ. Cuellar
EPI_ISL_7961384, EPI_ISL_7961385	Laboratorio de Genómica Microbiana - Universidad Peruana Cayetano Heredia	Laboratorio de Genómica Microbiana, Universidad Peruana Cayetano Heredia	Brenes Hebleen; Camacho Erwin; Campos-Sánchez Rebeca; Ceballos Ana; Cordero Estela; Cristancho Marco; Diego Cuicapuza; Duarte Francisco; Fernández-Do Porto Darío; Guillermo Salvatierra; Herrera-Estrella Alfredo; Janet Huancachoque; Jiménez-Moraila Beatriz; Kreuze Jan; Mireles-Rivera Guadalupe; Molina-Mora José; Muñoz-Medina José Esteban; Negri Tatiana; Nunes Gisele; Oliveira Guilherme; Oliveira Renato; Pedro E. Romero; Reales-González Jhonnatan; Remes-Lenicov Federico; Reyes Alejandro; Segundo Fuentes; Sosa Ezequiel; Soto Claudio; Tsukayama Pablo; Turjanski Adrián; and Zayat Jonathan / Alejandra Dávila-Barclay
EPI_ISL_6574179, EPI_ISL_6574229, EPI_ISL_6574318, EPI_ISL_6689772, EPI_ISL_6689773, EPI_ISL_6689774, EPI_ISL_6689775, EPI_ISL_6689842, EPI_ISL_6946002, EPI_ISL_6946040, EPI_ISL_6946056, EPI_ISL_7494925, EPI_ISL_7494926, EPI_ISL_7494931, EPI_ISL_7494932, EPI_ISL_7494933, EPI_ISL_7494934, EPI_ISL_7494935, EPI_ISL_7494936, EPI_ISL_7494937, EPI_ISL_7846229, EPI_ISL_7846327, EPI_ISL_7846353, EPI_ISL_8143835			
see above	Laboratorio de Referencia Nacional de Virus Respiratorios. Centro Nacional de Salud Publica. Instituto Nacional de Salud Peru.	Laboratorio de Referencia Nacional de Virus Respiratorios. Centro Nacional de Salud Publica. Instituto Nacional de Salud Peru.	Alicia Nuñez Llanos; Carlos Padilla Rojas; Edwart Rogger Rivera Serrano; Henri Bailon Calderon; Iris Silva Molina; Joseph Huayra Niquen; Kelly Vanessa Izarra Rojas; Lely Solari Zerpa; Luis Barcena Flores; Marco Galarza Perez; Nancy Rojas Serrano; Nieves Sevilla Castañeda; Omar Caceres Rey; Orson Mestanza Millones; Princesa Medrano Alhuay; Priscila Lope Pari; Sara Gordillo Vilchez; Steve Acedo Lazo; Veronica Hurtado Vela; Victor Jimenez Vasquez; Wendy Lizarraga Olivares
EPI_ISL_7747330, EPI_ISL_7747336, EPI_ISL_7747351, EPI_ISL_7747353, EPI_ISL_7747355, EPI_ISL_7747361, EPI_ISL_7747363, EPI_ISL_7747364, EPI_ISL_7747366, EPI_ISL_7747370, EPI_ISL_7747372, EPI_ISL_7747374, EPI_ISL_7747377, EPI_ISL_7747381, EPI_ISL_7747385, EPI_ISL_7747386, EPI_ISL_7747389, EPI_ISL_7747391, EPI_ISL_7747392, EPI_ISL_7747394, EPI_ISL_7747418, EPI_ISL_7747440, EPI_ISL_7835287, EPI_ISL_7835297			
see above	Laboratorio de Salud Pública de Bogotá	Gencore - Universidad de los Andes	Alejandro Gomez; Cristian Barrera; Gabriela Ariza; Gabriela Delgado; Johana Hernandez; Luisa Sacristan; Marcela Guevara; Silvia Restrepo
EPI_ISL_7719094	Laboratorio di Microbiologia e Virologia, Università Vita-Salute San Raffaele, Milano	Laboratorio di Microbiologia e Virologia, Università Vita-Salute San Raffaele, Milano	Elena Criscuolo; Enzo Boeri; Massimo Clementi; Massimo Locatelli; Matteo Castelli; Michela Sampaolo; Nicasio Mancini; Nicola Clementi; Roberta Antonia Diotti; Roberto Ferrarese
EPI_ISL_8264091, EPI_ISL_8367942, EPI_ISL_8469117, EPI_ISL_8469736	Laboratory Corporation of America	Centers for Disease Control and Prevention Division of Viral Diseases, Pathogen Discovery	Amanda Douglas; Amanda Suchanek; Andrea Throop; Ayla Burns; Benjamin Rambo-Martin; Bobbi Croy; Brian Krueger; Brian Norvell; Christopher Gulvick; Christos Petropoulos; Clinton Paden; Craig Lukasik; Dakota Howard; Debbie Boles; Dhwani Batra; Duncan MacCannell; Eyad Almasri; Goran Stevovic; Howard Engler; Hrushikesh Deshmukh; Jake Humphrey; Jana Schroth; Jason Caravas; Joe Voshell; John Pruitt; Jonathan Meltzer; Jonathan Williams; Kimberly Wagner; Kristine Lacek; Lax Iyer; Lisa Pfefferle; Lyndon Tilson; Manoj Jain; Marcia Eisenberg; Mary Cristobal; Mary Williamson; Matthew Robinson; Matthew Schmerer; Michael Levandoski; Mike Sapeta; Mindy Nye; Minoo Agarwal; Mohan Kolli; Nuthawin Charoensri; Oren Cohen; Peter Cook; Prashant Gupta; Qian Zeng; Rama Ghatti; Scott Parker; Scott Ryan; Scott Sammons; Shatavia Morrison; Stanley Letovsky; Steven Ragan; Suresh Selvaraju; Susan Countryman; Susan Hicks; Suzanne Dale; Thomas Urban; Tim Kuphal; Tricia Zwiefelhofer; Tymeckia Kendall; Victoria Caban Figueroa; Vincent Drouillon; Yvette Unoarumhi
EPI_ISL_7613697	Maasstad Ziekenhuis	Maasstad ziekenhuis	Pieter Smit
EPI_ISL_7462685	Molecular Virology Unit, Microbiology and Virology Depertment, Fondazione IRCCS Policlinico San Matteo, Pavia	Molecular Virology Unit, Microbiology and Virology Depertment, Fondazione IRCCS Policlinico San Matteo, Pavia	Antonio Piralla; Fausto Baldanti; Federica Giardina; Guglielmo Ferrari; Stefano Gaiarsa
EPI_ISL_7711154	NJDOH, Public Health and Environmental Laboratories	NJ_PHEL	Allison Roder; Byeong Jeong; Chelsea San Filippo; Dana Woell; Jacquelyn Deverell; Lindsey Bodnar; Maria-Magdalene Pugliese; Mohammad M. Ali; Ryan Pachucki; Shiv K. Verma
EPI_ISL_7263474	National Platform bis COVID ULB-IBC	National Platform bis COVID ULB-IBC	Arnaud Marchant; Benoit Haerlingen; Coralie Henin; Marie-Luce Delforge; Ricardo De Mendonça
EPI_ISL_6631981, EPI_ISL_6632036, EPI_ISL_6632044, EPI_ISL_6632077, EPI_ISL_6632100, EPI_ISL_6632101, EPI_ISL_6632107, EPI_ISL_6632112, EPI_ISL_6632116, EPI_ISL_6632119, EPI_ISL_6632127, EPI_ISL_6632131, EPI_ISL_6632132, EPI_ISL_6632133, EPI_ISL_6632134, EPI_ISL_6632135, EPI_ISL_6632142, EPI_ISL_6632149, EPI_ISL_6632161, EPI_ISL_6632166, EPI_ISL_6632172, EPI_ISL_6632179, EPI_ISL_6632185			
see above	National Public Health Laboratory, Ministry of Health & Wellness	Viral Special Pathogens Branch, Centers for Disease Control and	Antoni Comrie; Carl Bruce; Emir Talundzic; Joel Montgomery; John Klena; Justin Lee; Karen Webster-Kerr; Melissa Mobley; Michelle Brown; Michelle Hamilton; Monica Smikle; Nathlee McMorris; Sandra Jackson; Shannon Whitmer; Suwanie Lewis

		Prevention	
EPI_ISL_8133617	Northumbria University / South Tees Hospitals NHS Foundation Trust / North Cumbria Integrated Care NHS Foundation Trust / North Tees and Hartlepool NHS Foundation Trust / Newcastle Hospitals NHS Foundation Trust	COVID-19 Genomics UK (COG-UK) Consortium	Andrew Nelson; Brendan Payne; Clive Graham; Darren L Smith; Debra Padgett; Edward Barton; Emma Swindells; Garren Scott; Gary Black; Gary Eltringham; Giles S Holt; Greg R Young; Jane Greenaway; Jennifer Collins; John Allan; Joshua Loh; Lynn Dover; Matthew Bashton; Mohammad A Tariq; Paul Baker; Sarah Essex; Steve Liggett; Wen C Yew; Yusri Taha
EPI_ISL_7623977	OHSU Lab Services Molecular Microbiology Lab	Oregon SARS-CoV-2 Genome Sequencing Center	Alec J. Hirsch; Andrew C. Adey; Benjamin N. Bimber; Brendan L. O'Connell; Brian J. O'Roak; Cierra LaBlanc; Daniel N. Streblow; Destine Krenik; Donna Hansel; Guang Fan; Rabeka Ali; Ruth V. Nichols; Sonia Acharya; William B. Messer; Xuan Qin
EPI_ISL_7602560, EPI_ISL_7851219, EPI_ISL_8094966	Pandemic Response Lab - NYC	Pandemic Response Lab, R&D	Alex Carpio; Cybill del Castillo; Dylan Law; Haiping Hao; Henry Lee; Isabel Fernandez Escapa; Jon Laurent; Melissa Hopkins; Michael Hammerling; Pradeep Bugga; Shinyoung Clair Kang; Sol Rey; William Ward
EPI_ISL_8323023, EPI_ISL_8323077	Public Health Ontario Laboratory	Public Health Ontario Laboratory	Aimin Li; Alireza Eshaghi; Andre Villegas; Ashleigh Sullivan; Christine Frantz; Dean Maxwell; Esha Joshi; Jared Simpson; Jennifer L Guthrie; Jonathan B Gubbay; Karthikeyan Sivaraman; Lawrence Heisler; Matthew Watson; Michael CY Li; Michael Laszloffy; Nahuel Fittipaldi; Philip Banh; Richard de Borja; Samir N Patel; Sandeep Nagra; Sandra Zittermann; Sarah Teatero; Vanessa G Allen; Yao Chen; Yogi Sundaravadanam
EPI_ISL_8044419	Quadram Institute Bioscience	COVID-19 Genomics UK (COG-UK) Consortium	Alexander J Trotter; Alison E. Mather; Alp Aydin; Ana P. Tedim; Anastasia Kolyva; Andrew Bell; Andrew J. Page; Christopher Jeanes; Claire Stuart; Dave J. Baker; Ebenezer Foster-Nyarko; Gemma L. Kay; John Wain; Justin O'Grady; Leonardo de Oliveira Martins; Lewis G. Spurgin; Lindsay Coupland; Lizzie Meadows; Luke Bedford; Maria Diaz; Mark Webber; Martin Lott; Michaela Matthews; Muhammed Yasir; Nabil-Fareed Alikhan; Ngozi Elumogo; Nicholas M. Thomson; Rachael Stanley; Rachel Gilroy; Reenesh Prakash; Rose K Davidson; Samir Dervisevic; Samuel Bloomfield; Sophie J. Prosolek; Steven Rudder; Thanh Le-Viet
EPI_ISL_6783400, EPI_ISL_7936987	Quest Diagnostics Incorporated	Centers for Disease Control and Prevention Division of Viral Diseases, Pathogen Discovery	A. Gerasimova; A. Perez; B. Anderson; Benjamin Rambo-Martin; Christopher Gulvick; Clinton Paden; Dakota Howard; Dhwani Batra; Duncan MacCannell; Erisa Sula; F. Lacbawan; I. Shlyakhter; Jason Caravas; K. Livingston; Kristine Lacek; L. Bernstein; M. Hua; Matthew Schmerer; P. Tanpaiboon; Peter Cook; R. Kagan; R. Owen; R. Rolando; S. Rosenthal; Scott Sammons; Shatavia Morrison; Tymeckia Kendall; Victoria Caban Figueroa; Y. Liu; Yvette Unoarumhi
EPI_ISL_7166193	Respiratory Virus Unit, Microbiology Services Colindale, Public Health England	COVID-19 Genomics UK (COG-UK) Consortium	PHE Covid Sequencing Team
EPI_ISL_7979718	SURA	Gencore - Universidad de los Andes	Cristian Barrera; David González; Gabriela Ariza; Luisa Sacristan; Marcela Guevara; Marcela Mercado; Silvia Restrepo
EPI_ISL_6811664	Salud Digna	Instituto Nacional de Medicina Genomica	Abraham Campos-Romero; Cedro-Tanda A; Cruz-Islas Jazmin; Escobar-Arrazola MA; Garnica-Lopez Dora; Herrera-Montalvo LA.; Hidalgo-Miranda A; Luna-Ruiz Marco; Mendoza-Vargas A; Moreno-Camacho José Luis; Ramirez-Vega O; Rangel-DeLeon D; Reyes-Grajeda JP; Rodriguez-Gallegos Jorge; Yair Alfaro-Mora
EPI_ISL_7751221, EPI_ISL_7751429, EPI_ISL_7751639, EPI_ISL_7753089, EPI_ISL_7753141, EPI_ISL_7753763	Servicio Virosis Respiratorias-Departamento Virologia-INEI	Instituto Nacional Enfermedades Infecciosas C.G.Malbran	Avaro M.; Baumeister E.; Benedetti E.; Campos J.; Cisterna D.; Dattero ME; De Belder D.; Haim MS.; Lorenzo F.; Molina V.; Perandones C.; Poklepovich T.; Pontoriero A.; Russo M.; Sanchez Loria J.; Tuduri E.
EPI_ISL_6694518, EPI_ISL_6694533, EPI_ISL_6694694, EPI_ISL_6694854, EPI_ISL_6694858, EPI_ISL_6694884, EPI_ISL_6694888, EPI_ISL_6694956, EPI_ISL_6695030, EPI_ISL_6695103			
see above	Servicio Virosis Respiratorias-Departamento Virología-INEI	Instituto Nacional Enfermedades Infecciosas C.G.Malbran	Avaro M.; Baumeister E.; Benedetti E.; Campos J.; Cisterna D.; Dattero ME; De Belder D.; Haim MS.; Lorenzo F.; Molina V.; Perandones C.; Poklepovich T.; Pontoriero A.; Russo M.; Sanchez Loria J.; Tuduri E.
EPI_ISL_7710663, EPI_ISL_7710667, EPI_ISL_7938382, EPI_ISL_7938405	Servicios Medicos Olimpus	Laboratorio de Biologia Molecular, Universidad Cooperativa de Colombia, Santa Marta	Andrew S. Muñoz-Gamba; Daniel B. Ramírez-Osorio; José A. Usme-Ciro; Mayerlin Vasquez Cardenas; Paula A. Quintero-Cortés; Pricelis Paulin Polanco Fontalvo
EPI_ISL_6941451, EPI_ISL_6941452, EPI_ISL_6941460, EPI_ISL_6941461, EPI_ISL_6941462, EPI_ISL_6941463, EPI_ISL_6941464, EPI_ISL_6941465, EPI_ISL_6941466, EPI_ISL_6941467, EPI_ISL_6941468, EPI_ISL_6941469, EPI_ISL_6941470, EPI_ISL_6941471, EPI_ISL_6941472, EPI_ISL_6941493, EPI_ISL_6941494, EPI_ISL_6941495, EPI_ISL_6941496, EPI_ISL_6941497, EPI_ISL_6941498, EPI_ISL_6941499, EPI_ISL_6941501, EPI_ISL_6941502, EPI_ISL_6941503, EPI_ISL_6941506			
see above	The Caribbean Public Health Agency	Carrington Lab, Department of Preclinical Sciences, Faculty of Medical Sciences, The University of the West Indies, St Augustine Campus	Anushka Ramjag; Arianne Brown-Jordan; Avery Hinds; Christine V. F. Carrington; Christopher Oura; Gabriel Escobar; Irad Potter; Jacquiline Bisesor-McKenzie; Nikita S. D. Sahadeo; Nuno Faria; Oliver Pybus; Risha Singh; Sarah Hill; Simone Keizer-Beache; Soren Nicholls; SueMin Nathaniel; Vernie Ramkissoon

We gratefully acknowledge the following Authors from the Originating laboratories responsible for obtaining the specimens, as well as the Submitting laboratories where the genome data were generated and shared via GISAID, on which this research is based.

All Submitters of data may be contacted directly via www.gisaid.org

Authors are sorted alphabetically.

Acknowledgement EPI_SET Identifier: EPI_SET_20220328wx

Accession ID	Originating Laboratory	Submitting Laboratory	Authors
EPI_ISL_2621218	ADILAB	Universidad Nacional de Colombia - Laboratorio Genómico One Health	Andres F. Cardona-Rios; Carlos Franco-Muñoz; Carolina Muñoz-Arango; Celeny Ortiz; Daniel O. Maldonado-Perez; Diego A. Álvarez-Díaz; Hector Alejandro Ruiz-Moreno; Idabely Betancur Ortiz; Jorge E. Osorio; Juan P. Hernandez-Ortiz; Karl A Ciuoderis; Katherine Laiton-Donato; Laura Silvana Perez; Lina M. Hurtado; Marcela Mercado-Reyes; Maria Angélica Maya; Maria Stella López; Rita Almanza Payares; Sandra Ines Cano; Simón Villegas Velásquez
EPI_ISL_2828007, EPI_ISL_2828008	AREA DE SALUD SAN RAMON	Inciensa, Instituto Costarricense de Investigación y Enseñanza en Nutrición y Salud	Adriana Godínez; Claudio Soto-Garita; Estela Cordero; Francisco Duarte; Hebleen Porras; José Luis Vargas; Mariela Gutiérrez & Joselyn Prado; Melany Calderón
EPI_ISL_2975142, EPI_ISL_2975350, EPI_ISL_2975351, EPI_ISL_2975352, EPI_ISL_2975353	AULSS 6 Euganea	Istituto Zooprofilattico Sperimentale delle Venezie	Adelaide Milani; Alessia Schivo; Alice Fusaro; Ambra Pastori; Annalisa Salviato; Antonia Ricci; Calogero Terregino; Edoardo Giussani; Elisa Palumbo; Erika Giorgia Quaranta; Isabella Monne; Luca Tassoni
EPI_ISL_2927986	AULSS 7 Pedemontana	Istituto Zooprofilattico Sperimentale delle Venezie	Adelaide Milani; Alessia Schivo; Alice Fusaro; Ambra Pastori; Annalisa Salviato; Antonia Ricci; Calogero Terregino; Edoardo Giussani; Elisa Palumbo; Erika Giorgia Quaranta; Isabella Monne; Luca Tassoni
EPI_ISL_2975200	AULSS 8 Berica	Istituto Zooprofilattico Sperimentale delle Venezie	Adelaide Milani; Alessia Schivo; Alice Fusaro; Ambra Pastori; Annalisa Salviato; Antonia Ricci; Calogero Terregino; Edoardo Giussani; Elisa Palumbo; Erika Giorgia Quaranta; Isabella Monne; Luca Tassoni
EPI_ISL_2651228	AYUDAS DIAGNOSTICAS SURA	Universidad Nacional de Colombia - Laboratorio Genómico One Health	Andres F. Cardona-Rios; Carlos Franco-Muñoz; Carolina Muñoz-Arango; Celeny Ortiz; Daniel O. Maldonado-Perez; Diego A. Álvarez-Díaz; Hector Alejandro Ruiz-Moreno; Idabely Betancur Ortiz; Jorge E. Osorio; Juan P. Hernandez-Ortiz; Karl A Ciuoderis; Katherine Laiton-Donato; Laura Silvana Perez; Lina M. Hurtado; Marcela Mercado-Reyes; Maria Angélica Maya; Maria Stella López; Rita Almanza Payares; Sandra Ines Cano; Simón Villegas Velásquez
EPI_ISL_2438080	Adilab	Universidad Nacional de Colombia - Laboratorio Genómico One Health	Andres F. Cardona-Rios; Carlos Franco-Muñoz; Carolina Muñoz-Arango; Celeny Ortiz; Daniel O. Maldonado-Perez; Diego A. Álvarez-Díaz; Hector Alejandro Ruiz-Moreno; Idabely Betancur Ortiz; Jorge E. Osorio; Juan P. Hernandez-Ortiz; Karl A Ciuoderis; Katherine Laiton-Donato; Laura Silvana Perez; Lina M. Hurtado; Marcela Mercado-Reyes; Maria Angélica Maya; Maria Stella López; Rita Almanza Payares; Sandra Ines Cano; Simón Villegas Velásquez
EPI_ISL_1562674	Aegis Sciences Corporation	Centers for Disease Control and Prevention Division of Viral Diseases, Pathogen Discovery	Adrian Paskey; Alec Vest; Benjamin Rambo-Martin; Christopher Gulvick; Clinton R. Paden; Cyndi Clark; Dakota Howard; Darlene Wagner; Dhwani Batra; Dillon Nall; Duncan MacCannell; Ethan Sanders; Holly Houdeshell; Jason Caravas; Kara Moser; Matthew Hardison; Matthew Schmerer; Ola Kvalvaag; Patrick Campbell; Peter W. Cook; Rob Case; Scott Sammons; Shatavia Morrison; Shaun Westlund; Vikramsinha Ghorpade; Yvette Unoarumhi
EPI_ISL_2694880	Affidea	Instituto Nacional de Saude (INSA)	Borges et al
EPI_ISL_2158266, EPI_ISL_2473689, EPI_ISL_2504002, EPI_ISL_2969595	Alaska State Virology Laboratory	Alaska State Virology Laboratory	Elva House; Jack Chen; Jacob Zidek; Lisa Smith; Ph.D.; Stephanie DeRonde
EPI_ISL_2086307	Arizona State Public Health Laboratory	Arizona State Public Health Laboratory	Jessica Escobar; Katherine Fullerton; Linda Getsinger; Nobuko Fukushima; Stacy White; Trung Huynh; Victor Waddell
EPI_ISL_2617632, EPI_ISL_2617633, EPI_ISL_2617634, EPI_ISL_2617635, EPI_ISL_2617636, EPI_ISL_2617637, EPI_ISL_2617638, EPI_ISL_2617639, EPI_ISL_2617640, EPI_ISL_2617641, EPI_ISL_2617642, EPI_ISL_2617643, EPI_ISL_2617644, EPI_ISL_2617645, EPI_ISL_2617646, EPI_ISL_2617647, EPI_ISL_2617648, EPI_ISL_2617649, EPI_ISL_2617650, EPI_ISL_2617651, EPI_ISL_2617652, EPI_ISL_2617653, EPI_ISL_2617654, EPI_ISL_2617655, EPI_ISL_2617656, EPI_ISL_2617657, EPI_ISL_2617658, EPI_ISL_2617659			
see above	Austrian Agency for Health and Food Safety (AGES)	Bergthaler laboratory, CeMM Research Center for Molecular Medicine of the Austrian Academy of Sciences	Andreas Bergthaler; Anna Schedl; Bekir Erguner; Benedikt Agerer; Christoph Bock; Fabian Amman; Jan Laine; Lukas Endler; Maelle Le Moing; Martin Senekowitsch; Matthew Thornton; Michael Schuster; Petr Triska; Thomas Penz
EPI_ISL_2438009	Ayudas diagnosticas SURA	Universidad Nacional de Colombia - Laboratorio Genómico One Health	Andres F. Cardona-Rios; Carlos Franco-Muñoz; Carolina Muñoz-Arango; Celeny Ortiz; Daniel O. Maldonado-Perez; Diego A. Álvarez-Díaz; Hector Alejandro Ruiz-Moreno; Idabely Betancur Ortiz; Jorge E. Osorio; Juan P. Hernandez-Ortiz; Karl A Ciuoderis; Katherine Laiton-Donato; Laura Silvana Perez; Lina M. Hurtado; Marcela Mercado-Reyes; Maria Angélica Maya; Maria Stella López; Rita Almanza Payares; Sandra Ines Cano; Simón Villegas Velásquez
EPI_ISL_2872589, EPI_ISL_2872590	Azienda Ospedaliero Universitaria di Sassari	AMES Centro Polidiagnostico Strumentale S.r.l.	Anna Puggioni; Bianca Paglietti; Caterina Serra; Claudia Piu; Elena Rimini; Erica Mura; Flavia Angioj; Gabriele Ibba; Giulia Rocca; Laura Firino; Rosalba Govoni; Salvatore Rubino; Sergio Uzzau; Vincenzo Lai
EPI_ISL_2372852, EPI_ISL_2550664, EPI_ISL_2550696	Azienda Sanitaria dell'Alto Adige Laboratorio Aziendale di Microbiologia e Virologia	Istituto di Genomica Applicata	Davide Scaglione; Eleonora Paparelli; Elisa Masi; Elisabetta Giacobazzi; Elisabetta Pagani; Gabriele Magris; Irena Jurman; Irene Bianconi; Michele Morgante; Stefanie Wieser; Vera Vendramin
EPI_ISL_431154	BIOMNIS EUROFINS IVRY	Department of Virology, Henri Mondor University Hospital, Assistance Publique Hôpitaux de Paris, Université Paris-Est Créteil, INSERM U955	Alexandre Soulier; Christophe Rodriguez; Elisabeth Trawinski; Guillaume Gricourt; Jean-Michel Pawlotsky; Melissa N'Debi; Slim Fourati; Vanessa Demontant
EPI_ISL_2321586	BIOREFERENCIA	Universidad Nacional de Colombia - Laboratorio Genómico One Health	Andres F. Cardona-Rios; Carlos Franco-Muñoz; Carolina Muñoz-Arango; Celeny Ortiz; Daniel O. Maldonado-Perez; Diego A. Álvarez-Díaz; Hector Alejandro Ruiz-Moreno; Idabely Betancur Ortiz; Jorge E. Osorio; Juan P. Hernandez-Ortiz; Karl A Ciuoderis; Katherine Laiton-Donato; Laura Silvana Perez; Lina M. Hurtado; Marcela Mercado-Reyes; Maria Angélica Maya; Maria Stella López; Rita Almanza Payares; Sandra Ines Cano; Simón Villegas Velásquez
EPI_ISL_2999927	Berkshire and Surrey Pathology Services Lighthouse Laboratory	Wellcome Sanger Institute for the COVID-19 Genomics UK (COG-UK) Consortium	Berkshire and Surrey Pathology Services Lighthouse Laboratory and Alex Alderton; Cordelia Langford; David K. Jackson; Dominic Kwiatkowski; Ewan Harrison; Ian Johnston; Jeffrey Barrett; John Sillitoe on behalf of the Wellcome Sanger Institute COVID-19 Surveillance Team; Roberto Amato; Sonia Goncalves
EPI_ISL_2536144	Bundeswehrkrankenhaus Ulm	Bundeswehr Institute of Microbiology	Alexandra Rehn; Enrico Georgi; Malena Bestehorn-Willmann; Markus Antwerpen; Mathias Walter; Mike Pillukat; Roman Wölfel; Sabine Zange
EPI_ISL_2621207	CENTROLAB	Universidad Nacional de Colombia - Laboratorio Genómico One Health	Andres F. Cardona-Rios; Carlos Franco-Muñoz; Carolina Muñoz-Arango; Celeny Ortiz; Daniel O. Maldonado-Perez; Diego A. Álvarez-Díaz; Hector Alejandro Ruiz-Moreno; Idabely Betancur Ortiz; Jorge E. Osorio; Juan P. Hernandez-Ortiz; Karl A Ciuoderis; Katherine Laiton-Donato; Laura Silvana Perez; Lina M. Hurtado; Marcela Mercado-Reyes; Maria Angélica Maya; Maria Stella López; Rita Almanza Payares; Sandra Ines Cano; Simón Villegas Velásquez
EPI_ISL_2695346	CHEDV	Instituto Nacional de Saude (INSA)	Borges et al
EPI_ISL_2796193	CHU Sao Joao, Porto	Instituto Nacional de Saude (INSA)	Borges et al
EPI_ISL_2835967	CHUGA-IBP-laboratoire de Virologie	IBP-laboratoire de virologie	Alban Caporossi; Anne Signori -Schmuck; Anne-Karen Faure; Aurélie Truffot; Benjamin Nemoz; Hugo Jardin; Julien Andréani; Julien Lupo; Léa Ponderand; Pascal Poignard; Patrice Morand; Raphaële Germi; Sylvie Larrat
EPI_ISL_2695100, EPI_ISL_2695101, EPI_ISL_2695102	CHULN - H Santa Maria	Instituto Nacional de Saude (INSA)	Borges et al
EPI_ISL_2500506, EPI_ISL_2500516, EPI_ISL_2622106	CHUV	Laboratory of genomics and metagenomics	Claire Bertelli; Damien Jacot; Gilbert Greub; Sébastien Aeby; Trestan Pillonel
EPI_ISL_3008830	Center of Advanced Studies and Technology, Molecular Genetics Laboratory	Center of Advanced Studies and Technology, Molecular Genetics Laboratory	Anaclerio Federico; De Fabritiis Simone
EPI_ISL_1970836, EPI_ISL_1970837	Centro de Investigación en Enfermedades Infecciosas (CIENI), Instituto Nacional de Enfermedades Respiratorias (INER)	Centro de Investigación en Enfermedades Infecciosas (CIENI), Instituto Nacional de Enfermedades Respiratorias (INER)	Alejandra Hernández-Terán; Alejandro Sanchez-Flores; Blanca Taboada; Carlos F. Arias; Celia Boukadida; Eduardo Becerril-Vargas; Fidencio Mejía-Nepomuceno; Francisco Pulido; Hector Esteban Paz-Juárez; Joel Armando Vázquez-Pérez and Consorcio Mexicano de Vigilancia Genómica (CoViGen-Mex); Jorge Salas-Hernández; José Arturo Martínez-Orozco; Margarita Matías-Florentino; Mario Mújica-Sánchez; Pavel Isa; Ricardo Grande; Santiago Ávila-Ríos
EPI_ISL_2434137, EPI_ISL_2927083, EPI_ISL_2966323	Cliniques universitaires Saint-Luc	UCLouvain/IREC/MBLG	Benoit Kabamba Mukadi; Bertrand Bearzatto; Jean Ruelle; Ophélie Simon
EPI_ISL_2784135	Curative Labs	Curative Labs	Elias L. Salfati; Eugenia Khorosheva; George Way; J.Cesar Ignacio-Espinoza; Janet Chen; Mikhail Hanewich-Hollatz; Nabjot Sandhu; Sophia Quasem; Vladimir Slepnev; Zhiyi Xie
EPI_ISL_1678824	Department of Medical Microbiology & Infection prevention, Amsterdam University Medical Centers location AMC	Department of Medical Microbiology & Infection prevention, Amsterdam University Medical Centers location AMC	Fokla Zorgdrager; Janke Schinkel; Marcel Jonges; Matthijs Welkers; Menno de Jong; Robin van Houdt; Sebastien Matamoros; Sjoerd Rebers
EPI_ISL_1868200, EPI_ISL_1890559, EPI_ISL_2025772,	Department of Virus and Microbiological Special Diagnostics, Statens Serum	Aalborg University	Danish Covid-19 Genome Consortium

EPI_ISL_2296939, EPI_ISL_2298986	Institut, Copenhagen, Denmark		
EPI_ISL_2694746	Diagnostyka. Laboratoria Medyczne.	1. ViroGenetics - BSL3 Laboratory of Virology, Małopolska Centre of Biotechnology, Jagiellonian University; 2. Diagtron Laboratoria Łukasz Rąbalski	Gromowski, T.; Kowalski, M.; Labaj; Maciej Kosinski; Mazur-Panasiuk, N.; Natalia Derewonko; P.P.; Pyrc, K.; Rabalski L.; Rogalska-Kupiec M.; Swadzba J.; Sylwia Januszczak; Szulc, P.; Wydmanski, W.
EPI_ISL_2955372	Dianalabs SA	Genesupport	Geraldine Jost; Katia Jaton; Nadia Liassine; Tanguy ARAUD
EPI_ISL_2613602, EPI_ISL_2613603, EPI_ISL_2613604, EPI_ISL_2613605, EPI_ISL_2613606, EPI_ISL_2613607, EPI_ISL_2613608, EPI_ISL_2613609, EPI_ISL_2613610, EPI_ISL_2613611, EPI_ISL_2966247			
see above	Dipartimento di Medicina di Laboratorio, Azienda sanitaria universitaria Friuli Centrale (ASU FC)	Dipartimento di Medicina di Laboratorio, Azienda sanitaria universitaria Friuli Centrale (ASU FC)	Catia Mio; Chiara Dal Secco; Corrado Pipan; Francesco Curcio; Stefania Marzinotto
EPI_ISL_1970344, EPI_ISL_1970345, EPI_ISL_1970346, EPI_ISL_2483545, EPI_ISL_2483546, EPI_ISL_2483547, EPI_ISL_2887845			
see above	Dutch COVID-19 response team	Erasmus Medical Center	Anne van der Linden; Annemiek van der Eijk; Bas Oude Munnink; Corine GeurtsvanKessel; David Nieuwenhuijse; Emmanuelle Munger; Irina Chestakova; Marion Koopmans; Marjan Boter; Reina Sikkema; Richard Molenkamp; on behalf of the Dutch national COVID-19 respo; on behalf of the Dutch national COVID-19 response team.
EPI_ISL_1962951, EPI_ISL_1962952, EPI_ISL_2094359, EPI_ISL_2094360, EPI_ISL_2094361, EPI_ISL_2094362, EPI_ISL_2094363, EPI_ISL_2094364, EPI_ISL_2094365, EPI_ISL_2094366, EPI_ISL_2094367, EPI_ISL_2094371, EPI_ISL_2220450, EPI_ISL_2220453, EPI_ISL_2220456, EPI_ISL_2220458, EPI_ISL_2220460, EPI_ISL_2220461, EPI_ISL_2303941, EPI_ISL_2303942, EPI_ISL_2303943, EPI_ISL_2303944, EPI_ISL_2303945, EPI_ISL_2303946, EPI_ISL_2303947, EPI_ISL_2405403, EPI_ISL_2405469, EPI_ISL_2405492, EPI_ISL_2405505, EPI_ISL_2405516, EPI_ISL_2405527, EPI_ISL_2405550, EPI_ISL_2405596, EPI_ISL_2406319, EPI_ISL_2406427, EPI_ISL_2476271, EPI_ISL_2476273, EPI_ISL_2476276, EPI_ISL_2609616, EPI_ISL_2609687, EPI_ISL_2610006, EPI_ISL_2610152, EPI_ISL_2610357, EPI_ISL_2610368, EPI_ISL_2610471, EPI_ISL_2610607, EPI_ISL_2610618, EPI_ISL_2610630, EPI_ISL_2610681, EPI_ISL_2610905, EPI_ISL_2672446, EPI_ISL_2672828, EPI_ISL_2672859, EPI_ISL_2672922, EPI_ISL_2673226, EPI_ISL_2673250, EPI_ISL_2673263, EPI_ISL_2673286, EPI_ISL_2788181, EPI_ISL_2788182, EPI_ISL_2788183, EPI_ISL_2788185, EPI_ISL_2788186, EPI_ISL_2788187, EPI_ISL_2788188, EPI_ISL_2862953, EPI_ISL_2862955, EPI_ISL_2862957, EPI_ISL_2981128, EPI_ISL_2981262, EPI_ISL_2981334, EPI_ISL_2981446, EPI_ISL_2981465, EPI_ISL_2981466, EPI_ISL_2981467, EPI_ISL_2981888, EPI_ISL_2981891, EPI_ISL_2981893, EPI_ISL_2981894, EPI_ISL_2981896, EPI_ISL_2981901			
see above	Dutch COVID-19 response team	National Institute for Public Health and the Environment (RIVM)	Adam Meijer; AnneMarie van den Brandt; Annelies Kroneman; Bas van der Veer; Chantal Reusken; Dennis Schmitz; Dirk Eggink; Euníce Then; Florian Zwagemaker; Harry Vennema; James Groot; Jeroen Cremer; Karim Hajji; Kim Freriks; Linda van de Nes; Lisa Wijsman; Lynn Aarts; Melissa van Tuil; Ryanne Jaarsma; Sanne Bos; Sharon van den Brink; Sjoerd Kuiling; on behalf of the national COVID-19 response team
EPI_ISL_1582978, EPI_ISL_1582980	E.S.E. HOSPITAL SAN JOSE DE MAICAO	Instituto Nacional de Salud- Dirección de Investigación en Salud Pública	Carlos Franco-Muñoz; Carmen Osorio; Diana Malo; Diego A. Álvarez-Díaz; Diego Andrés Prada; Gerardo Santamaría; Hector Alejandro Ruiz-Moreno; Jhonnatan Reales-González; Juan Camilo Martinez; Julian Naizaque; Katherine Laiton-Donato; Lisseth Pardo; Magdalena Wiesner; Marcela Mercado-Reyes; Maria T. Herrera-Sepúlveda; Marta Lopez Blanco; Martha Lucia Ospina Martinez; Paola Rojas; Sergio Gomez; Sheryll Corchuelo; Ángela Alarcon Cruz
EPI_ISL_2686819	EHNV	Laboratory of genomics and metagenomics	Claire Bertelli; Damien Jacot; Gilbert Greub; Sébastien Aeby; Trestan Pillonel
EPI_ISL_2617660, EPI_ISL_2617661, EPI_ISL_2617662, EPI_ISL_2617663, EPI_ISL_2617664, EPI_ISL_2617665, EPI_ISL_2617666, EPI_ISL_2617667, EPI_ISL_2617668, EPI_ISL_2617669, EPI_ISL_2617670, EPI_ISL_2617671, EPI_ISL_2617672, EPI_ISL_2617673, EPI_ISL_2617674, EPI_ISL_2617675, EPI_ISL_2617676, EPI_ISL_2617677, EPI_ISL_2617678, EPI_ISL_2617679			
see above	Elling group, Institute of Molecular Biotechnology (IMBA)	Bergthaler laboratory, CeMM Research Center for Molecular Medicine of the Austrian Academy of Sciences	Andreas Bergthaler; Anna Schedl; Bekir Erguner; Benedikt Agerer; Christoph Bock; Fabian Amman; Jan Laine; Lukas Endler; Maelle Le Moing; Martin Senekowitsch; Matthew Thornton; Michael Schuster; Petr Triska; Thomas Penz
EPI_ISL_2634614	Eurofins LifeCodexx GmbH	Robert Koch Institute	
EPI_ISL_2975271	Fimlab Laboratories	Fimlab Laboratories	Bruno Luukinen; Leena Huhti; Mauri Keinänen; Minna Paloniemi; Sara Lehtinen; Tapio Seiskari
EPI_ISL_2860417	Florida Bureau of Public Health Laboratories	Florida Bureau of Public Health Laboratories	Jason Blanton; Sarah Schmedes
EPI_ISL_2365426	GERENCIA DE ASISITENCIA SANITARIA DE SORIA	Instituto de Salud Carlos III	A. Monzón; CARMEN; F. Casas; I. Jiménez; I.ALDEA MANSILLA; M. Sandonís; P. Zaballos; S. Cuesta; S. Iglesias-Caballero; S. Pozo; S. Varona; V. Camarero; Vázquez-Morón
EPI_ISL_2659196	Genetica Molecular and Subdepartamento de Virologia ISP Chile	Instituto de Salud Publica de Chile	Andres Castillo; Barbara Parra; Constanza Campano; Gisselle Barra; Javier Tognarelli; Jorge Fernandez; Karen Orostica; Loredana Arata; Patricia Bustos; Rodrigo Fasce; Soledad Ulloa
EPI_ISL_2339796, EPI_ISL_2339814, EPI_ISL_2339855, EPI_ISL_2584533, EPI_ISL_2584563, EPI_ISL_2584574	Grupo de Investigación en Enfermedades Tropicales del Ejército (GINETEJ), Laboratorio de Referencia e Investigación, Dirección de Sanidad Ejército, Bogotá, Colombia	Centro de Investigaciones en Microbiología y Biotecnología-UR (CIMBIUR), Facultad de Ciencias Naturales, Universidad del Rosario, Bogotá, Colombia	Camilo A. Correa-Cárdenas; Carolina Oliveros; Claudia Méndez; Elizabeth K. Márquez; Frank de los Santos Ortiz; Juan David Ramírez; Julie Pérez; Lorena Albarracín; Luz H. Patiño; Maria Clara Duque; Marina Muñoz; María Teresa Alvarado; Nathalia Ballesteros; Sergio Castañeda; Sergio Gutíerrez-Riveros; Yanira Romero; Zulma Cucunubá
EPI_ISL_2694913, EPI_ISL_2694919, EPI_ISL_2694924, EPI_ISL_2895188	H Garcia de Orta	Instituto Nacional de Saude (INSA)	Borges et al
EPI_ISL_2695087	H Vila Franca Xira	Instituto Nacional de Saude (INSA)	Borges et al
EPI_ISL_1821073	HIC	Instituto Nacional de Salud- Dirección de Investigación en Salud Pública	Carlos Franco-Muñoz; Carmen Osorio; Diana Malo; Diego A. Álvarez-Díaz; Diego Andrés Prada; Gerardo Santamaría; Hector Alejandro Ruiz-Moreno; Jhonnatan Reales-González; Jorge Rivera; Juan Camilo Martinez; Julian Naizaque; Katherine Laiton-Donato; Lisseth Pardo; Magdalena Wiesner; Marcela Mercado-Reyes; Maria T. Herrera-Sepúlveda; Marta Lopez Blanco; Martha Lucia Ospina Martinez; Paola Rojas; Sergio Gomez; Sheryll Corchuelo; Ángela Alarcon Cruz
EPI_ISL_1821071	HIGUERA ESCALANTE	Instituto Nacional de Salud- Dirección de Investigación en Salud Pública	Carlos Franco-Muñoz; Carmen Osorio; Diana Malo; Diego A. Álvarez-Díaz; Diego Andrés Prada; Gerardo Santamaría; Hector Alejandro Ruiz-Moreno; Jhonnatan Reales-González; Jorge Rivera; Juan Camilo Martinez; Julian Naizaque; Katherine Laiton-Donato; Lisseth Pardo; Magdalena Wiesner; Marcela Mercado-Reyes; Maria T. Herrera-Sepúlveda; Marta Lopez Blanco; Martha Lucia Ospina Martinez; Paola Rojas; Sergio Gomez; Sheryll Corchuelo; Ángela Alarcon Cruz
EPI_ISL_2500969	HOSPITAL DEPARTAMENTAL MARIA INMACULADA	Instituto Nacional de Salud- Dirección de Investigación en Salud Pública	Carlos Franco-Muñoz; Carmen Osorio; Diana Malo; Diego A. Álvarez-Díaz; Diego Andrés Prada; Gerardo Santamaría; Hector Alejandro Ruiz-Moreno; Jhonnatan Reales-González; Jorge Rivera; Juan Camilo Martinez; Julian Naizaque; Katherine Laiton-Donato; Lisseth Pardo; Magdalena Wiesner; Marcela Mercado-Reyes; Maria T. Herrera-Sepúlveda; Marta Lopez Blanco; Martha Lucia Ospina Martinez; Paola Rojas; Sergio Gomez; Sheryll Corchuelo; Ángela Alarcon Cruz
EPI_ISL_2657688, EPI_ISL_2827987	HOSPITAL DR. CARLOS LUIS VALVERDE VEGA	Inciensa, Instituto Costarricense de Investigación y Enseñanza en Nutrición y Salud	Adriana Godínez; Claudio Soto-Garita; Estela Cordero; Francisco Duarte; Hebleen Porras; Joselyn Prado & Yahaira Ramos; José Luis Vargas; Mariela Gutiérrez; Melany Calderón
EPI_ISL_2178400, EPI_ISL_2178401	HOSPITAL GENERAL UNIVERSITARIO REINA SOFIA	Instituto de Salud Carlos III	A. Monzón; F. Casas; I. Jiménez; I.NUÑEZ TRIGUEROS; M. Sandonís; Mª LUZ; P. Zaballos; S. Cuesta; S. Iglesias-Caballero; S. Pozo; S. Varona; V. Camarero; Vázquez-Morón
EPI_ISL_2828015	HOSPITAL MEXICO	Inciensa, Instituto Costarricense de Investigación y Enseñanza en Nutrición y Salud	Adriana Godínez; Claudio Soto-Garita; Estela Cordero; Francisco Duarte; Hebleen Porras; Joselyn Prado & Juan Carlos Villalobos; José Luis Vargas; Mariela Gutiérrez; Melany Calderón
EPI_ISL_2674346, EPI_ISL_2674348	HOSPITAL REGIONAL DE LA ORINOQUIA	Instituto Nacional de Salud- Dirección de Investigación en Salud Pública	Carlos Franco-Muñoz; Carmen Osorio; Diana Malo; Diego A. Álvarez-Díaz; Diego Andrés Prada; Gerardo Santamaría; Hector Alejandro Ruiz-Moreno; Jhonnatan Reales-González; Jorge Rivera; Juan Camilo Martinez; Julian Naizaque; Katherine Laiton-Donato; Lisseth Pardo; Magdalena Wiesner; Marcela Mercado-Reyes; Maria T. Herrera-Sepúlveda; Marta Lopez Blanco; Martha Lucia Ospina Martinez; Paola Rojas; Sergio Gomez; Sheryll Corchuelo; Ángela Alarcon Cruz
EPI_ISL_2674347	HOSPITAL REGIONAL ORINOQUIA	Instituto Nacional de Salud- Dirección de Investigación en Salud Pública	Carlos Franco-Muñoz; Carmen Osorio; Diana Malo; Diego A. Álvarez-Díaz; Diego Andrés Prada; Gerardo Santamaría; Hector Alejandro Ruiz-Moreno; Jhonnatan Reales-González; Jorge Rivera; Juan Camilo Martinez; Julian Naizaque; Katherine Laiton-Donato; Lisseth Pardo; Magdalena Wiesner; Marcela Mercado-Reyes; Maria T. Herrera-Sepúlveda; Marta Lopez Blanco; Martha Lucia Ospina Martinez; Paola Rojas; Sergio Gomez; Sheryll Corchuelo; Ángela Alarcon Cruz
EPI_ISL_2178393, EPI_ISL_2178394, EPI_ISL_2178395, EPI_ISL_2178396, EPI_ISL_2178397, EPI_ISL_2178398, EPI_ISL_2178399, EPI_ISL_2178402, EPI_ISL_2178403, EPI_ISL_2178404, EPI_ISL_2178405, EPI_ISL_2227874, EPI_ISL_2227889			
see above	HOSPITAL UNIVERSITARIO VIRGEN DE LA ARRIXACA	Instituto de Salud Carlos III	A. Monzón; F. Casas; I. Jiménez; I. MORENO PARRADO; I.MORENO PARRADO; Iglesias-Caballero; LAURA; M. Camarero; M. Sandonís; P. Zaballos; S. Camarero; S. Cuesta; S. Iglesias-Caballero; S. Pozo; S. Varona; Sandonís; V. Camarero; V. Vázquez-Morón; Vázquez-Morón
EPI_ISL_1581369	Helix/Illumina	Centers for Disease Control and Prevention Division of Viral Diseases, Pathogen Discovery	Adrian Paskey; Alexandre Bolze; Ary Ascencio; Benjamin Rambo-Martin; Brad Sickler; Charlotte Rivera-Garcia; Christine Tran; Christopher Gulvick; Clinton R. Paden; Dakota Howard; Darlene Wagner; David Becker; Dhwani Batra; Duncan MacCannell; Efren Sandoval; Eileen de Feo; Elizabeth Cirulli; Eric Allen; Geraint Levan; James Lu; Jan Antico; Jason Caravas; Jason Nguyen; Jimmy Ramirez; Jingtao Liu; Kara Moser; Kelly Schiabor Barrett; Kim Gietzen; Magnus Isaksson; Marc Laurent; Matthew Schmerer; Matthew Tolentino; Nicole L. Washington; Peter W. Cook; Phil Febbo; Ryan Cho; Scott Sammons; Shannon Wickline; Shatavia Morrison; Sherry Wang; Simon White; Tyler Cassens; William Lee; Yvette Unoarumhi
EPI_ISL_1401468, EPI_ISL_1401469, EPI_ISL_1647948, EPI_ISL_1647949, EPI_ISL_1647956	Hospital General Universitario Gregorio Marañón	Hospital General Universitario Gregorio Marañón	Cristina Rodriguez-Grande; Darío García de Viedma; Laura Pérez-Lago; Patricia Muñoz; Pedro Sola Campoy; Pilar Catalán; Sergio Buenestado Serrano
EPI_ISL_1908878	Hospital Universitari Arnau de Vilanova	Hospital Universitari Vall d'Hebron - Vall d'Hebron Institut de Recerca	Andrés Antón; Ariadna Rando; Carla Castillo; Cristina Andrés; Damir Garcia-Cehic; Josep Quer; Juliana Esperalba; Maria Carmen Martin; Maria Gema Codina; Maria Piñana; Tomàs Pumarola
EPI_ISL_2466275	Hospital Universitari Vall d'Hebron - Vall d'Hebron Institut de Recerca	Hospital Universitari Vall d'Hebron - Vall d'Hebron Institut de Recerca	Alejandra González-Sánchez; Andrés Antón; Ariadna Rando; Carla Castillo; Cristina Andrés; Damir Garcia-Cehic; Josep Quer; Juliana Esperalba; Maria Carmen Martin; Maria Gema Codina; Maria Piñana; Tomàs Pumarola
EPI_ISL_2621160, EPI_ISL_2651224	ICMT-SABANETA	Universidad Nacional de Colombia - Laboratorio Genómico One Health	Andres F. Cardona-Rios; Carlos Franco-Muñoz; Carolina Muñoz-Arango; Celeny Ortiz; Daniel O. Maldonado-Perez; Diego A. Álvarez-Díaz; Hector Alejandro Ruiz-Moreno; Idabely Betancur Ortiz; Jorge E. Osorio; Juan P. Hernandez-Ortiz; Karl A Ciuoderis; Katherine Laiton-Donato; Laura Silvana Perez; Lina M. Hurtado; Marcela Mercado-Reyes; Maria Angélica Maya; Maria Stella López; Rita Almanza Payares; Sandra Ines Cano; Simón Villegas Velásquez
EPI_ISL_2458116, EPI_ISL_2488730, EPI_ISL_2897538, EPI_ISL_2897539, EPI_ISL_2897542, EPI_ISL_2897545, EPI_ISL_2897555, EPI_ISL_2897557, EPI_ISL_3010424, EPI_ISL_3010434, EPI_ISL_3010438, EPI_ISL_3010439, EPI_ISL_3010440, EPI_ISL_3010448, EPI_ISL_3010452, EPI_ISL_3010456, EPI_ISL_3010463			
see above	INSPI-CRN DE INFLUENZA Y OTROS VIRUS RESPIRATORIOS	NIC-INSPI	Alfredo Bruno; Domenica de Mora.; Jimmy Garcés; Johanna Laines; Lizbeth Patiño; Manuel Gonzalez; Maritza Olmedo; Michelle Páez
EPI_ISL_2786928	Inovie AS LABOSUD	Inovie AS GenBio	Florian VERCRUYSSEN / Pauline LAURENT
EPI_ISL_2100436, EPI_ISL_2603836	Institute of Microbiology, Universidad San Francisco de	Institute of Microbiology, Universidad San Francisco de Quito	Belén Prado-Vivar; Bernardo Gutiérrez; Fernanda Zurita; Gabriel Trueba; Juan José Guadalupe; Luis Morales; Michelle Grunauer; Monica Becerra-Wong; Nelson Montalvan; Patricio Rojas-Silva; Paúl Cárdenas; Sully Márquez; Verónica Barragán

	Quito		
EPI_ISL_2562149	Institute of Virology, Biomedical Research Center of the Slovak Academy of Sciences, Bratislava	Faculty of Natural Sciences, Comenius University, Bratislava	Boris Klempa; Brona Brejova; Jozef Nosek; Juraj Kopacek; Kristina Borsova; Lubomira Lukacikova; Martina Lickova; Martina Nebohacova; Monika Slavikova; Sabina Fumacova Havlikova; Tomas Vinar; Viktoria Cabanova; Viktoria Hodorova
EPI_ISL_2500936, EPI_ISL_2500944	Instituto Nacional de Salud	Instituto Nacional de Salud- Dirección de Investigación en Salud Pública	Carlos Franco-Muñoz; Carmen Osorio; Diana Malo; Diego A. Álvarez-Díaz; Diego Andrés Prada; Gerardo Santamaría; Hector Alejandro Ruiz-Moreno; Jhonnatan Reales-González; Jorge Rivera; Juan Camilo Martinez; Julian Naizaque; Katherine Laiton-Donato; Lisseth Pardo; Magdalena Wiesner; Marcela Mercado-Reyes; Maria T. Herrera-Sepúlveda; Marta Lopez Blanco; Martha Lucia Ospina Martinez; Paola Rojas; Sergio Gomez; Sheryll Corchuelo; Ángela Alarcon Cruz
EPI_ISL_2642004, EPI_ISL_2642005, EPI_ISL_2642006, EPI_ISL_2642008, EPI_ISL_2642009, EPI_ISL_2642010	Istituto Zooprofilattico Sperimentale del Mezzogiorno	Telethon Institute of Genetics and Medicine (TIGEM)	Antonio Grimaldi Patrizia Annunziata Francesco Panariello Biancamaria Pierri Claudia Tiberio Teresa Giuliano Valentina Bouche Chiara Colantuono Maria Concetta Cuomo Denise Di Concilio Lucio Di Filippo Anna Manfredi Marcello Salvi Antonio Limone Luigi Atripaldi Pellegrino Cerino Andrea Ballabio Davide Cacchiarelli
EPI_ISL_2864200, EPI_ISL_2877584, EPI_ISL_2886222, EPI_ISL_2886223, EPI_ISL_2886224, EPI_ISL_2886225, EPI_ISL_2886227, EPI_ISL_2886228			
see above	KU Leuven, Rega Institute, Clinical and Epidemiological Virology	KU Leuven, Rega Institute, Clinical and Epidemiological Virology	Bert Vanmechelen; Joan Marti-Carerras; Piet Maes; Tony Wawina-Bokalanga
EPI_ISL_2898054	Karolinska University Hospital Huddinge	Karolinska University Hospital	Annelie Bjerkner; Isak Sylvin; Jan Albert; Karolina Ininbergs; Lina Guerra Blomqvist; Lynda Eneh; Martin Ekman; Martina Wahlund; Robert Dyrdak; Sandra Broddesson; Tanja Normark; Tobias Allander; Valtteri Wirta; Zhibing Yun
EPI_ISL_2657873	LABORATORIO BILOGIA MOLECULAR IMAT SAS	Instituto Nacional de Salud- Dirección de Investigación en Salud Pública	Carlos Franco-Muñoz; Carmen Osorio; Diana Malo; Diego A. Álvarez-Díaz; Diego Andrés Prada; Gerardo Santamaría; Hector Alejandro Ruiz-Moreno; Jhonnatan Reales-González; Jorge Rivera; Juan Camilo Martinez; Julian Naizaque; Katherine Laiton-Donato; Lisseth Pardo; Magdalena Wiesner; Marcela Mercado-Reyes; Maria T. Herrera-Sepúlveda; Marta Lopez Blanco; Martha Lucia Ospina Martinez; Paola Rojas; Sergio Gomez; Sheryll Corchuelo; Ángela Alarcon Cruz
EPI_ISL_2674283	LABORATORIO CONTINENTAL	Instituto Nacional de Salud- Dirección de Investigación en Salud Pública	Carlos Franco-Muñoz; Carmen Osorio; Diana Malo; Diego A. Álvarez-Díaz; Diego Andrés Prada; Gerardo Santamaría; Hector Alejandro Ruiz-Moreno; Jhonnatan Reales-González; Jorge Rivera; Juan Camilo Martinez; Julian Naizaque; Katherine Laiton-Donato; Lisseth Pardo; Magdalena Wiesner; Marcela Mercado-Reyes; Maria T. Herrera-Sepúlveda; Marta Lopez Blanco; Martha Lucia Ospina Martinez; Paola Rojas; Sergio Gomez; Sheryll Corchuelo; Ángela Alarcon Cruz
EPI_ISL_1820934	LABORATORIO DE INVESTIGACION HORMONAL	Instituto Nacional de Salud- Dirección de Investigación en Salud Pública	Carlos Franco-Muñoz; Carmen Osorio; Christian Romero; Diana Malo; Diego A. Álvarez-Díaz; Diego Andrés Prada; Gerardo Santamaría; Hector Alejandro Ruiz-Moreno; Jhonnatan Reales-González; Jorge Rivera; Juan Camilo Martinez; Julian Naizaque; Katherine Laiton-Donato; Lisseth Pardo; Magdalena Wiesner; Marcela Mercado-Reyes; Maria T. Herrera-Sepúlveda; Marta Lopez Blanco; Martha Lucia Ospina Martinez; Paola Rojas; Patricia del Portillo; Sergio Gomez; Sheryll Corchuelo; Ángela Alarcon Cruz
EPI_ISL_2621868	LABORATORIO ECHAVARRIA	Universidad Nacional de Colombia - Laboratorio Genómico One Health	Andres F. Cardona-Rios; Carlos Franco-Muñoz; Carolina Muñoz-Arango; Celeny Ortiz; Daniel O. Maldonado-Perez; Diego A. Álvarez-Díaz; Hector Alejandro Ruiz-Moreno; Idabely Betancur Ortiz; Jorge E. Osorio; Juan P. Hernandez-Ortiz; Karl A Ciuoderis; Katherine Laiton-Donato; Laura Silvana Perez; Lina M. Hurtado; Marcela Mercado-Reyes; Maria Angélica Maya; Maria Stella López; Rita Almanza Payares; Sandra Ines Cano; Simón Villegas Velásquez
EPI_ISL_2362580	LABORATORIO NACY FLOREZ GARCIA	Instituto Nacional de Salud- Dirección de Investigación en Salud Pública	Carlos Franco-Muñoz; Carmen Osorio; Diana Malo; Diego A. Álvarez-Díaz; Diego Andrés Prada; Gerardo Santamaría; Hector Alejandro Ruiz-Moreno; Jhonnatan Reales-González; Jorge Rivera; Juan Camilo Martinez; Julian Naizaque; Katherine Laiton-Donato; Lisseth Pardo; Magdalena Wiesner; Marcela Mercado-Reyes; Maria T. Herrera-Sepúlveda; Marta Lopez Blanco; Martha Lucia Ospina Martinez; Paola Rojas; Sergio Gomez; Sheryll Corchuelo; Ángela Alarcon Cruz
EPI_ISL_2896232	LACEN do Estado de Mato Grosso	Instituto Adolfo Lutz, Interdiciplinary Procedures Center, Strategic Laboratory	Caio Vinicius Dias Lopes; Claudia Regina Gonçalves; Claudio Tavares Sacchi; Karoline Rodrigues Campos
EPI_ISL_1960057	LDSP	Universidad Nacional de Colombia - Laboratorio Genómico One Health	Andres F. Cardona-Rios; Carlos Franco-Muñoz; Carolina Muñoz-Arango; Celeny Ortiz; Daniel O. Maldonado-Perez; Diego A. Álvarez-Díaz; Hector Alejandro Ruiz-Moreno; Idabely Betancur Ortiz; Jorge E. Osorio; Juan P. Hernandez-Ortiz; Karl A Ciuoderis; Katherine Laiton-Donato; Laura Silvana Perez; Lina M. Hurtado; Marcela Mercado-Reyes; Maria Angélica Maya; Maria Stella López; Rita Almanza Payares; Sandra Ines Cano; Simón Villegas Velásquez
EPI_ISL_2827782	LDSP - LAB CLINICO ESPECIALIZADO AIDA ASCENCIO	Instituto Nacional de Salud- Dirección de Investigación en Salud Pública	Carlos Franco-Muñoz; Carmen Osorio; Diana Malo; Diego A. Álvarez-Díaz; Diego Andrés Prada; Gerardo Santamaría; Hector Alejandro Ruiz-Moreno; Jhonnatan Reales-González; Jorge Rivera; Juan Camilo Martinez; Julian Naizaque; Katherine Laiton-Donato; Lisseth Pardo; Magdalena Wiesner; Marcela Mercado-Reyes; Maria T. Herrera-Sepúlveda; Marta Lopez Blanco; Martha Lucia Ospina Martinez; Paola Rojas; Sergio Gomez; Sheryll Corchuelo; Ángela Alarcon Cruz
EPI_ISL_1582996	LDSP BARRANQUILLA	Instituto Nacional de Salud- Dirección de Investigación en Salud Pública	Carlos Franco-Muñoz; Carmen Osorio; Diana Malo; Diego A. Álvarez-Díaz; Diego Andrés Prada; Gerardo Santamaría; Hector Alejandro Ruiz-Moreno; Jhonnatan Reales-González; Juan Camilo Martinez; Julian Naizaque; Katherine Laiton-Donato; Lisseth Pardo; Magdalena Wiesner; Marcela Mercado-Reyes; Maria T. Herrera-Sepúlveda; Marta Lopez Blanco; Martha Lucia Ospina Martinez; Paola Rojas; Sergio Gomez; Sheryll Corchuelo; Ángela Alarcon Cruz
EPI_ISL_2158315, EPI_ISL_2158316, EPI_ISL_2158317	LESP Ciudad de Mexico	Instituto de Diagnostico y Referencia Epidemiologicos (INDRE)	Abril Rodriguez-Maldonado; Ariadna Medina-Benitez; Claudia Wong-Arambula; Ernesto Ramirez-Gonzalez.; Gisela Barrera-Badillo; Irma Lopez-Martinez; Joaquin Quiroz-Mercado; Lucia Hernandez-Rivas; Natividad Cruz-Ortiz; Sergio Rangel-Guerrero; Tatiana Nunez-Garcia; Vanessa Rivero-Arredondo
EPI_ISL_2601972	LKO	Jessa	Berden et al. on behalf of the Jessa_cmdLab
EPI_ISL_2657876	Lab microbiologia FVL	Instituto Nacional de Salud- Dirección de Investigación en Salud Pública	Carlos Franco-Muñoz; Carmen Osorio; Diana Malo; Diego A. Álvarez-Díaz; Diego Andrés Prada; Gerardo Santamaría; Hector Alejandro Ruiz-Moreno; Jhonnatan Reales-González; Jorge Rivera; Juan Camilo Martinez; Julian Naizaque; Katherine Laiton-Donato; Lisseth Pardo; Magdalena Wiesner; Marcela Mercado-Reyes; Maria T. Herrera-Sepúlveda; Marta Lopez Blanco; Martha Lucia Ospina Martinez; Paola Rojas; Sergio Gomez; Sheryll Corchuelo; Ángela Alarcon Cruz
EPI_ISL_2844837	Lab voor klinische biologie	Lab voor klinische biologie	Bruno Verhasselt; Hannelore Hamerlinck; Marija Janevska
EPI_ISL_2382623, EPI_ISL_2466775, EPI_ISL_2466824, EPI_ISL_2694812	Labor Berlin Charité Vivantes GmbH / Institut für Virologie	Charité Universitätsmedizin Berlin, Institut für Virologie/Labor Berlin	Barbara Mühlemann; Christian Drosten; Christine Stephan; Peter Menzel; Rolf Schwarzer; Terry Jones; Victor M Corman
EPI_ISL_2111528, EPI_ISL_2111598	Labor ZOTZ|KLIMAS; MVZ Dusseldorf-Centrum	Robert Koch Institute	
EPI_ISL_2636058, EPI_ISL_2678752	Labor ZOTZ|KLIMAS; MVZ Düsseldorf-Centrum	Robert Koch Institute	
EPI_ISL_2361500, EPI_ISL_2361501	Laboratorio HUB -Azienda Ospedaliero Universitaria - AOU – Cagliari	Laboratorio SPOKE Biologia Molecolare -Azienda Ospedaliero Universitaria - AOU – Cagliari	Alessandra Scano; Ferdinando Coghe; Germano Orrù; Miriam Loddo; Riccardo Cappai; Sara Fais; Valentina Medda
EPI_ISL_2674362	Laboratorio Salud Publica San Andres Islas	Instituto Nacional de Salud- Dirección de Investigación en Salud Pública	Carlos Franco-Muñoz; Carmen Osorio; Diana Malo; Diego A. Álvarez-Díaz; Diego Andrés Prada; Gerardo Santamaría; Hector Alejandro Ruiz-Moreno; Jhonnatan Reales-González; Jorge Rivera; Juan Camilo Martinez; Julian Naizaque; Katherine Laiton-Donato; Lisseth Pardo; Magdalena Wiesner; Marcela Mercado-Reyes; Maria T. Herrera-Sepúlveda; Marta Lopez Blanco; Martha Lucia Ospina Martinez; Paola Rojas; Sergio Gomez; Sheryll Corchuelo; Ángela Alarcon Cruz
EPI_ISL_1582988, EPI_ISL_1582989	Laboratorio de Biología Molecular – Universidad del Magdalena	Instituto Nacional de Salud- Dirección de Investigación en Salud Pública	Carlos Franco-Muñoz; Carmen Osorio; Diana Malo; Diego A. Álvarez-Díaz; Diego Andrés Prada; Gerardo Santamaría; Hector Alejandro Ruiz-Moreno; Jhonnatan Reales-González; Juan Camilo Martinez; Julian Naizaque; Katherine Laiton-Donato; Lisseth Pardo; Magdalena Wiesner; Marcela Mercado-Reyes; Maria T. Herrera-Sepúlveda; Marta Lopez Blanco; Martha Lucia Ospina Martinez; Paola Rojas; Sergio Gomez; Sheryll Corchuelo; Ángela Alarcon Cruz
EPI_ISL_2544236, EPI_ISL_2708977	Laboratorio di Microbiologia	Laboratorio di Microbiologia	Martinetti Lucchini Gladys; Valeria Spina
EPI_ISL_2614407	Lifebrain Covid Labor GmbH	Dept. of Laboratory Medicine	Anna Gschaider; Claudia Weber; Fabian Konig; Harald Esterbauer; Oswald Wagner; Petra Jurkowitsch; Robert Strassl; Sabina Plumer
EPI_ISL_2682959	Lighthouse Lab in Glasgow	Wellcome Sanger Institute for the COVID-19 Genomics UK (COG-UK) Consortium	Anna Dominiczak and Alex Alderton; Carol Clugston; Cordelia Langford; David Gray; David K. Jackson; Dominic Kwiatkowski; Ewan Harrison; Harper VanSteenhouse; Ian Johnston; Jeffrey Barrett; John Sillitoe on behalf of the Wellcome Sanger Institute COVID-19 Surveillance Team; Roberto Amato; Sonia Goncalves; Yumi Kasai
EPI_ISL_2392230, EPI_ISL_2651559, EPI_ISL_2729283, EPI_ISL_2911018	Lighthouse Lab in Milton Keynes	Wellcome Sanger Institute for the COVID-19 Genomics UK (COG-UK) Consortium	Cordelia Langford; David K. Jackson; Dominic Kwiatkowski; Ewan Harrison; Ian Johnston; Jeffrey Barrett; John Sillitoe on behalf of the Wellcome Sanger Institute COVID-19 Surveillance Team; Roberto Amato; Sonia Goncalves; The Lighthouse Lab in Milton Keynes and Alex Alderton
EPI_ISL_2849837	Lighthouse Laboratory Plymouth	Wellcome Sanger Institute for the COVID-19 Genomics UK (COG-UK) Consortium	Cordelia Langford; David K. Jackson; Dominic Kwiatkowski; Ewan Harrison; Ian Johnston; Jeffrey Barrett; John Sillitoe on behalf of the Wellcome Sanger Institute COVID-19 Surveillance Team; Lighthouse Laboratory Plymouth and Alex Alderton; Roberto Amato; Sonia Goncalves
EPI_ISL_2689085	MD PHL	MD PHL	Maryland Department of Health Laboratories Administration
EPI_ISL_2626466	MIRIALIS CLUSES BECHET	CNR Virus des Infections Respiratoires - France SUD	Antonin Bal; Bruno Lina; Bruno Simon; Gregory Destras; Gwendolyne Burfin; Hadrien Regue; Laurence Josset; Martine Valette; Quentin Semanas
EPI_ISL_2918865	Medica	Institute of Medical Virology	Alexandra Trkola; Annette Audigé; Cyril Shah; Gabriela Ziltener; Guido Bloemberg; Jon Huder; Jürg Böni; Kevin Steiner; Maria Grünberg; Maryam Zaheri; Michael Huber; Riccarda Capaul; Stefan Schmutz; Verena Kufner
EPI_ISL_2650339	Microbiologia CATLAB	Can Ruti SARS-CoV-2 Sequencing Hub (HUGTiP/IrsiCaixa/IGTP)	Alba Sánchez; Anna Not; Antoni E Bordoy; Bonaventura Clotet; Cristina Casañ; Cristina Esteban; Francesc Catala-Moll; Gemma Clara; Ignacio Blanco; Marc Noguera-Julian; Maria Casadellà; Mariona Parera; Mercedes Guerrero; Montserrat Giménez; Pere-Joan Cardona; Pilar Armengol; Roger Paredes; Verónica Saludes; and Elisa Martró on behalf of the Can Ruti SARS-CoV-2 Sequencing Hub
EPI_ISL_2926201	Microbiology Department, Laboratori Clínic Metropolitana Nord. Hospital Universitari Germans Trias i Pujol	Can Ruti SARS-CoV-2 Sequencing Hub (HUGTiP/IrsiCaixa/IGTP)	Alba Sánchez; Alexia París; Anna Not; Antoni E Bordoy; Bonaventura Clotet; Cristina Casañ; David Panisello; Francesc Catala-Moll; Gemma Clara; Ignacio Blanco; Laia Soler; Lauro Sumoy; Marc Noguera-Julian; Maria Casadellà; Mariona Parera; Mercedes Guerrero; Montserrat Giménez; Pere-Joan Cardona; Pilar Armengol; Roger Paredes; Verónica Saludes; and Elisa Martró on behalf of the Can Ruti SARS-CoV-2 Sequencing Hub
EPI_ISL_1820898, EPI_ISL_1820899, EPI_ISL_2259146, EPI_ISL_2259158, EPI_ISL_2259204, EPI_ISL_2259205, EPI_ISL_2420759			
see above	Microvida	Microvida	Jaco Verweij; Joep Stohr; Joep Stöhr; Suzan D. Pas
EPI_ISL_2854655	Molecular Diagnostics Pathology Department Mater Dei Hospital Malta	Molecular Diagnostics Pathology Department Mater Dei Hospital Malta	C Cilia; G Zahra; L Grech; M Briffa; R Borg
EPI_ISL_2784724	National Platform bis UMONS/Jolimont	National Platform bis UMONS/Jolimont	Eric Tarantino; Florian Juszczak; François Dufrasne; Gautier Detry; Guillaume Bayon-Vicente; Ruddy Wattiez

EPI_ISL_2932231, EPI_ISL_2932234, EPI_ISL_3011367	National Virus Reference Laboratory	National Virus Reference Laboratory	Charlene Bennett; Cillian F De Gascun; Gabriel Gonzalez; Jonathan Dean; Michael Carr; Zoe Yandle
EPI_ISL_2876172	Ohio Department of Health Laboratory	Ohio Department of Health Laboratory	Allison Black; Brent Lee; Caitlin McDonnell; Eric Brandt; Erica Leasure; Glen McGillivary; Heather Blankenship; Holmes; Jade Mowery; Jennifer; Kelsey Florek; Keoni Omura; Kirtana Ramadugu; Quanta Brown; Stephanie Mccracken; Tyler Payne; and Tammy Bannerman
EPI_ISL_2905966	Originating lab: Wales Specialist Virology Centre Sequencing lab: Pathogen Genomics Unit	Public Health Wales Microbiology Cardiff Wales Specialist Virology Centre	Alec Birchley; Alexander Adams; Amy Gaskin; Angela Marchbank; Bree Gatica-Wilcox; Catherine Moore; Jason Coombes; Joanne Watkins; Joel Southgate; Johnathan Evans; Laura Gifford; Lauren Gilbert; Lee Graham; Malorie Perry; Matthew Bull; Nicole Pacchiarini; Sally Corden; Sara Kumziene-Summerhayes; Sara Rey; Sarah Taylor; Simon Cottrell; Sophie Jones; Tom Connor
EPI_ISL_2621179	PRIME DIAGNOSTICS	Universidad Nacional de Colombia - Laboratorio Genómico One Health	Andres F. Cardona-Rios; Carlos Franco-Muñoz; Carolina Muñoz-Arango; Celeny Ortiz; Daniel O. Maldonado-Perez; Diego A. Álvarez-Díaz; Hector Alejandro Ruiz-Moreno; Idabely Betancur Ortiz; Jorge E. Osorio; Juan P. Hernandez-Ortiz; Karl A Ciuoderis; Katherine Laiton-Donato; Laura Silvana Perez; Lina M. Hurtado; Marcela Mercado-Reyes; Maria Angélica Maya; Maria Stella López; Rita Almanza Payares; Sandra Ines Cano; Simón Villegas Velásquez
EPI_ISL_1385573, EPI_ISL_2965056	Pandemic Response Lab - NYC	Pandemic Response Lab, R&D	Cybill del Castillo; Dylan Law; Haiping Hao; Henry Lee; Isabel Fernandez Escapa; Jon Laurent; Melissa Hopkins; Michael Hammerling; Pradeep Bugga; Shinyoung Clair Kang; Sol Rey; William Ward
EPI_ISL_2455384	Premier Medical Lab	Minnesota Department of Health, Public Health Laboratory	Alexandra Lorentz; Jacob Garfin; Matt Plumb; and Xiong Wang
EPI_ISL_2611881, EPI_ISL_2673694	Public Health Authority of the Slovak Republic	Laboratory of Genomics and Bioinformatics, Comenius University Science Park	Anna Gičová; Diana Rusňáková; Jakub Styk; Jaroslav Budiš; Miroslav Böhmer; Tatiana Sedláčková; Tomáš Szemes
EPI_ISL_2818170, EPI_ISL_2818299, EPI_ISL_2904293	Respiratory Virus Unit, Microbiology Services Colindale, Public Health England	COVID-19 Genomics UK (COG-UK) Consortium	PHE Covid Sequencing Team
EPI_ISL_2933813, EPI_ISL_2933815	SARS-CoV-2 testing team, National Institute of Infectious Diseases	Pathogen Genomics Center, National Institute of Infectious Diseases	Hazuka Y Furihata; Hiromizu Takahashi; Kentaro Itokawa; Makoto Kuroda; Masanori Hashino; Masumichi Saito; Naomi Nojiri; Nozomu Hanaoka; Rina Tanaka; Tsuguto Fujimoto; Tsuyoshi Sekizuka
EPI_ISL_2566238, EPI_ISL_2566240	SC (UCO) Igiene e Sanità Pubblica, ASUGI, Trieste	ARGO Laboratorio Genomica ed Epigenomica	Barbone F; Braida C; Busetti M; D'Agaro P; Dal Monego S; Degasperi M; Licastro D; Maggiore A; Marcello A; Piscianz E; Segat L
EPI_ISL_2178749, EPI_ISL_2178755	Salud Digna	Instituto Nacional de Medicina Genomica	Abraham Campos-Romero; Cedro-Tanda A; Cisneros-Villanueva M; Escobar-Arrazola; Gonzalez-Barrera D; Herrera-Montalvo LA.; Hidalgo-Miranda A; Luna-Ruiz Marco; M; Mendoza-Vargas A; Moreno-Camacho José Luis; Munguia-Garza P; Orjuela-Rodríguez M; Ramirez-Vega O; Rangel-DeLeon D; Reyes-Grajeda JP; Rodriguez-Gallegos Jorge
EPI_ISL_2179709	Servicio Microbiología Hospital La Paz	Servicio Microbiología Hospital La Paz	Elie Dahdouh; Fernando Lázaro; Jesús Mingorance Cruz; Rubén Cáceres
EPI_ISL_2925507, EPI_ISL_2931128	Servicio de Microbiología Clínica (Complejo Hospitalario de Navarra, Pamplona)	Centro de Secuenciación NASERTIC	Ana Miqueleiz; Ana Navascués; Carmen Ezpeleta Baquedano
EPI_ISL_2438026, EPI_ISL_2438034, EPI_ISL_2621190, EPI_ISL_2621192, EPI_ISL_2651211	Somer	Universidad Nacional de Colombia - Laboratorio Genómico One Health	Andres F. Cardona-Rios; Carlos Franco-Muñoz; Carolina Muñoz-Arango; Celeny Ortiz; Daniel O. Maldonado-Perez; Diego A. Álvarez-Díaz; Hector Alejandro Ruiz-Moreno; Idabely Betancur Ortiz; Jorge E. Osorio; Juan P. Hernandez-Ortiz; Karl A Ciuoderis; Katherine Laiton-Donato; Laura Silvana Perez; Lina M. Hurtado; Marcela Mercado-Reyes; Maria Angélica Maya; Maria Stella López; Rita Almanza Payares; Sandra Ines Cano; Simón Villegas Velásquez
EPI_ISL_2875838	Spital Limmattal	Institute of Medical Virology	Alexandra Trkola; Annette Audigé; Catharine Aquino; Cyril Shah; Daniel Ehrsam; Gabriela Ziltener; Guido Bloemberg; Hubert Rehrauer; Isabel Stürmer; Joel Wirz; Jon Huder; Jürg Böni; Kevin Steiner; Maria Grünberg; Maryam Zaheri; Michael Huber; Riccarda Capaul; Stefan Schmutz; Verena Kufner; Weihong Qi
EPI_ISL_2769123, EPI_ISL_2769124	Spital Männedorf AG	Institute of Medical Virology	Alexandra Trkola; Annette Audigé; Cyril Shah; Gabriela Ziltener; Guido Bloemberg; Jon Huder; Jürg Böni; Kevin Steiner; Maria Grünberg; Maryam Zaheri; Michael Huber; Riccarda Capaul; Stefan Schmutz; Verena Kufner
EPI_ISL_2438017, EPI_ISL_2621151	Synlab	Universidad Nacional de Colombia - Laboratorio Genómico One Health	Andres F. Cardona-Rios; Carlos Franco-Muñoz; Carolina Muñoz-Arango; Celeny Ortiz; Daniel O. Maldonado-Perez; Diego A. Álvarez-Díaz; Hector Alejandro Ruiz-Moreno; Idabely Betancur Ortiz; Jorge E. Osorio; Juan P. Hernandez-Ortiz; Karl A Ciuoderis; Katherine Laiton-Donato; Laura Silvana Perez; Lina M. Hurtado; Marcela Mercado-Reyes; Maria Angélica Maya; Maria Stella López; Rita Almanza Payares; Sandra Ines Cano; Simón Villegas Velásquez
EPI_ISL_2651119, EPI_ISL_2651120	Temporary Specimen Collection Centre at the AsiaWorld-Expo	Hong Kong Department of Health	Alan K.L. Tsang; Dominic N.C. Tsang; Edman T.K. Lam; Ken H.L. Ng; Peter C.W. Yip; Rickjason C.W. Chan
EPI_ISL_2964673	The Caribbean Public Health Agency	Carrington Lab, Department of Preclinical Sciences, Faculty of Medical Sciences, The University of the West Indies, St Augustine Campus	Anushka Ramjag; Arianne Brown-Jordan; Avery Hinds; Christine V. F. Carrington; Gabriel Escobar; Kenneth George; Nikita S. D. Sahadeo; Nuno Faria; Oliver Pybus; Risha Singh; Sarah Hill; SueMin Nathaniel; Vernie Ramkissoon
EPI_ISL_2993634, EPI_ISL_2993635	The Centre for Clinical Infection & Diagnostics Research, KCL	The Centre for Clinical Infection & Diagnostics Research, KCL	Adela Alcolea-Medina; Gaia Nebbia; Jonathan D Edgeworth; Luke B Snell; Penny R Cliff; Rahul Batra; Sam T Douthwaite; Themoula Charalampous
EPI_ISL_2681041	UM im. Karola Marcinkowskiego w Poznaniu Laboratorium UCA_Covid-19 Centrum Biologii Medycznej	1. Tricity SARS-CoV-2 sequencing consortium: University of Gdansk, Medical University of Gdansk, Vaxican Ltd., Invicta Ltd. 2. National Institute of Public Health - National Institute of Hygiene, Warsaw, Poland	Celina Cybulska; Karolina Gackowska; Katarzyna Groth; Katarzyna Zacharczuk; Krystyna Bienkowska Szewczyk; Lukasz Rabalski; Maciej Grzybek; Maciej Kosinski; Magdalena Nowakowska; Marcin Lubocki; Małgorzata Sadkowska-Todys; Tomasz Wołkowicz
EPI_ISL_2136319, EPI_ISL_2321609	UNIGEM	Universidad Nacional de Colombia - Laboratorio Genómico One Health	Andres F. Cardona-Rios; Carlos Franco-Muñoz; Carolina Muñoz-Arango; Celeny Ortiz; Daniel O. Maldonado-Perez; Diego A. Álvarez-Díaz; Hector Alejandro Ruiz-Moreno; Idabely Betancur Ortiz; Jorge E. Osorio; Juan P. Hernandez-Ortiz; Karl A Ciuoderis; Katherine Laiton-Donato; Laura Silvana Perez; Lina M. Hurtado; Marcela Mercado-Reyes; Maria Angélica Maya; Maria Stella López; Rita Almanza Payares; Sandra Ines Cano; Simón Villegas Velásquez
EPI_ISL_2628889, EPI_ISL_2628969, EPI_ISL_2645992, EPI_ISL_2810514, EPI_ISL_2810516, EPI_ISL_2810523, EPI_ISL_2895450			
see above	UNILABS	Instituto Nacional de Saude (INSA)	Borges et al
EPI_ISL_2810398	UNILABS	Instituto Nacional de Saude (INSA) and BioSystems & Integrative Sciences Institute (BioISI) Genomics Unit, FCUL	Borges et al
EPI_ISL_2796029, EPI_ISL_2796098, EPI_ISL_2796104	UNILABS	Instituto Nacional de Saude (INSA) and Instituto Gulbenkian de Ciencia (IGC)	Borges et al
EPI_ISL_1820935	UNIVERSIDAD DE CARTAGENA	Instituto Nacional de Salud- Dirección de Investigación en Salud Pública	Carlos Franco-Muñoz; Carmen Osorio; Christian Romero; Diana Malo; Diego A. Álvarez-Díaz; Diego Andrés Prada; Gerardo Santamaría; Hector Alejandro Ruiz-Moreno; Jhonnatan Reales-González; Jorge Rivera; Juan Camilo Martinez; Julian Naizaque; Katherine Laiton-Donato; Lisseth Pardo; Magdalena Wiesner; Marcela Mercado-Reyes; Maria T. Herrera-Sepúlveda; Marta Lopez Blanco; Martha Lucia Ospina Martinez; Paola Rojas; Patricia del Portillo; Sergio Gomez; Sheryll Corchuelo; Ángela Alarcon Cruz
EPI_ISL_1820926	UNIVERSIDAD DE Magdalena	Instituto Nacional de Salud- Dirección de Investigación en Salud Pública	Carlos Franco-Muñoz; Carmen Osorio; Christian Romero; Diana Malo; Diego A. Álvarez-Díaz; Diego Andrés Prada; Gerardo Santamaría; Hector Alejandro Ruiz-Moreno; Jhonnatan Reales-González; Jorge Rivera; Juan Camilo Martinez; Julian Naizaque; Katherine Laiton-Donato; Lisseth Pardo; Magdalena Wiesner; Marcela Mercado-Reyes; Maria T. Herrera-Sepúlveda; Marta Lopez Blanco; Martha Lucia Ospina Martinez; Paola Rojas; Patricia del Portillo; Sergio Gomez; Sheryll Corchuelo; Ángela Alarcon Cruz
EPI_ISL_431116	Unidad de Investigacion Biomedica de Zacatecas (UIBZ)	Unidad de Genomica Avanzada	; Alejandra Garcia-Gasca; Alejandra Hernandez-Teran; Alejandro Sanchez-Flores; Alfredo Herrera-Estrella; Alicia Ocaña-Mondragon; Andreu Comas-Garcia; Angel Gustavo Salas-Lais; Antonio Loza Roman; Bernardo Martinez-Miguel; Blanca Taboada; Brenda Irasema Maldonado-Meza; Bruno Gomez-Gil; Carla Ivon Herrera-Najera; Carlos F. Arias; Celia Boukadida; Celida Duque Molina; Celida Martinez- Rodriguez; Clara Esperanza Santacruz-Tinoco; Concepcion Grajales-Muñiz; Consorcio Mexicano de Vigilancia Genomica (CoViGen-Mex). Authors (in alphabetical order): Julio Elias Alvarado-Yaah; Cristobal Chaidez-Quiroz; Daniel Fregoso-Rueda; Daniel Lira Morales; Eduardo Becerril-Vargas; Fernando Fontove-Herrera; Fidencio Mejia-Nepomuceno; Francisco Pulido; Gloria Elena Espinosa-Ayala; Gloria Maria Molina-Salinas; Gloria Vazquez; Hector Esteban Paz-Juarez; Hector Montoya-Fuentes; Helen Haydee Fernanda Ramirez-Plascencia; Irvin Gonzalez-Lopez; Jean Pierre Gonzalez; Jesus Hernandez; Joel Armando Vazquez-Perez.; Jorge Salas-Hernandez; Jose Antonio Enciso-Moreno; Jose Arturo Martinez-Orozco; Jose Esteban Muñoz-Medina; Jose de Jesus Nuñez-Contreras; Juan Bautista Chale-Dzul; Julissa Enciso-Ibarra; Luis Alberto Ochoa-Carrera; Margarita Matias-Florentino; Maria Guadalupe Santiago-Mauricio; Maria Guadalupe de Jesus Mireles-Rivera; Mario Mujica-Sanchez; Marissa Perez-Garcia; Nelly Selem-Mojica; Pavel Isa; Ricardo Ciria Merce; Ricardo Grande; Rosa Maria Gutierrez Rios; Santiago avila-Rios; Selene Zarate; Susana Lopez; Veronica Mata-Haro; Victor Eduardo Garcia-Arias; Victor Hugo Borja-Aburto
EPI_ISL_2438098	Universidad Nacional de Colombia - Laboratorio Genómico One Health	Universidad Nacional de Colombia - Laboratorio Genómico One Health	Andres F. Cardona-Rios; Carlos Franco-Muñoz; Carolina Muñoz-Arango; Celeny Ortiz; Daniel O. Maldonado-Perez; Diego A. Álvarez-Díaz; Hector Alejandro Ruiz-Moreno; Idabely Betancur Ortiz; Jorge E. Osorio; Juan P. Hernandez-Ortiz; Karl A Ciuoderis; Katherine Laiton-Donato; Laura Silvana Perez; Lina M. Hurtado; Marcela Mercado-Reyes; Maria Angélica Maya; Maria Stella López; Rita Almanza Payares; Sandra Ines Cano; Simón Villegas Velásquez
EPI_ISL_1220045	Universidad de Magdalena	Instituto Nacional de Salud- Dirección de Investigación en Salud Pública	Carlos Franco-Muñoz; Diego A. Álvarez-Díaz; Diego Andrés Prada; Gerardo Santamaría; Hector Alejandro Ruiz-Moreno; Jhonnatan Reales-González; Julian Naizaque; Katherine Laiton-Donato; Magdalena Wiesner; Marcela Mercado-Reyes.; Maria T. Herrera-Sepúlveda; Martha Lucia Ospina Martínez; Sheryll Corchuelo
EPI_ISL_1582993	Universidad del Atlántico Laboratorio de Investigación en Biología Molecular	Instituto Nacional de Salud- Dirección de Investigación en Salud Pública	Carlos Franco-Muñoz; Carmen Osorio; Diana Malo; Diego A. Álvarez-Díaz; Diego Andrés Prada; Gerardo Santamaría; Hector Alejandro Ruiz-Moreno; Jhonnatan Reales-González; Juan Camilo Martinez; Julian Naizaque; Katherine Laiton-Donato; Lisseth Pardo; Magdalena Wiesner; Marcela Mercado-Reyes; Maria T. Herrera-Sepúlveda; Marta Lopez Blanco; Martha Lucia Ospina Martinez; Paola Rojas; Sergio Gomez; Sheryll Corchuelo; Ángela Alarcon Cruz
EPI_ISL_1424054, EPI_ISL_1424055	Universidad del Magdalena	Instituto Nacional de Salud- Dirección de Investigación en Salud Pública	Carlos Franco-Muñoz; Diego A. Álvarez-Díaz; Diego Andrés Prada; Gerardo Santamaría; Hector Alejandro Ruiz-Moreno; Jhonnatan Reales-González; Julian Naizaque; Katherine Laiton-Donato; Magdalena Wiesner; Marcela Mercado-Reyes.; Maria T. Herrera-Sepúlveda; Martha Lucia Ospina Martínez; Sheryll Corchuelo
EPI_ISL_2355434, EPI_ISL_2517670	University College London, Great Ormond Street Hospital for Children NHS Foundation Trust, Imperial College Healthcare NHS Trust	COVID-19 Genomics UK (COG-UK) Consortium	Alison Holmes; Charlotte Williams; Helena Tutill; Jacqueline Findlay; James Price; Judith Breuer; Julianne Brown; Kathryn Harris; Leysa Forrest; Mark Kristiansen; Paola Niola; Paola Resende Silva; Patricia Dyal; Paul Randell; Rachel Williams; Samuel Weeks; Sergi Castellano; Sunando Roy; Tony Brooks; Yasmin Panchbhaya
EPI_ISL_2405310, EPI_ISL_2405311, EPI_ISL_2601042, EPI_ISL_2601071, EPI_ISL_2601076, EPI_ISL_2601085, EPI_ISL_2885486, EPI_ISL_2885495, EPI_ISL_2885496, EPI_ISL_2981946			

see above	University Hospitals of Geneva, Laboratory of Virology	HUG, Laboratory of Virology and the Health2030 Genome Center	Ana Rita Goncalves; Deborah Penet; Emmanouil Dermitzakis; Henri Pegeot; Ioannis Xenarios; Keith Harshman; Laurent Kaiser; Lorenzo Cerutti; Melyssa Elies; Samuel Cordey
EPI_ISL_2769134, EPI_ISL_2769148, EPI_ISL_2918872, EPI_ISL_2978325	UniversitätsSpital Zürich	Institute of Medical Virology	Alexandra Trkola; Annette Audigé; Cyril Shah; Gabriela Ziltener; Guido Bloemberg; Jon Huder; Jürg Böni; Kevin Steiner; Maria Grünberg; Maryam Zaheri; Michael Huber; Riccarda Capaul; Stefan Schmutz; Verena Kufner
EPI_ISL_2390184	Universitätsklinikum Bonn - Institut für Virologie	Robert Koch Institute	
EPI_ISL_1820955	VIROLOGIA INS DRSP	Instituto Nacional de Salud- Dirección de Investigación en Salud Pública	Carlos Franco-Muñoz; Carmen Osorio; Christian Romero; Diana Malo; Diego A. Álvarez-Díaz; Diego Andrés Prada; Gerardo Santamaría; Hector Alejandro Ruiz-Moreno; Jhonnatan Reales-González; Jorge Rivera; Juan Camilo Martinez; Julian Naizaque; Katherine Laiton-Donato; Lisseth Pardo; Magdalena Wiesner; Marcela Mercado-Reyes; Maria T. Herrera-Sepúlveda; Marta Lopez Blanco; Martha Lucia Ospina Martinez; Paola Rojas; Patricia del Portillo; Sergio Gomez; Sheryll Corchuelo; Ángela Alarcon Cruz
EPI_ISL_2379728, EPI_ISL_2420116, EPI_ISL_2521342, EPI_ISL_2521344	Valais Hospital, Central Institute	Valais Hospital, Central Institute	Alexis Dumoulin; Deborah Penet; Emmanouil Dermitzakis; Henri Pegeot; Ioannis Xenarios; Keith Harshman; Lorenzo Cerutti; Melyssa Elies
EPI_ISL_2152100, EPI_ISL_2544315, EPI_ISL_2662426, EPI_ISL_2724442, EPI_ISL_2820613	Viollier AG	Department of Biosystems Science and Engineering, ETH Zürich	Andrea Patrignani; Andrea Patrizia Salzmann; Andreia Cabral de Gouvea; Catharine Aquino; Chaoran Chen; Christian Beisel; Christiane Beckmann; Christoph Noppen; Daniel Ehrsam; David Dreifuss; Doris Popovic; Elodie Burcklen; Griffin White; Henriette Kurth; Ina Nissen; Isabel Stürmer; Ivan Topolsky; Jay Tracy; Katharina Jahn; Kim Philipp Jablonski; Lara Fuhrmann; Laura Neff; Lennart Opitz; Maria Domenica Moccia; Maurice Redondo; Mirjam Feldkamp; Natascha Santacroce; Niko Beerenwinkel; Noemie Santamaria de Souza; Olivier Kobel; Philipp Jablonski; Ralph Schlapbach; Rebecca Denes; Sarah Nadeau; Simon Grüter; Sophie Seidel; Tanja Stadler; Timothy Sykes
EPI_ISL_2833685	Zentrallabor Zürich	Institute of Medical Virology	Alexandra Trkola; Annette Audigé; Cyril Shah; Gabriela Ziltener; Guido Bloemberg; Jon Huder; Jürg Böni; Kevin Steiner; Maria Grünberg; Maryam Zaheri; Michael Huber; Riccarda Capaul; Stefan Schmutz; Verena Kufner
EPI_ISL_2600375	cerballiance-IDF	Cerba lab	Aude Lessenne; Bénédicte Roquebert; Emmanuel Lecorche; Kader Merah; Laura Verdurme; Patrice Herisson; Sabine Trombert-Paolantoni; Stéphanie Haïm-Boukobza; Thierry Collin
